# Transcriptional responses to polycyclic aromatic hydrocarbon-induced stress in *Arabidopsis thaliana* reveal the involvement of hormone and defense signaling pathways

**DOI:** 10.1186/1471-2229-10-59

**Published:** 2010-04-07

**Authors:** David Weisman, Merianne Alkio, Adán Colón-Carmona

**Affiliations:** 1 Department of Biology, University of Massachusetts Boston, 100 Morrissey Blvd, Boston, MA 02125, USA; 2 Institute of Biological Production Systems, Fruit Science Section, Leibniz University Hannover, Herrenhäuser Str 2, D-30419 Hannover, Germany

## Abstract

**Background:**

Polycyclic aromatic hydrocarbons (PAHs) are toxic, widely-distributed, environmentally persistent, and carcinogenic byproducts of carbon-based fuel combustion. Previously, plant studies have shown that PAHs induce oxidative stress, reduce growth, and cause leaf deformation as well as tissue necrosis. To understand the transcriptional changes that occur during these processes, we performed microarray experiments on *Arabidopsis thaliana *L. under phenanthrene treatment, and compared the results to published Arabidopsis microarray data representing a variety of stress and hormone treatments. In addition, to probe hormonal aspects of PAH stress, we assayed transgenic ethylene-inducible reporter plants as well as ethylene pathway mutants under phenanthrene treatment.

**Results:**

Microarray results revealed numerous perturbations in signaling and metabolic pathways that regulate reactive oxygen species (ROS) and responses related to pathogen defense. A number of glutathione S-transferases that may tag xenobiotics for transport to the vacuole were upregulated. Comparative microarray analyses indicated that the phenanthrene response was closely related to other ROS conditions, including pathogen defense conditions. The ethylene-inducible transgenic reporters were activated by phenanthrene. Mutant experiments showed that PAH inhibits growth through an ethylene-independent pathway, as PAH-treated ethylene-insensitive *etr1-4 *mutants exhibited a greater growth reduction than WT. Further, phenanthrene-treated, constitutive ethylene signaling mutants had longer roots than the untreated control plants, indicating that the PAH inhibits parts of the ethylene signaling pathway.

**Conclusions:**

This study identified major physiological systems that participate in the PAH-induced stress response in Arabidopsis. At the transcriptional level, the results identify specific gene targets that will be valuable in finding lead compounds and engineering increased tolerance. Collectively, the results open a number of new avenues for researching and improving plant resilience and PAH phytoremediation.

## Background

Polycyclic aromatic hydrocarbons (PAH) are a family of persistent, hydrophobic environmental toxins that originate from the incomplete combustion of carbon-based fuels as well as from the release of petroleum into the environment [1,2]. As PAHs are potent carcinogens in humans [3,4], remediation of PAH contamination is an ongoing endeavor. Traditionally, removal of pollutants from soil is a disruptive and costly physical process; consequently, there is strong interest in applying phytoremediation, the use of plants to sequester, volatilize, or degrade pollutants [5,6].

An idealized plant used for PAH removal would uptake large amounts of the pollutant into the root system, transport the molecules to cellular compartments, metabolize the pollutant, and utilize or volatilize the nontoxic byproducts. In practice, these processes are rate- or capacity-limited, thereby limiting the net removal of PAH from soil. Over time, stress from pollutants and their byproducts can cause cumulative plant damage, further reducing pollutant flux through the system. With the goals of identifying and relaxing these constraints, theoretical and applied research is ongoing. As an example of enhanced arsenic phytoremediation, a series of experiments identified limiting processes and introduced transgenic constructs into Arabidopsis, resulting in greatly increased uptake and tolerance of the pollutant [7-9]. Unlike in arsenic phytoremediation, where plant hyperaccumulation followed by harvesting is the goal, phytoremediation of PAHs could ultimately lead to complete degradation of the organic compounds.

Following PAH treatment, plants exhibit a variety of stresses. Previous studies have shown that PAHs cause trichome and leaf deformations, accumulation of H_2_O_2_, oxidative stress, cell death, upregulation of antioxidant systems, and reduced plant growth [1,10-14]. In many regards, these symptoms broadly resemble the pathogenic hypersensitive response (HR) [14]. While there is substantial evidence of oxidative stress, the signaling and biochemical changes leading to the complex PAH symptoms are unknown.

The phytohormone ethylene has long been known to play central roles in oxidative stress responses and cell death [15], in plant growth inhibition [16], and in abiotic as well as pathogen responses [17,18]. These broad parallels, as well as the observation that the ethylene-responsive gene *GSTF2 *is upregulated in PAH-treated Arabidopsis [14,19], suggest that ethylene signaling may play a role in the PAH stress response. To better understand these areas, this study performed DNA microarray experiments to measure global transcriptional changes in Arabidopsis when treated with the three-ringed PAH phenanthrene. In addition, possible roles of ethylene signaling were investigated using ethylene-responsive reporter plants, ethylene production mutants, ethylene signaling mutants, and exogenous application of an ethylene precursor.

## Results

### Transcriptional responses to phenanthrene

To assess differential transcript levels of PAH-treated Arabidopsis, microarray experiments were performed on wild type (WT) whole plants grown for 21 days on sterile medium containing 0 mM or 0.25 mM phenanthrene. The PAH treatment level is comparable to levels found in polluted land and water sites [10]. A statistically significant set of transcripts was selected using a Benjamini and Hochberg false discovery rate (FDR) of 0.05. Of these, high-stringency biological relevance was defined as the genes with greater than two-fold change in either direction, resulting in 1031 phenanthrene-responsive transcripts that were analyzed further. The full microarray dataset is available in Additional File 1, and the differentially-expressed subset is available in Additional File 2.

To elucidate classes of transcripts affected by phenanthrene, gene ontology (GO) analyses were performed on the 1031 differentially-expressed genes. A summary of this analysis is available in Additional File 3. Complementing the GO analysis, MapMan figures (Additional File 4) were produced to visualize phenanthrene-induced changes in cellular processes. Additional File 5 highlights relevant transcriptional changes related to stress, hormone signaling, and other selected processes.

A striking feature is the downregulation of photosynthesis-related mRNA levels (Additional File 3, Additional File 4a,b). In concert with the reduced photosynthesis, chlorophyll and carotenoid biosynthesis as well as protein targeting to the chloroplasts were reduced (Additional File 3, Additional File 4c,d). Downregulated processes further included protein biosynthesis and gluconeogenesis (Additional File 3).

Of the differentially-expressed transcripts, there is a strong overrepresentation of genes involved in biotic and abiotic stresses, oxidative stress, wounding, immunity, and defense responses (Additional File 3, Additional File 4e). For instance, the genes coding for the ethylene-inducible defense response proteins *PDF1.2a *and *PDF1.2b *[20] were strongly upregulated on the microarray (Additional File 5). The pathogenesis related (PR) gene *PR-1*, which is the marker gene for systemic acquired resistance (SAR) was upregulated over 200-fold. *PR-1 *is induced by salicylic acid (SA) but does not require ethylene or jasmonate [21]. Transcript levels of *PR-2*, -3 (*B-CHI*, basic chitinase), -4, and -5 were also increased by phenanthrene (Additional File 1, Additional File 5, and Additional File 6).

A variety of antioxidant and detoxification systems were affected (Additional File 3 and Additional File 4f). The transcript level of the arginine decarboxylase *ADC2*, a key enzyme in polyamine synthesis, was increased on the PAH microarray (Additional File 2). Twelve microarray probes representing glutathione transferases (GST), enzymes that tag xenobiotics with glutathione for transport into the vacuole [22,23], reported significant increases (Additional File 2 and Additional File 5). For instance, the GST *AtGSTU24 *was upregulated on phenanthrene. Additionally, the microarray probe that recognized *AtGSTF2 *(*At4g02520*) and *AtGSTF3 *(*At2g02930*) indicated a 3.7-fold increase of the transcripts on phenanthrene. Similarly, the probe that binds the GSTs *At1g02920 *and *At1g02930 *indicated 12-fold upregulation of these genes. Among the phenanthrene responsive GSTs, *AtGSTU24 *has previously been shown to be sharply and rapidly induced by the herbicides acetochlor and metolachlor, as well as the explosives 2,4,6-trinitrotoluene and hexahydro-1,3,5-trinitro-1,3,5-triazine [24]. Along similar lines, UDP-glucoronosyl and UDP-glucosyl transferase *UGT74F2 *(*At2g43820*, Additional File 5) was strongly upregulated by phenanthrene. This gene was constitutively upregulated in antioxidant loss-of-function mutants [25], which is consistent with upregulation in response to reactive oxygen species (ROS). Activation of the secretory system is further indicated by upregulation of protein targeting through the ER and the Golgi apparatus (Additional File 4d). Inversely, mRNA levels of several antioxidant genes were diminished (Additional File 4e). Downregulated mRNAs include the catalases (Additional File 1) *CAT1*, *CAT3*, as well as *CAT2 *which is consistent with previous RT-PCR data [10]. The ascorbate peroxidases *APX4 *(Additional File 5) and *TAPX *(Additional File 2) as well as the superoxide-dismutase *FSD1 *(Additional File 5) were also downregulated on phenanthrene.

Expression levels of many hormone-responsive genes were changed: Generally, jasmonic acid (JA), SA, or abscisic acid responsive genes were induced, whereas gibberellic acid, brassinolide or auxin responsive genes were repressed (Additional File 3, Additional File 4f, Additional File 5). Expression of many typical ethylene-inducible genes was induced, including defensins, *HEL*, GSTs and basic chitinase (Additional File 5). However, other typical ethylene-responsive genes, such as *HLS1*, were unaffected. Two genes of the ethylene biosynthesis pathway were downregulated: *ACS6*, an aminocyclopropane-1-carboxylic acid (ACC) synthase, and *ACO2*, an 1-aminocyclopropane-1-carboxylic acid (ACC) oxidase. Of the 145 putative ethylene-regulated AP2/EREBP transcription factor genes [26], 126 are represented on the microarray (Additional File 1), and mRNA levels of ten of these were more than two-fold affected by phenanthrene. Interestingly, the ethylene response factor *ERF1-1*, which integrates ethylene and JA signals [27], was significantly upregulated in the PAH dataset (Additional File 1). An overview of the transcriptional changes in hormonal and other regulatory processes is given in Additional File 3 and Additional File 4f.

### Comparison between phenanthrene and other stress and hormone treatments

**Figure 1:**
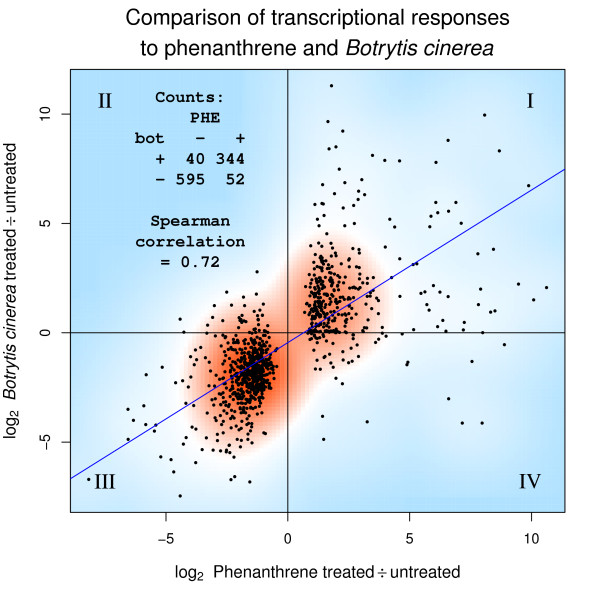
**Comparison of transcriptional responses to phenanthrene and *Botrytis cinerea***. Scatter plot of 1031 differentially-expressed transcripts from microarray data of 21-day old phenanthrene-treated Arabidopsis plants, compared to *B. cinerea *treatment. Counts represent the number of transcripts up (+) or down (-) regulated in each condition. Roman numerals identify the quadrants described in the text.

The gene ontology and MapMan analyses (Additional File 3 and Additional File 4e) of the transcriptional profile indicate that the PAH response shares commonality with biotic stress responses. Illustrating this relationship, Figure 1 compares the phenanthrene dataset to the treatment with the pathogenic fungus *Botrytis cinerea*, and indicates a strong correlation (*ρ *= 0.72) between the two treatments. In Figure 1, Quadrants I and III contain the transcripts that were jointly up- or downregulated on both treatments. The vast majority of the phenanthrene responsive transcripts fall into these categories. For instance, the cell wall expansins *AtEXP1*, *AtEXP8 *[14], and *AtEXP11 *were downregulated on both treatments (Quadrant III). Quadrant II contains transcripts that were downregulated by phenanthrene but upregulated by the *B. cinerea *fungal attack, and includes the ethylene biosynthesis gene *ACS6*. Inversely, Quadrant IV contains transcripts that are highly expressed on phenanthrene and diminished by the pathogen, including the cell wall expansin *AtEXP4*, *AtNAP2 *(*POP1*), which encodes a NAP-type ABC transporter, and *At1g47400 *of unknown function.

**Figure 2:**
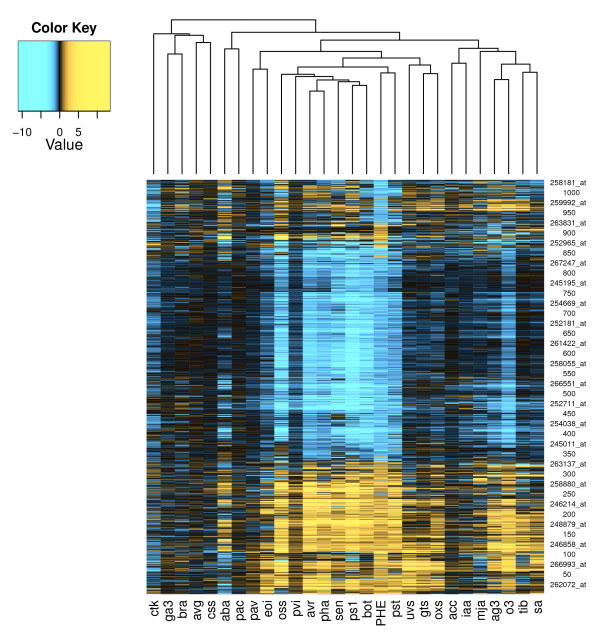
**Gene and experiment clustering of phenanthrene microarray dataset**. Hierarchical clusterings of genes and experiments, created from phenanthrene and published Arabidopsis microarray datasets. Values in the Color Key are log_2_(treated/control) microarray intensity values. Experiment codes are listed in Table 1, and the heatmap is detailed further in Additional File 6.

To further compare the PAH response with other experimental conditions, the phenanthrene dataset was clustered with a variety of published microarray datasets measuring responses to biotic, abiotic, chemical, and physical stresses as well as hormone and hormone inhibitor treatments. Table 1 shows correlations between the phenanthrene microarray and other experimental conditions. The heatmap in Figure 2 shows the results from clustering genes and experimental conditions. The complete dataset of the heatmap is available in Additional File 6. The manifest clusters in the heatmap show strong similarity with various strains of *Pseudomonas syringae*, as well as the fungi *B. cinerea *and *Erysiphe orontii*. Ozone, osmotic, and oxidative stresses, as well as senescence, also correlated with the phenanthrene response.

**Table 1:** Transcriptional correlations between phenanthrene and other treatments.

Code	Treatment	Correlation	NASC
PHE	Phenanthrene (3 w, 3 w)	1.00	
bot	*Botrytis cinerea *(4 w, 48 h)	0.72	167
pst	*P. syringae patovar tomato *(5 w, 24 h)	0.71	330
o3	Ozone (2 w, 6 h)	0.67	26
avr	*P. syringae avrRpm1 *(5 w, 24 h)	0.66	120
pha	*P. syringae phaseolicola *(5 w, 24 h)	0.64	120
oss	Osmotic stress (2 w, 24 h; shoot)	0.64	139
ps1	*P. syringae DC3000 *(5 w, 24 h)	0.63	120
sen	None; senescence (mid flowering; leaves)	0.62	52
pvi	*P. syringae ES4326 *(4 w, 48 h)	0.60	168
sa	Salicylic acid (1 w, 3 h)	0.55	192
eoi	*Erysiphe orontii *(5 w, 5 d)	0.52	169
oxs	Oxidative stress (2 w, 24 h; shoot)	0.51	139
ag3	AgNO_3 _(1 w, 3 h)	0.48	188
gts	Genotoxicity (2 w, 24 h; shoot)	0.46	142
uvs	UV radiation (2 w, 24 h; shoot)	0.42	144
tib	2,3,5-triiodobenzoic acid (TIBA; 1 w, 3 h)	0.36	186
pav	*P. syringae ES4326 avrRpt2 *(4 w, 48 h)	0.32	168
iaa	Indoleacetic acid (IAA; 1 w, 3 h)	0.27	175
mja	Methyl jasmonate (1 w, 3 h)	0.26	174
ga3	Gibberellic aid (1 w, 3 h)	0.20	177
aba	Abscisic acid (1 w, 3 h)	0.19	176
ctk	Cytokinin (3 w, 3 h)	0.18	181
acc	1-aminocycloprop. 1-carbox. acid (1 w, 3 h)	0.18	172
pac	Paclobutrazol (1 w, 12 h)	-0.01	185
avg	Aminoethoxyvinylglycine (1 w, 3 h)	-0.01	188
bra	Brassinolide (1 w, 3 h)	-0.03	178
css	Caesium-137 (shoot; 3 w, 2 w)	-0.07	324

Correlations between microarray profiles of 1031 phenanthrene-responsive genes under phenanthrene treatment and 27 other stress and hormone treatments. Plant age in weeks and duration of treatment are given in parenthesis; whole plant tissue was analyzed if not otherwise indicated. Correlation represents Spearman correlation (*ρ*) with the phenanthrene treatment. NASC is the Nottingham Arabidopsis Stock Centre microarray reference number.

In contrast with the phenanthrene-induced downregulation of *ACS6*, the transcript was upregulated by *B. cinerea *attack and in other biotic stresses, oxidative stress, O_3_, SA, genotoxicity, indoleacetic acid (IAA), TIBA (inhibitor of polar auxin transport) and AgNO_3 _(inhibitor of ethylene signaling) treatments. *WRKY40*, a member of a transcription factor family that frequently plays critical roles in stress responses [28], followed a similar pattern. *bHLH101*, a basic helix-loop-helix transcription factor, was sharply upregulated on the phenanthrene, O_3_, and genotoxicity microarrays, but little affected by the bacterial infections. *AtOPT3*, an oligopeptide transporter was similarly regulated.

Among the hormone treatment microarrays, the SA dataset had the strongest correlation with the phenanthrene data (Spearman correlation *ρ *= 0.55, Table 1). In addition to *PR-1 *and other pathogen resistance (PR) genes, the phenanthrene microarray identified additional transcripts that indicate SA involvement. First, *ICS1*, an isochorismate synthase involved in SA biosynthesis, is normally induced by pathogen infection [29] and was upregulated on phenanthrene (Additional File 1). Second, the transcript of *EDS5 *(*SID1*), a MATE transporter necessary for SA signaling, was also upregulated by the PAH (Additional File 1), and is also induced in the O_3_, ultraviolet, and some biotic stress datasets. Finally, several SA early-response transcripts were induced on the phenanthrene and SA microarrays, including the UDP-glycosyl transferase *UGT1 *and *GST25*.

Low correlations with the phenanthrene treatment were found for treatments with abscisic acid, the auxin transport inhibitor triiodobenzoic acid (TIBA), brassinolide, cytokinin, the auxin indoleacetic acid, the gibberellic acid biosynthesis inhibitor paclobutrazol (PAC), the ethylene precursor ACC, and the inhibitor of ethylene biosynthesis, aminoethoxyvinylglycine (AVG) (Table 1). However, treatment with AgNO_3_, which inhibits ethylene signaling [30], correlated noticeably with phenanthrene (*ρ *= 0.48). The inconsistency of the two ethylene inhibitors could be due to non-ethylene side-effects of AgNO_3 _or AVG. Analyzing the full set of ~23 × 10^3^ probes on the microarray, these two treatments produced a paradoxically low Spearman correlation coefficient of *ρ *= 0.21, thereby supporting a side-effect hypothesis. Furthermore, the correlation between the AgNO_3 _and O_3 _microarray datasets was *ρ *= 0.60, hinting that silver nitrate induced oxidative stress. Taken together, these data indicate that the similarities between phenanthrene and AgNO_3 _induced stress responses are not related to perturbed ethylene signaling.

### Analyses of transgenic ethylene-responsive GUS-reporter plants

Ethylene is commonly known as a stress hormone. The microarray results clearly indicated involvement of ethylene-regulated genes in the phenanthrene response, but the downregulated ethylene biosynthesis transcripts *ACO2 *and *ACS6 *suggested that the PAH reduced ethylene production. At the same time, comparisons of the phenanthrene data with ethylene inhibition and precursor spike-in datasets (AVG, AgNO_3_, and ACC in Table 1 and Figure 2) suggested that ethylene involvement was more nuanced than a global up- or down-regulation of ethylene signaling. To better understand this relationship, we analysed the role of ethylene under phenanthrene treatment more closely.

**Figure 3:**
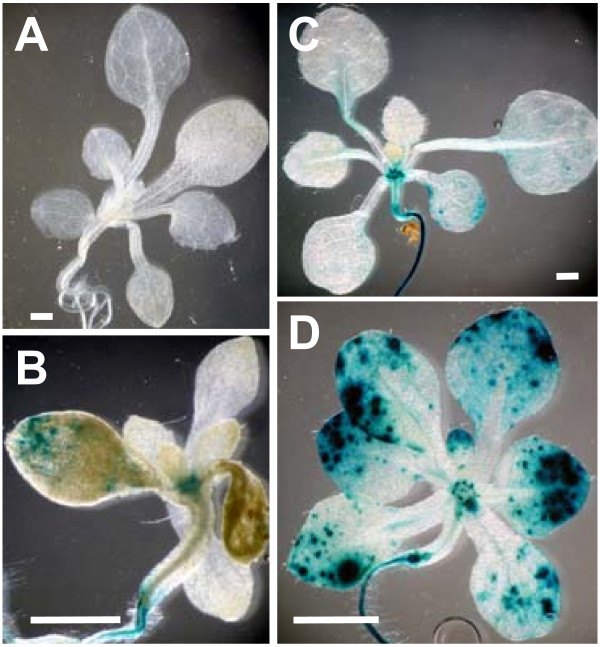
**Ethylene reporter gene expression in plants treated with phenanthrene and grown in long day light**. Histochemical staining of GUS activity in *CH5B::GUS *(A, B), *AtGSTF2::GUS *(C, D) transgenic Arabidopsis plants in absence (A, C) or presence (B, D) of phenanthrene. Plants were grown in long days for 14 d. Seedlings were stained for 15 h for GUS activity in staining buffer containing 2 mM 5-bromo-4-chloro-3-indolyl-b-D-glucuronide. Scale bars 1 mm.

**Figure 4:**
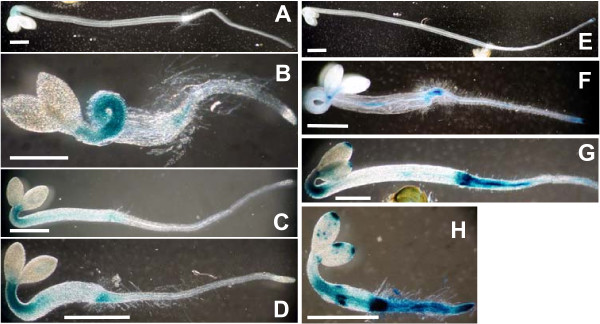
**Ethylene reporter gene expression in plants treated with phenanthrene and ACC, and grown in the dark**. Histochemical staining of GUS activity in *CH5B::GUS *(A-D) and *AtGSTF2::GUS *(E-H) transgenic Arabidopsis plants grown for 4 d in the dark. A and E, 0 mM phenanthrene, 0 *μ*M ACC; B and F, 0 mM phenanthrene, 20 *μ*M ACC; C and G, 0.25 mM phenanthrene and 0 *μ*M ACC; D and H, 0.25 mM phenanthrene, 20 *μ*M ACC. Seedlings were stained for 15 h for GUS activity in staining buffer containing 2 mM 5-bromo-4-chloro-3-indolyl-b-D-glucuronide. Scale bars 1 mm.

First, to observe localized effects of phenanthrene on ethylene signaling targets, we used the transgenic reporter plants *CH5B::GUS *and *AtGSTF2::GUS*, which indicate *GUS *expression driven by ethylene-inducible promoters from the bean basic chitinase [31] and *AtGSTF2 *genes [19], respectively. Activation of transcription from the *CH5B *promoter in Arabidopsis leaves requires ethylene signaling through the ethylene receptor *ETR1 *[31]. In contrast, while being responsive to ethylene, the *AtGSTF *promoter can also be activated through an *ETR1*-independent mechanism after treatment with glutathione, paraquat, copper, and naphthalene acetic acid (NAA) [19]. Figure 3 shows reporter gene expression in both lines when grown in long days in the presence of phenanthrene. In both lines, the reporter expression occurred in scattered patches on the leaf blades. These spatial patterns are similar to the patterns of necrotic lesions induced by phenanthrene [14]. To dissect the contributions of phenanthrene and ethylene in activating these promoters, the two reporter lines were grown in the dark for 4 d while treated with combinations of phenanthrene and ACC. Figure 4 shows that in both lines, compared to the untreated controls (Figure 4A and 4E), PAH treatment upregulated *GUS *expression (Figure 4C and 4G). The treatments with ACC alone (Figure 4B and 4F) or in combination with phenanthrene (Figure 4D and 4H) produced similar *GUS *expression patterns.

Although the histological GUS-assay is not quantitative, the relative intensity of staining can provide meaningful information. When treated with phenanthrene, *GUS *expression was generally stronger in *AtGSTF2::GUS *plants than in the *CH5B::GUS *plants (Figure 3 and Figure 4). The strongest GUS activity in *CH5B::GUS *was due to ACC treatment (Figure 4B). In contrast, in *AtGSTF2::GUS *GUS activity was strongest in plants exposed to both ACC and phenanthrene (Figure 4H).

### Responses of ethylene mutants to phenanthrene treatment

To further determine whether PAH stress involves ethylene signaling, we compared the phenotypes of several ethylene mutants to WT Arabidopsis grown on phenanthrene-containing media. The classic triple response of dark-grown seedlings grown in the presence of ethylene produces an exaggerated apical hook, a thickened, short hypocotyl, and a short root. Utilizing this behavior, ethylene signaling mutants and WT were grown in the dark to examine how the mutations affected phenanthrene-induced growth responses in seedlings. The mutants included the ethylene overproducer *eto3 *[32]; the ethylene-insensitive gain-of-function receptor mutant *etr1-4 *[33]; the mutant *etr1-7 *[34,35] which exhibits slightly enhanced ethylene sensitivity, and the *etr1-6;etr2-3;ein4-4 *[34] triple ethylene receptor mutant which exhibits constitutive ethylene signaling. Because the WT and ethylene signaling mutants differ in root and shoot lengths even under control conditions, absolute length comparisons under phenanthrene treatment are not meaningful between genotypes. To facilitate comparison, the relative length change within each genotype was defined as the ratio (%) of phenanthrene-treated length to non-treated length. These response ratios were then compared between the genotypes.

**Figure 5:**
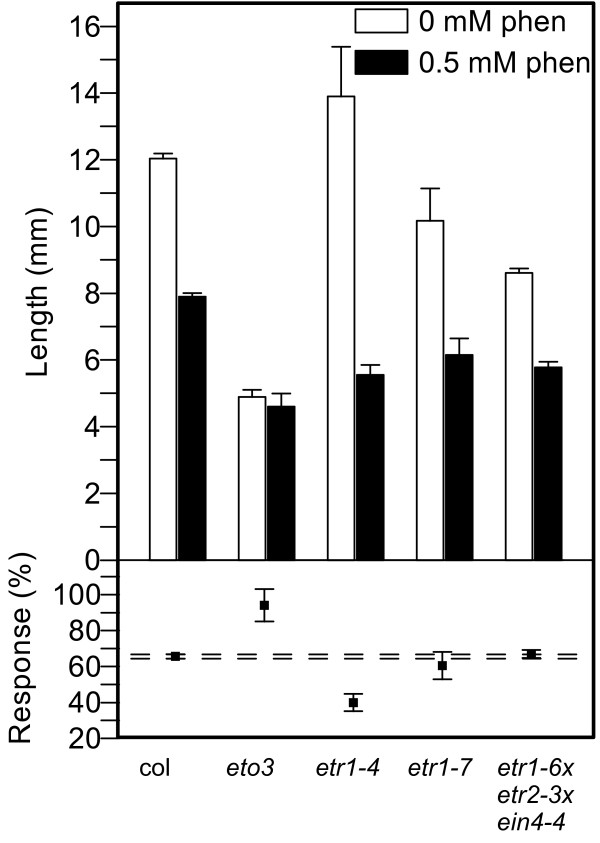
**Hypocotyl lengths of ethylene mutants treated with phenanthrene**. Hypocotyl length of 4 d old Arabidopsis seedlings grown in absence or presence of phenanthrene in the dark. Top: Mean root lengths with standard error bars. Bottom: Response (%) is the ratio of root length on phenanthrene to root length without phenanthrene treatment. Bars represent mean ± error (see Methods section for calculation). Horizontal, dashed lines mark the error range for Columbia WT. At least ten seedlings were measured for each treatment and genotype.

Figure 5 shows phenanthrene-induced hypocotyl growth responses in dark-grown seedlings. Phenanthrene treatment reduced hypocotyl elongation in all plants except in *eto3*, in which hypocotyl length was unaffected. As a baseline, the hypocotyls of WT were 12.0 ± 0.2 mm long on control medium, and 7.9 ± 0.1 mm long on 0.5 mM phenanthrene, giving a response ratio of 66 ± 1.3%. In the ethylene-overproduction mutant *eto3*, the length-reducing effect of phenanthrene was mitigated, producing hypocotyls as long as in the untreated control. Conversely, *etr1-4*, the ethylene-insensitive mutant grew to only 40 ± 4.8% of the length of the untreated mutant. The genotypes *etr1-7 *(response 60 ± 7.6%) and *etr1-6;etr2-3;ein4-4 *(response 67 ± 2.2%) did not differ significantly from WT in their hypocotyl responses to phenanthrene.

**Figure 6:**
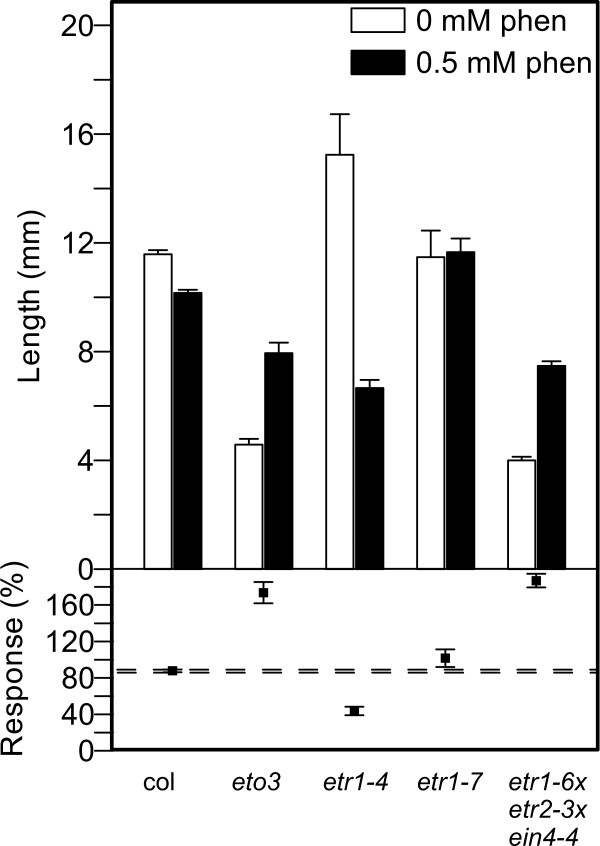
**Root lengths of ethylene mutants treated with phenanthrene**. Root length of 4-day old Arabidopsis seedlings grown in absence or presence of phenanthrene in the dark. For further explanations, see Figure 5 legend.

Root growth of phenanthrene-treated mutants differed markedly from the hypocotyl responses (Figure 6). Contrasting with the hypocotyl, root elongation of dark-grown WT was only marginally affected by phenanthrene, and the *etr1-7 *mutant was unaffected. Surprisingly, *eto3 *(response 174 ± 12%) and the triple mutant *etr1-6;etr2-3;ein4-4 *(response 187 ± 7.4%) grew even longer roots on phenanthrene than on control medium. In contrast, *etr1-4 *roots were significantly shorter on phenanthrene than on control medium (response 44 ± 4.7%).

## Discussion

Broadly, the response of Arabidopsis to phenanthrene is a complex perturbation of multiple systems, with a dominant theme of oxidative stress and similarities to pathogenic responses.

### Phenanthrene induces oxidative stress and a metabolic shift from anabolism to catabolism

Consistent with physiological studies that associated PAH treatment with oxidative stress [10-12,14], transcripts related to oxidative stress were overrepresented among the phenanthrene responsive genes (Additional File 3 and Additional File 4e). In addition, polyamine levels and ADC enzyme activity were reported to increase in the aquatic plant *Riccia fluitans *when treated with phenanthrene [12], which is consistent with the present data that indicate an upregulation of *ADC2 *mRNA.

At the systemic level, the microarray results bear strong resemblance to the transcriptional responses induced by fungal, bacterial pathogen, ozone or osmotic shock treatments (Figure 1, Figure 2, Table 1, and Additional File 4e). As the phenanthrene-treated plants were grown in sterile conditions, it is unlikely that the similarities to pathogen treatments were caused by confounding microbial effects. More likely, the unifying theme of these treatments is the production of ROS [15,36-40]. Following the initial oxidative burst, PAH-treated plants activate mechanisms similar to a pathogen defense including HR-like cell death [14] and induction of a battery of defense genes.

Similar responses have been described in ozone-treated plants, which also generate ROS and erroneously activate pathogen defense programs [15]. However, while oxidative stress was occurring under phenanthrene treatment, several antioxidant genes were downregulated. This scenario can occur when plants invoke a positive feedback loop that amplifies ROS to serve as signaling molecules [28,41]. An early perturbation of the redox network is clear as downregulation of catalase mRNA [10], as well as increased H_2_O_2 _levels and cell death [14], were detected within 12 h of PAH treatment. Along similar lines, prior work in Arabidopsis found *CAT2 *downregulated within three hours of O_3 _treatment [42]. Supporting the notion of ROS positive feedback activation, the respiratory burst oxidase *AtRbohD *was upregulated in phenanthrene-treated plants (Additional File 5), and was similarly upregulated by O_3 _and pathogenic attack conditions. Similarly, the tobacco ortholog *NtrbohD *was induced during an oxidative burst under O_3 _treatment [15]. The widespread destruction of chloroplast and mitochondrial membranes [10] may have injected additional ROS into the system.

PAH treatment caused downregulation of genes involved in photosynthesis and protein biosynthesis (Additional File 3 and Additional File 4a), which agrees with previous studies reporting overall diminished plant size and reduced chlorophyll levels [10,14]. Up-regulation of glycolysis and the citric acid cycle (Additional File 3 and Additional File 4a), as well as the similarity to the senescence microarray data (Table 1, Figure 2), further reveal a major metabolic shift from anabolism to catabolism. In addition, growth inhibition and the breakdown of the photosynthetic machinery are commonly observed ethylene effects [16].

### Phenanthrene interferes with hormone signaling networks

Results presented here suggest that the complex physiological PAH stress symptoms likely involve multiple hormone pathways, including SA, ethylene, JA, and abscisic acid (ABA). Furthermore, the GUS expression patterns in phenanthrene-treated *CH5B::GUS *and *AtGSTF2::GUS *lines suggest that ethylene and SA levels are locally elevated in PAH-stressed plant tissues (Figure 3 and Figure 4). The spatial patterns in leaves resemble previous observations of phenanthrene-induced, localized cell death and H_2_O_2_accumulation [14], supporting the hypothesis that, in addition to SA, ethylene is involved in the development of the PAH symptoms. Interestingly, reporter activity was consistently more pronounced in PAH-treated *AtGSTF2::GUS *than in the *CH5B::GUS *transgenic plants (Figure 3 and Figure 4). This difference in *GUS *expression may be caused by a differential ethylene sensitivity of the two promoters. This explanation is plausible as the *CH5B *promoter in transgenic Arabidopsis leaves is approximately an order of magnitude less sensitive to ethylene than the endogenous basic chitinase promoter [31]. A further explanation for the differential reporter levels is that the two transcriptional programs involve other signals in addition to ethylene [19,31]. Indeed, SA signaling is necessary for strong *AtGSTF2 *induction by ethylene [43].

Analyses of quantitative growth responses of dark-grown ethylene mutants exposed to phenanthrene revealed further interesting interactions between phenanthrene and ethylene signaling. Without PAH, the ethylene overproducer *eto3 *and the constitutively-signaling triple mutant grew short hypocotyls and roots, consistent with the standard model of ethylene-induced growth reduction. However, when treated with phenanthrene, these two lines grew longer roots than on control medium, suggesting that the treatment inhibits ethylene signal transduction. This hypothesis is supported by the observations that the exaggerated apical hook, which is typical in ACC-treated dark-grown plants (Figure 4B and 4F), was absent in PAH-treated plants (Figure 4D and 4H). This phenotype was frequently observed in WT plants treated with both ACC and phenanthrene (not shown). The observation that typical triple-response symptoms were attenuated under phenanthrene treatment suggests that the PAH negatively interferes with the ethylene signal transduction pathway or with ethylene biosynthesis in conditions of elevated ethylene levels or signaling. It has been proposed that ethylene can exhibit inhibiting or stimulating effect on growth, depending on the ethylene concentration [16]. Furthermore, the ethylene-insensitive *etr1-4 *mutant responded to the PAH with significantly stronger growth inhibition than the WT. This result clearly shows that the phenanthrene-induced growth reduction does not require ethylene signaling through the ETR1 receptor. Taken together, the mutant experiments suggest that ethylene is not required for the development of some of the PAH stress symptoms, and, phenanthrene inhibits some ethylene responses under conditions of elevated ethylene levels.

### Integrated model of PAH response in Arabidopsis

With these and previous results taken in total, we propose a model of the PAH response in plants. Shortly following uptake, the PAH molecules may be oxidized by mono- or dioxygenases into reactive compounds. An analogous biochemical process occurs in animals, catalyzed by cytochrome P450s [44,45], producing toxic and mutagenic electrophiles. ROS deriving from PAH oxidation would increase the overall ROS level, and thereby contribute towards activation of ROS-dependent signaling pathways. Alternatively, the PAH molecule may be directly recognized by a receptor such as a PAS-domain protein, a large and widely-distributed class of environmental sensors that includes the vertebrate aryl hydrocarbon receptor [46,47]. The strong similarities to biotic stress also suggest that the PAH could be cross-reacting with a pathogen recognition system. Regardless of the initial mechanism of action, the hormones SA, ethylene, and JA appear to be involved in the response, and other unidentified signals also are likely relevant. Finally, the oxidized intermediates can be conjugated with a sugar or glutathione, and sequestered into the vacuole or cell wall. The initial PAH or its downstream products have been shown to accumulate in trichomes and other epidermal cells [14], although the recognition and transport mechanisms remain unknown.

Additional studies will help elucidate causality in the complex PAH stress response. It would be instructive to perform high-resolution time-series experiments to measure transcripts implicated in the earliest modes of action, as well as direct measurement of hormone levels. In addition, it would be valuable to perform tissue-specific molecular and enzyme assays, particularly of the zones implicated by the positive GUS results and necrotic areas. Furthermore, in addition to the ethylene mutants, the PAH response in other signaling mutants should be analysed.

Even though many remaining questions surround PAH stress, the microarray data provide a number of leads for improving PAH phytoremediation. Relaxing the rate- and capacity-limiting bottlenecks in the PAH detoxification pathway would reduce the cytoplasmic concentration of PAHs, thereby decreasing the effective toxicity to the plant and allowing increased uptake of the pollutant. For example, further increasing GST and UGT protein levels, or artificially up-regulating vacuolar transporters of conjugated xenobiotics, may produce plants with improved phytoremediation capabilities. The present results, as well as the suggested follow-on research, will be of great value in breeding and engineering plants for phytoremediation of polycyclic aromatic hydrocarbons.

## Conclusions

The microarray experiments and comparative analyses show that phenanthrene treatment of Arabidopsis induces oxidative stress networks, closely resembling pathogen defense programs. A battery of altered transcripts revealed perturbations of the ROS, HR, and SAR systems. The present data support the hypothesis that the hormones SA, ethylene, and JA are involved in PAH response. In total, the results provide a large number of new pathway targets for researching and engineering plants for PAH phytoremediation.

## Methods

### Plants and Growth Conditions

Seeds of the Arabidopsis ecotype Colombia were obtained from Arabidopsis Research Centre and used as the WT control in all experiments. Seeds of the mutants *eto3*, *etr1-7*, *etr1-4*, and *etr1-6;etr2-3;ein4-4 *were a gift from Eric Schaller or were obtained from ABRC. Seeds of *AtGSTF2::GUS *fusion plants [19] were a gift from Peter Goldsbrough. Seeds of bean basic chitinase *CH5B::GUS *fusion plants [31] were a gift from Sara Patterson.

Seeds were surface-sterilized, stratified, and placed in Petri dishes containing half-strength Murashige and Skoog medium, supplemented with sucrose and 0, 0.25 or 0.5 mM of phenanthrene, as described previously [14]. ACC was added to the growth medium in appropriate amounts before autoclaving. Plants were grown at 23 ± 1°C either in the dark or under long-day conditions (16/8 h photoperiod at approximately 130 *μ*mol photons *m*^-2^*s*^-1^) for 4-21 d as indicated in the text. Before plates were put in darkness, they were exposed to white light for 10-12 h to achieve uniform germination. When root or hypocotyl lengths were to be measured, plates were kept in vertical orientation. Each plate contained seeds of the WT Columbia and at least of one mutant. Plants were observed under a Zeiss 2000-C dissection microscope equipped with an Olympus 340 digital camera.

All experiments were conducted at least twice with each mutant, with at least ten plants of each genotype per treatment.

### DNA Microarray Analysis

PAH treated (0.25 mM phenanthrene) and control plants (0 mM phenanthrene) were grown under long days and harvested at 21 d, and at least 20 plants were pooled and stored at -80°C. 500 mg tissue was removed from each pool and RNA was isolated using TRIzol (Molecular Research Center) per the manufacturer's instructions. Resulting samples were treated with DNase I (Invitrogen) and purified with RNeasy Mini Cleanup (Qiagen) per the manufacturers' instructions. Labeling was performed with the Affymetrix Enzo kit and processed on a Affymetrix Fluidics Station Model 450. Hybridized chips were read on a model M10 scanner.

Two rounds of biological replication were analyzed. In the first replicate, treated and control samples were each run on one Affymetrix ATH1-121501 microarray. In the second biological replicate, the treated sample was applied to one microarray, and the control sample was applied to two microarrays as a technical replicate. See Additional File 7, Additional File 8, and Additional File 9 for further technical details on the microarray experiment.

Validating the microarray data, previous RT-PCR analysis of *actin-7*, *eif4a*, *PR-1*, *PDF1.2b*, and *AtEXP8 *[14] (and unpublished data) are consistent with the present results. In addition, using RT-qPCR with four replicates per reaction and *actin-7 *as a reference, we validated that the differential responses for *GSTF6 *and *PR-1 *are consistent with the microarray dataset (data not shown).

### Bioinformatic Analyses

Data analysis was performed in R version 2.9.2 [48] and Bioconductor version 2.4.1 [49] installed on x86 hardware running Debian Linux Version 5.0. All of the procedures below were scripted in R and Python software written for this project.

To determine differential expression of the phenanthrene microarray dataset, the Affymetrix .CEL files were normalized by the Bioconductor just.gcrma algorithm using default parameters [50]. To reduce the false discovery rate, nonspecific prefiltering was performed using the Bioconductor genefilter package, eliminating probes with raw signal intensity less than 100 on all microarrays, and eliminating probes with an interquartile intensity ratio of less than 1.41 across the microarrays. The prefiltered set was then tested for statistical significance by a linear model using Limma [51], corrected for multiple comparisons with a Benjamini and Hochberg false discovery rate limit of 0.05. To identify genes with putative biological significance, probes with differential expression ratios greater than 2-fold up or 2-fold down were preserved, and these remaining probes were defined as the set of 1031 differentially-expressed, phenanthrene responsive genes used in subsequent analysis. The Affymetrix probe identifiers were mapped to Arabidopsis Genome Identifiers (AGIs), symbols, and annotations using the ath1121501.db metadata in Bioconductor.

To compare the phenanthrene microarray data with published microarray data, Affymetrix ATH1 .CEL files were obtained from the AffyWatch service of the Nottingham Arabidopsis Stock Centre http://affymetrix.arabidopsis.info. The published .CEL files and our phenanthrene .CEL files were normalized together using just.gcrma as described above. To perform the hierarchical clustering shown by the heatmap, Kendall tau correlation matrices between genes and experiments were computed, and complete linkage clustering was computed by the R hclust function. The resulting clustering was visualized by the R heatmap.2 algorithm.

Gene ontology analysis for overrepresented biological process (BP) terms was performed with the GOstats package of Bioconductor [52]. The set of 1031 differentially-expressed probes was partitioned into up-regulated and down-regulated subsets, and their Affymetrix probe identifiers were mapped to Arabidopsis Genome Identifiers (AGI). These AGI sets were tested against the universe of probed AGIs using the hyperGTest function, using a p-value cutoff of 0.05 and with the conditional scoring algorithm enabled.

MapMan [53] maps were produced to visualize cellular processes affected by the phenanthrene treatment. log_2_-transformed mean differences between transcript signals in phenanthrene-treated and control microarrays served as input to MapMan.

### Root and Shoot Measurements

Root and shoot lengths were measured on digital photographs using NIH ImageJ v 1.3.1_13 software [54]. In each experiment phenanthrene response percentage (R) of a genotype was calculated as *R *= 100 × (*AV E*_*p*_/*AV E*_*c*_), where *AV E*_*p *_is the mean organ length in phenanthrene treatment and *AV E*_*c *_is the mean organ length in control (i.e., in absence of phenanthrene). Error of R (RE) was calculated as , where *δ*_*c *_and *δ*_*p *_are standard deviations of organ length in control and phenanthrene treatment, respectively; *n*_*c *_and *n*_*p *_are numbers of roots or hypocotyls measured in control and phenanthrene treatment, respectively. In the root and hypocotyl length assay mutant's response to phenanthrene *R*_*m *_was considered significantly different from the WT response (R [wt]), if the intervals [*R*_*wt *_- *RE*_*wt*_, *R*_*wt *_+ *RE*_*wt*_] for the WT, and [*R*_*m *_- *RE*_*m*_, *R*_*m *_+ *RE*_*m*_] for the mutant, did not overlap.

### Histochemistry

GUS-staining was performed as described by [55]. To facilitate relative comparisons of reporter activity, identical GUS staining conditions were used in all experiments. For all histochemical methods whole plants or shoots were photographed under a Zeiss 2000-C dissecting microscope equipped with an Olympus 340 digital camera before and after staining. Images representative of at least ten plants per treatment and experiment are shown in Figure 3 and Figure 4.

## Authors' contributions

DW performed the microarray experiments and bioinformatics analyses. MA performed the mutant growth and histochemistry experiments. DW and MA contributed equally to this work. ACC conceived of the studies and contributed to the planning and oversight of the experiments. All authors contributed to the data analysis and composition of the manuscript.

## Supplementary Material

Additional file 1 **Full microarray dataset**. This .csv file contains the full, non-prefiltered microarray experiment dataset. All values were log_2 _transformed before calculations. Ratio, replicate means, and p-values were computed by the Bioconductor limma package. Click here for file

Additional file 2 **Differentially-expressed transcripts**. This .csv file contains the set of 1031 differentially-expressed transcripts, computed as described in the Methods section. Ratio values are log_2 _transformed. Click here for file

Additional file 3 **Gene Ontology Summary**. This .html file describes the results of the gene ontology analysis, performed separately on the sets of differentially-expressed upregulated and downregulated transcripts. The diffExpr column represents the log_2 _transformed values. Click here for file

Additional file 4 **MapMan illustrations of phenanthrene-responsive cellular processes and pathways**. Phenanthrene responsive cellular processes and pathways in Arabidopsis. MapMan illustrations of transcripts in plants grown on 0.25 mM phenanthrene for 21 d as compared to transcript levels in untreated control plants. Figure a: Overview of metabolism; Figure b: Overview of photosynthesis; Figure c: Overview of carotenoid biosynthesis; Figure d: Overview of protein targeting; Figure e: Overview of cellular responses; Figure f: Overview of gene regulation. Signal colors: Red downregulated, blue, upregulated transcripts in phenanthrene-treated plants. Scale values represent the differences between the mean log_2_-transformed values of the treated and untreated microarray sets. Click here for file

Additional file 5 **Phenanthrene induced changes in gene expression**. Arabidopsis seedlings were grown in absence (CTR) or presence (PHE) of 0.25 mM phenanthrene for 21 days and total RNA was extracted. Microarray analysis was carried out as described in the Methods section. Columns CTR (mean microarray signal from control plants), PHE (mean microarray signal from phenanthrene-treated plants), and Fold-change (PHE/CTR) are log_2 _transformed. Click here for file

Additional file 6 **Heatmap gene details**. This .html file details the contents of Figure 2. Prior to clustering, the full set of microarrays was batch-normalized as described in the Methods section; consequently, the phenanthrene experiment microarray values in this file differ slightly from the values elsewhere in this report. Click here for file

Additional file 7 **Microarray quality control analysis**. This file contains a quality control analysis of the raw microarray data used in this study. The analysis was produced using the Bioconductor package arrayQualityMetrics. Jun04 no phe.cel Jun04 phe.cel represent the untreated control and phenanthrene-treated samples, respectively, of the first replicate experiment. From the second replicate experiment, Aug04_no_phe_A.cel and Aug04_no_phe_C.cel represent the control, and Aug04_phe_B.cel represents the treated sample. Click here for file

Additional file 8 **Microarray volcano plot**. The volcano plot represents the dataset from the five microarray chips after gcRMA normalization and linear model processing by the Bioconductor limma package. Click here for file

Additional file 9 **Minimum information about a microarray experiment (MIAME) checklist**. The minimum information about a microarray experiment (MIAME) data is supplied in Additional File 9. Click here for file
