# Supplementary material for: Transcriptional responses to polycyclic aromatic hydrocarbon-induced stress in Arabidopsis thaliana reveal the involvement of hormone and defense signaling pathways
Source: BMC Plant Biol. 2010 Apr 7;10:59. doi: 10.1186/1471-2229-10-59 (PMC2923533; doi:10.1186/1471-2229-10-59)
Supplement: Additional file 3 — Gene Ontology Summary. This .html file describes the results of the gene ontology analysis, performed separately on the sets of differentially-expressed upregulated and downregulated transcripts. The diffExpr column represents the log2 transformed values. [file 1471-2229-10-59-S3.HTML]

GO analysis on genes upregulated on phenanthrene Gene to GO BP Conditional test for over-representation

| GOBPID | Pvalue | OddsRatio | ExpCount | Count | Size | Term |
| GO:0009628 | 0.000 | 3.738 | 19 | 58 | 1021 | response to abiotic stimulus |
| GO:0019748 | 0.000 | 6.471 | 6 | 31 | 315 | secondary metabolic process |
| GO:0010035 | 0.000 | 5.573 | 6 | 29 | 334 | response to inorganic substance |
| GO:0046686 | 0.000 | 5.673 | 5 | 25 | 279 | response to cadmium ion |
| GO:0006575 | 0.000 | 6.412 | 4 | 21 | 208 | amino acid derivative metabolic process |
| GO:0009407 | 0.000 | 17.939 | 1 | 10 | 41 | toxin catabolic process |
| GO:0009737 | 0.000 | 5.247 | 5 | 21 | 249 | response to abscisic acid stimulus |
| GO:0006979 | 0.000 | 5.336 | 4 | 20 | 233 | response to oxidative stress |
| GO:0009636 | 0.000 | 13.045 | 1 | 10 | 53 | response to toxin |
| GO:0009651 | 0.000 | 4.538 | 5 | 21 | 287 | response to salt stress |
| GO:0050832 | 0.000 | 9.723 | 1 | 11 | 74 | defense response to fungus |
| GO:0006725 | 0.000 | 4.965 | 4 | 18 | 227 | cellular aromatic compound metabolic process |
| GO:0009611 | 0.000 | 7.720 | 2 | 12 | 100 | response to wounding |
| GO:0009813 | 0.000 | 10.606 | 1 | 9 | 56 | flavonoid biosynthetic process |
| GO:0006869 | 0.000 | 6.721 | 2 | 11 | 102 | lipid transport |
| GO:0009269 | 0.000 | 27.407 | 0 | 5 | 15 | response to desiccation |
| GO:0009056 | 0.000 | 2.819 | 10 | 27 | 580 | catabolic process |
| GO:0006099 | 0.000 | 36.438 | 0 | 4 | 10 | tricarboxylic acid cycle |
| GO:0010224 | 0.000 | 12.677 | 1 | 6 | 32 | response to UV-B |
| GO:0050896 | 0.000 | 6.530 | 2 | 9 | 127 | response to stimulus |
| GO:0045333 | 0.000 | 11.364 | 1 | 6 | 35 | cellular respiration |
| GO:0051707 | 0.000 | 4.421 | 3 | 11 | 159 | response to other organism |
| GO:0009109 | 0.000 | 21.858 | 0 | 4 | 14 | coenzyme catabolic process |
| GO:0009414 | 0.000 | 4.559 | 2 | 10 | 134 | response to water deprivation |
| GO:0002376 | 0.000 | 3.851 | 3 | 12 | 185 | immune system process |
| GO:0009753 | 0.000 | 4.287 | 3 | 10 | 139 | response to jasmonic acid stimulus |
| GO:0009631 | 0.000 | 15.609 | 0 | 4 | 18 | cold acclimation |
| GO:0045087 | 0.000 | 3.832 | 3 | 11 | 170 | innate immune response |
| GO:0009751 | 0.000 | 4.434 | 2 | 9 | 121 | response to salicylic acid stimulus |
| GO:0042435 | 0.000 | 8.830 | 1 | 5 | 36 | indole derivative biosynthetic process |
| GO:0006094 | 0.000 | 27.244 | 0 | 3 | 9 | gluconeogenesis |
| GO:0010120 | 0.000 | 27.244 | 0 | 3 | 9 | camalexin biosynthetic process |
| GO:0016998 | 0.001 | 12.852 | 0 | 4 | 21 | cell wall catabolic process |
| GO:0009719 | 0.001 | 2.290 | 10 | 22 | 575 | response to endogenous stimulus |
| GO:0046217 | 0.001 | 23.351 | 0 | 3 | 10 | indole phytoalexin metabolic process |
| GO:0052315 | 0.001 | 23.351 | 0 | 3 | 10 | phytoalexin biosynthetic process |
| GO:0006084 | 0.001 | 11.498 | 0 | 4 | 23 | acetyl-CoA metabolic process |
| GO:0042430 | 0.001 | 7.602 | 1 | 5 | 41 | indole and derivative metabolic process |
| GO:0046482 | 0.001 | 108.671 | 0 | 2 | 3 | para-aminobenzoic acid metabolic process |
| GO:0046364 | 0.001 | 10.402 | 0 | 4 | 25 | monosaccharide biosynthetic process |
| GO:0006096 | 0.001 | 7.016 | 1 | 5 | 44 | glycolysis |
| GO:0051186 | 0.001 | 3.167 | 4 | 11 | 203 | cofactor metabolic process |
| GO:0044275 | 0.001 | 4.038 | 2 | 8 | 117 | cellular carbohydrate catabolic process |
| GO:0019320 | 0.001 | 5.305 | 1 | 6 | 68 | hexose catabolic process |
| GO:0042221 | 0.001 | 2.143 | 11 | 22 | 728 | response to chemical stimulus |
| GO:0006807 | 0.002 | 2.451 | 7 | 16 | 379 | nitrogen compound metabolic process |
| GO:0009617 | 0.002 | 6.319 | 1 | 5 | 49 | response to bacterium |
| GO:0006950 | 0.002 | 2.390 | 7 | 16 | 485 | response to stress |
| GO:0009627 | 0.002 | 8.088 | 1 | 4 | 31 | systemic acquired resistance |
| GO:0009620 | 0.003 | 7.767 | 1 | 4 | 33 | response to fungus |
| GO:0009699 | 0.003 | 5.726 | 1 | 5 | 54 | phenylpropanoid biosynthetic process |
| GO:0046164 | 0.003 | 4.631 | 1 | 6 | 77 | alcohol catabolic process |
| GO:0009808 | 0.003 | 7.529 | 1 | 4 | 33 | lignin metabolic process |
| GO:0042631 | 0.003 | 36.219 | 0 | 2 | 5 | cellular response to water deprivation |
| GO:0009695 | 0.004 | 10.892 | 0 | 3 | 18 | jasmonic acid biosynthetic process |
| GO:0042538 | 0.004 | 6.615 | 1 | 4 | 37 | hyperosmotic salinity response |
| GO:0010188 | 0.005 | 27.163 | 0 | 2 | 6 | response to microbial phytotoxin |
| GO:0009416 | 0.006 | 2.192 | 7 | 15 | 394 | response to light stimulus |
| GO:0031407 | 0.006 | 9.075 | 0 | 3 | 21 | oxylipin metabolic process |
| GO:0031540 | 0.007 | 21.729 | 0 | 2 | 7 | regulation of anthocyanin biosynthetic process |
| GO:0009991 | 0.007 | 4.480 | 1 | 5 | 66 | response to extracellular stimulus |
| GO:0046283 | 0.007 | 8.597 | 0 | 3 | 22 | anthocyanin metabolic process |
| GO:0006586 | 0.008 | 8.167 | 0 | 3 | 23 | indolalkylamine metabolic process |
| GO:0010150 | 0.008 | 8.167 | 0 | 3 | 23 | leaf senescence |
| GO:0019430 | 0.009 | 18.107 | 0 | 2 | 8 | removal of superoxide radicals |
| GO:0042128 | 0.009 | 18.107 | 0 | 2 | 8 | nitrate assimilation |
| GO:0042558 | 0.009 | 18.107 | 0 | 2 | 8 | pteridine and derivative metabolic process |
| GO:0046688 | 0.009 | 18.107 | 0 | 2 | 8 | response to copper ion |
| GO:0006082 | 0.009 | 1.894 | 10 | 19 | 576 | organic acid metabolic process |
| GO:0042742 | 0.010 | 2.832 | 3 | 8 | 163 | defense response to bacterium |
| GO:0010039 | 0.011 | 15.519 | 0 | 2 | 9 | response to iron ion |
| GO:0046685 | 0.011 | 15.519 | 0 | 2 | 9 | response to arsenic |
| GO:0006752 | 0.011 | 7.100 | 0 | 3 | 26 | group transfer coenzyme metabolic process |
| GO:0042493 | 0.013 | 4.742 | 1 | 4 | 50 | response to drug |
| GO:0006952 | 0.013 | 2.268 | 5 | 11 | 296 | defense response |
| GO:0009861 | 0.013 | 13.693 | 0 | 2 | 10 | jasmonic acid and ethylene-dependent systemic resistance |
| GO:0032787 | 0.014 | 2.242 | 5 | 11 | 281 | monocarboxylic acid metabolic process |
| GO:0010149 | 0.015 | 6.280 | 1 | 3 | 29 | senescence |
| GO:0006829 | 0.016 | 12.069 | 0 | 2 | 11 | zinc ion transport |
| GO:0006649 | 0.018 | Inf | 0 | 1 | 1 | phospholipid transfer to membrane |
| GO:0009643 | 0.018 | Inf | 0 | 1 | 1 | photosynthetic acclimation |
| GO:0009715 | 0.018 | Inf | 0 | 1 | 1 | chalcone biosynthetic process |
| GO:0010111 | 0.018 | Inf | 0 | 1 | 1 | glyoxysome organization |
| GO:0010299 | 0.018 | Inf | 0 | 1 | 1 | detoxification of cobalt ion |
| GO:0010312 | 0.018 | Inf | 0 | 1 | 1 | detoxification of zinc ion |
| GO:0015690 | 0.018 | Inf | 0 | 1 | 1 | aluminum ion transport |
| GO:0019477 | 0.018 | Inf | 0 | 1 | 1 | L-lysine catabolic process |
| GO:0019595 | 0.018 | Inf | 0 | 1 | 1 | non-phosphorylated glucose catabolic process |
| GO:0030397 | 0.018 | Inf | 0 | 1 | 1 | membrane disassembly |
| GO:0032101 | 0.018 | Inf | 0 | 1 | 1 | regulation of response to external stimulus |
| GO:0032107 | 0.018 | Inf | 0 | 1 | 1 | regulation of response to nutrient levels |
| GO:0042180 | 0.018 | Inf | 0 | 1 | 1 | cellular ketone metabolic process |
| GO:0009625 | 0.019 | 10.862 | 0 | 2 | 12 | response to insect |
| GO:0006800 | 0.020 | 5.629 | 1 | 3 | 32 | oxygen and reactive oxygen species metabolic process |
| GO:0006857 | 0.022 | 3.964 | 1 | 4 | 59 | oligopeptide transport |
| GO:0001666 | 0.023 | 9.874 | 0 | 2 | 13 | response to hypoxia |
| GO:0006006 | 0.023 | 3.928 | 1 | 4 | 60 | glucose metabolic process |
| GO:0009409 | 0.028 | 2.321 | 4 | 8 | 199 | response to cold |
| GO:0044271 | 0.028 | 2.487 | 3 | 7 | 161 | nitrogen compound biosynthetic process |
| GO:0005996 | 0.031 | 2.999 | 2 | 5 | 97 | monosaccharide metabolic process |
| GO:0042401 | 0.031 | 4.663 | 1 | 3 | 38 | biogenic amine biosynthetic process |
| GO:0052544 | 0.033 | 7.756 | 0 | 2 | 16 | callose deposition in cell wall during defense response |
| GO:0007047 | 0.035 | 2.099 | 4 | 9 | 244 | cell wall organization |
| GO:0002697 | 0.036 | 54.169 | 0 | 1 | 2 | regulation of immune effector process |
| GO:0006569 | 0.036 | 54.169 | 0 | 1 | 2 | tryptophan catabolic process |
| GO:0009446 | 0.036 | 54.169 | 0 | 1 | 2 | putrescine biosynthetic process |
| GO:0009961 | 0.036 | 54.169 | 0 | 1 | 2 | response to 1-aminocyclopropane-1-carboxylic acid |
| GO:0010266 | 0.036 | 54.169 | 0 | 1 | 2 | response to vitamin B1 |
| GO:0010315 | 0.036 | 54.169 | 0 | 1 | 2 | auxin efflux |
| GO:0010421 | 0.036 | 54.169 | 0 | 1 | 2 | hydrogen peroxide-mediated programmed cell death |
| GO:0015904 | 0.036 | 54.169 | 0 | 1 | 2 | tetracycline transport |
| GO:0019496 | 0.036 | 54.169 | 0 | 1 | 2 | serine-isocitrate lyase pathway |
| GO:0030002 | 0.036 | 54.169 | 0 | 1 | 2 | cellular anion homeostasis |
| GO:0030643 | 0.036 | 54.169 | 0 | 1 | 2 | cellular phosphate ion homeostasis |
| GO:0043620 | 0.036 | 54.169 | 0 | 1 | 2 | regulation of transcription in response to stress |
| GO:0046900 | 0.036 | 54.169 | 0 | 1 | 2 | tetrahydrofolylpolyglutamate metabolic process |
| GO:0050691 | 0.036 | 54.169 | 0 | 1 | 2 | regulation of defense response to virus by host |
| GO:0051176 | 0.036 | 54.169 | 0 | 1 | 2 | positive regulation of sulfur metabolic process |
| GO:0055061 | 0.036 | 54.169 | 0 | 1 | 2 | di-, tri-valent inorganic anion homeostasis |
| GO:0009723 | 0.039 | 2.501 | 2 | 6 | 137 | response to ethylene stimulus |
| GO:0006767 | 0.040 | 3.252 | 1 | 4 | 71 | water-soluble vitamin metabolic process |
| GO:0016265 | 0.041 | 2.754 | 2 | 5 | 104 | death |
| GO:0012501 | 0.042 | 3.214 | 1 | 4 | 72 | programmed cell death |
| GO:0055085 | 0.042 | 3.204 | 1 | 4 | 72 | transmembrane transport |
| GO:0000162 | 0.046 | 6.387 | 0 | 2 | 19 | tryptophan biosynthetic process |
| GO:0009084 | 0.046 | 6.387 | 0 | 2 | 19 | glutamine family amino acid biosynthetic process |
| GO:0052386 | 0.046 | 6.387 | 0 | 2 | 19 | cell wall thickening |
| GO:0046483 | 0.048 | 2.371 | 3 | 6 | 146 | heterocycle metabolic process |
| GO:0009743 | 0.049 | 2.186 | 3 | 7 | 182 | response to carbohydrate stimulus |


GO analysis on genes downregulated on phenanthrene Gene to GO BP Conditional test for over-representation

| GOBPID | Pvalue | OddsRatio | ExpCount | Count | Size | Term |
| GO:0015979 | 0.000 | 31.360 | 1 | 22 | 48 | photosynthesis |
| GO:0055114 | 0.000 | 19.561 | 2 | 26 | 71 | oxidation reduction |
| GO:0009773 | 0.000 | 120.910 | 0 | 11 | 14 | photosynthetic electron transport in photosystem I |
| GO:0009628 | 0.000 | 2.733 | 30 | 72 | 1007 | response to abiotic stimulus |
| GO:0015995 | 0.000 | 16.470 | 1 | 11 | 33 | chlorophyll biosynthetic process |
| GO:0033014 | 0.000 | 11.301 | 2 | 13 | 51 | tetrapyrrole biosynthetic process |
| GO:0006778 | 0.000 | 8.735 | 2 | 14 | 67 | porphyrin metabolic process |
| GO:0042742 | 0.000 | 4.931 | 5 | 21 | 163 | defense response to bacterium |
| GO:0042440 | 0.000 | 6.164 | 3 | 16 | 102 | pigment metabolic process |
| GO:0009416 | 0.000 | 3.522 | 9 | 28 | 302 | response to light stimulus |
| GO:0010205 | 0.000 | 163.047 | 0 | 5 | 6 | photoinhibition |
| GO:0009409 | 0.000 | 3.956 | 7 | 23 | 217 | response to cold |
| GO:0010207 | 0.000 | 39.196 | 0 | 6 | 11 | photosystem II assembly |
| GO:0009637 | 0.000 | 16.435 | 1 | 8 | 24 | response to blue light |
| GO:0051188 | 0.000 | 4.857 | 4 | 16 | 125 | cofactor biosynthetic process |
| GO:0009987 | 0.000 | 1.520 | 242 | 296 | 8073 | cellular process |
| GO:0008152 | 0.000 | 1.681 | 102 | 144 | 3768 | metabolic process |
| GO:0018119 | 0.000 | 21.770 | 0 | 6 | 15 | peptidyl-cysteine S-nitrosylation |
| GO:0019684 | 0.000 | 21.065 | 0 | 6 | 16 | photosynthesis, light reaction |
| GO:0010206 | 0.000 | 130.194 | 0 | 4 | 5 | photosystem II repair |
| GO:0010218 | 0.000 | 10.076 | 1 | 8 | 34 | response to far red light |
| GO:0015976 | 0.000 | 17.810 | 1 | 6 | 17 | carbon utilization |
| GO:0010114 | 0.000 | 7.975 | 1 | 9 | 46 | response to red light |
| GO:0009772 | 0.000 | 65.093 | 0 | 4 | 6 | photosynthetic electron transport in photosystem II |
| GO:0045038 | 0.000 | 65.093 | 0 | 4 | 6 | protein import into chloroplast thylakoid membrane |
| GO:0006000 | 0.000 | Inf | 0 | 3 | 3 | fructose metabolic process |
| GO:0006412 | 0.000 | 2.399 | 14 | 31 | 463 | translation |
| GO:0046483 | 0.000 | 3.303 | 6 | 17 | 187 | heterocycle metabolic process |
| GO:0009071 | 0.000 | 26.033 | 0 | 4 | 9 | serine family amino acid catabolic process |
| GO:0009765 | 0.000 | 99.296 | 0 | 3 | 4 | photosynthesis, light harvesting |
| GO:0009768 | 0.000 | 97.464 | 0 | 3 | 4 | photosynthesis, light harvesting in photosystem I |
| GO:0010196 | 0.000 | 97.464 | 0 | 3 | 4 | nonphotochemical quenching |
| GO:0009733 | 0.000 | 2.843 | 7 | 19 | 240 | response to auxin stimulus |
| GO:0043085 | 0.000 | 9.324 | 1 | 6 | 27 | positive regulation of catalytic activity |
| GO:0019253 | 0.000 | 21.693 | 0 | 4 | 10 | reductive pentose-phosphate cycle |
| GO:0009744 | 0.000 | 6.930 | 1 | 7 | 40 | response to sucrose stimulus |
| GO:0009853 | 0.000 | 8.512 | 1 | 6 | 29 | photorespiration |
| GO:0051707 | 0.000 | 2.212 | 13 | 28 | 449 | response to other organism |
| GO:0009769 | 0.000 | 48.729 | 0 | 3 | 5 | photosynthesis, light harvesting in photosystem II |
| GO:0019464 | 0.000 | 48.729 | 0 | 3 | 5 | glycine decarboxylation via glycine cleavage system |
| GO:0042549 | 0.000 | 48.729 | 0 | 3 | 5 | photosystem II stabilization |
| GO:0009743 | 0.000 | 3.190 | 5 | 14 | 159 | response to carbohydrate stimulus |
| GO:0009657 | 0.000 | 7.065 | 1 | 6 | 34 | plastid organization |
| GO:0009767 | 0.000 | 33.664 | 0 | 3 | 6 | photosynthetic electron transport chain |
| GO:0006544 | 0.001 | 13.013 | 0 | 4 | 14 | glycine metabolic process |
| GO:0006979 | 0.001 | 2.593 | 7 | 17 | 233 | response to oxidative stress |
| GO:0009902 | 0.001 | 24.362 | 0 | 3 | 7 | chloroplast relocation |
| GO:0051644 | 0.001 | 24.362 | 0 | 3 | 7 | plastid localization |
| GO:0035304 | 0.001 | Inf | 0 | 2 | 2 | regulation of protein amino acid dephosphorylation |
| GO:0010027 | 0.001 | 10.843 | 0 | 4 | 16 | thylakoid membrane organization |
| GO:0005996 | 0.001 | 3.248 | 4 | 11 | 122 | monosaccharide metabolic process |
| GO:0042254 | 0.001 | 3.248 | 4 | 11 | 122 | ribosome biogenesis |
| GO:0042548 | 0.001 | 19.596 | 0 | 3 | 8 | regulation of photosynthesis, light reaction |
| GO:0009611 | 0.002 | 3.080 | 4 | 11 | 128 | response to wounding |
| GO:0065009 | 0.002 | 3.843 | 2 | 8 | 76 | regulation of molecular function |
| GO:0006461 | 0.002 | 3.116 | 3 | 10 | 115 | protein complex assembly |
| GO:0010362 | 0.003 | 64.855 | 0 | 2 | 3 | negative regulation of anion channel activity by blue light |
| GO:0032413 | 0.003 | 64.855 | 0 | 2 | 3 | negative regulation of ion transmembrane transporter activity |
| GO:0051051 | 0.003 | 64.855 | 0 | 2 | 3 | negative regulation of transport |
| GO:0044042 | 0.003 | 3.306 | 3 | 9 | 98 | glucan metabolic process |
| GO:0051656 | 0.003 | 13.919 | 0 | 3 | 10 | establishment of organelle localization |
| GO:0009644 | 0.004 | 5.477 | 1 | 5 | 35 | response to high light intensity |
| GO:0009750 | 0.004 | 12.178 | 0 | 3 | 11 | response to fructose stimulus |
| GO:0031324 | 0.004 | 3.806 | 2 | 7 | 67 | negative regulation of cellular metabolic process |
| GO:0043933 | 0.004 | 2.149 | 8 | 17 | 277 | macromolecular complex subunit organization |
| GO:0034622 | 0.005 | 2.258 | 7 | 15 | 233 | cellular macromolecular complex assembly |
| GO:0009310 | 0.006 | 4.786 | 1 | 5 | 39 | amine catabolic process |
| GO:0022607 | 0.007 | 2.100 | 8 | 16 | 266 | cellular component assembly |
| GO:0009612 | 0.008 | 8.855 | 0 | 3 | 14 | response to mechanical stimulus |
| GO:0009739 | 0.008 | 2.747 | 3 | 9 | 116 | response to gibberellin stimulus |
| GO:0010190 | 0.008 | 21.616 | 0 | 2 | 5 | cytochrome b6f complex assembly |
| GO:0010359 | 0.008 | 21.616 | 0 | 2 | 5 | regulation of anion channel activity |
| GO:0022898 | 0.008 | 21.616 | 0 | 2 | 5 | regulation of transmembrane transporter activity |
| GO:0042592 | 0.009 | 2.398 | 5 | 11 | 161 | homeostatic process |
| GO:0044264 | 0.010 | 2.671 | 4 | 9 | 119 | cellular polysaccharide metabolic process |
| GO:0006457 | 0.011 | 2.171 | 6 | 13 | 209 | protein folding |
| GO:0007623 | 0.011 | 4.067 | 1 | 5 | 45 | circadian rhythm |
| GO:0019252 | 0.011 | 7.492 | 0 | 3 | 16 | starch biosynthetic process |
| GO:0010304 | 0.012 | 16.211 | 0 | 2 | 6 | PSII associated light-harvesting complex II catabolic process |
| GO:0016123 | 0.012 | 16.211 | 0 | 2 | 6 | xanthophyll biosynthetic process |
| GO:0019220 | 0.012 | 16.211 | 0 | 2 | 6 | regulation of phosphate metabolic process |
| GO:0005982 | 0.013 | 6.990 | 1 | 3 | 17 | starch metabolic process |
| GO:0006352 | 0.013 | 4.815 | 1 | 4 | 31 | transcription initiation |
| GO:0009828 | 0.013 | 4.815 | 1 | 4 | 31 | plant-type cell wall loosening |
| GO:0009749 | 0.016 | 6.492 | 1 | 3 | 18 | response to glucose stimulus |
| GO:0009719 | 0.016 | 1.548 | 21 | 32 | 714 | response to endogenous stimulus |
| GO:0010035 | 0.016 | 1.817 | 10 | 18 | 343 | response to inorganic substance |
| GO:0019320 | 0.016 | 3.151 | 2 | 6 | 68 | hexose catabolic process |
| GO:0006006 | 0.017 | 3.100 | 2 | 6 | 69 | glucose metabolic process |
| GO:0009058 | 0.018 | 1.259 | 101 | 120 | 3348 | biosynthetic process |
| GO:0019288 | 0.022 | 10.806 | 0 | 2 | 8 | isopentenyl diphosphate biosynthetic process, mevalonate-independent pathway |
| GO:0046688 | 0.022 | 10.806 | 0 | 2 | 8 | response to copper ion |
| GO:0009411 | 0.024 | 3.252 | 2 | 5 | 55 | response to UV |
| GO:0019752 | 0.025 | 1.560 | 17 | 26 | 574 | carboxylic acid metabolic process |
| GO:0042221 | 0.028 | 1.441 | 26 | 36 | 888 | response to chemical stimulus |
| GO:0046164 | 0.028 | 2.750 | 2 | 6 | 77 | alcohol catabolic process |
| GO:0006783 | 0.028 | 9.262 | 0 | 2 | 9 | heme biosynthetic process |
| GO:0009751 | 0.029 | 2.307 | 4 | 8 | 121 | response to salicylic acid stimulus |
| GO:0019685 | 0.030 | Inf | 0 | 1 | 1 | photosynthesis, dark reaction |
| GO:0008631 | 0.030 | Inf | 0 | 1 | 1 | induction of apoptosis by oxidative stress |
| GO:0009405 | 0.030 | Inf | 0 | 1 | 1 | pathogenesis |
| GO:0009800 | 0.030 | Inf | 0 | 1 | 1 | cinnamic acid biosynthetic process |
| GO:0010028 | 0.030 | Inf | 0 | 1 | 1 | xanthophyll cycle |
| GO:0010378 | 0.030 | Inf | 0 | 1 | 1 | temperature compensation of the circadian clock |
| GO:0016480 | 0.030 | Inf | 0 | 1 | 1 | negative regulation of transcription from RNA polymerase III promoter |
| GO:0018316 | 0.030 | Inf | 0 | 1 | 1 | peptide cross-linking via L-cystine |
| GO:0032527 | 0.030 | Inf | 0 | 1 | 1 | protein exit from endoplasmic reticulum |
| GO:0043617 | 0.030 | Inf | 0 | 1 | 1 | cellular response to sucrose starvation |
| GO:0051973 | 0.030 | Inf | 0 | 1 | 1 | positive regulation of telomerase activity |
| GO:0034284 | 0.031 | 4.868 | 1 | 3 | 23 | response to monosaccharide stimulus |
| GO:0005975 | 0.033 | 1.473 | 21 | 30 | 700 | carbohydrate metabolic process |
| GO:0006869 | 0.034 | 2.399 | 3 | 7 | 102 | lipid transport |
| GO:0009145 | 0.034 | 4.636 | 1 | 3 | 24 | purine nucleoside triphosphate biosynthetic process |
| GO:0006879 | 0.035 | 8.104 | 0 | 2 | 10 | cellular iron ion homeostasis |
| GO:0006949 | 0.035 | 8.104 | 0 | 2 | 10 | syncytium formation |
| GO:0006753 | 0.037 | 2.349 | 3 | 7 | 104 | nucleoside phosphate metabolic process |
| GO:0009605 | 0.038 | 2.191 | 4 | 8 | 129 | response to external stimulus |
| GO:0048527 | 0.039 | 3.331 | 1 | 4 | 43 | lateral root development |
| GO:0009061 | 0.041 | 7.203 | 0 | 2 | 11 | anaerobic respiration |
| GO:0009646 | 0.041 | 7.203 | 0 | 2 | 11 | response to absence of light |
| GO:0010025 | 0.041 | 7.203 | 0 | 2 | 11 | wax biosynthetic process |
| GO:0046490 | 0.041 | 7.203 | 0 | 2 | 11 | isopentenyl diphosphate metabolic process |
| GO:0009658 | 0.042 | 3.265 | 1 | 4 | 44 | chloroplast organization |
| GO:0009141 | 0.042 | 4.232 | 1 | 3 | 26 | nucleoside triphosphate metabolic process |
| GO:0006096 | 0.042 | 3.248 | 1 | 4 | 44 | glycolysis |
| GO:0051094 | 0.042 | 3.248 | 1 | 4 | 44 | positive regulation of developmental process |
| GO:0046686 | 0.043 | 1.726 | 8 | 14 | 279 | response to cadmium ion |
| GO:0016116 | 0.046 | 4.056 | 1 | 3 | 27 | carotenoid metabolic process |
| GO:0045454 | 0.049 | 3.093 | 1 | 4 | 46 | cell redox homeostasis |
| GO:0009638 | 0.049 | 6.482 | 0 | 2 | 12 | phototropism |
