# Supplementary material for: Transcriptional responses to polycyclic aromatic hydrocarbon-induced stress in Arabidopsis thaliana reveal the involvement of hormone and defense signaling pathways
Source: BMC Plant Biol. 2010 Apr 7;10:59. doi: 10.1186/1471-2229-10-59 (PMC2923533; doi:10.1186/1471-2229-10-59)
Supplement: Additional file 4 — MapMan illustrations of phenanthrene-responsive cellular processes and pathways. Phenanthrene responsive cellular processes and pathways in Arabidopsis. MapMan illustrations of transcripts in plants grown on 0.25 mM phenanthrene for 21 d as compared to transcript levels in untreated control plants. Figure a: Overview of metabolism; Figure b: Overview of photosynthesis; Figure c: Overview of carotenoid biosynthesis; Figure d: Overview of protein targeting; Figure e: Overview of cellular responses; Figure f: Overview of gene regulation. Signal colors: Red downregulated, blue, upregulated transcripts in phenanthrene-treated plants. Scale values represent the differences between the mean log2-transformed values of the treated and untreated microarray sets. [file 1471-2229-10-59-S4.PDF]

Phenanthrene responsive cellular processes and pathways in Arabidopsis. MapMan illustrations of transcripts in plants grown on 0.25 mM phenanthrene for 21 d as compared to transcript levels in untreated control plants. Figure a: Overview of metabolism; Figure b: Overview of photosynthesis; Figure c: Overview of carotenoid biosynthesis; Figure d: Overview of protein targeting; Figure e: Overview of cellular responses; Figure f: Overview of gene regulation. Signal colors: Red downregulated, blue, upregulated transcripts in phenanthrene treated plants. Scale values represent the differences between the mean $\log_2$-transformed values of the treated and untreated microarray sets.

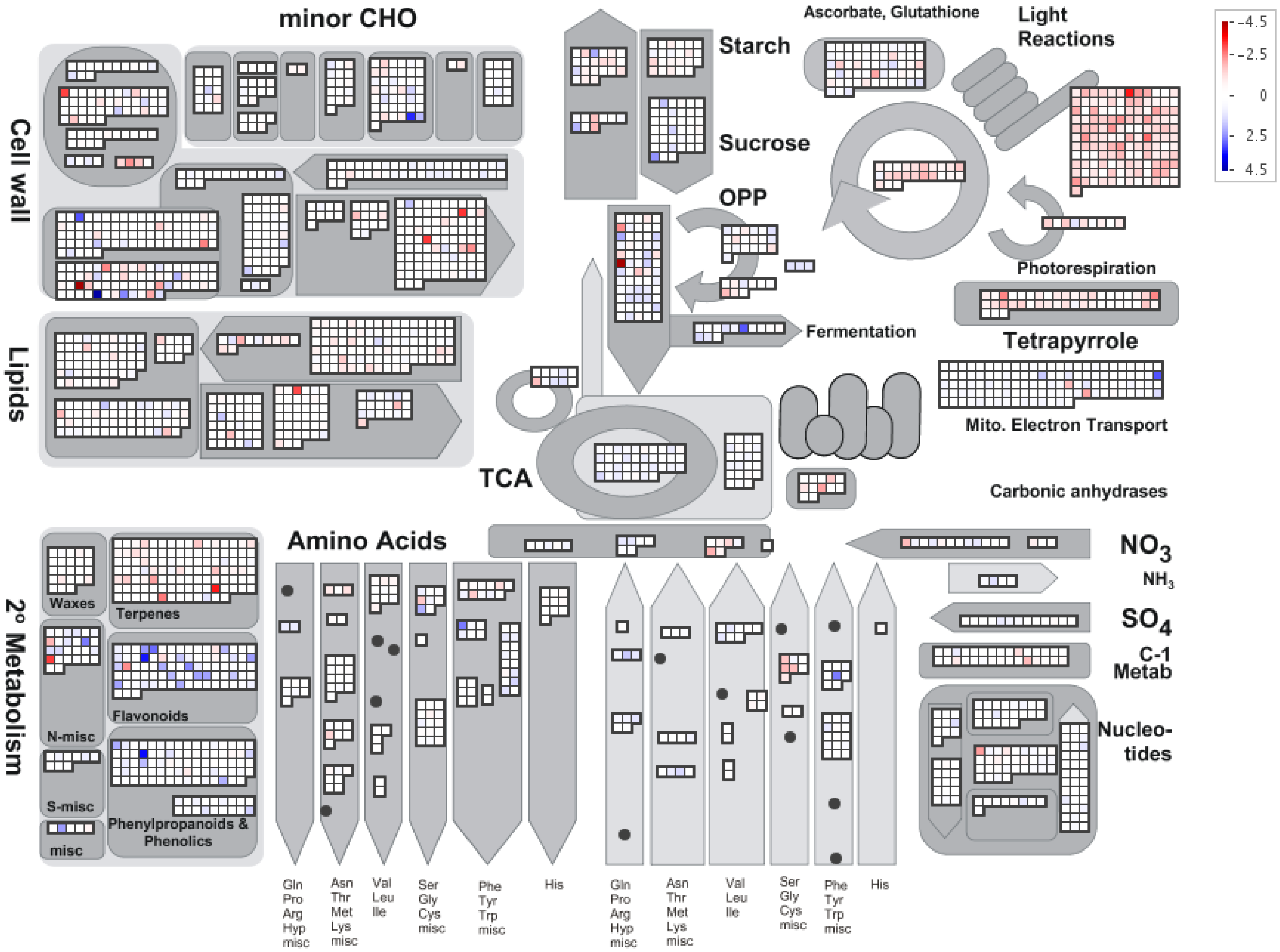

Figure a: Overview of metabolism

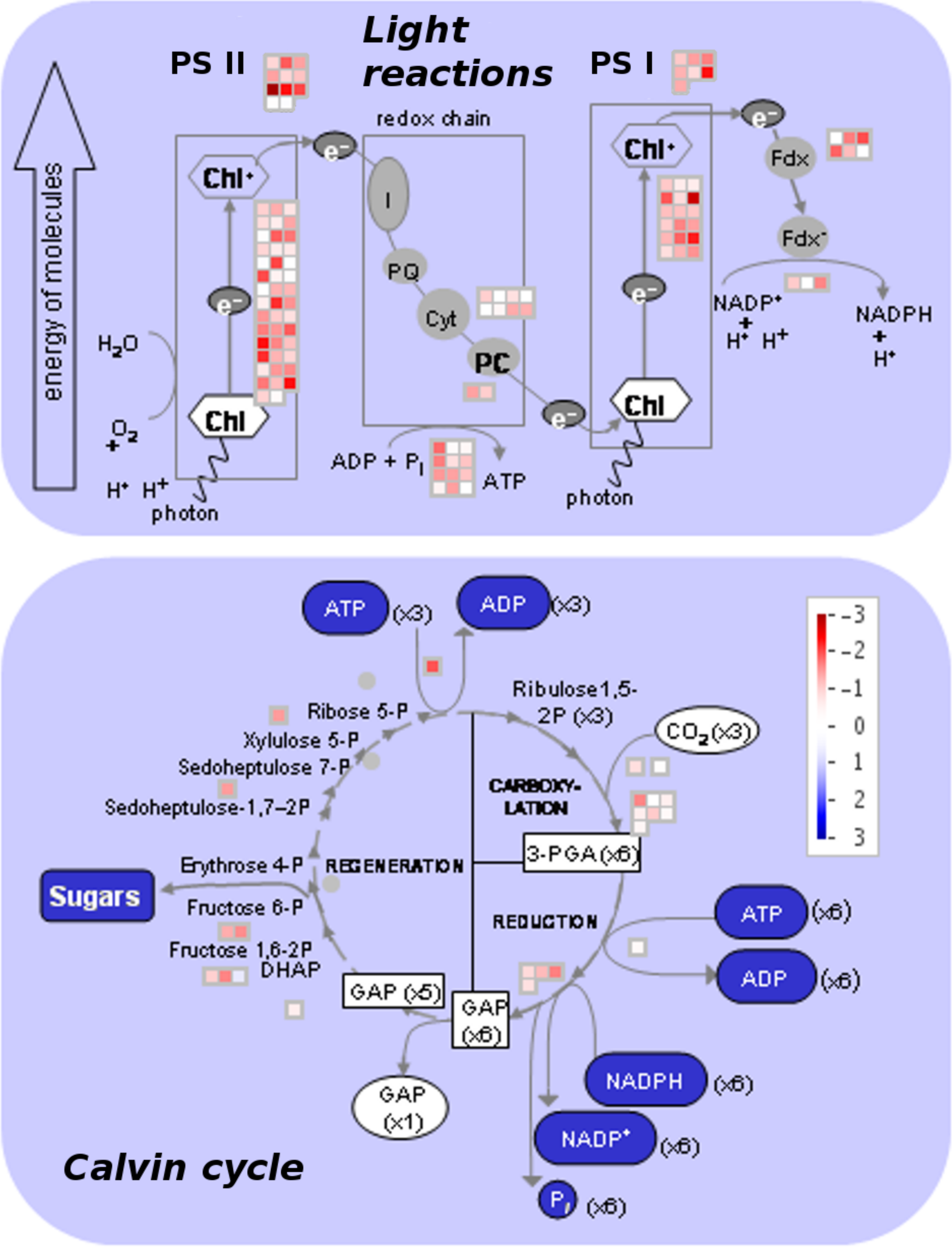

Figure b: Overview of photosynthesis

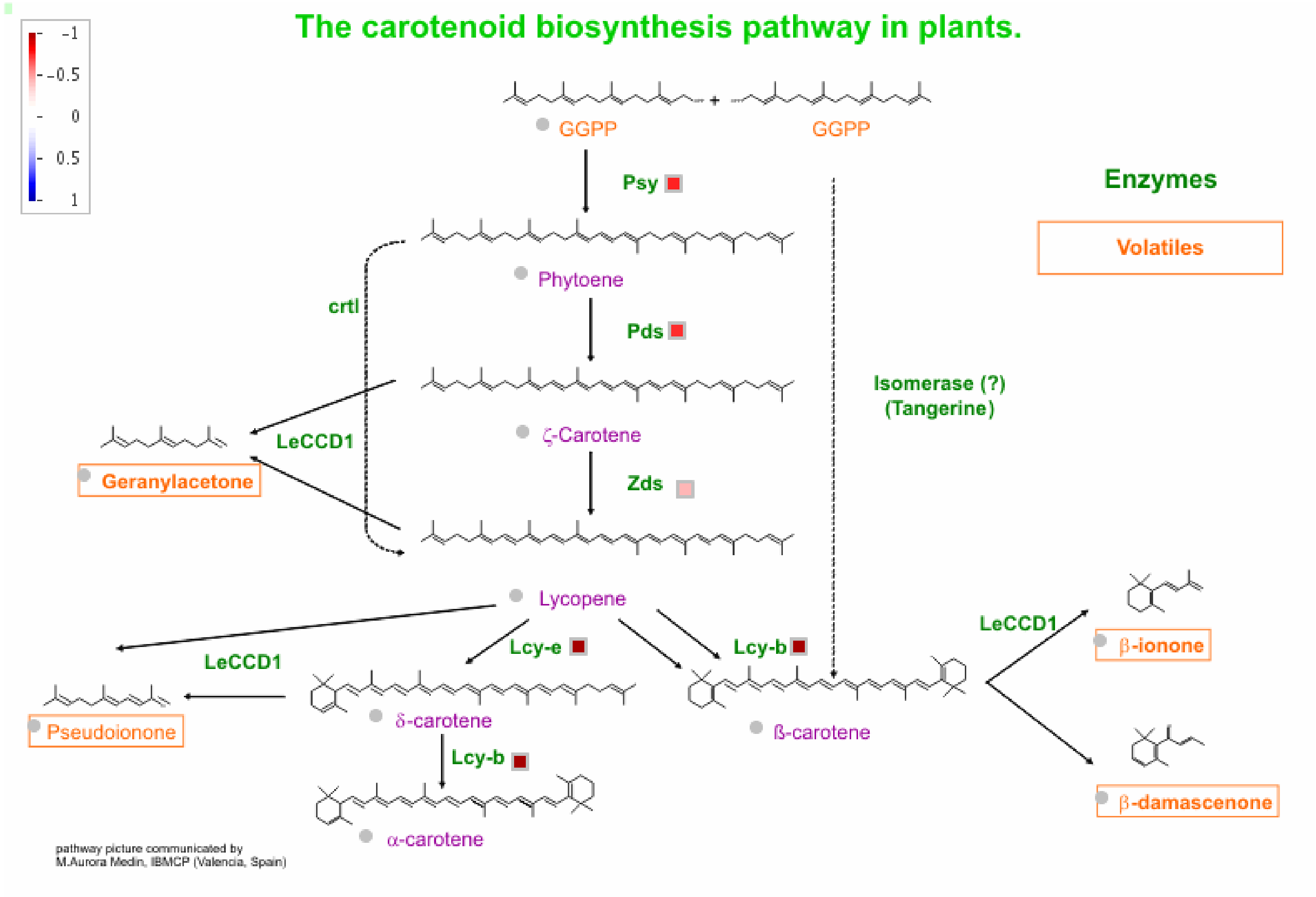

Figure c: Overview of carotenoid biosynthesis

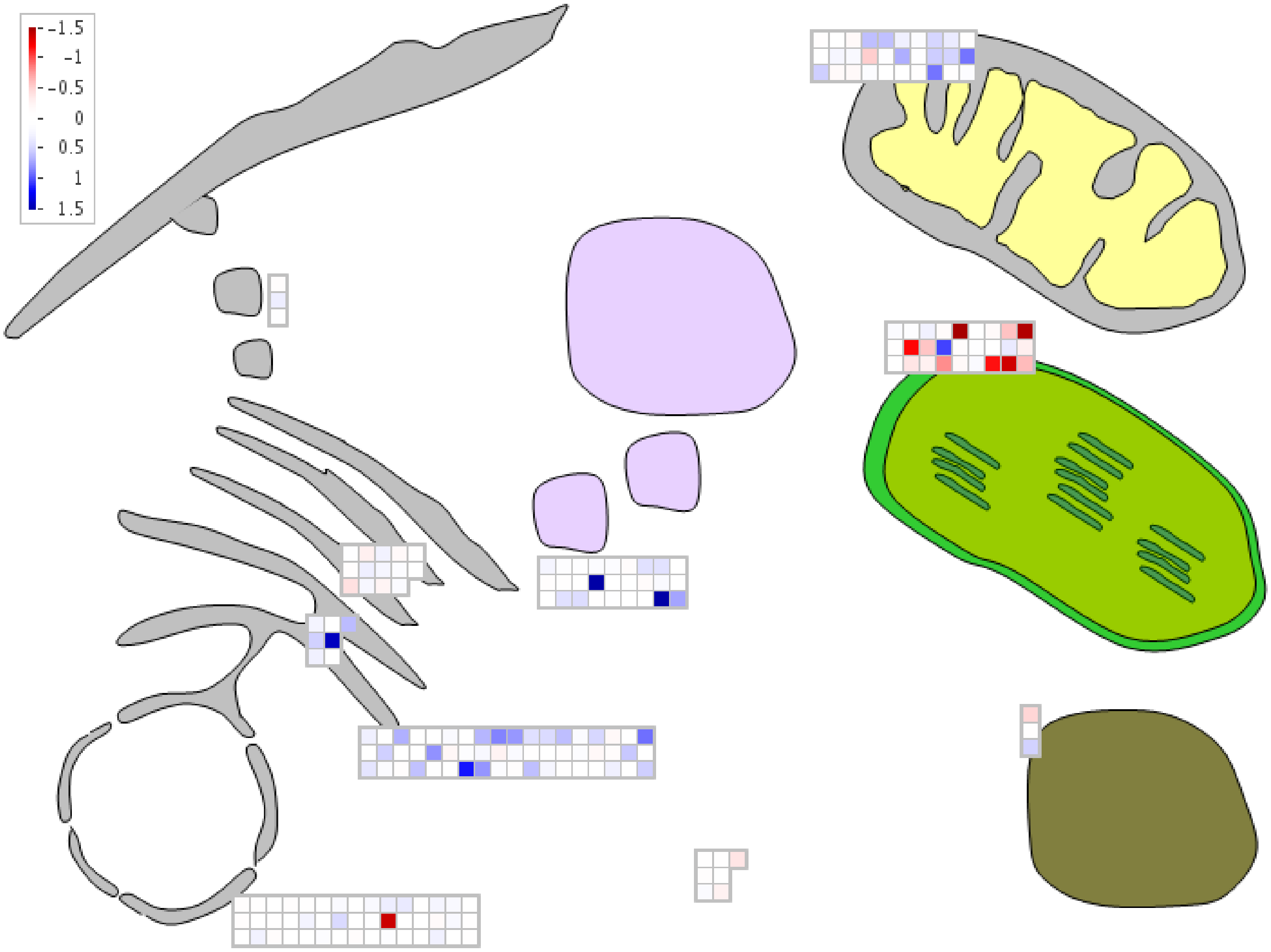

Figure d: Overview of protein targeting

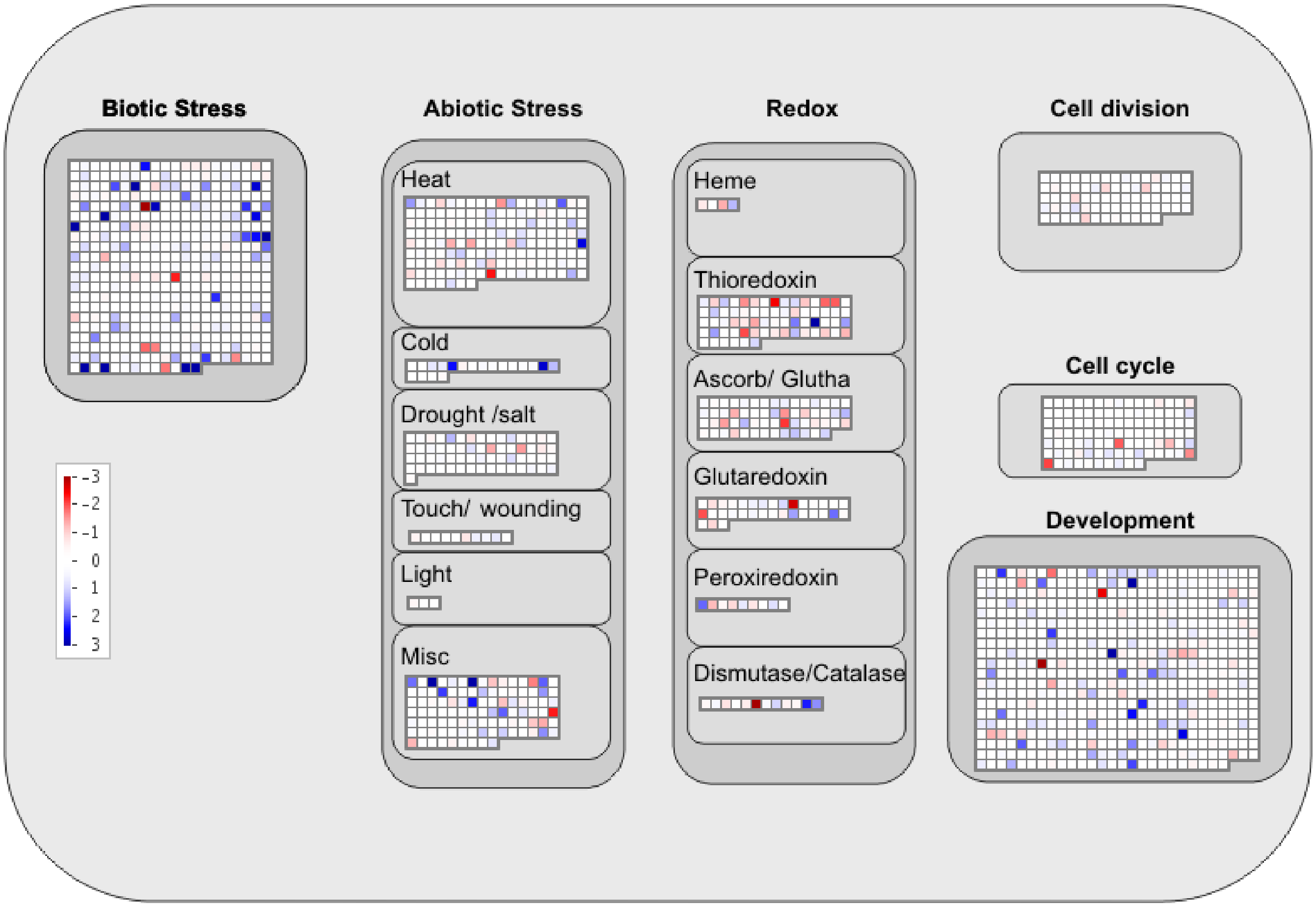

Figure e: Overview of cellular responses

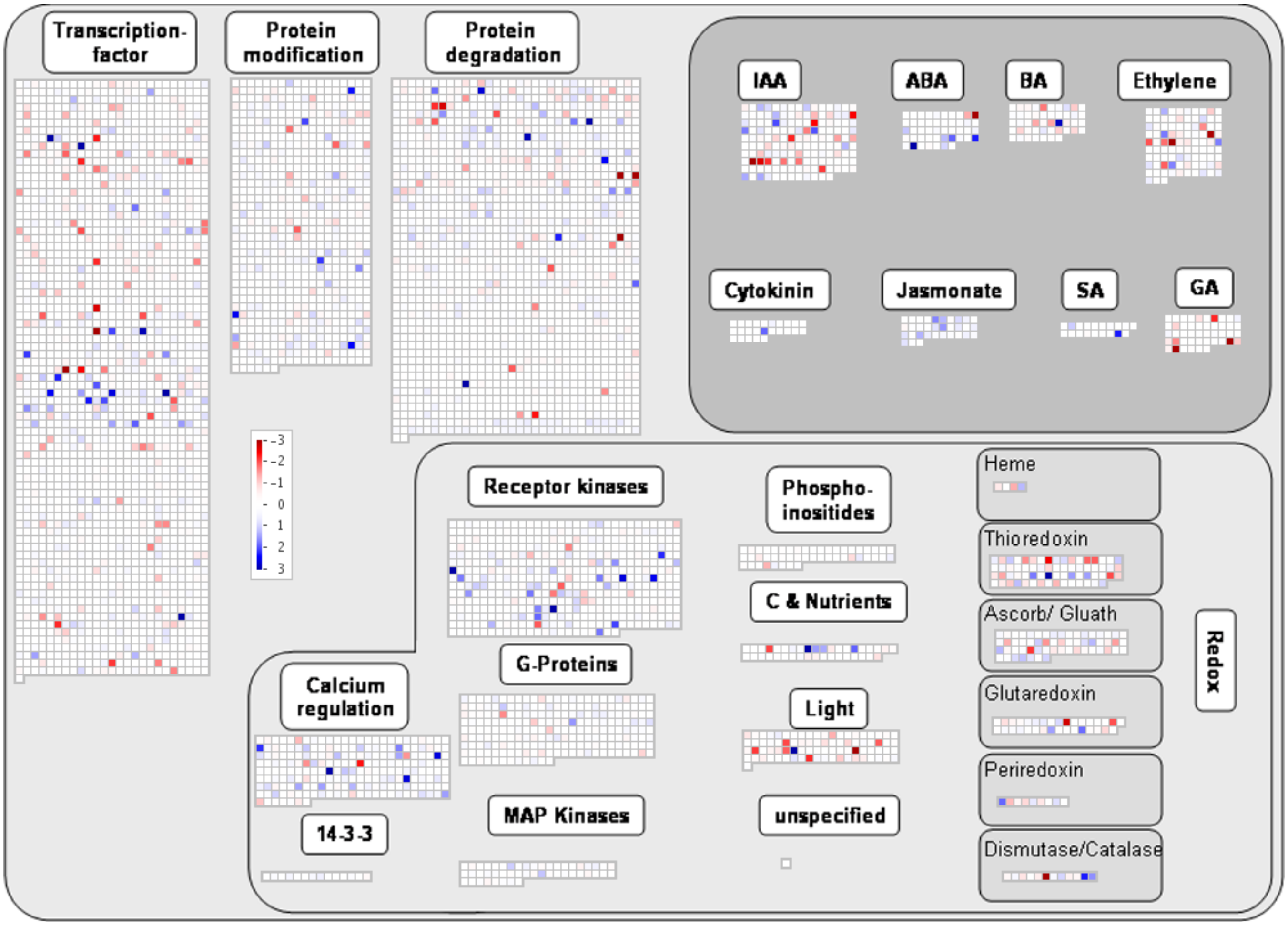

Figure f: Overview of gene regulation
