# Supplementary material for: Transcriptional responses to polycyclic aromatic hydrocarbon-induced stress in Arabidopsis thaliana reveal the involvement of hormone and defense signaling pathways
Source: BMC Plant Biol. 2010 Apr 7;10:59. doi: 10.1186/1471-2229-10-59 (PMC2923533; doi:10.1186/1471-2229-10-59)
Supplement: Additional file 5 — Phenanthrene induced changes in gene expression. Arabidopsis seedlings were grown in absence (CTR) or presence (PHE) of 0.25 mM phenanthrene for 21 days and total RNA was extracted. Microarray analysis was carried out as described in the Methods section. Columns CTR (mean microarray signal from control plants), PHE (mean microarray signal from phenanthrene-treated plants), and Fold-change (PHE/CTR) are log2 transformed. [file 1471-2229-10-59-S5.PDF]

Arabidopsis seedlings were grown in absence (CTR) or presence (PHE) of 0.25 mM phenanthrene for 21 days and total RNA was extracted. Microarray analysis was carried out as described in the Methods section. Columns CTR (mean microarray signal from control plants), PHE (mean microarray signal from phenanthrene-treated plants), and Fold-change (PHE/CTR) are $\log_2$ transformed.

AGI	*p*-value	Fold-change	CTR	PHE	Description of gene product
**Abscisic acid regulated genes**					
At2g33380	$8.4e-5$	3.4	4.4	7.8	Calcium-binding protein (RD20). Induced by NaCl, ABA and dessication
At3g02480	$4.8e-5$	5.4	3.5	8.9	ABA-responsive protein-related
At5g13200	0.00069	2.3	4.9	7.2	ABA-responsive protein-related
At5g59310	0.0014	5.5	4.6	10.1	Lipid transfer protein 4 (LTP4). Strongly up-regulated by ABA
**Auxin regulated genes**					
At2g22670	0.00054	-1.1	9.3	8.2	Auxin-responsive protein / IAA-induced protein 8 (IAA8)
At5g54490	0.53	-0.2	5.6	5.4	PINOID (PID)-binding protein. Induced by auxin
At5g54500	0.0046	1.7	9.4	11.1	FMN binding quinone reductase, putative. A primary auxin-response gene
At5g65670	0.098	0.5	8.2	8.6	Auxin-responsive protein / IAA induced protein 9 (IAA9). Transcription factor
**Cytochrome P450 enzymes**					
At2g24180	0.00071	1.4	6.7	8.1	Cytochrome P450 (CYP71B6)
At3g14620	$5.2e-5$	1.8	6.5	8.4	Cytochrome P450, putative (CYP72A8)
At3g26830	0.00026	3.7	3.4	7.1	Cytochrome P450, putative (CYP71B15). Camalexin bs, response to ABA
At4g39950	0.011	1.8	5.9	7.7	Cytochrome P450, putative (CYP79B2). Converts tryptophan to indo-3-acetaldoxime, a precursor to IAA, indole glucosinolates, and camalexin
At5g05690	0.00022	-1.8	8.5	6.8	Cytochrome P450 (CYP90A1). Involved in brassinolide bs
At5g24160	0.00024	2.8	5.2	8.0	Squalene monooxygenase 1,2 / squalene epoxidase 1,2 (SQP1,2). Sterol bs
**Defense related genes**					
At1g75040	$2.6e-5$	6.1	3.1	9.2	Pathogenesis-related protein 5 (PR-5). Response to other organism, UV-B; regulation of anthocyanin bs
At2g14610	$3.8e-6$	7.8	3.6	11.3	Pathogenesis-related protein 1 (PR-1). Response to pathogens and SA
At2g26020	$3.9e-6$	5.1	3.7	8.7	Plant defensin-fusion protein, putative (PDF1.2b)
At2g43510	$2.6e-5$	5.2	5.0	10.2	Defensin-like (DEFL) protein. Putative trypsin inhibitor, defense against herbivory
At2g43570	$4.0e-7$	6.1	3.5	9.6	Endochitinase, putative. Response to other organism
At3g12500	0.02	1.3	6.6	7.9	Basic endochitinase. Involved in ET/JA mediated signaling
At3g51240	$1.6e-5$	2.2	6.1	8.3	Naringenin 3-dioxygenase / flavanone 3-hydroxylase. Flavonoid bs; response to UV-B

AGI	*p*-value	Fold-change	CTR	PHE	Description of gene product
At4g11650	0.0041	2.1	8.4	10.5	Osmotin-like protein (OSM34) Thaumatin family. Response to bacterium
At5g08640	0.0023	2.2	5.4	7.6	Flavonol synthase 1 (FLS1). Flavonoid bs; response to wounding and JA
At5g13930	$5.4e-5$	2.2	6.9	9.1	Chalcone synthase (CHS). Flavonoid bs; defense, communication; regulation of auxin transport; response to oxidative stress, gravity, wounding, JA and UV-B
At5g44420	0.00049	4.0	4.1	8.2	Plant defensin protein, putative (PDF1.2a). Response to insect, ET and JA; not responsive to SA, but JA and SA can synergistically enhance expression
**Ethylene regulated genes**					
At1g62380	0.0071	-1.0	9.5	8.5	ACC oxidase, putative (ACO2). Induced by ET
At2g05520	0.00065	1.7	11.0	12.7	Glycine-rich protein (GRP). Induced in response to ABA, SA and ET; repressed in response to dessication
At3g04720	0.00056	2.2	8.0	10.2	Hevein-like protein (HEL). Induced in response to ET, JA, virus; chitin binding
At3g16770	0.01	-1.4	8.4	7.0	Member of the ERF/AP2 transcription factor family (RAP2.3). Transcriptional activator; overexpression causes upregulation of PDF1.2 and GST6
At4g11280	0.00017	-1.6	7.7	6.1	ACC synthase 6 (ACS6). Response to auxin, ET, JA and wounding
**Genes involved in conjugation of xenobiotics**					
At1g17170	0.0044	2.1	6.7	8.8	Glutathione S-transferase, putative (ATGSTU24) (induced in roots by TNT)
At2g02390	0.0035	1.6	6.8	8.4	Glutathione S-transferase (GSTZ1)
At2g16890	$1.3e-7$	3.8	4.1	7.9	UDP-glucoronosyl and UDP-glucosyl transferase
At2g43820	0.00028	3.1	5.2	8.3	UDP-glucoronosyl and UDP-glucosyl transferase. Induced by SA, virus, fungus and bacteria; involved in tryptophan bs
**Genes involved in oxidative stress defense**					
At1g08830	0.0018	2.3	8.8	11.1	Cytoplasmic copper/zinc superoxide dismutase (CSD1). Can detoxify superoxide radicals; response to oxidative stress, copper ion, iron ion
At2g28190	0.083	1.6	8.6	10.2	Chloroplastic copper/zinc superoxide dismutase (CSD2). Can detoxify superoxide radicals, response to oxidative stress, copper ion, iron ion

AGI	*p*-value	Fold-change	CTR	PHE	Description of gene product
At4g09010	$4.1e-6$	-1.5	9.5	8.0	L-ascorbate peroxidase, putative (APX4). Response to oxidative stress
At4g25100	0.00019	-4.0	10.3	6.3	Iron superoxide dismutase (FSD1). Response to oxidative stress
At5g47910	0.0089	1.3	6.0	7.3	Respiratory burst oxidase protein D (RbohD) / NADPH oxidase. Response to heat, oxygen, reactive oxygen species; negative regulation of progr. cell death
**Gibberellic acid regulated genes**					
At1g22690	0.00076	-4.2	9.2	5.0	GA -responsive protein, putative. GA regulated
At1g74670	0.00066	-3.8	10.7	6.9	GA -responsive protein, putative. GA regulated
**Jasmonic acid regulated genes**					
At1g55020	0.033	1.3	5.9	7.2	Lipoxygenase (LOX1). Confers resistance to Xanthomonas campestris
At3g45140	0.006	1.7	7.4	9.0	Lipoxygenase (LOX2). JA bs; response to JA
At4g01370	0.093	0.3	6.8	7.1	MAP kinase. Response to cold, hyperosmotic response, fungus, salt stress
**Others**					
At1g77120	$9.3e-7$	2.9	5.4	8.3	Alcohol dehydrogenase (ADH) / Anaerobic response polypeptide (ANP). Response to osmotic stress, hypoxia; cellular respiration.
At2g29350	$7.7e-8$	4.8	2.8	7.6	Senescence-associated gene (SAG13); alcohol dehydrogenase
**Transcription factors**					
At1g71030	0.0027	-3.1	8.5	5.4	Myb family transcription factor, putative
At4g31800	0.0019	3.5	4.3	7.8	WRKY18 transcription factor
At4g38620	0.0031	2.9	5.2	8.1	Myb family transcription factor (MYB4). Repressor of gene expression; response to salt stress, UV-B, ABA, auxin, ET, GA, JA, SA stimulus, and to cadmium ion.
At5g13080	$7.3e-5$	2.5	4.5	7.0	WRKY75 transcription factor
**Transporters**					
At1g11260	0.00043	-2.3	9.2	6.9	Glucose transporter (STP1)
At3g53480	0.0033	1.6	6.4	8.0	ABC transporter family protein
At4g04770	0.00028	-2.1	8.6	6.5	ATP-binding-cassette transporter (ABC1). Regulation of iron homeostasis
At4g19690	0.00072	4.2	4.3	8.5	Iron-responsive transporter (IRT1)

**AGI**	***p*-value**	**Fold-change**	**CTR**	**PHE**	**Description of gene product**
At5g50200	0.013	2.8	4.3	7.1	High-affinity nitrate transporter. Up-regulated by nitrate; involved in JA-independent wound signal transduction.
