# Supplementary material for: Transcriptional responses to polycyclic aromatic hydrocarbon-induced stress in Arabidopsis thaliana reveal the involvement of hormone and defense signaling pathways
Source: BMC Plant Biol. 2010 Apr 7;10:59. doi: 10.1186/1471-2229-10-59 (PMC2923533; doi:10.1186/1471-2229-10-59)
Supplement: Additional file 6 — Heatmap gene details. This .html file details the contents of Figure 2. Prior to clustering, the full set of microarrays was batch-normalized as described in the Methods section; consequently, the phenanthrene experiment microarray values in this file differ slightly from the values elsewhere in this report. [file 1471-2229-10-59-S6.HTML]

Phenanthrene microarray compared with published datasets.


# Phenanthrene microarray compared with published datasets.

| index | probe | GenBank | Symbol | PubMed | ctk | ga3 | bra | avg | css | aba | pac | pav | eoi | oss | pvi | avr | pha | sen | ps1 | bot | PHE | pst | uvs | gts | oxs | acc | iaa | mja | ag3 | o3 | tib | sa | Description |
| --- | --- | --- | --- | --- | --- | --- | --- | --- | --- | --- | --- | --- | --- | --- | --- | --- | --- | --- | --- | --- | --- | --- | --- | --- | --- | --- | --- | --- | --- | --- | --- | --- | --- |
| 1031 | 258845\_at | AT3G03150 |  |  | -0.5 | 0 | 0.1 | 0.2 | -0.1 | 1.2 | 0 | -0.2 | -0.9 | 0.7 | -0.1 | -0.3 | -0.9 | -0.7 | 0.1 | -0.5 | -1.8 | -0.3 | 0.1 | 0 | -0.4 | -0.1 | 0 | 0.6 | 0.4 | -0.4 | -0.2 | -0.4 | similar to unknown protein [Arabidopsis thaliana] (TAIR:AT5G17165.1); similar to seed specific protein Bn15D1B [Brassica napus] (GB:AAP37967.1) |
| 1030 | 253317\_at | AT4G33960 |  |  | -0.8 | 0 | -0.6 | 0.7 | -0.4 | 0.8 | 0 | -0.6 | -1.1 | 0.8 | -0.5 | -0.4 | -1 | -1.2 | 0 | -1.1 | -1.3 | -0.3 | -0.7 | -0.3 | 0 | 0 | -0.4 | 0.3 | 0.2 | -0.9 | -0.2 | -0.9 | similar to unknown protein [Arabidopsis thaliana] (TAIR:AT2G15830.1) |
| 1029 | 262399\_at | AT1G49500 |  |  | -0.4 | -0.1 | -1 | 0.2 | -0.1 | 0.9 | 0.3 | -0.4 | -1 | 0.8 | -0.1 | 0 | -0.8 | -1.7 | 0.7 | -0.6 | -1 | -0.4 | 0 | 0.2 | -1 | -0.1 | -0.1 | 0.4 | -0.5 | -0.3 | -1.1 | -1.1 | similar to unknown protein [Arabidopsis thaliana] (TAIR:AT3G19030.1) |
| 1028 | 247903\_at | AT5G57340 |  |  | -0.2 | -0.6 | 0.2 | -1.3 | -0.1 | 2.2 | 0.8 | -0.7 | -2 | -3.3 | -0.7 | 0.4 | -1.1 | -1.9 | 1.2 | -1.6 | -2 | -0.3 | -0.7 | 0.6 | -0.7 | 0.7 | 1.6 | 1.3 | -1.3 | 0.1 | -0.8 | -2.3 | similar to unknown protein [Arabidopsis thaliana] (TAIR:AT5G67390.2); similar to conserved hypothetical protein [Medicago truncatula] (GB:ABE83997.1) |
| 1027 | 259983\_at | AT1G76490 | HMG1, HMGR1 | 21 | -0.3 | -0.1 | 0.1 | 0 | 0.2 | 0.5 | 0.3 | -0.3 | -0.8 | 1 | -0.4 | 0.9 | -0.4 | -0.7 | 1.8 | 0.8 | -1.2 | 0.4 | -0.2 | 0 | 0.5 | -0.6 | -0.2 | 0.8 | -1 | 0.1 | -0.7 | -0.4 | Encodes a 3-hydroxy-3-methylglutaryl coenzyme A reductase, which is involved in melavonate biosynthesis and performs the first committed step in isoprenoid biosynthesis. Expression is activated in dark in leaf tissue but not controlled by light in the root (confine, Encodes a 3-hydroxy-3-methylglutaryl coenzyme A reductase, which is involved in melavonate biosynthesis and performs the first committed step in isoprenoid biosynthesis. Expression is activated in dark in leaf tissue but not controlled by light in the root (confine |
| 1026 | 248793\_at | AT5G47240 | atnudt8 | 1 | -0.2 | 0.2 | 1.4 | 0.8 | 1.2 | 1 | -0.7 | -0.4 | -1.1 | 3.8 | 0.2 | -0.7 | -0.8 | -1.4 | -0.3 | 0.7 | -3.1 | 0 | -0.8 | -1.4 | 1 | -1 | -1.2 | 6.9 | 0.8 | 1.8 | -0.9 | -1.9 | ATNUDT8 (Arabidopsis thaliana Nudix hydrolase homolog 8); hydrolase; Identical to Nudix hydrolase 8 (EC 3.6.1.-) (AtNUDT8) (NUDT8) [Arabidopsis Thaliana] (GB:Q8L7W2;GB:Q8LAJ0;GB:Q9LVT5); similar to ATNUDT2 (Arabidopsis thaliana Nudix hydrolase homolog 2), ADP-ribose diphosphatase/ NAD binding / hydrolase [Arabidopsis thaliana] (TAIR:AT5G47650.1); similar to hydrolase, NUDIX family protein [Brassica oleracea] (GB:ABD64957.1); contains InterPro domain NUDIX hydrolase; (InterPro:IPR000086); contains InterPro domain Anti-sense to fibroblast growth factor protein GFG; (InterPro:IPR003293) |
| 1025 | 258181\_at | AT3G21670 |  |  | 1.8 | -1 | -1 | 0.9 | 0.1 | 1.5 | 0.1 | -0.7 | -1.1 | 0.1 | 0.4 | -0.2 | -1.2 | 0.4 | 1.5 | -1.1 | -1.9 | 1 | -0.7 | -0.4 | -0.6 | -1.1 | -2.8 | 1.3 | -1.1 | 0.4 | -0.6 | -1.1 | nitrate transporter (NTP3); similar to nitrate transporter (NTP2) [Arabidopsis thaliana] (TAIR:AT2G26690.1); similar to Os02g0580900 [Oryza sativa (japonica cultivar-group)] (GB:NP\_001047240.1); similar to nitrate transporter [Prunus persica] (GB:BAC81420.1); similar to nitrate transporter [Prunus persica] (GB:BAD22820.1); contains InterPro domain TGF-beta receptor, type I/II extracellular region; (InterPro:IPR000109) |
| 1024 | 256772\_at | AT3G13750 | BGAL1 | 3 | -0.4 | -0.2 | -0.2 | 0.1 | -0.1 | -1 | 0.1 | 0.1 | -0.4 | -5.9 | -0.2 | 0.1 | -0.6 | -3.5 | 0.7 | -3.4 | -1.8 | -2.8 | 0.1 | -0.8 | -0.6 | -0.7 | -0.5 | -1.3 | -1.1 | -1.4 | -1.3 | -1.7 | beta-galactosidase, glycosyl hydrolase family 35 |
| 1023 | 253305\_at | AT4G33666 |  |  | -0.7 | -0.3 | -0.5 | 0.2 | 0 | 1.1 | 0.5 | -0.3 | -0.8 | -4.3 | -0.4 | -0.3 | -0.4 | -3.2 | 1.4 | -1.8 | -1.7 | -0.6 | -0.4 | -0.6 | -0.9 | -0.2 | -0.9 | -0.6 | -1.3 | -0.8 | 1.3 | -0.7 | unknown protein |
| 1022 | 255008\_at | AT4G10060 |  |  | -0.1 | -0.2 | 0.2 | 0.1 | -0.1 | 0.2 | 0.1 | 0 | -0.1 | -1.5 | 0 | -0.3 | -0.5 | -0.7 | 0.4 | -0.3 | -2.1 | 0.2 | -0.2 | -0.5 | -0.7 | -0.2 | -0.4 | 1.2 | -0.5 | -0.7 | -0.3 | -0.6 | similar to unknown protein [Arabidopsis thaliana] (TAIR:AT5G49900.1); similar to unknown protein [Arabidopsis thaliana] (TAIR:AT1G33700.2); similar to unknown protein [Arabidopsis thaliana] (TAIR:AT3G24180.2); similar to unknown protein [Oryza sativa] (GB:AAL31035.1); similar to Os11g0242100 [Oryza sativa (japonica cultivar-group)] (GB:NP\_001067588.1); similar to At5g49900 [Oryza sativa (japonica cultivar-group)] (GB:AAX95400.1); contains InterPro domain Protein of unknown function DUF608; (InterPro:IPR006775) |
| 1021 | 267523\_at | AT2G30600 |  |  | -0.3 | -0.2 | 0.2 | -0.4 | 1.1 | -0.9 | 0.1 | 0.5 | -0.5 | 1 | -0.4 | -0.1 | -0.8 | -0.9 | -0.6 | -0.5 | -2.5 | -1.3 | 1.1 | -0.6 | 0 | 0.4 | -0.2 | 0.9 | 0 | -0.1 | -0.9 | -0.8 | BTB/POZ domain-containing protein; similar to BTB/POZ domain-containing protein [Arabidopsis thaliana] (TAIR:AT2G46260.1); similar to unknown protein [Oryza sativa (japonica cultivar-group)] (GB:AAV43802.1); contains InterPro domain BTB/Kelch-associated; (InterPro:IPR011705); contains InterPro domain Coagulation factor 5/8 type, C-terminal; (InterPro:IPR000421); contains InterPro domain BTB; (InterPro:IPR000210); contains InterPro domain BTB/POZ; (InterPro:IPR013069), protein binding; similar to BTB/POZ domain-containing protein [Arabidopsis thaliana] (TAIR:AT2G46260.1); similar to unknown protein [Oryza sativa (japonica cultivar-group)] (GB:AAV43802.1); contains InterPro domain BTB/Kelch-associated; (InterPro:IPR011705); contains InterPro domain Coagulation factor 5/8 type, C-terminal; (InterPro:IPR000421); contains InterPro domain BTB; (InterPro:IPR000210); contains InterPro domain BTB/POZ; (InterPro:IPR013069) |
| 1020 | 248502\_at | AT5G50450 |  |  | -1.6 | -0.3 | 0 | 0.3 | 0.3 | -0.6 | 0 | 0.1 | -0.6 | 0.2 | 0.4 | -0.4 | -1.2 | -0.3 | -0.5 | -0.2 | -2.6 | -0.8 | 0.1 | 0.1 | -0.1 | 0.3 | -0.3 | -0.1 | -0.3 | -1 | -0.4 | -0.8 | zinc finger (MYND type) family protein; similar to zinc finger (MYND type) family protein / F-box family protein [Arabidopsis thaliana] (TAIR:AT1G67340.1); similar to hypothetical protein [Oryza sativa (japonica cultivar-group)] (GB:BAD88375.1); similar to Os01g0921800 [Oryza sativa (japonica cultivar-group)] (GB:NP\_001045230.1); similar to Os04g0385600 [Oryza sativa (japonica cultivar-group)] (GB:NP\_001052618.1); contains InterPro domain Zinc finger, MYND-type; (InterPro:IPR002893); contains InterPro domain Tetratricopeptide-like helical; (InterPro:IPR011990); contains InterPro domain Sel1-like; (InterPro:IPR006597) |
| 1019 | 252238\_at | AT3G49960 |  |  | -0.4 | -0.1 | 0.5 | -0.3 | 0.1 | -0.6 | -0.2 | 0.1 | -0.1 | 0 | 0 | 0 | -0.1 | -0.1 | 0 | 0 | -4.3 | 0 | 0 | 0 | 0 | 0.8 | 0.1 | 0.2 | -7.9 | -0.1 | -8 | -2.7 | peroxidase, putative; Identical to Peroxidase 35 precursor (EC 1.11.1.7) (Atperox P35) (ATP21a) (PER35) [Arabidopsis Thaliana] (GB:Q96510); similar to peroxidase 73 (PER73) (P73) (PRXR11) [Arabidopsis thaliana] (TAIR:AT5G67400.1); similar to peroxidase 1 [Artemisia annua] (GB:AAO45182.1); contains InterPro domain Haem peroxidase; (InterPro:IPR010255); contains InterPro domain Haem peroxidase, plant/fungal/bacterial; (InterPro:IPR002016); contains InterPro domain Plant peroxidase; (InterPro:IPR000823) |
| 1018 | 251192\_at | AT3G62720 | ATXT1, XT1, XXT1 | 2 | -2.1 | 0.1 | 1.1 | 0.1 | 0.4 | -0.3 | -0.6 | 0 | -0.5 | -0.9 | -0.1 | 1.4 | -0.2 | -2.3 | -0.4 | 0.8 | -1.9 | 0.2 | -0.1 | -0.1 | 0.3 | -0.7 | 0.5 | -0.5 | 0.1 | 1.6 | -0.2 | -0.8 | Encodes a protein with xylosyltransferase activity, which is specific for UDP-xylose as donor substrate and for oligosaccharides with a degree of polymerization >4. Although the enzyme utilizes either cellopentaose or cellohexaose, its activity is four-fold higher with cellohexaose as an acceptor compared to cellopentaose. The enzyme is able to add several xylosyl residues to the acceptor forming mono-, di- and trixylosylated polysaccharides., Encodes a protein with xylosyltransferase activity, which is specific for UDP-xylose as donor substrate and for oligosaccharides with a degree of polymerization >4. Although the enzyme utilizes either cellopentaose or cellohexaose, its activity is four-fold higher with cellohexaose as an acceptor compared to cellopentaose. The enzyme is able to add several xylosyl residues to the acceptor forming mono-, di- and trixylosylated polysaccharides., Encodes a protein with xylosyltransferase activity, which is specific for UDP-xylose as donor substrate and for oligosaccharides with a degree of polymerization >4. Although the enzyme utilizes either cellopentaose or cellohexaose, its activity is four-fold higher with cellohexaose as an acceptor compared to cellopentaose. The enzyme is able to add several xylosyl residues to the acceptor forming mono-, di- and trixylosylated polysaccharides. |
| 1017 | 257635\_at | AT3G26280 | CYP71B4 | 1 | -0.6 | -0.6 | -1.1 | 0.1 | 0 | 1.4 | 0.6 | -0.1 | 0 | 1.8 | 0 | 1 | 1.1 | 1.3 | 1.5 | 0.1 | -1.2 | -0.6 | 0.3 | 0.4 | -0.5 | -0.5 | -0.8 | -0.5 | 1.3 | -1.4 | 0.4 | -0.4 | cytochrome P450 monooxygenase |
| 1016 | 254874\_at | AT4G11570 |  |  | -0.4 | -0.3 | -0.4 | 0 | -0.1 | 1.6 | 0 | -0.4 | -0.6 | 0.8 | 0.1 | 1.2 | 0.7 | 0.1 | 1.8 | 0.1 | -0.9 | -0.5 | -0.3 | 0.4 | 0.4 | -0.2 | -0.4 | -0.1 | 0.6 | 1.2 | 0.3 | 0 | haloacid dehalogenase-like hydrolase family protein; similar to haloacid dehalogenase-like hydrolase family protein [Arabidopsis thaliana] (TAIR:AT3G10970.1); similar to Histidine acid phosphatase; HAD-superfamily hydrolase subfamily IA, variant 3 [Medicago truncatula] (GB:ABE92120.1); contains InterPro domain Haloacid dehalogenase-like hydrolase; (InterPro:IPR005834) |
| 1015 | 253161\_at | AT4G35770 | ATSEN1, DIN1, SEN1 | 15 | -1.6 | 0 | -1 | -0.4 | -0.5 | 0.8 | 1.7 | -0.3 | -1.1 | 3.3 | -0.1 | 3.2 | 2 | 0.6 | 3.9 | 0.3 | -3.3 | -1.4 | 1.8 | 0 | -1.3 | 0.4 | 0.9 | -0.5 | 2 | 1.4 | -0.5 | -0.8 | Senescence-associated gene that is strongly induced by phosphate starvation. Transcripts are differentially regulated at the level of mRNA stability at different times of day. mRNAs are targets of the mRNA degradation pathway mediated by the downstream (DST) instability determinant., Senescence-associated gene that is strongly induced by phosphate starvation. Transcripts are differentially regulated at the level of mRNA stability at different times of day. mRNAs are targets of the mRNA degradation pathway mediated by the downstream (DST) instability determinant., Senescence-associated gene that is strongly induced by phosphate starvation. Transcripts are differentially regulated at the level of mRNA stability at different times of day. mRNAs are targets of the mRNA degradation pathway mediated by the downstream (DST) instability determinant. |
| 1014 | 264210\_at | AT1G22640 | ATMYB3, MYB3 | 6 | -0.2 | -0.2 | 0.1 | 0.2 | -0.1 | 3.4 | 0.5 | -0.3 | -0.7 | 1.7 | -0.1 | 0.8 | 0.5 | 1.8 | 1.6 | -0.4 | -2.1 | -0.5 | -0.2 | -0.1 | -0.8 | 0 | 0.2 | -0.1 | 0.6 | -0.1 | 0 | 0.8 | MYB-type transcription factor (MYB3) that represses phenylpropanoid biosynthesis gene expression, MYB-type transcription factor (MYB3) that represses phenylpropanoid biosynthesis gene expression |
| 1013 | 267550\_at | AT2G32800 | AP4.3A | 1 | -1.6 | 0.4 | 0.3 | 0 | 0.4 | 3.1 | 0 | 0.4 | 1.2 | 3.3 | 0.5 | 2.3 | 1.7 | 2.1 | 1.4 | 0 | 2.4 | 0.1 | -0.2 | -0.2 | -0.1 | -1.1 | -0.2 | -0.2 | 1.1 | 1.9 | -0.8 | 0 | AP4.3A; ATP binding / protein kinase; similar to lectin protein kinase, putative [Arabidopsis thaliana] (TAIR:AT3G53810.1); similar to putative receptor kinase Lecrk [Oryza sativa (japonica cultivar-group)] (GB:BAD10609.1); similar to putative receptor-type protein kinase LRK1 [Oryza sativa (japonica cultivar-group)] (GB:BAD08845.1); contains InterPro domain Serine/threonine protein kinase; (InterPro:IPR002290); contains InterPro domain Protein kinase-like; (InterPro:IPR011009); contains InterPro domain Protein kinase; (InterPro:IPR000719); contains InterPro domain Tyrosine protein kinase; (InterPro:IPR001245); contains InterPro domain Serine/threonine protein kinase, active site; (InterPro:IPR008271) |
| 1012 | 265511\_at | AT2G05540 |  |  | 0.1 | 0.5 | -0.5 | 0.2 | 0.4 | 2.8 | -0.2 | -0.3 | 1.4 | 1.8 | 0.6 | 1.2 | 0.7 | 2.4 | 2.7 | 0.6 | -4.4 | -1.2 | 0.1 | -0.5 | -0.8 | -0.3 | -0.1 | -0.3 | 0.6 | 0.5 | -0.8 | 0.3 | glycine-rich protein; similar to glycine-rich protein [Arabidopsis thaliana] (TAIR:AT2G05530.1); similar to Glycine rich [Medicago truncatula] (GB:ABE77579.1); contains InterPro domain Glycine rich; (InterPro:IPR010800) |
| 1011 | 252563\_at | AT3G45970 | ATEXLA1, ATEXPL1, ATHEXP, EXPL1 | 3 | -1.9 | 0.6 | 2 | 0.9 | 0.8 | 3.3 | -1.4 | -0.5 | -2.1 | 2.7 | 0.6 | 1.7 | 1.7 | 1.6 | 0.2 | -2.6 | -1.1 | -3.3 | 1.1 | -1 | 0.4 | -2.1 | 0.3 | -2.9 | -1.6 | 0.1 | -0.2 | -0.9 | member of EXPANSIN-LIKE. Naming convention from the Expansin Working Group (Kende et al, 2004. Plant Mol Bio), member of EXPANSIN-LIKE. Naming convention from the Expansin Working Group (Kende et al, 2004. Plant Mol Bio), member of EXPANSIN-LIKE. Naming convention from the Expansin Working Group (Kende et al, 2004. Plant Mol Bio), member of EXPANSIN-LIKE. Naming convention from the Expansin Working Group (Kende et al, 2004. Plant Mol Bio) |
| 1010 | 247925\_at | AT5G57560 | TCH4, XTH22 | 12 | -2.6 | 1.3 | 4.6 | 1 | 0.1 | 3 | -2.9 | 0.4 | 0.8 | 3.9 | 0.5 | 2.5 | 1.2 | -0.3 | 0.1 | -1.7 | -1.3 | -2.1 | 1 | 2.2 | 1.6 | -2.1 | 0.9 | -0.9 | 1.4 | 4 | 0.7 | -0.3 | Encodes a cell wall-modifying enzyme, rapidly upregulated in response to environmental stimuli, Encodes a cell wall-modifying enzyme, rapidly upregulated in response to environmental stimuli |
| 1009 | 267509\_at | AT2G45660 | AGL20, SOC1 | 46 | -1.5 | 0.8 | 0.8 | -0.1 | 0.2 | 3.9 | -0.3 | 0.4 | -0.1 | 0.9 | 0.5 | -0.3 | 0 | 1.3 | 0.7 | -0.8 | -2.3 | -1.1 | 0.4 | 0.3 | -0.4 | 0.9 | 0.1 | 0.9 | -1.2 | -1 | -1.2 | 0.8 | Controls flowering and is required for CO to promote flowering. It acts downstream of FT. Overexpression of (SOC1) AGL20 suppresses not only the late flowering of plants that have functional FRI and FLC alleles but also the delayed phase transitions during the vegetative stages of, Controls flowering and is required for CO to promote flowering. It acts downstream of FT. Overexpression of (SOC1) AGL20 suppresses not only the late flowering of plants that have functional FRI and FLC alleles but also the delayed phase transitions during the vegetative stages of |
| 1008 | 249922\_at | AT5G19140 | AILP1, ATAILP1 | 2 | -1.1 | 0.2 | 0.8 | 0.3 | -0.1 | 0.2 | 0.2 | 0.1 | 0.2 | -0.6 | 0.1 | 0.2 | 0.4 | 0.8 | 0.3 | -0.6 | -0.7 | -0.5 | 0.2 | 0 | -0.7 | 0.2 | 0.1 | -0.4 | 0 | -1 | -0.5 | 0.3 | auxin/aluminum-responsive protein, putative; similar to unknown protein [Arabidopsis thaliana] (TAIR:AT5G43830.1); similar to aluminum-induced [Brassica napus] (GB:BAA25999.1); contains domain ASPARAGINE SYNTHETASE (PTHR11772); contains domain no description (G3D.3.60.20.10); contains domain N-terminal nucleophile aminohydrolases (Ntn hydrolases) (SSF56235), auxin/aluminum-responsive protein, putative; similar to unknown protein [Arabidopsis thaliana] (TAIR:AT5G43830.1); similar to aluminum-induced [Brassica napus] (GB:BAA25999.1); contains domain ASPARAGINE SYNTHETASE (PTHR11772); contains domain no description (G3D.3.60.20.10); contains domain N-terminal nucleophile aminohydrolases (Ntn hydrolases) (SSF56235) |
| 1007 | 251356\_at | AT3G61060 | AtPP2-A13, AtPP2-A13 | 1 | -1.1 | -0.2 | 0.7 | -0.1 | -0.1 | 4.9 | 0.8 | -0.2 | -0.9 | 0.9 | -0.3 | 1.5 | 1.2 | 3.4 | 1.5 | -2.8 | -2.7 | -1.6 | 0.4 | -0.4 | -1.6 | -1 | -0.9 | 0.9 | -0.8 | -0.6 | -2.9 | -1.8 | ATPP2-A13; similar to ATPP2-A12 (Phloem protein 2-A12) [Arabidopsis thaliana] (TAIR:AT1G12710.1); similar to Cyclin-like F-box; Galactose-binding like [Medicago truncatula] (GB:ABE86301.1); contains InterPro domain Cyclin-like F-box; (InterPro:IPR001810), ATPP2-A13; similar to ATPP2-A12 (Phloem protein 2-A12) [Arabidopsis thaliana] (TAIR:AT1G12710.1); similar to Cyclin-like F-box; Galactose-binding like [Medicago truncatula] (GB:ABE86301.1); contains InterPro domain Cyclin-like F-box; (InterPro:IPR001810); contains InterPro domain Galactose-binding like; (InterPro:IPR008979) |
| 1006 | 248622\_at | AT5G49360 | ATBXL1, BXL1 | 3 | -1.9 | 0.7 | 0.7 | 0.5 | 0.3 | 0.1 | 0.5 | -0.2 | -0.5 | -1.2 | 0 | 1.8 | 1 | 0.2 | 3.2 | -0.3 | -1.5 | -1.6 | 0.9 | -0.4 | -0.9 | -1 | -1.9 | -1.4 | -1.8 | -0.7 | -2 | -1.5 | encodes a beta-xylosidase located in the extracellular matrix. Gene is expressed specifically in tissues undergoing secondary wall thickening. This is a member of glycosyl hydrolase family 3 and has six other closely related members., encodes a beta-xylosidase located in the extracellular matrix. Gene is expressed specifically in tissues undergoing secondary wall thickening. This is a member of glycosyl hydrolase family 3 and has six other closely related members. |
| 1005 | 266656\_at | AT2G25900 | ATCTH | 1 | -0.4 | 0.5 | 0 | 0 | 0 | 1.4 | 1.1 | -0.2 | -0.7 | -3.1 | -0.2 | -2.2 | -1.7 | -2.4 | -2.5 | -3.2 | -1.9 | -3.8 | 0.2 | 0.6 | -0.3 | 0.1 | 0.8 | -0.6 | 0.6 | -0.2 | -0.1 | -0.6 | putative Cys3His zinc finger protein (ATCTH) mRNA, complete |
| 1004 | 249862\_at | AT5G22920 |  |  | -0.2 | 0.3 | 0.3 | -0.4 | 0.2 | -0.5 | 0.4 | -0.1 | -0.9 | -1 | -0.6 | -0.2 | -0.8 | -1.9 | -1 | -1.9 | -1.4 | -2.5 | 1.2 | -0.2 | -0.6 | -0.1 | -0.3 | -0.3 | 0 | -0.5 | -1.3 | -0.5 | zinc finger (C3HC4-type RING finger) family protein; similar to zinc finger (C3HC4-type RING finger) family protein [Arabidopsis thaliana] (TAIR:AT5G25560.1); similar to PGPD14 [Petunia x hybrida] (GB:AAD02556.1); contains InterPro domain Zinc finger, CHY-type; (InterPro:IPR008913); contains InterPro domain Zinc finger, RING-type; (InterPro:IPR001841) |
| 1003 | 262986\_at | AT1G23390 |  |  | -1.7 | 0.5 | -0.1 | -0.1 | 0.2 | -0.1 | 0.6 | 0.1 | 0.3 | -2 | -0.2 | 0.6 | 0.9 | -1.3 | -0.9 | -2.3 | -1.7 | -3 | 0.5 | 0.8 | -0.2 | 0.3 | 0 | -2.2 | 0.8 | -0.7 | -1.2 | 0.2 | kelch repeat-containing F-box family protein; similar to F-box family protein [Arabidopsis thaliana] (TAIR:AT3G24760.1); similar to Os09g0292900 [Oryza sativa (japonica cultivar-group)] (GB:NP\_001062790.1); similar to kelch repeat-containing F-box-like protein [Oryza sativa (japonica cultivar-group)] (GB:BAD19847.1); contains InterPro domain Cyclin-like F-box; (InterPro:IPR001810); contains InterPro domain Galactose oxidase, central; (InterPro:IPR011043); contains InterPro domain Kelch repeat; (InterPro:IPR006652) |
| 1002 | 259751\_at | AT1G71030 | ATMYBL2, MYBL2 | 6 | -1.5 | -1.5 | -3.4 | -1 | -0.3 | 0.7 | 1.6 | -0.1 | -0.9 | -1.5 | 0.5 | -1.4 | 0.1 | -0.2 | -0.4 | -1.4 | -3.2 | -2.4 | -0.1 | -0.1 | -0.9 | 1.6 | 2.1 | -1.2 | 3 | -0.9 | 1.6 | -1.4 | Encodes a putative myb family transcription factor. In contrast to most other myb-like proteins its myb domain consists of a single repeat. A proline-rich region potentially involved in transactivation is found in the C-terminal part of the protein. Its transcript accumulates mainly in leaves., Encodes a putative myb family transcription factor. In contrast to most other myb-like proteins its myb domain consists of a single repeat. A proline-rich region potentially involved in transactivation is found in the C-terminal part of the protein. Its transcript accumulates mainly in leaves. |
| 1001 | 247921\_at | AT5G57660 |  | 1 | -0.4 | 0.3 | -1.1 | 0 | -0.1 | 0.2 | 0.1 | -0.3 | -0.3 | -0.5 | 0.4 | -0.2 | 0 | -0.5 | 0.5 | -0.4 | -2.1 | -1 | 0.3 | 0.1 | -0.5 | 0.3 | 0.2 | 0.1 | 0.4 | 0 | -0.2 | -0.2 | zinc finger (B-box type) family protein; Identical to Zinc finger protein CONSTANS-LIKE 5 (COL5) [Arabidopsis Thaliana] (GB:Q9FHH8;GB:Q8L9H4;GB:Q93ZC8); similar to zinc finger (B-box type) family protein [Arabidopsis thaliana] (TAIR:AT5G24930.1); similar to Zinc finger, B-box; CCT [Medicago truncatula] (GB:ABE83395.1); contains InterPro domain Zinc finger, CONSTANS-type; (InterPro:IPR002926); contains InterPro domain CCT; (InterPro:IPR010402); contains InterPro domain Zinc finger, B-box; (InterPro:IPR000315) |
| 1000 | 260856\_at | AT1G21910 |  | 2 | -1.8 | -0.4 | 1.1 | -0.4 | 0.6 | -2.4 | -0.1 | -0.2 | 0.1 | -0.5 | -0.8 | 1.3 | 0.7 | -1.5 | -1.7 | -4.9 | -1.6 | -3.7 | 1.6 | 2.1 | 1.4 | 0.8 | 0.3 | -3.6 | -2 | 0.9 | -0.8 | -0.8 | encodes a member of the DREB subfamily A-5 of ERF/AP2 transcription factor family. The protein contains one AP2 domain. There are 15 members in this subfamily including RAP2.1, RAP2.9 and RAP2.10. |
| 999 | 245757\_at | AT1G35140 | EXL7, PHI-1 | 1 | -5.2 | 0 | 3.8 | 0.9 | 0 | 0 | -0.1 | 0.7 | 0 | -1.5 | 1 | 4.5 | 3.5 | 0 | 0.6 | -2.5 | -4.1 | -2.4 | 2 | 0.5 | 2 | 0 | 0 | 0 | -0.3 | 2.1 | 0.3 | -0.3 | At1g35140 (At1g35140/T32G9\_32) mRNA, complete cds, At1g35140 (At1g35140/T32G9\_32) mRNA, complete cds |
| 998 | 259364\_at | AT1G13260 | RAV1 | 6 | -1.1 | 0.4 | -0.5 | -0.3 | -0.1 | -1.9 | 0 | 0.1 | 0.2 | -2.5 | 0.1 | -0.1 | 1 | 0.6 | 0.6 | -0.8 | -1 | -1.5 | 1.5 | 2.1 | 1 | 0.2 | 0.1 | 0.4 | -0.1 | 1.6 | -0.3 | 0.6 | Encodes an AP2/B3 domain transcription factor which is upregulated in response to low temperature. It contains a B3 DNA binding domain. It has circadian regulation and may function as a negative growth regulator. |
| 997 | 253061\_at | AT4G37610 | BT5 | 2 | -1.2 | 0.5 | 0.5 | 0.5 | -0.2 | -1.4 | 1.2 | 0.5 | -0.6 | -2.4 | 0.4 | 3.7 | 2.1 | 1.2 | 3.8 | -1.2 | -2.4 | -0.7 | 0.7 | 0.3 | -0.6 | 0 | -0.8 | 1.1 | 1.4 | 1 | -2.1 | -1.1 | BT5 (BTB and TAZ domain protein 5); protein binding / transcription regulator; similar to BT4 (BTB AND TAZ DOMAIN PROTEIN 4), protein binding [Arabidopsis thaliana] (TAIR:AT5G67480.2); similar to Zinc finger, TAZ-type; BTB/POZ [Medicago truncatula] (GB:ABE77424.1); contains InterPro domain Zinc finger, TAZ-type; (InterPro:IPR000197); contains InterPro domain BTB; (InterPro:IPR000210); contains InterPro domain BTB/POZ; (InterPro:IPR013069) |
| 996 | 246114\_at | AT5G20250 | DIN10 | 3 | -0.5 | -0.1 | 0.3 | 0.3 | 0.5 | -2.3 | 0.4 | 0.1 | -0.8 | -1.7 | -0.4 | 1.3 | 0.9 | 0.5 | 1.3 | -0.9 | -1.3 | -1 | 1.4 | -0.1 | -0.5 | 0 | -0.6 | -0.1 | 0.8 | 0.2 | -0.6 | -0.1 | encodes a member of glycosyl hydrolase family 36. Expression is induced within 3 hours of dark treatment, in senescing leaves and treatment with exogenous photosynthesis inhibitor. Induction of gene expression was suppressed in excised leaves supplied with sugar. The authors suggest that the gene's expression pattern is responding to the level of sugar in the cell. |
| 995 | 249923\_at | AT5G19120 |  |  | -0.8 | 0.1 | 0.1 | -0.2 | -0.1 | -2.7 | 0.6 | -0.2 | 0 | -2.3 | -0.3 | 1.6 | 1.2 | -1.2 | 1.9 | -1.5 | -1.7 | -1.7 | 0.5 | 0.7 | 0.7 | 0.5 | 0.4 | 0.5 | 0.7 | 0.6 | -0.3 | 0.1 | pepsin A; similar to extracellular dermal glycoprotein, putative / EDGP, putative [Arabidopsis thaliana] (TAIR:AT1G03220.1); similar to EDGP precursor [Daucus carota] (GB:BAA03413.1); contains InterPro domain Peptidase A1, pepsin; (InterPro:IPR001461); contains InterPro domain Peptidase aspartic, catalytic; (InterPro:IPR009007) |
| 994 | 258402\_at | AT3G15450 |  |  | -0.4 | 0.6 | 0.5 | -0.3 | 0 | 0.1 | 1.1 | 0 | -1 | -0.7 | 0 | 2.5 | 0.3 | 0 | 2 | -0.8 | -0.5 | -1.2 | 1 | 0 | -0.3 | 1.2 | 1.9 | 1.7 | 1.6 | 1.1 | 0 | -0.5 | similar to unknown protein [Arabidopsis thaliana] (TAIR:AT4G27450.1); similar to unknown [Glycine max] (GB:AAG00940.1); contains domain ASPARAGINE SYNTHETASE (PTHR11772); contains domain no description (G3D.3.60.20.10); contains domain N-terminal nucleophile aminohydrolases (Ntn hydrolases) (SSF56235), similar to unknown protein [Arabidopsis thaliana] (TAIR:AT4G27450.1); similar to unknown [Glycine max] (GB:AAG00940.1); contains domain no description (G3D.3.60.20.10); contains domain N-terminal nucleophile aminohydrolases (Ntn hydrolases) (SSF56235) |
| 993 | 246028\_at | AT5G21170 |  | 1 | -1.4 | 0 | 0.2 | 0.1 | 0.5 | -0.1 | 0.7 | -0.2 | -0.1 | -2.2 | -0.2 | 0.6 | 0.1 | -0.8 | 1.4 | -0.8 | -1.4 | -1.4 | 0.7 | -0.5 | -0.7 | 0.2 | 0.4 | -0.1 | 0.2 | 0.5 | -0.4 | -0.4 | 5'-AMP-activated protein kinase beta-2 subunit, putative; Identical to SNF1-related protein kinase regulatory subunit beta-1 (AKIN beta1) (AKINB1) (KINB1) [Arabidopsis Thaliana] (GB:Q84VQ1;GB:Q9SCY6); similar to 5'-AMP-activated protein kinase beta-2 subunit, putative [Arabidopsis thaliana] (TAIR:AT4G16360.1); similar to AKIN beta1 [Medicago truncatula] (GB:AAO61676.1); contains InterPro domain 5-AMP-activated protein kinase, beta subunit, complex-interacting region; (InterPro:IPR006828), AMP-activated protein kinase; similar to 5'-AMP-activated protein kinase beta-2 subunit, putative [Arabidopsis thaliana] (TAIR:AT4G16360.1); similar to AKIN beta1 [Medicago truncatula] (GB:AAO61676.1); contains InterPro domain 5-AMP-activated protein kinase, beta subunit, complex-interacting region; (InterPro:IPR006828) |
| 992 | 262488\_at | AT1G21830 |  |  | -0.3 | -0.1 | 1.8 | -0.8 | -0.1 | -1.1 | -0.2 | -0.3 | 1 | -1.1 | -0.3 | 0.8 | 1.4 | -0.3 | 0.6 | -1.4 | -2.1 | -0.8 | 0.4 | 0.3 | 0.3 | 0.2 | 0.6 | -0.5 | 0.4 | 0.4 | 0 | -0.6 | similar to unknown protein [Arabidopsis thaliana] (TAIR:AT1G44608.1); similar to Os04g0690500 [Oryza sativa (japonica cultivar-group)] (GB:NP\_001054352.1); similar to CAA30371.1 protein [Oryza sativa] (GB:CAB53474.1); similar to H0814G11.20 [Oryza sativa (indica cultivar-group)] (GB:CAJ86353.1) |
| 991 | 262456\_at | AT1G11260 | ATSTP1, ATSTP1, STP1, STP1 | 9 | -1.2 | 0.6 | 1.4 | 0 | 0.2 | -1 | 0.3 | -0.1 | 0.3 | -2.2 | 0.3 | 0.7 | 1 | -0.1 | 0.8 | -2.2 | -2.1 | -1.5 | 0.6 | -0.2 | -0.7 | 0.6 | 0.8 | -0.3 | 0.4 | -0.2 | -1.8 | -0.6 | Encodes a H+/hexose cotransporter., Encodes a H+/hexose cotransporter., Encodes a H+/hexose cotransporter., Encodes a H+/hexose cotransporter. |
| 990 | 253874\_at | AT4G27450 |  | 1 | -0.1 | 0.1 | -0.5 | -0.3 | 0.2 | -1.1 | 0.7 | 0 | 0.8 | -1.9 | 0.1 | 4.3 | 4.5 | -0.3 | 5.7 | -1.5 | -1.5 | -0.2 | 1.1 | -0.1 | -1.1 | 0.7 | 0.1 | -0.4 | -0.1 | 1.7 | -2 | -0.6 | similar to unknown protein [Arabidopsis thaliana] (TAIR:AT3G15450.1); similar to unknown [Glycine max] (GB:AAG00940.1); contains domain no description (G3D.3.60.20.10); contains domain N-terminal nucleophile aminohydrolases (Ntn hydrolases) (SSF56235) |
| 989 | 254828\_at | AT4G12550 | AIR1 | 2 | 0.1 | 0 | 0.1 | 0.2 | 0.3 | -0.1 | 0.4 | 0.4 | 0.1 | 0.1 | 0 | 0.7 | 0.7 | -0.3 | 0.8 | 0 | -1.6 | 0.3 | 0.5 | 0.3 | 0.3 | 0 | 0.1 | 0.4 | -2.2 | -2.2 | -1.2 | -0.2 | isolated from differential screening of a cDNA library from auxin-treated root culture. encodes a protein that is related to a large family of proteins that consist of a proline-rich or glycine-rich N-terminus and a hydrophobic, possibly membrane spanning C-terminus. |
| 988 | 252367\_at | AT3G48360 | ATBT2, BT2 | 3 | -0.8 | -0.3 | -2.1 | 0.2 | -0.1 | -2.2 | 0.6 | -0.3 | 0 | -1.8 | -0.1 | 3.7 | 1 | -0.2 | 4 | 0.2 | -1.3 | 1.9 | 2.7 | 0.4 | 0.6 | 0.4 | 0.4 | 0.6 | 0.1 | 0.1 | -0.8 | -0.7 | encodes a protein (BT2) that is an essential component of the TAC1-mediated telomerase activation pathway, encodes a protein (BT2) that is an essential component of the TAC1-mediated telomerase activation pathway |
| 987 | 258434\_at | AT3G16770 | ATEBP, ERF72, RAP2.3 | 11 | -0.3 | 0.1 | -0.2 | -0.5 | -0.1 | -0.2 | 0.4 | 0 | -0.9 | -0.2 | -0.5 | 0.6 | 0.5 | -0.1 | 0.6 | -1.5 | -1.5 | 0 | 0.3 | 1 | -0.1 | 0.5 | 0.1 | -0.4 | -0.5 | 0.2 | -0.6 | -0.3 | Encodes a member of the ERF (ethylene response factor) subfamily B-2 of the plant specific ERF/AP2 transcription factor family (RAP2.3). The protein contains one AP2 domain. There are 5 members in this subfamily including RAP2.2 AND RAP2.12.It is localized to the nucleus and acts as a transcriptional activator through the GCC-box. It has been identified as a suppressor of Bax-induced cell death by functional screening in yeast and can also suppress Bax-induced cell death in tobacco plants. Overexpression of this gene in tobacco BY-2 cells confers resistance to H2O2 and heat stresses. Overexpression in Arabidopsis causes upregulation of PDF1.2 and GST6. It is part of the ethylene signaling pathway and is predicted to act downstream of EIN2 and CTR1, but not under EIN3., Encodes a member of the ERF (ethylene response factor) subfamily B-2 of the plant specific ERF/AP2 transcription factor family (RAP2.3). The protein contains one AP2 domain. There are 5 members in this subfamily including RAP2.2 AND RAP2.12.It is localized to the nucleus and acts as a transcriptional activator through the GCC-box. It has been identified as a suppressor of Bax-induced cell death by functional screening in yeast and can also suppress Bax-induced cell death in tobacco plants. Overexpression of this gene in tobacco BY-2 cells confers resistance to H2O2 and heat stresses. Overexpression in Arabidopsis causes upregulation of PDF1.2 and GST6. It is part of the ethylene signaling pathway and is predicted to act downstream of EIN2 and CTR1, but not under EIN3., Encodes a member of the ERF (ethylene response factor) subfamily B-2 of the plant specific ERF/AP2 transcription factor family (RAP2.3). The protein contains one AP2 domain. There are 5 members in this subfamily including RAP2.2 AND RAP2.12.It is localized to the nucleus and acts as a transcriptional activator through the GCC-box. It has been identified as a suppressor of Bax-induced cell death by functional screening in yeast and can also suppress Bax-induced cell death in tobacco plants. Overexpression of this gene in tobacco BY-2 cells confers resistance to H2O2 and heat stresses. Overexpression in Arabidopsis causes upregulation of PDF1.2 and GST6. It is part of the ethylene signaling pathway and is predicted to act downstream of EIN2 and CTR1, but not under EIN3. |
| 986 | 261751\_at | AT1G76080 | ATCDSP32, CDSP32 | 4 | 0.1 | -0.2 | -0.2 | -0.1 | 0.1 | -0.3 | 0.1 | -0.2 | -0.3 | -0.3 | -0.4 | 0.1 | 0 | -0.3 | 0.3 | -0.5 | -2 | -0.3 | -0.2 | -0.2 | -0.2 | -0.1 | -0.3 | -0.3 | -0.1 | -0.5 | 0 | -0.2 | ATCDSP32/CDSP32 (CHLOROPLASTIC DROUGHT-INDUCED STRESS PROTEIN OF 32 KD); thiol-disulfide exchange intermediate; similar to ATTRX4 (thioredoxin H-type 4), thiol-disulfide exchange intermediate [Arabidopsis thaliana] (TAIR:AT1G19730.1); similar to CDSP32 protein (Chloroplast Drought-induced Stress Protein of 32kDa) [Solanum tuberosum] (GB:CAA71103.1); contains InterPro domain Thioredoxin-like fold; (InterPro:IPR012336); contains InterPro domain Thioredoxin-related; (InterPro:IPR006662); contains InterPro domain Thioredoxin domain 2; (InterPro:IPR006663); contains InterPro domain Thioredoxin domain; (InterPro:IPR013766); contains InterPro domain Thioredoxin fold; (InterPro:IPR012335), ATCDSP32/CDSP32 (CHLOROPLASTIC DROUGHT-INDUCED STRESS PROTEIN OF 32 KD); thiol-disulfide exchange intermediate; similar to ATTRX4 (thioredoxin H-type 4), thiol-disulfide exchange intermediate [Arabidopsis thaliana] (TAIR:AT1G19730.1); similar to CDSP32 protein (Chloroplast Drought-induced Stress Protein of 32kDa) [Solanum tuberosum] (GB:CAA71103.1); contains InterPro domain Thioredoxin-like fold; (InterPro:IPR012336); contains InterPro domain Thioredoxin-related; (InterPro:IPR006662); contains InterPro domain Thioredoxin domain 2; (InterPro:IPR006663); contains InterPro domain Thioredoxin domain; (InterPro:IPR013766); contains InterPro domain Thioredoxin fold; (InterPro:IPR012335) |
| 985 | 251918\_at | AT3G54040 |  |  | -0.6 | 0 | -0.1 | 0.2 | 3.5 | -1.2 | -0.2 | 0 | -0.2 | 3.1 | 0 | 0.5 | 0.3 | -0.2 | 0.6 | 0 | 2.1 | 0.3 | 0 | 0.2 | 1.5 | 0.4 | 0.1 | -0.7 | 0.4 | -0.3 | -2 | -0.1 | photoassimilate-responsive protein-related; similar to photoassimilate-responsive protein, putative [Arabidopsis thaliana] (TAIR:AT5G52390.1); similar to NtEIG-E80 [Nicotiana tabacum] (GB:BAB16427.1); contains InterPro domain PAR1; (InterPro:IPR009489) |
| 984 | 247297\_at | AT5G64100 |  |  | 0.3 | 0.1 | -0.2 | -0.3 | 1.3 | -1.9 | 0.2 | -0.2 | -0.3 | -0.4 | 0.1 | 1.4 | 8.7 | 0 | 2.8 | -0.1 | 1.1 | 0.7 | 0.3 | -0.2 | 2.1 | 0.9 | 1.3 | -1.6 | -1.1 | -0.4 | -1.4 | -0.9 | peroxidase, putative; Identical to Peroxidase 69 precursor (EC 1.11.1.7) (Atperox P69) (ATP3a) (PER69) [Arabidopsis Thaliana] (GB:Q96511;GB:Q8RWL8); similar to peroxidase, putative [Arabidopsis thaliana] (TAIR:AT5G64110.1); similar to peroxidase precursor [Solanum lycopersicum] (GB:CAA64413.1); similar to cationic peroxidase [Solanum lycopersicum] (GB:CAG25463.1); contains InterPro domain Haem peroxidase; (InterPro:IPR010255); contains InterPro domain Haem peroxidase, plant/fungal/bacterial; (InterPro:IPR002016); contains InterPro domain Plant peroxidase; (InterPro:IPR000823) |
| 983 | 254331\_s\_at | AT4G22690, AT4G22710 AT4G22690, AT4G22710 | CYP706A1, CYP706A2 | 2 | -0.9 | -0.1 | 0 | 0.1 | -0.2 | -2.4 | -0.1 | 0.2 | 0.1 | 1.2 | 0 | -0.5 | -0.2 | -0.5 | -2.4 | 0.1 | -1.4 | 0.1 | 0.8 | 0.7 | 0.9 | 0.2 | -0.5 | -0.2 | 1.7 | 1.3 | 1 | 0.1 | member of CYP706A, member of CYP706A |
| 982 | 245765\_at | AT1G33600 |  | 1 | -0.2 | -0.2 | -0.5 | 0.1 | -0.6 | -2.4 | 0.2 | 0.1 | -0.2 | 1.2 | -0.2 | -0.3 | -0.4 | -1.2 | -1.6 | 1 | -1.4 | 0.7 | -0.3 | 0.3 | 0.8 | 0.2 | 0.3 | 0.1 | 2.1 | 1.7 | 1.8 | 0.6 | leucine-rich repeat family protein; similar to disease resistance protein-related / LRR protein-related [Arabidopsis thaliana] (TAIR:AT2G26380.1); similar to disease resistance protein-related / LRR protein-related [Arabidopsis thaliana] (TAIR:AT1G33590.1); similar to H0402C08.6 [Oryza sativa (indica cultivar-group)] (GB:CAJ86230.1); contains InterPro domain Leucine-rich repeat; (InterPro:IPR001611); contains InterPro domain Leucine-rich repeat, plant specific; (InterPro:IPR007090); contains InterPro domain Leucine rich repeat, N-terminal; (InterPro:IPR013210) |
| 981 | 247693\_at | AT5G59730 | ATEXO70H7 | 2 | -1 | 0.3 | -0.3 | 0.2 | -0.1 | -0.6 | 0.2 | 0.2 | -0.6 | 0.1 | 0.7 | -0.7 | -1.1 | 0 | -0.7 | 0.2 | -2 | 1.4 | -0.1 | 0.3 | 0.9 | 0.1 | -0.5 | 1.2 | 2.3 | 1.6 | 1.3 | -0.1 | A member of EXO70 gene family, putative exocyst subunits, conserved in land plants. Arabidopsis thaliana contains 23 putative EXO70 genes, which can be classified into eight clusters on the phylogenetic tree. |
| 980 | 246270\_at | AT4G36500 |  |  | -1 | 0.6 | 0.2 | 0.8 | -0.1 | -0.2 | -0.5 | 0.3 | -0.2 | 0.9 | 0.2 | -0.2 | -0.3 | 0.3 | -0.7 | 0.6 | -1.2 | 0.7 | 0.5 | 0.9 | 1.6 | -0.1 | -0.4 | 2.7 | 3.1 | 2.2 | 2.4 | 0.8 | similar to unknown protein [Arabidopsis thaliana] (TAIR:AT2G18210.1); similar to hypothetical protein [Thellun (GB:ABB45855.1) |
| 979 | 252679\_at | AT3G44260 |  |  | -1.8 | -0.2 | 1.2 | -0.7 | -0.5 | 0.2 | 0.2 | 0.7 | -1.1 | 1.9 | 0.8 | -0.1 | 0.1 | 1.3 | 0.3 | 1.2 | -1.8 | 0.2 | 0 | 2.1 | 1.4 | 1.1 | 1 | 1.4 | 1.7 | 3 | 1.1 | 0.1 | CCR4-NOT transcription complex protein, putative; similar to CCR4-NOT transcription complex protein, putative [Arabidopsis thaliana] (TAIR:AT5G22250.1); similar to CCR4 associated factor 1-related protein [Capsicum annuum] (GB:ABG66307.1); contains InterPro domain Ribonuclease CAF1; (InterPro:IPR006941); contains InterPro domain Polynucleotidyl transferase, Ribonuclease H fold; (InterPro:IPR012337) |
| 978 | 261453\_at | AT1G21130 |  | 2 | -1.9 | 0.2 | 0.4 | 0.6 | -0.1 | 1.3 | -0.3 | 0.1 | 0.2 | 2.7 | -0.3 | -2.4 | -1.1 | -1.1 | -4.5 | -0.3 | -1.7 | -1 | 1.2 | 0.4 | 1.4 | 0.4 | -0.4 | -1.2 | 3.8 | 0.7 | 0.7 | -0.8 | O-methyltransferase, putative; similar to O-methyltransferase, putative [Arabidopsis thaliana] (TAIR:AT1G21120.1); similar to O-methyltransferase, putative [Arabidopsis thaliana] (TAIR:AT1G21100.1); similar to O-methyltransferase, putative [Arabidopsis thaliana] (TAIR:AT1G21110.1); similar to phloroglucinol O-methyltransferase [Rosa chinensis var. spontanea] (GB:BAD18975.1); contains InterPro domain SAM (and some other nucleotide) binding motif; (InterPro:IPR000051); contains InterPro domain O-methyltransferase, family 2; (InterPro:IPR001077); contains InterPro domain Dimerisation; (InterPro:IPR012967); contains InterPro domain Generic methyltransferase; (InterPro:IPR001601); contains InterPro domain Winged helix repressor DNA-binding; (InterPro:IPR011991), O-methyltransferase, putative; similar to O-methyltransferase, putative [Arabidopsis thaliana] (TAIR:AT1G21120.1); similar to phloroglucinol O-methyltransferase [Rosa chinensis var. spontanea] (GB:BAD18975.1); contains InterPro domain O-methyltransferase, family 2; (InterPro:IPR001077); contains InterPro domain Dimerisation; (InterPro:IPR012967) |
| 977 | 256221\_at | AT1G56300 |  | 1 | -0.8 | 0.3 | 0.6 | 0.3 | 0.1 | 2 | -0.2 | -0.1 | -0.6 | 3.8 | 0.1 | 0.1 | -0.2 | -0.5 | 0.1 | 0.8 | -1 | -0.4 | 0.8 | -0.6 | 1.6 | 0.3 | 0.3 | 0.5 | 0.9 | 0.5 | 0.7 | 0.2 | DNAJ heat shock N-terminal domain-containing protein; similar to DNAJ heat shock N-terminal domain-containing protein [Arabidopsis thaliana] (TAIR:AT3G14200.1); similar to DnaJ-like protein [Glycine max] (GB:AAM23263.1); contains InterPro domain Heat shock protein DnaJ, N-terminal; (InterPro:IPR001623) |
| 976 | 249741\_at | AT5G24470 | APRR5, PRR5 | 22 | -1.2 | -0.7 | -0.5 | 0.5 | -0.3 | 0.5 | -0.7 | -0.8 | -0.7 | 0.2 | 0.5 | -0.9 | -1.8 | 0.3 | -1.1 | 0.7 | -2 | -0.5 | 0 | -0.1 | -0.1 | 0.2 | 0.2 | -0.4 | 0.1 | -0.4 | 0.1 | -0.8 | Encodes a pseudo-response regulator whose mutation affects various circadian-associated biological events such as flowering time in the long-day photoperiod conditions, red light sensitivity of seedlings during early photomorphogenesis, and the period of free-running rhythms of certain clock-controlled genes including CCA1 and APRR1/TOC1 in constant white light., Encodes a pseudo-response regulator whose mutation affects various circadian-associated biological events such as flowering time in the long-day photoperiod conditions, red light sensitivity of seedlings during early photomorphogenesis, and the period of free-running rhythms of certain clock-controlled genes including CCA1 and APRR1/TOC1 in constant white light. |
| 975 | 259992\_at | AT1G67970 | AT-HSFA8, HSFA8 | 1 | -0.7 | -0.6 | 0.2 | -0.3 | 0.4 | -0.9 | -0.1 | 0.6 | 1 | 3.3 | 0.8 | 0.2 | 0.7 | 0.4 | 0 | 0.6 | -1.2 | 0.3 | 4 | 2.7 | 2.1 | 0.1 | 1.2 | 0.3 | 2.3 | 1.7 | 1.8 | 1.3 | member of Heat Stress Transcription Factor (Hsf) family, member of Heat Stress Transcription Factor (Hsf) family |
| 974 | 249128\_at | AT5G43440 |  | 1 | -1 | -0.2 | -1.9 | -0.2 | 0 | -0.6 | 0.5 | 0.1 | -0.4 | 2.4 | 0.4 | -0.7 | 0.1 | -0.2 | -0.4 | -0.9 | -1.4 | -0.7 | 1.3 | 2.2 | -0.3 | -0.3 | 0.3 | -1.8 | 0.7 | 0 | 0.6 | 0.8 | encodes a protein whose sequence is similar to ACC oxidase |
| 973 | 261143\_at | AT1G19770 | ATPUP14 | 1 | -1.6 | -0.1 | 0.1 | 0.2 | -0.2 | -1.2 | 0.5 | 0 | -0.9 | -0.1 | 0.1 | -0.8 | -0.8 | 0 | -1.1 | -0.3 | -1.5 | -0.4 | 0.3 | 0.3 | 0.4 | 0.1 | 0.1 | -0.7 | 0.6 | 0.6 | 0.5 | 0.1 | Member of a family of proteins related to PUP1, a purine transporter. May be involved in the transport of purine and purine derivatives such as cytokinins, across the plasma membrane. |
| 972 | 248870\_at | AT5G46710 |  |  | -1.2 | -0.4 | 0.1 | 1.5 | 0.6 | -1 | 0.4 | 0.2 | -0.7 | -0.6 | -0.1 | -1.2 | -0.9 | -0.5 | -1.4 | -0.6 | -2.7 | -1.6 | 1.2 | 2.7 | 4.2 | 0.5 | 0.6 | -0.8 | 0.5 | 1 | 1.8 | 0.9 | zinc-binding family protein; similar to zinc-binding family protein [Arabidopsis thaliana] (TAIR:AT4G17900.1); similar to putative zinc-binding protein [Platanus x acerifolia] (GB:CAL25342.1); contains InterPro domain Protein of unknown function DUF597; (InterPro:IPR006734) |
| 971 | 257766\_at | AT3G23030 | IAA2 | 9 | -1.6 | 0.4 | -1.1 | -2.5 | -0.1 | -0.3 | 0.1 | -0.1 | 0.4 | 0.5 | -0.3 | -1.5 | -1.5 | -0.1 | -0.1 | -0.6 | -0.9 | -1.6 | 0.6 | 0 | 0 | 0.9 | 3.7 | 0.7 | 0.8 | 1.3 | 1.2 | 0 | auxin inducible gene expressed in the nucleus |
| 970 | 254120\_at | AT4G24570 |  | 1 | -2.8 | 0.5 | 1.4 | 0.4 | 0.1 | -1.2 | -0.6 | 0.6 | -0.2 | 1.1 | 1.1 | 1 | 1.2 | 1.4 | -1 | 1.2 | -3 | 0.2 | 0.2 | 2.4 | 1.7 | -0.1 | 0.2 | -1.1 | 4.4 | 3.7 | 1.8 | 0.2 | mitochondrial substrate carrier family protein; similar to mitochondrial substrate carrier family protein [Arabidopsis thaliana] (TAIR:AT2G22500.1); similar to putative mitochondrial dicarboxylate carrier protein [Trifolium pratense] (GB:BAE71294.1); contains InterPro domain Mitochondrial carrier protein; (InterPro:IPR002067); contains InterPro domain Mitochondrial substrate carrier; (InterPro:IPR001993) |
| 969 | 253915\_at | AT4G27280 |  |  | -2.5 | -0.2 | 1.2 | 0.6 | 0.2 | -0.6 | -0.5 | 0.4 | 0.5 | 1.4 | 0.5 | 1.3 | 2.1 | 2.4 | 0.9 | -0.1 | -2.7 | -0.4 | 1.1 | 2.6 | 2 | 0 | 2.1 | -1.2 | 2.5 | 5 | 2.5 | 0.2 | calcium-binding EF hand family protein; similar to PBP1 (PINOID-BINDING PROTEIN 1), calcium ion binding [Arabidopsis thaliana] (TAIR:AT5G54490.1); similar to Avr9/Cf-9 rapidly elicited protein 20 [Nicotiana tabacum] (GB:AAV92890.1); contains InterPro domain EF-Hand type; (InterPro:IPR011992); contains InterPro domain Calcium-binding EF-hand; (InterPro:IPR002048) |
| 968 | 247543\_at | AT5G61600 |  | 3 | -1.8 | 0.3 | 1.3 | 0.7 | -1.1 | 0 | -0.1 | 0.2 | 0 | 3.1 | 0.3 | 0 | 0.7 | 1.8 | -0.3 | -0.3 | -2.2 | -2.6 | 1.7 | 4 | 2.4 | 1 | -1 | 0 | 1.6 | 2.1 | 0.7 | 0.4 | encodes a member of the ERF (ethylene response factor) subfamily B-3 of ERF/AP2 transcription factor family. The protein contains one AP2 domain. There are 18 members in this subfamily including ATERF-1, ATERF-2, AND ATERF-5. |
| 967 | 245866\_s\_at | AT1G57980, AT1G57990 AT1G57980, AT1G57990 | ATPUP18 | 1 | -4.3 | 3.1 | 2.8 | 0.2 | -0.1 | 0.8 | -0.1 | 0.5 | -0.1 | 0.8 | 0.6 | 2.1 | 1.1 | 3 | 1 | 0.6 | -2 | -0.2 | 1.4 | -0.2 | 2.6 | 1.2 | 1.4 | 0 | 2.3 | 3.1 | 1.4 | -0.7 | purine permease-related; similar to ATPUP18 (Arabidopsis thaliana purine permease 18), purine transporter [Arabidopsis thaliana] (TAIR:AT1G57990.1); similar to putative purine permease [Oryza sativa (japonica cultivar-group)] (GB:BAD07671.1); contains InterPro domain Protein of unknown function DUF250; (InterPro:IPR004853), Member of a family of proteins related to PUP1, a purine transporter. May be involved in the transport of purine and purine derivatives such as cytokinins, across the plasma membrane. |
| 966 | 263931\_at | AT2G36220 |  |  | -1.8 | 0.1 | 2 | -0.7 | -0.4 | 1 | -0.3 | 0.4 | -0.2 | 4.6 | 0.5 | 0.7 | 1 | 1.8 | 1.7 | 2.3 | 0.9 | -0.1 | 0.6 | 0.8 | 1.6 | 0.7 | 2.5 | -0.4 | 1.8 | 3.4 | 2.7 | 0.2 | similar to unknown protein [Arabidopsis thaliana] (TAIR:AT3G52710.1); similar to Os11g0153300 [Oryza sativa (japonica cultivar-group)] (GB:NP\_001065777.1); similar to calcium/calmodulin protein kinase 1 [Nicotiana tabacum] (GB:AAN71903.1) |
| 965 | 246289\_at | AT3G56880 |  |  | -1.8 | 0.5 | 1.1 | 0.3 | -0.1 | 2.7 | -0.1 | 0 | -1.1 | 2.5 | 0 | 0.1 | 1.3 | 2 | 1.3 | 0 | -1.5 | 0 | -0.1 | 1.1 | 0.6 | -0.2 | 0.7 | -0.8 | 1.2 | 1.9 | 1.6 | 0.8 | VQ motif-containing protein; similar to ATCAMBP25 (ARABIDOPSIS THALIANA CALMODULIN (CAM)-BINDING PROTEIN OF 25 KDA), calmodulin binding [Arabidopsis thaliana] (TAIR:AT2G41010.1); similar to VQ [Medicago truncatula] (GB:ABD32874.1); contains InterPro domain VQ; (InterPro:IPR008889) |
| 964 | 267357\_at | AT2G40000 | ATHSPRO2, HSPRO2 | 2 | -3.3 | 0.2 | 0.6 | -0.3 | 0.1 | 2 | 0.2 | 0.4 | 0.4 | 0.1 | 0.6 | 2.9 | 2.3 | 2.8 | 3.5 | 0.5 | -1.9 | 0.4 | 0.5 | 0.8 | 0.5 | 0.6 | 2.1 | -0.1 | 2.4 | 4.2 | 1.6 | 0.8 | similar to unknown protein [Arabidopsis thaliana] (TAIR:AT3G55840.1); similar to putative Hs1pro-1-like receptor [Glycine max] (GB:AAG44839.1); contains InterPro domain Hs1pro-1, C-terminal; (InterPro:IPR009743); contains InterPro domain Hs1pro-1, N-terminal; (InterPro:IPR009869), similar to unknown protein [Arabidopsis thaliana] (TAIR:AT3G55840.1); similar to putative Hs1pro-1-like receptor [Glycine max] (GB:AAG44839.1); contains InterPro domain Hs1pro-1, C-terminal; (InterPro:IPR009743); contains InterPro domain Hs1pro-1, N-terminal; (InterPro:IPR009869) |
| 963 | 254926\_at | AT4G11280 | ACS6 | 13 | -0.4 | 0.9 | 2.3 | 0.2 | 0 | 2.7 | -1.1 | 0.4 | 0.9 | 2.2 | 0.6 | 3.3 | 3.1 | 1.9 | 3.6 | 1.6 | -1.8 | 2.4 | 0.5 | 2.3 | 2.2 | 0.8 | 3.1 | -0.1 | 5.7 | 3.6 | 3.9 | 1.4 | encodes a a member of the 1-aminocyclopropane-1-carboxylate (ACC) synthase (S-adenosyl-L-methionine methylthioadenosine-lyase, EC 4.4.1.14) gene family |
| 962 | 262382\_at | AT1G72920 |  |  | -0.9 | 0.8 | 0.6 | 2.2 | 0.9 | -2.3 | 0 | 0.4 | 0.7 | -0.2 | 0.3 | 1 | 1.2 | 0.1 | 0.7 | 0.6 | -2.7 | 0.5 | 1.5 | 1.9 | 1.2 | 1.2 | -0.2 | 3.2 | 5.6 | 3.8 | 3.8 | 1.6 | disease resistance protein (TIR-NBS class), putative; similar to disease resistance protein (TIR-NBS class), putative [Arabidopsis thaliana] (TAIR:AT1G72940.1); similar to TMV resistance protein N (GB:Q40392); contains InterPro domain Toll-Interleukin receptor; (InterPro:IPR000157) |
| 961 | 252483\_at | AT3G46600 |  | 3 | -0.9 | 0.1 | 0.9 | 0.5 | 0.2 | -0.7 | 0 | 0.1 | 0.3 | -0.2 | 0.2 | -0.3 | 0 | 0.6 | 0.7 | -0.5 | -1.4 | -0.5 | 0.9 | 1.5 | 0.6 | 0.3 | 0.2 | 0.2 | 2.4 | 1.5 | 1.6 | 1.5 | scarecrow transcription factor family protein; similar to scarecrow-like transcription factor 11 (SCL11) [Arabidopsis thaliana] (TAIR:AT5G59450.1); similar to GRAS1 [Nicotiana tabacum] (GB:ABE02823.1); contains InterPro domain GRAS transcription factor; (InterPro:IPR005202) |
| 960 | 266617\_at | AT2G29670 |  |  | -1.5 | -0.1 | -0.3 | 0.9 | -0.1 | -0.6 | -0.3 | 0.3 | -0.3 | -1.3 | 0.5 | -0.2 | 0.3 | 1.3 | 0.2 | -1.3 | -1.9 | -0.3 | -0.1 | 0.6 | -0.3 | -0.2 | -0.4 | 1.4 | 1.2 | -0.5 | 0.4 | 0.2 | binding; similar to binding [Arabidopsis thaliana] (TAIR:AT1G07280.2); similar to Os05g0447700 [Oryza sativa (japonica cultivar-group)] (GB:NP\_001055695.1); similar to Os01g0855200 [Oryza sativa (japonica cultivar-group)] (GB:NP\_001044840.1); similar to putative peroxidase [Oryza sativa (japonica cultivar-group)] (GB:AAM93690.1); contains InterPro domain Tetratricopeptide region; (InterPro:IPR013026); contains InterPro domain Tetratricopeptide-like helical; (InterPro:IPR011990) |
| 959 | 261193\_at | AT1G32920 |  |  | -1.4 | 0.4 | 1.5 | 0.3 | 0.4 | -0.4 | -0.4 | 0.4 | -0.1 | 0.8 | 0.3 | 1.9 | 0.9 | 1.3 | 2.3 | 0.8 | -1 | 1.5 | 0.3 | 1.9 | 2.2 | -0.1 | 0.8 | 1 | 1.5 | 2.4 | 2.3 | 0.1 | similar to unknown protein [Arabidopsis thaliana] (TAIR:AT1G32928.1) |
| 958 | 245041\_at | AT2G26530 | AR781 | 7 | -0.9 | 2.1 | 1.5 | -1.3 | 0.9 | -2.1 | 1.1 | 0.1 | -0.4 | 2.8 | 0.4 | 2.2 | 1.4 | 0.2 | 2.8 | 1.3 | -2.3 | 1.8 | 2.8 | 4 | 5.2 | 0.6 | 1.5 | 4.8 | 4.6 | 4.5 | 3.4 | 0.4 | unknown function |
| 957 | 261892\_at | AT1G80840 | ATWRKY40, WRKY40 | 8 | -2.7 | 0.4 | -1.1 | -0.3 | 0.5 | 0 | 0.6 | 0.7 | 1.9 | 4.6 | 0.8 | 1 | 1.6 | 2.7 | 3.7 | 1.9 | -2.5 | 4.7 | 1.2 | 2.5 | 4 | 0.4 | 2.6 | 4.3 | 5.3 | 7.4 | 5.5 | 2.3 | Pathogen-induced transcription factor. Binds W-box sequences in vitro. Forms protein complexes with itself and with WRKY40 and WRKY60. Coexpression with WRKY18 or WRKY60 made plants more susceptible to both P. syringae and B. cinerea. WRKY18, WRKY40, and WRKY60 have partially redundant roles in response to the hemibiotrophic bacterial pathogen Pseudomonas syringae and the necrotrophic fungal pathogen Botrytis cinerea, with WRKY18 playing a more important role than the other two., Pathogen-induced transcription factor. Binds W-box sequences in vitro. Forms protein complexes with itself and with WRKY40 and WRKY60. Coexpression with WRKY18 or WRKY60 made plants more susceptible to both P. syringae and B. cinerea. WRKY18, WRKY40, and WRKY60 have partially redundant roles in response to the hemibiotrophic bacterial pathogen Pseudomonas syringae and the necrotrophic fungal pathogen Botrytis cinerea, with WRKY18 playing a more important role than the other two. |
| 956 | 251745\_at | AT3G55980 | ATSZF1, SZF1 | 1 | -3 | 0.5 | 0.7 | 0.6 | 0.7 | -0.6 | 0.4 | 0.6 | 1.2 | 3 | 0.8 | 0.6 | 1.5 | 1.4 | 0.7 | 0.6 | -1.9 | 2 | 1.4 | 2.7 | 2.8 | 0.2 | 0.6 | 1.1 | 3.4 | 3.9 | 2.8 | 1.1 | zinc finger (CCCH-type) family protein; similar to CZF1/ZFAR1 [Arabidopsis thaliana] (TAIR:AT2G40140.2); similar to Cys-3-His zinc finger protein [Capsicum annuum] (GB:ABI30334.1); contains InterPro domain Ankyrin; (InterPro:IPR002110); contains InterPro domain Zinc finger, CCCH-type; (InterPro:IPR000571), zinc finger (CCCH-type) family protein; similar to CZF1/ZFAR1 [Arabidopsis thaliana] (TAIR:AT2G40140.2); similar to Cys-3-His zinc finger protein [Capsicum annuum] (GB:ABI30334.1); contains InterPro domain Ankyrin; (InterPro:IPR002110); contains InterPro domain Zinc finger, CCCH-type; (InterPro:IPR000571) |
| 955 | 262383\_at | AT1G72940 |  |  | -1.9 | 0.7 | -0.7 | 0 | 0.5 | -1.3 | -0.1 | 0.5 | 0.7 | 0.3 | 0.5 | 0.4 | 0 | 1.4 | 2.1 | 0.2 | -1.4 | 1.8 | 0.5 | 1.2 | 0.7 | 1.9 | 0.4 | 3.5 | 2.5 | 2.4 | 1.4 | 0 | disease resistance protein (TIR-NBS class), putative; similar to disease resistance protein (TIR-NBS class), putative [Arabidopsis thaliana] (TAIR:AT1G72950.1); similar to resistance gene analog PU3 [Helianthus annuus] (GB:AAL07535.1); contains InterPro domain Disease resistance protein; (InterPro:IPR000767); contains InterPro domain Toll-Interleukin receptor; (InterPro:IPR000157) |
| 954 | 265417\_at | AT2G20920 |  |  | -0.8 | -0.1 | -0.4 | 0.2 | -0.2 | -0.1 | 0.4 | -0.1 | -0.7 | 0.3 | 0 | 0.3 | -0.3 | 1.5 | 1.1 | 0.4 | -1.2 | 0.3 | -0.4 | 0.3 | 0 | -0.2 | -0.2 | -0.2 | 0.7 | 0 | 0.4 | 0.1 | similar to CDF1 (CELL GROWTH DEFECT FACTOR 1), heat shock protein binding [Arabidopsis thaliana] (TAIR:AT5G23040.2); similar to expressed protein [Oryza sativa (japonica cultivar-group)] (GB:ABF93866.1) |
| 953 | 260012\_at | AT1G67865 |  |  | -0.2 | -2 | -3 | 0.7 | 0.2 | 0 | 0.6 | 0.2 | -0.2 | 0 | 0.3 | 0.5 | 0.5 | 0.6 | 1.5 | 0 | -3.3 | -0.9 | 0 | -0.2 | -0.9 | -2.4 | -1.4 | 0.3 | 0.6 | 0.6 | 0.6 | 1 | similar to unknown protein [Arabidopsis thaliana] (TAIR:AT1G67860.1) |
| 952 | 246962\_s\_at | AT5G24800 | AtbZIP9, BZO2H2 | 4 | -1 | 0.1 | -0.7 | -0.2 | 0.2 | 2.2 | 0.8 | -0.2 | -1.1 | -0.8 | -0.4 | 2.1 | -0.2 | -0.5 | 3.7 | -0.5 | -1 | 1.2 | 0 | -0.6 | -0.5 | -0.3 | -0.2 | 0.7 | 1.3 | 0.8 | 1.2 | 1.2 | Encodes bZIP protein BZO2H2., Encodes bZIP protein BZO2H2. |
| 951 | 247025\_at | AT5G67030 | ABA1, ATABA1, ATZEP, IBS3, LOS6, NPQ2, ZEP | 58 | -1.7 | -0.1 | -0.1 | 0.1 | -0.1 | 1.9 | 0.2 | -0.2 | -0.5 | 0.3 | 0.1 | -0.4 | -0.1 | 0.1 | 0.6 | -1.1 | -1.4 | -0.7 | -0.3 | 0 | -0.1 | 0.1 | -0.2 | -0.5 | -0.8 | -0.8 | 0.1 | -0.4 | Encodes a single copy zeaxanthin epoxidase gene that functions in first step of the biosynthesis of the abiotic stress hormone abscisic acid (ABA). Mutants in this gene are unable to express female sterility in response to beta-aminobutyric acid, as wild type plants do., Encodes a single copy zeaxanthin epoxidase gene that functions in first step of the biosynthesis of the abiotic stress hormone abscisic acid (ABA). Mutants in this gene are unable to express female sterility in response to beta-aminobutyric acid, as wild type plants do., Encodes a single copy zeaxanthin epoxidase gene that functions in first step of the biosynthesis of the abiotic stress hormone abscisic acid (ABA). Mutants in this gene are unable to express female sterility in response to beta-aminobutyric acid, as wild type plants do., Encodes a single copy zeaxanthin epoxidase gene that functions in first step of the biosynthesis of the abiotic stress hormone abscisic acid (ABA). Mutants in this gene are unable to express female sterility in response to beta-aminobutyric acid, as wild type plants do., Encodes a single copy zeaxanthin epoxidase gene that functions in first step of the biosynthesis of the abiotic stress hormone abscisic acid (ABA). Mutants in this gene are unable to express female sterility in response to beta-aminobutyric acid, as wild type plants do., Encodes a single copy zeaxanthin epoxidase gene that functions in first step of the biosynthesis of the abiotic stress hormone abscisic acid (ABA). Mutants in this gene are unable to express female sterility in response to beta-aminobutyric acid, as wild type plants do., Encodes a single copy zeaxanthin epoxidase gene that functions in first step of the biosynthesis of the abiotic stress hormone abscisic acid (ABA). Mutants in this gene are unable to express female sterility in response to beta-aminobutyric acid, as wild type plants do. |
| 950 | 246226\_at | AT4G37200 | HCF164 | 4 | -0.4 | 0 | -0.1 | -0.1 | -0.1 | 0.7 | 0 | -0.3 | -0.6 | 0.4 | -0.2 | -0.3 | -0.3 | 0.2 | 0.6 | -0.8 | -2.4 | -0.2 | -0.2 | 0.1 | -0.1 | 0.3 | -0.1 | 0.1 | -0.3 | -0.8 | 0 | -0.2 | Encodes thioredoxin-like protein with disulfide reductase activity that is involved in the biogenesis of the plastid cytochrome b6f complex. Protein is located in the thylakoid membrane with the C-terminal hydrophilic portion, containing the thioredoxin like domain, extending into the thylakoid lumen. |
| 949 | 257611\_at | AT3G26580 |  |  | -0.5 | 0 | -0.2 | 0.1 | 0.1 | 0.9 | -0.1 | -0.1 | -0.7 | 1.7 | -0.1 | 0.1 | -0.4 | 1.3 | 0.5 | -0.1 | -1 | -0.9 | -0.1 | -0.3 | 0 | 0 | -0.2 | -0.1 | -0.2 | -0.7 | 0.1 | -0.3 | binding; similar to hypothetical protein [Spinacia oleracea] (GB:CAA74590.1); contains InterPro domain Tetratricopeptide region; (InterPro:IPR013026); contains InterPro domain Tetratricopeptide-like helical; (InterPro:IPR011990) |
| 948 | 252036\_at | AT3G52070 |  |  | -1.4 | -0.5 | -1 | 0.3 | 0.3 | 0.8 | 0.7 | -0.1 | -1.2 | 1.3 | 0 | -1.2 | -0.9 | -0.4 | -0.3 | -1.6 | -2.1 | -2.1 | -0.6 | -0.2 | -0.2 | 0 | 0 | 0.2 | 0.5 | -1.2 | -1.1 | -1.6 | similar to hypothetical protein MtrDRAFT\_AC136506g13v1 [Medicago truncatula] (GB:ABE82322.1) |
| 947 | 247637\_at | AT5G60600 | CLB4, CSB3, GCPE, HDS, ISPG | 6 | -0.4 | 0 | -0.2 | 0 | 0.1 | 0.1 | 0 | -0.1 | -0.5 | 0.5 | -0.2 | -0.3 | -0.1 | -0.2 | -0.1 | -0.5 | -1.1 | -0.7 | -0.5 | 0 | 0 | -0.1 | -0.1 | -0.1 | -0.1 | -0.8 | 0 | -0.1 | Encodes a chloroplast-localized hydroxy-2-methyl-2-(E)-butenyl 4-diphosphate (HMBPP) synthase (HDS), catalyzes the formation of HMBPP from 2-C-methyl-D-erythrytol 2,4-cyclodiphosphate (MEcPP). The HDS enzyme controls the penultimate steps of the biosynthesis of IPP and dimethylallyl diphosphate (DMAPP) via the MEP pathway and may serve as a metabolic control point for SA-mediated disease resistance. In the light, the electrons required for the reaction catalyzed by HDS are directly provided by the electron flow from photosynthesis via ferredoxin. In the dark however, the enzyme requires an electron shuttle: ferredoxin-NADP+ reductase., Encodes a chloroplast-localized hydroxy-2-methyl-2-(E)-butenyl 4-diphosphate (HMBPP) synthase (HDS), catalyzes the formation of HMBPP from 2-C-methyl-D-erythrytol 2,4-cyclodiphosphate (MEcPP). The HDS enzyme controls the penultimate steps of the biosynthesis of IPP and dimethylallyl diphosphate (DMAPP) via the MEP pathway and may serve as a metabolic control point for SA-mediated disease resistance. In the light, the electrons required for the reaction catalyzed by HDS are directly provided by the electron flow from photosynthesis via ferredoxin. In the dark however, the enzyme requires an electron shuttle: ferredoxin-NADP+ reductase., Encodes a chloroplast-localized hydroxy-2-methyl-2-(E)-butenyl 4-diphosphate (HMBPP) synthase (HDS), catalyzes the formation of HMBPP from 2-C-methyl-D-erythrytol 2,4-cyclodiphosphate (MEcPP). The HDS enzyme controls the penultimate steps of the biosynthesis of IPP and dimethylallyl diphosphate (DMAPP) via the MEP pathway and may serve as a metabolic control point for SA-mediated disease resistance. In the light, the electrons required for the reaction catalyzed by HDS are directly provided by the electron flow from photosynthesis via ferredoxin. In the dark however, the enzyme requires an electron shuttle: ferredoxin-NADP+ reductase., Encodes a chloroplast-localized hydroxy-2-methyl-2-(E)-butenyl 4-diphosphate (HMBPP) synthase (HDS), catalyzes the formation of HMBPP from 2-C-methyl-D-erythrytol 2,4-cyclodiphosphate (MEcPP). The HDS enzyme controls the penultimate steps of the biosynthesis of IPP and dimethylallyl diphosphate (DMAPP) via the MEP pathway and may serve as a metabolic control point for SA-mediated disease resistance. In the light, the electrons required for the reaction catalyzed by HDS are directly provided by the electron flow from photosynthesis via ferredoxin. In the dark however, the enzyme requires an electron shuttle: ferredoxin-NADP+ reductase., Encodes a chloroplast-localized hydroxy-2-methyl-2-(E)-butenyl 4-diphosphate (HMBPP) synthase (HDS), catalyzes the formation of HMBPP from 2-C-methyl-D-erythrytol 2,4-cyclodiphosphate (MEcPP). The HDS enzyme controls the penultimate steps of the biosynthesis of IPP and dimethylallyl diphosphate (DMAPP) via the MEP pathway and may serve as a metabolic control point for SA-mediated disease resistance. In the light, the electrons required for the reaction catalyzed by HDS are directly provided by the electron flow from photosynthesis via ferredoxin. In the dark however, the enzyme requires an electron shuttle: ferredoxin-NADP+ reductase. |
| 946 | 265481\_at | AT2G15960 |  |  | -1.2 | 0.3 | -0.4 | -0.2 | -0.2 | 1.8 | 0.5 | -0.1 | -0.9 | 3.3 | -0.1 | -0.2 | -0.4 | 0 | 0.3 | -0.1 | -0.9 | -1 | 1.2 | -0.3 | 0.6 | 0.4 | 0.3 | -0.3 | 0.3 | 0.1 | -0.3 | -0.7 | unknown protein |
| 945 | 245668\_at | AT1G28330 | DRM1, DYL1 | 4 | -1.9 | 1.4 | 1.2 | 0.5 | 0.3 | 3.1 | 2.2 | -0.2 | -1.5 | 3.1 | -0.3 | -0.8 | -1.3 | 3.9 | -0.3 | -0.4 | -0.9 | -3.7 | 1.2 | -0.8 | -0.4 | 1.6 | 1.9 | 0.2 | 1.2 | 0 | -2 | -0.6 | dormancy-associated protein (DRM1), dormancy-associated protein (DRM1) |
| 944 | 266614\_at | AT2G14910 |  |  | -0.5 | -0.1 | -0.1 | 0.2 | -0.1 | 0.3 | 0.2 | -0.2 | -0.7 | -0.2 | -0.4 | -0.6 | -0.9 | 0.1 | 0.4 | -1.1 | -1.5 | -0.2 | -0.2 | -0.1 | -0.5 | 0.1 | -0.2 | -0.1 | 0.2 | -0.3 | 0 | -0.1 | similar to unknown protein [Arabidopsis thaliana] (TAIR:AT5G14970.1); similar to seed maturation protein PM23 [Glycine max] (GB:AAF21309.1) |
| 943 | 251860\_at | AT3G54660 | ATGR2, EMB2360, GR | 6 | -0.6 | -0.1 | 0 | -0.1 | -0.1 | -0.1 | 0.2 | -0.3 | -0.4 | 0.6 | -0.3 | -0.8 | -1.1 | -0.4 | -0.3 | -0.7 | -1.5 | -0.3 | -0.4 | -0.2 | -0.3 | 0.1 | -0.2 | 0.2 | -0.3 | -0.6 | 0 | -0.3 | Encodes glutathione reductase that is most likely localized in the chloroplast., Encodes glutathione reductase that is most likely localized in the chloroplast., Encodes glutathione reductase that is most likely localized in the chloroplast. |
| 942 | 262259\_s\_at | AT1G53870, AT1G53890 AT1G53870, AT1G53890 |  |  | -1.3 | 0.5 | 0 | -0.4 | 0.1 | 0.8 | 2.1 | -0.2 | -1.5 | -0.5 | -0.2 | 0.3 | 0.7 | 1.2 | 0.9 | -0.4 | -1.8 | -0.9 | -0.5 | -0.1 | -0.1 | 2.4 | 1.4 | 4.7 | 1.3 | -1.4 | 0.8 | -0.2 | similar to unknown protein [Arabidopsis thaliana] (TAIR:AT1G53890.1); similar to Protein of unknown function DUF567 [Medicago truncatula] (GB:ABE86462.1); contains InterPro domain Protein of unknown function DUF567; (InterPro:IPR007612), similar to unknown protein [Arabidopsis thaliana] (TAIR:AT1G53890.2); similar to Protein of unknown function DUF567 [Medicago truncatula] (GB:ABE86462.1); contains InterPro domain Protein of unknown function DUF567; (InterPro:IPR007612), similar to unknown protein [Arabidopsis thaliana] (TAIR:AT1G53870.1); similar to Protein of unknown function DUF567 [Medicago truncatula] (GB:ABE86462.1); contains InterPro domain Protein of unknown function DUF567; (InterPro:IPR007612), similar to unknown protein [Arabidopsis thaliana] (TAIR:AT1G53870.2); similar to Protein of unknown function DUF567 [Medicago truncatula] (GB:ABE86462.1); contains InterPro domain Protein of unknown function DUF567; (InterPro:IPR007612) |
| 941 | 247931\_at | AT5G57040 |  | 1 | -1.2 | -0.3 | -0.3 | 0.1 | 0 | 2.4 | 0.3 | -0.1 | -0.6 | 0.7 | 0 | -0.7 | -0.7 | -0.8 | 0 | -1.8 | -1.1 | -1.7 | -0.6 | 0 | -0.2 | -0.1 | -0.2 | -0.3 | 0 | -0.7 | 0.1 | -0.3 | lactoylglutathione lyase family protein / glyoxalase I family protein; similar to lactoylglutathione lyase family protein / glyoxalase I family protein [Arabidopsis thaliana] (TAIR:AT1G80160.1); similar to Os07g0160400 [Oryza sativa (japonica cultivar-group)] (GB:NP\_001058939.1); similar to unknown protein [Oryza sativa (japonica cultivar-group)] (GB:BAC79640.1); contains InterPro domain Glyoxalase/extradiol ring-cleavage dioxygenase; (InterPro:IPR011588); contains InterPro domain Glyoxalase/bleomycin resistance protein/dioxygenase; (InterPro:IPR004360) |
| 940 | 246977\_at | AT5G24930 |  | 2 | -0.5 | -0.3 | -0.6 | 0.1 | -0.2 | 1 | 0.2 | -0.2 | -0.2 | 0.5 | -0.1 | -0.6 | -0.2 | -0.2 | 0.1 | -1.2 | -1.8 | -1.1 | -0.1 | -0.1 | -0.2 | -0.1 | -0.2 | -0.2 | -0.2 | -0.2 | 0 | -0.2 | zinc finger (B-box type) family protein; Identical to Zinc finger protein CONSTANS-LIKE 4 (COL4) [Arabidopsis Thaliana] (GB:Q940T9); similar to COL3 (CONSTANS-LIKE 3), protein binding / transcription factor/ zinc ion binding [Arabidopsis thaliana] (TAIR:AT2G24790.1); similar to Zinc finger protein CONSTANS-LIKE 4 (GB:Q940T9); contains InterPro domain Zinc finger, CONSTANS-type; (InterPro:IPR002926); contains InterPro domain CCT; (InterPro:IPR010402); contains InterPro domain Zinc finger, B-box; (InterPro:IPR000315) |
| 939 | 253751\_at | AT4G29070 |  |  | -0.4 | -0.2 | -1.1 | 0.1 | -0.3 | 0.6 | 0.1 | 0 | -0.4 | 0.6 | -0.2 | 0 | -0.2 | -0.7 | 0.4 | -0.9 | -1.1 | -0.9 | -0.3 | 0 | -0.3 | 0.1 | 0.1 | -0.5 | -0.4 | -0.2 | 0.8 | -0.1 | similar to hypothetical protein [Platanus x acerifolia] (GB:CAL07987.1); contains domain Phospholipase A2, PLA2 (SSF48619); contains domain no description (G3D.1.20.90.10) |
| 938 | 265387\_at | AT2G20670 |  | 1 | -1.5 | 0.2 | -0.7 | 0.5 | -0.1 | 1.1 | 0.3 | -0.9 | -0.7 | 0.3 | -0.4 | 1.2 | 0.1 | -2.5 | 2.5 | -3.2 | -1.9 | -4.8 | 0.5 | 1.2 | 0.5 | -0.9 | -0.3 | -1.3 | -0.2 | -0.9 | -0.3 | -0.9 | similar to unknown protein [Arabidopsis thaliana] (TAIR:AT4G32480.1); similar to Protein of unknown function DUF506, plant [Medicago truncatula] (GB:ABD32359.1); contains InterPro domain Protein of unknown function DUF506, plant; (InterPro:IPR006502) |
| 937 | 250972\_at | AT5G02840 | LCL1, LCL1 | 4 | -1.4 | -0.3 | -0.1 | 0.3 | 0.1 | 0.1 | 0.4 | 0 | -0.7 | 0 | -0.3 | 0.1 | -0.3 | -0.3 | 1.8 | -2.3 | -1.1 | -0.1 | -0.1 | -0.2 | -0.3 | -0.1 | -0.4 | -0.5 | -0.6 | -1 | -0.3 | -0.1 | DNA binding / transcription factor; similar to myb family transcription factor [Arabidopsis thaliana] (TAIR:AT3G09600.1); similar to MYB transcription factor MYB118 [Glycine max] (GB:ABH02853.1); contains InterPro domain Homeodomain-related; (InterPro:IPR012287); contains InterPro domain Homeodomain-like; (InterPro:IPR009057); contains InterPro domain Myb-like DNA-binding region, SHAQKYF class; (InterPro:IPR006447); contains InterPro domain Myb, DNA-binding; (InterPro:IPR001005), myb family transcription factor; similar to DNA binding / transcription factor [Arabidopsis thaliana] (TAIR:AT3G09600.2); similar to MYB transcription factor MYB118 [Glycine max] (GB:ABH02853.1); contains InterPro domain Homeodomain-related; (InterPro:IPR012287); contains InterPro domain Homeodomain-like; (InterPro:IPR009057); contains InterPro domain Myb-like DNA-binding region, SHAQKYF class; (InterPro:IPR006447); contains InterPro domain Myb, DNA-binding; (InterPro:IPR001005) |
| 936 | 247222\_at | AT5G64840 | ATGCN5 | 2 | -1 | -0.2 | -0.2 | 0.1 | -0.1 | 0.8 | 0.1 | 0.2 | -0.5 | 0.3 | -0.1 | 0.1 | 0.2 | 0 | 1.3 | -1.8 | -1.6 | -0.2 | -0.3 | -0.2 | -0.1 | -0.2 | -0.3 | -0.5 | -0.3 | -1.2 | 0.1 | -0.2 | member of GCN subfamily |
| 935 | 267336\_at | AT2G19310 |  | 1 | 0.7 | -0.1 | 0.4 | 0 | 0 | 0.2 | 0.2 | 0 | -0.1 | 2.3 | -0.1 | -0.3 | -0.5 | 0 | 0.6 | -0.2 | -1.3 | -0.7 | 0.7 | 0.4 | 0.9 | 0.1 | 0 | -0.6 | 0.3 | -0.2 | 0.7 | 0.5 | similar to HSP18.2 (HEAT SHOCK PROTEIN 18.2) [Arabidopsis thaliana] (TAIR:AT5G59720.1); similar to low molecular weight heat-shock protein [Corylus avellana] (GB:AAD15628.1); similar to 18.5 kDa class I heat shock protein (HSP 18.5) (GB:P05478); contains InterPro domain HSP20-like chaperone; (InterPro:IPR008978) |
| 934 | 245047\_at | ATCG00020 | PSBA | 38 | -0.2 | -0.1 | -0.2 | 0.2 | 0 | -0.2 | 0.2 | -0.5 | 0.1 | 0.6 | -0.1 | 0 | -0.2 | 0.1 | 0.2 | -0.4 | -1.3 | -2 | -0.3 | 0.2 | 0.3 | 0.1 | -0.2 | -0.3 | 0.3 | -0.2 | 0 | 0.3 | Encodes chlorophyll binding protein D1, a part of the photosystem II reaction center core |
| 933 | 254688\_at | AT4G13830 | J20 |  | -1.3 | -0.3 | 0.1 | 0.2 | 0 | -1 | 0.5 | 0 | -0.7 | 0.4 | 0 | -0.6 | -0.1 | 0 | -0.2 | -1.2 | -1.8 | -1.8 | 0 | -0.2 | -0.9 | 0.3 | 0 | -0.8 | 1.3 | -0.7 | -0.1 | 0 | DnaJ-like protein (J20); nuclear gene |
| 932 | 253722\_at | AT4G29190 |  | 1 | -1.5 | -0.7 | -0.6 | -2.5 | -0.5 | 3.4 | 0.6 | 0 | -0.4 | 2.8 | 0.2 | -0.7 | 0.1 | 0.6 | -1.4 | -1.2 | -1.3 | -1.8 | -0.2 | 1.2 | 0.3 | 0.7 | 1.1 | -1.7 | 0.5 | -0.1 | -0.2 | -0.1 | zinc finger (CCCH-type) family protein; similar to zinc finger (CCCH-type) family protein [Arabidopsis thaliana] (TAIR:AT2G19810.1); similar to Zinc finger, CCCH-type [Medicago truncatula] (GB:ABE91976.1); contains InterPro domain Zinc finger, CCCH-type; (InterPro:IPR000571) |
| 931 | 247585\_at | AT5G60680 |  |  | -2.2 | 0 | 0.2 | -0.7 | -0.3 | 2.6 | 0.4 | -0.1 | -0.8 | 0.4 | -0.1 | -0.2 | -0.7 | -1 | 0.9 | -1.4 | -0.9 | -2.1 | -0.2 | 0.8 | -0.2 | 0.5 | 1.1 | -1.6 | -0.1 | 0.3 | -1 | -1.4 | similar to unknown protein [Arabidopsis thaliana] (TAIR:AT3G45210.1); similar to Protein of unknown function DUF584 [Medicago truncatula] (GB:ABE80969.1); contains InterPro domain Protein of unknown function DUF584; (InterPro:IPR007608) |
| 930 | 255381\_at | AT4G03510 | RMA1 | 4 | -1.3 | -0.4 | -0.3 | 0 | -0.4 | 0.2 | 0.2 | -0.2 | -1.1 | 1.4 | -0.3 | 0.7 | 0.3 | -0.4 | 0.8 | -1.3 | -2.6 | -1.8 | 0.9 | 1.1 | 0.7 | 0.6 | -0.1 | 0.3 | 1 | -0.3 | -0.2 | -0.3 | RMA1 encodes a novel 28 kDa protein with a RING finger motif and a C-terminal membrane-anchoring domain that is involved in the secretory pathway. |
| 929 | 246253\_at | AT4G37260 | ATMYB73, MYB73 | 4 | -2 | 0.1 | -0.4 | 0.1 | -0.3 | 1.9 | 0.2 | 0.1 | -0.9 | 2.2 | 0.1 | 0.3 | -0.8 | 1.3 | 0.7 | -0.4 | -2.2 | -1.6 | 0.6 | 0.9 | 1.1 | 0.4 | 0.7 | 1.2 | 0.8 | 0.4 | 0.1 | 0.3 | Member of the R2R3 factor gene family., Member of the R2R3 factor gene family. |
| 928 | 255621\_at | AT4G01390 |  |  | -1.4 | 0 | -0.2 | -0.1 | -1 | -0.5 | 0.5 | -0.3 | -2.5 | -4.6 | 1.4 | -3.6 | -0.3 | 5.3 | -3.3 | -1.1 | 2.9 | -3.9 | -1.4 | 1.2 | 1.4 | 0 | -0.4 | -1.1 | 0.4 | -2.4 | 0.9 | 0.6 | meprin and TRAF homology domain-containing protein / MATH domain-containing protein; similar to meprin and TRAF homology domain-containing protein / MATH domain-containing protein [Arabidopsis thaliana] (TAIR:AT4G00780.1); similar to Os12g0489100 [Oryza sativa (japonica cultivar-group)] (GB:NP\_001066793.1); similar to ubiquitin-specific protease 12, putative, expressed [Oryza sativa (japonica cultivar-group)] (GB:ABA98280.2); contains InterPro domain TRAF-like; (InterPro:IPR008974); contains InterPro domain MATH; (InterPro:IPR002083); contains InterPro domain TRAF-type; (InterPro:IPR013322) |
| 927 | 252183\_at | AT3G50740 | UGT72E1 | 4 | -0.7 | 0.1 | -0.2 | 0 | -0.3 | -7.2 | 0.2 | -0.7 | -0.4 | 1.1 | -0.2 | -0.5 | 2.9 | 2.4 | -3.9 | -0.1 | 1.1 | -1.4 | 1.8 | 2.6 | 1.3 | 0.3 | 0 | 0.1 | 1.4 | -1.9 | 0.1 | 0.2 | UGT72E1 is an UDPG:coniferyl alcohol glucosyltransferase which specifically glucosylates sinapyl- and coniferyl aldehydes. The enzyme is thought to be involved in lignin metabolism. |
| 926 | 253041\_at | AT4G37870 | PCK1, PEPCK, PCK1, PEPCK | 3 | 0.2 | -0.1 | -0.3 | -0.1 | -0.1 | -0.5 | 0.3 | -0.5 | -0.7 | -0.3 | -0.3 | 0 | 0.7 | 1.2 | 0.2 | -0.3 | 0.9 | -0.7 | -0.5 | 0.2 | 0.1 | 0 | 0.2 | 0.2 | 0.2 | 0.1 | -0.7 | -0.4 | ATP binding / phosphoenolpyruvate carboxykinase (ATP); Identical to Phosphoenolpyruvate carboxykinase [ATP] (EC 4.1.1.49) (PEP carboxykinase) (Phosphoenolpyruvate carboxylase) (PEPCK) [Arabidopsis Thaliana] (GB:Q9T074); similar to phosphoenolpyruvate carboxykinase (ATP), putative / PEP carboxykinase, putative / PEPCK, putative [Arabidopsis thaliana] (TAIR:AT5G65690.1); similar to phosphoenolpyruvate carboxykinase [Flaveria trinervia] (GB:BAB43908.1); similar to phosphoenolpyruvate carboxykinase [Flaveria pringlei] (GB:BAB43909.1); similar to phosphoenolpyruvate carboxykinase [Flaveria trinervia] (GB:BAB43907.1); contains InterPro domain Phosphoenolpyruvate carboxykinase (ATP); (InterPro:IPR001272); contains InterPro domain Phosphoenolpyruvate carboxykinase, N-terminal; (InterPro:IPR008210), ATP binding / phosphoenolpyruvate carboxykinase (ATP); Identical to Phosphoenolpyruvate carboxykinase [ATP] (EC 4.1.1.49) (PEP carboxykinase) (Phosphoenolpyruvate carboxylase) (PEPCK) [Arabidopsis Thaliana] (GB:Q9T074); similar to phosphoenolpyruvate carboxykinase (ATP), putative / PEP carboxykinase, putative / PEPCK, putative [Arabidopsis thaliana] (TAIR:AT5G65690.1); similar to phosphoenolpyruvate carboxykinase [Flaveria trinervia] (GB:BAB43908.1); similar to phosphoenolpyruvate carboxykinase [Flaveria pringlei] (GB:BAB43909.1); similar to phosphoenolpyruvate carboxykinase [Flaveria trinervia] (GB:BAB43907.1); contains InterPro domain Phosphoenolpyruvate carboxykinase (ATP); (InterPro:IPR001272); contains InterPro domain Phosphoenolpyruvate carboxykinase, N-terminal; (InterPro:IPR008210), Encodes a putative phosphoenolpyruvate carboxykinase (ATP-dependent)., Encodes a putative phosphoenolpyruvate carboxykinase (ATP-dependent). |
| 925 | 263831\_at | AT2G40300 | ATFER4 | 2 | -0.1 | -0.8 | 0.7 | 0.1 | 0 | 0.4 | -0.1 | 0 | -0.4 | -1 | 0.2 | 0.2 | -0.2 | 0.3 | -0.1 | -0.2 | -3 | -1 | 0.3 | -1.1 | 1.1 | -0.2 | -0.3 | 0 | -0.4 | -1.5 | 0 | 0 | ATFER4 (FERRITIN 4); ferric iron binding; Identical to Ferritin-4, chloroplast precursor (EC 1.16.3.1) (FER4) [Arabidopsis Thaliana] (GB:Q9S756;GB:Q8WHW5); similar to ATFER3 (FERRITIN 3), ferric iron binding [Arabidopsis thaliana] (TAIR:AT3G56090.1); similar to ferritin [Chorispora bungeana] (GB:ABB22752.1); contains InterPro domain Ferritin-related; (InterPro:IPR012347); contains InterPro domain Ferritin; (InterPro:IPR001519); contains InterPro domain Ferritin/ribonucleotide reductase-like; (InterPro:IPR009078); contains InterPro domain Ferritin-like; (InterPro:IPR009040); contains InterPro domain Ferritin and Dps; (InterPro:IPR008331) |
| 924 | 261956\_at | AT1G64590 |  |  | 1.7 | -0.6 | 0.2 | 0.8 | 0.7 | 0.8 | 2 | 0 | -0.1 | 0 | 0 | 0 | -0.1 | 0 | 0 | 0 | -1.3 | -0.1 | 0 | 0 | 0.1 | -1.8 | -0.9 | 0.5 | -6.6 | -1.7 | -0.2 | 0.9 | short-chain dehydrogenase/reductase (SDR) family protein; similar to short-chain dehydrogenase/reductase (SDR) family protein [Arabidopsis thaliana] (TAIR:AT4G24050.1); similar to Os06g0129100 [Oryza sativa (japonica cultivar-group)] (GB:NP\_001056678.1); similar to Short-chain dehydrogenase/reductase SDR [Medicago truncatula] (GB:ABE79697.1); similar to Os03g0115700 [Oryza sativa (japonica cultivar-group)] (GB:NP\_001048754.1); contains InterPro domain Short-chain dehydrogenase/reductase SDR; (InterPro:IPR002198); contains InterPro domain Glucose/ribitol dehydrogenase; (InterPro:IPR002347) |
| 923 | 255305\_at | AT4G04770 | ATABC1, ATNAP1, LAF6 | 7 | -0.1 | -0.4 | 0 | -0.2 | -0.1 | -0.1 | 0.1 | -0.3 | -0.6 | 0.2 | -0.1 | -0.1 | 0.2 | 0.8 | -0.2 | -0.1 | -2.1 | -1.1 | 0 | -0.3 | 0.5 | 0.1 | -0.2 | -0.2 | -0.4 | -1.3 | -0.1 | -0.2 | Encodes an iron-stimulated ATPase. A member of the NAP subfamily of ABC transporters. Involved in Fe-S cluster assembly. Similar to SufB. Involved in the regulation of iron homeostasis. Able to form homodimers. Interacts with AtNAP7 inside the chloroplast., Encodes an iron-stimulated ATPase. A member of the NAP subfamily of ABC transporters. Involved in Fe-S cluster assembly. Similar to SufB. Involved in the regulation of iron homeostasis. Able to form homodimers. Interacts with AtNAP7 inside the chloroplast., Encodes an iron-stimulated ATPase. A member of the NAP subfamily of ABC transporters. Involved in Fe-S cluster assembly. Similar to SufB. Involved in the regulation of iron homeostasis. Able to form homodimers. Interacts with AtNAP7 inside the chloroplast. |
| 922 | 252698\_at | AT3G43670 |  |  | -0.5 | -0.3 | 0.4 | -0.3 | -0.4 | 0 | -0.1 | -0.3 | -0.9 | 2.5 | -0.1 | -0.7 | 0.4 | 0.2 | -2.6 | -0.8 | -2.2 | -1.6 | 2.8 | -0.4 | 1 | -0.1 | -0.2 | -0.6 | -1.8 | -3.4 | -1.8 | -0.4 | copper amine oxidase, putative; similar to copper amine oxidase, putative [Arabidopsis thaliana] (TAIR:AT1G62810.1); similar to copper amine oxidase, putative [Arabidopsis thaliana] (TAIR:AT4G12290.1); similar to amine oxidase [Canavalia lineata] (GB:AAD49420.1); similar to copper-containing amine oxidase [Solanum lycopersicum] (GB:CAI39243.1); similar to Os04g0269600 [Oryza sativa (japonica cultivar-group)] (GB:NP\_001052338.1); contains InterPro domain Copper amine oxidase; (InterPro:IPR000269) |
| 921 | 264211\_at | AT1G22770 | FB, GI | 58 | 2.4 | -0.1 | -0.4 | -0.4 | -0.6 | 0.2 | -0.1 | -1.1 | -0.3 | 1.4 | 0.6 | -0.6 | -0.3 | 1.2 | -1.3 | 0.6 | -1.2 | -0.7 | 1.2 | 0.1 | 0.9 | 0.1 | -0.5 | -0.7 | -1.1 | -0.9 | -0.9 | -1.2 | Together with CONSTANTS (CO) and FLOWERING LOCUS T (FT), GIGANTEA promotes flowering under long days in a circadian clock-controlled flowering pathway. GI acts earlier than CO and FT in the pathway by increasing CO and FT mRNA abundance. Located in the nucleus. Regulates several developmental processes, including photoperiod-mediated flowering, phytochrome B signaling, circadian clock, carbohydrate metabolism, and cold stress response. The gene's transcription is controlled by the circadian clock and it is post-transcriptionally regulated by light and dark., Together with CONSTANTS (CO) and FLOWERING LOCUS T (FT), GIGANTEA promotes flowering under long days in a circadian clock-controlled flowering pathway. GI acts earlier than CO and FT in the pathway by increasing CO and FT mRNA abundance. Located in the nucleus. Regulates several developmental processes, including photoperiod-mediated flowering, phytochrome B signaling, circadian clock, carbohydrate metabolism, and cold stress response. The gene's transcription is controlled by the circadian clock and it is post-transcriptionally regulated by light and dark. |
| 920 | 266578\_at | AT2G23910 |  |  | 0.2 | -0.7 | 0.4 | 0.5 | 0.1 | 2.7 | -0.4 | -0.9 | -0.1 | 2.2 | 0 | 0.6 | -0.7 | 4.5 | -0.2 | 2 | 2.1 | 0.1 | 6.9 | 0.3 | 0 | -1.6 | -1.1 | 0.4 | -0.8 | 0.4 | 1.5 | 0.7 | cinnamoyl-CoA reductase-related; similar to cinnamoyl-CoA reductase-related [Arabidopsis thaliana] (TAIR:AT4G30470.1); similar to Ribosome-binding factor A [Medicago truncatula] (GB:ABE85146.1); contains InterPro domain NAD-dependent epimerase/dehydratase; (InterPro:IPR001509) |
| 919 | 258321\_at | AT3G22840 | ELIP, ELIP1 | 8 | 1.1 | -0.6 | -0.2 | 0.1 | -0.3 | 2.5 | -0.7 | 0.3 | -1.2 | 5 | -1 | 0.1 | -0.1 | 4.4 | 1.7 | 0 | 3.9 | 1.2 | 1.9 | -0.1 | -0.3 | -0.1 | -0.3 | -1 | -0.6 | -0.4 | -0.1 | -0.2 | Encodes an early light-inducible protein., Encodes an early light-inducible protein. |
| 918 | 252123\_at | AT3G51240 | F3'H, F3H, TT6 | 18 | 0.5 | -0.4 | 0.2 | 0.1 | -1.3 | 0.9 | -0.3 | -2.6 | -3.1 | 1.8 | -0.2 | -2.3 | -2.8 | 8.9 | 0.3 | -1.2 | 2.3 | 5.3 | 0.3 | 0 | -0.1 | -0.6 | -0.9 | 0.4 | -3.1 | -0.8 | -0.3 | -0.3 | Encodes flavanone 3-hydroxylase that is coordinately expressed with chalcone synthase and chalcone isomerases. Regulates flavonoid biosynthesis., Encodes flavanone 3-hydroxylase that is coordinately expressed with chalcone synthase and chalcone isomerases. Regulates flavonoid biosynthesis., Encodes flavanone 3-hydroxylase that is coordinately expressed with chalcone synthase and chalcone isomerases. Regulates flavonoid biosynthesis. |
| 917 | 250207\_at | AT5G13930 | ATCHS, CHS, TT4 | 60 | 0 | -0.5 | -0.1 | 0.2 | -2 | 0 | -0.3 | -1.2 | -2.7 | 0.2 | 0.2 | -5.3 | -5.3 | 5.2 | 0.4 | 0 | 2.4 | 2.3 | 0.3 | 0.2 | 0 | -0.3 | -0.4 | 0 | -1.6 | -1 | 0 | 0.1 | Encodes chalcone synthase (CHS), a key enzyme involved in the biosynthesis of flavonoids. Required for the accumulation of purple anthocyanins in leaves and stems. Also involved in the regulation of auxin transport and the modulation of root gravitropism., Encodes chalcone synthase (CHS), a key enzyme involved in the biosynthesis of flavonoids. Required for the accumulation of purple anthocyanins in leaves and stems. Also involved in the regulation of auxin transport and the modulation of root gravitropism., Encodes chalcone synthase (CHS), a key enzyme involved in the biosynthesis of flavonoids. Required for the accumulation of purple anthocyanins in leaves and stems. Also involved in the regulation of auxin transport and the modulation of root gravitropism. |
| 916 | 251827\_at | AT3G55120 | A11, CFI, TT5 | 25 | 0.5 | -0.2 | -0.6 | -0.1 | -0.2 | 0.5 | -0.2 | -0.9 | -1.4 | 0.5 | -1 | -0.8 | -0.1 | 2.3 | -0.7 | -0.5 | 1.9 | 0.6 | 0.3 | 0.1 | 0.3 | 0 | -0.6 | 0.4 | -1.5 | -1 | 0 | 0.1 | Catalyzes the conversion of chalcones into flavanones. Required for the accumulation of purple anthocyanins in leaves and stems., Catalyzes the conversion of chalcones into flavanones. Required for the accumulation of purple anthocyanins in leaves and stems., Catalyzes the conversion of chalcones into flavanones. Required for the accumulation of purple anthocyanins in leaves and stems. |
| 915 | 266141\_at | AT2G02120 | LCR70, PDF2.1 | 2 | -0.8 | 0.3 | 0 | 1 | 0.2 | 1 | 0.9 | 0.1 | 0 | 0.3 | -0.3 | -0.6 | -0.9 | 0.3 | -0.6 | -0.1 | 2.7 | 0.4 | -0.4 | -0.4 | -0.5 | 0.3 | -0.3 | -1.1 | -0.1 | -0.2 | 1.8 | 0.4 | LCR70/PDF2.1 (Low-molecular-weight cysteine-rich 70); protease inhibitor; Identical to Low-molecular-weight cysteine-rich protein LCR70 precursor (LCR70) [Arabidopsis Thaliana] (GB:Q41914;GB:Q9ZUL6); similar to LCR69/PDF2.2 (Low-molecular-weight cysteine-rich 69), protease inhibitor [Arabidopsis thaliana] (TAIR:AT2G02100.1); similar to protease inhibitor II (GB:AAA91049.1); contains InterPro domain Gamma thionin; (InterPro:IPR008176); contains InterPro domain Gamma Purothionin; (InterPro:IPR008177); contains InterPro domain Knottin; (InterPro:IPR003614), LCR70/PDF2.1 (Low-molecular-weight cysteine-rich 70); protease inhibitor; Identical to Low-molecular-weight cysteine-rich protein LCR70 precursor (LCR70) [Arabidopsis Thaliana] (GB:Q41914;GB:Q9ZUL6); similar to LCR69/PDF2.2 (Low-molecular-weight cysteine-rich 69), protease inhibitor [Arabidopsis thaliana] (TAIR:AT2G02100.1); similar to protease inhibitor II (GB:AAA91049.1); contains InterPro domain Gamma thionin; (InterPro:IPR008176); contains InterPro domain Gamma Purothionin; (InterPro:IPR008177); contains InterPro domain Knottin; (InterPro:IPR003614) |
| 914 | 260582\_at | AT2G47200 |  |  | -0.6 | 0 | 0.1 | 0.4 | 0 | 0.3 | -0.5 | 0 | 0.1 | 0.1 | 0 | -0.1 | -0.2 | -0.1 | 0 | 0.1 | 1.8 | 0.3 | -0.3 | -0.3 | -0.1 | -0.2 | 0.1 | -0.4 | -0.3 | -0.1 | 0.5 | 0 | unknown protein |
| 913 | 251531\_at | AT3G58550 |  | 1 | -0.7 | -0.2 | 0 | 0.2 | -0.5 | 0.3 | 0.1 | 0.6 | -0.4 | 0.6 | 0.2 | -0.1 | -0.3 | -0.5 | 0 | 0.3 | 1.2 | 0 | -0.4 | -0.2 | 0 | -0.2 | 0.9 | -1.4 | -2.4 | 0 | -0.4 | 1.4 | protease inhibitor/seed storage/lipid transfer protein (LTP) family protein; similar to lipid transfer protein-related [Arabidopsis thaliana] (TAIR:AT2G44300.1); similar to lipid transfer protein [Triticum aestivum] (GB:ABB90545.1); similar to Os03g0804200 [Oryza sativa (japonica cultivar-group)] (GB:NP\_001051617.1); similar to Os07g0198300 [Oryza sativa (japonica cultivar-group)] (GB:NP\_001059125.1); contains InterPro domain Plant lipid transfer/seed storage/trypsin-alpha amylase inhibitor; (InterPro:IPR003612) |
| 912 | 267121\_at | AT2G23540 |  |  | -0.3 | 0 | 0.2 | 0.6 | 0.1 | 0.9 | 0.4 | 0.1 | 0.1 | 3.4 | 0.2 | -0.1 | -0.1 | 0 | 0 | 0 | 1 | 0.1 | -0.4 | 0.8 | 1 | 0.1 | 0.1 | -0.2 | -2.2 | 0 | 1.1 | 0.9 | GDSL-motif lipase/hydrolase family protein; similar to GDSL-motif lipase/hydrolase family protein [Arabidopsis thaliana] (TAIR:AT3G50400.1); similar to H0404F02.4 [Oryza sativa (indica cultivar-group)] (GB:CAH66728.1); similar to Os03g0310000 [Oryza sativa (japonica cultivar-group)] (GB:NP\_001049914.1); contains InterPro domain Lipolytic enzyme, G-D-S-L; (InterPro:IPR001087) |
| 911 | 248144\_at | AT5G54800 | GPT1 | 1 | -0.5 | -0.1 | -0.4 | 0.1 | 0 | -0.7 | 0.2 | -0.2 | 0.2 | 0.5 | 0 | -0.3 | -0.2 | 0 | -0.4 | 0 | 0.9 | -0.1 | -0.5 | 0.5 | 0.2 | 0 | -0.1 | -0.8 | -0.2 | -0.3 | 0 | 0.1 | glucose6-Phosphate/phosphate transporter 1 |
| 910 | 249576\_at | AT5G37690 |  |  | -0.2 | -0.3 | 0.2 | -0.2 | -0.2 | -0.2 | -0.1 | 0.6 | -0.1 | 0 | 0 | -0.2 | -0.1 | 0.1 | 0 | 0.4 | 1.5 | -0.1 | -0.1 | 0.4 | 0.3 | -0.6 | -0.2 | -0.6 | -2.8 | -0.8 | 0.2 | 1.1 | GDSL-motif lipase/hydrolase family protein; similar to GDSL-motif lipase/hydrolase family protein [Arabidopsis thaliana] (TAIR:AT1G74460.1); similar to Os06g0257600 [Oryza sativa (japonica cultivar-group)] (GB:NP\_001057321.1); similar to GDSL-motif lipase/hydrolase-like [Oryza sativa (japonica cultivar-group)] (GB:BAD16143.1); contains InterPro domain Lipolytic enzyme, G-D-S-L; (InterPro:IPR001087) |
| 909 | 260803\_at | AT1G78340 | ATGSTU22 | 3 | -0.9 | -1.1 | -0.5 | 0.8 | 0.1 | -1.5 | 1.6 | 0.2 | 0 | -0.1 | -0.2 | -0.2 | -0.3 | -0.2 | -0.1 | 0.1 | 1.3 | 0.1 | -0.1 | -0.1 | 1.4 | -0.1 | -0.6 | -2.4 | 2.1 | 1.3 | 5.6 | 4.7 | Encodes glutathione transferase belonging to the tau class of GSTs. Naming convention according to Wagner et al. (2002). |
| 908 | 248978\_at | AT5G45070 | AtPP2-A8 | 1 | -4 | -0.5 | -1.5 | 0.6 | 0.6 | 1.9 | -0.6 | 0 | 0.1 | -0.1 | 0 | -0.1 | 0 | -0.1 | 0 | 0 | 2 | -0.2 | 0 | -0.1 | 0 | 0.8 | -2.5 | -2.3 | 3.9 | -1.6 | 2.1 | 7.5 | ATPP2-A8 (Phloem protein 2-A8); transmembrane receptor; similar to ATPP2-A6 (Phloem protein 2-A6), transmembrane receptor [Arabidopsis thaliana] (TAIR:AT5G45080.1); similar to TIR-NBS-LRR type disease resistance protein [Populus trichocarpa] (GB:ABF81468.1); contains InterPro domain Toll-Interleukin receptor; (InterPro:IPR000157); contains InterPro domain Galactose-binding like; (InterPro:IPR008979) |
| 907 | 262317\_at | AT2G48140 | EDA4 | 2 | -0.4 | -0.4 | -0.4 | 0.8 | 0 | 1.9 | 0.8 | 0 | 0.1 | 0.2 | -0.1 | 0 | 0 | 0 | 0 | 0 | 0.9 | 0 | -0.1 | -0.1 | 0 | -1.8 | -0.7 | 0 | 0.3 | 0.5 | 1.5 | 1.8 | EDA4 (embryo sac development arrest 4); lipid binding; similar to protease inhibitor/seed storage/lipid transfer protein (LTP) family protein [Arabidopsis thaliana] (TAIR:AT3G22620.1); similar to Plant lipid transfer/seed storage/trypsin-alpha amylase inhibitor [Medicago truncatula] (GB:ABE87134.1); contains InterPro domain Plant lipid transfer/seed storage/trypsin-alpha amylase inhibitor; (InterPro:IPR003612) |
| 906 | 245305\_at | AT4G17215 |  |  | -0.7 | -0.3 | -0.5 | 0.4 | 1.2 | 2.2 | 0.4 | 0 | 0.2 | 1.1 | 0.4 | 0.1 | 0.6 | 0.4 | 0.8 | 2.9 | 1.4 | 0.7 | -0.3 | -0.8 | 0 | -0.1 | -0.5 | -0.6 | 1.6 | 0.5 | 2.5 | 1.3 | similar to unknown protein [Arabidopsis thaliana] (TAIR:AT5G47635.1); similar to hypothetical protein 40.t00006 [Brassica oleracea] (GB:ABD65131.1) |
| 905 | 259478\_at | AT1G18980 |  |  | 0 | -0.2 | -0.3 | 0.8 | 0.1 | 0.5 | 0.3 | 0 | 1.5 | 0.1 | -0.6 | 1.8 | 0.4 | -0.3 | 0.6 | 0.7 | 1 | 0.1 | -0.1 | 0.2 | 1.7 | 0.7 | 0 | -1.1 | -1.2 | 0.6 | 0.9 | 1.8 | germin-like protein, putative; Identical to Germin-like protein subfamily T member 2 precursor [Arabidopsis Thaliana] (GB:Q9LMC9); similar to GLP4 (GERMIN-LIKE PROTEIN 4), manganese ion binding / metal ion binding / nutrient reservoir [Arabidopsis thaliana] (TAIR:AT1G18970.1); similar to germin-like protein [Ananas comosus] (GB:AAM28275.1); contains InterPro domain Cupin 1; (InterPro:IPR006045); contains InterPro domain Cupin, RmlC-type; (InterPro:IPR011051); contains InterPro domain Cupin region; (InterPro:IPR007113); contains InterPro domain Germin; (InterPro:IPR001929) |
| 904 | 247765\_at | AT5G58860 | CYP86, CYP86A1 | 6 | -0.5 | 0 | -0.3 | 0.2 | 0 | 0.6 | 0.4 | 0 | 0 | 0.1 | 0 | 0 | 0 | 0 | 0 | 0.1 | 1 | 0 | 0 | 0.1 | 0.1 | -0.2 | -0.2 | -0.3 | -1.9 | 0 | 0.4 | 0 | Encodes a member of the CYP86A subfamily of cytochrome p450 genes. Expressed significantly only in root tissue., Encodes a member of the CYP86A subfamily of cytochrome p450 genes. Expressed significantly only in root tissue. |
| 903 | 259276\_at | AT3G01190 |  |  | -2.5 | -0.2 | -0.1 | 1.7 | 2.2 | -0.1 | 0 | 0.1 | -0.1 | 0 | 0 | 0 | -0.1 | -0.1 | 0 | 0.1 | 1.9 | 0 | 0 | 0 | 0 | -0.6 | -5 | -0.9 | -4.6 | -1.6 | -0.5 | 2.1 | peroxidase 27 (PER27) (P27) (PRXR7); Identical to Peroxidase 27 precursor (EC 1.11.1.7) (Atperox P27) (PRXR7) (ATP12a) (PER27) [Arabidopsis Thaliana] (GB:Q43735); similar to peroxidase, putative [Arabidopsis thaliana] (TAIR:AT5G15180.1); similar to peroxidase [Spinacia oleracea] (GB:CAA76374.2); contains InterPro domain Haem peroxidase; (InterPro:IPR010255); contains InterPro domain Haem peroxidase, plant/fungal/bacterial; (InterPro:IPR002016); contains InterPro domain Plant peroxidase; (InterPro:IPR000823) |
| 902 | 253957\_at | AT4G26320 | AGP13, AGP13 | 2 | -1.4 | -0.3 | 0.4 | 1.2 | 0.7 | -1.1 | -0.1 | 0.1 | 0 | -0.1 | -0.3 | 0 | -0.1 | -0.2 | 0 | -0.3 | 2.7 | -0.1 | -0.1 | -0.4 | -0.4 | -2.1 | -4.5 | -0.7 | -6 | -1.6 | -0.4 | 0.2 | AGP13 (ARABINOGALACTAN PROTEIN 13); Identical to Arabinogalactan peptide 13 precursor (AG-peptide 13) (AGP13) [Arabidopsis Thaliana] (GB:Q9STQ3); similar to AGP14 (ARABINOGALACTAN PROTEIN 14) [Arabidopsis thaliana] (TAIR:AT5G56540.1); similar to unknown [Brassica rapa] (GB:ABB97032.1) |
| 901 | 257946\_at | AT3G21710 |  |  | 0.2 | 0.1 | 0.4 | 0.6 | 2.3 | 0.3 | 0.4 | 0.6 | 0.4 | 1.2 | -0.1 | -0.1 | -0.3 | 2.3 | 0.5 | -0.1 | 1.4 | 0.1 | -0.2 | -0.2 | 0.5 | 0.2 | -0.1 | -0.4 | -1 | -0.2 | -0.5 | 0.4 | similar to Os07g0524000 [Oryza sativa (japonica cultivar-group)] (GB:NP\_001059820.1), similar to Os07g0524000 [Oryza sativa (japonica cultivar-group)] (GB:NP\_001059820.1); similar to hypothetical protein S250\_18C08.7 [Sorghum bicolor] (GB:AAS82597.1) |
| 900 | 267307\_at | AT2G30210 | LAC3 | 2 | -0.4 | -1.1 | -0.4 | 0 | 0.1 | -0.1 | -0.4 | 0.4 | 0.2 | -0.7 | -0.2 | -0.1 | -0.2 | -1.5 | -0.1 | 0.1 | 1.5 | 0 | -0.6 | -0.6 | -0.8 | -0.6 | -0.5 | -1.2 | -3.8 | -0.7 | -2.2 | -0.3 | putative laccase, a member of laccase family of genes (17 members in Arabidopsis). |
| 899 | 254820\_s\_at | AT4G12510, AT4G12520 AT4G12510, AT4G12520 |  |  | 0.5 | 0.1 | 0.2 | 0.6 | 0.7 | -0.7 | 0 | 1 | 0.6 | -0.2 | 0.2 | -0.3 | -0.6 | -1.8 | 0.3 | 0.7 | 1.1 | 0 | -0.5 | -1 | -0.5 | -1.7 | -3.4 | -0.1 | -3.7 | -1.9 | -3.3 | -1.1 | protease inhibitor/seed storage/lipid transfer protein (LTP) family protein; similar to protease inhibitor/seed storage/lipid transfer protein (LTP) family protein [Arabidopsis thaliana] (TAIR:AT4G12520.1); similar to extensin like protein [Populus nigra] (GB:BAA11854.1); contains InterPro domain Plant lipid transfer/seed storage/trypsin-alpha amylase inhibitor; (InterPro:IPR003612), protease inhibitor/seed storage/lipid transfer protein (LTP) family protein; similar to protease inhibitor/seed storage/lipid transfer protein (LTP) family protein [Arabidopsis thaliana] (TAIR:AT4G12510.1); similar to extensin like protein [Populus nigra] (GB:BAA11854.1); contains InterPro domain Plant lipid transfer/seed storage/trypsin-alpha amylase inhibitor; (InterPro:IPR003612) |
| 898 | 267053\_s\_at | AT2G38380, AT2G38390 AT2G38380, AT2G38390 |  |  | -0.1 | 0 | 0.2 | 0.1 | 0.7 | -0.7 | -0.2 | 0 | -0.8 | 0.4 | 0 | 6.1 | 1.2 | 1.8 | 3.2 | 1.2 | 1.4 | 1.6 | -0.8 | 0.7 | 1.2 | 0.3 | 0.1 | 0 | -0.9 | -0.2 | -1.7 | 0.3 | peroxidase 22 (PER22) (P22) (PRXEA) / basic peroxidase E; Identical to Peroxidase 22 precursor (EC 1.11.1.7) (Atperox P22) (ATPEa) (Basic peroxidase E) (PER22) [Arabidopsis Thaliana] (GB:P24102;GB:O80913); similar to peroxidase, putative [Arabidopsis thaliana] (TAIR:AT2G38390.1); similar to peroxidase (GB:AAA32842.1); contains InterPro domain Haem peroxidase; (InterPro:IPR010255); contains InterPro domain Haem peroxidase, plant/fungal/bacterial; (InterPro:IPR002016); contains InterPro domain Plant peroxidase; (InterPro:IPR000823), peroxidase, putative; Identical to Peroxidase 23 precursor (EC 1.11.1.7) (Atperox P23) (ATP34) (PER23) [Arabidopsis Thaliana] (GB:O80912); similar to peroxidase 22 (PER22) (P22) (PRXEA) / basic peroxidase E [Arabidopsis thaliana] (TAIR:AT2G38380.1); similar to Peroxidase C3 precursor (GB:P17180); contains InterPro domain Haem peroxidase; (InterPro:IPR010255); contains InterPro domain Haem peroxidase, plant/fungal/bacterial; (InterPro:IPR002016); contains InterPro domain Plant peroxidase; (InterPro:IPR000823) |
| 897 | 247059\_at | AT5G66690 | UGT72E2 | 3 | 0.4 | -0.1 | -0.3 | 0.4 | 0.7 | -3 | -0.1 | -0.2 | 0.4 | 2.6 | 0 | 0.7 | 1 | 1.3 | 2.1 | 1.4 | 2.1 | 0.8 | -1 | 0 | 0.8 | -0.5 | -0.7 | -1.7 | -3.2 | -2.4 | -3.7 | -0.7 | UGT72E2 is an UDPG:coniferyl alcohol glucosyltransferase which glucosylates sinapyl- and coniferyl aldehydes as well as sinapyl- and coniferyl alcohol. The enzyme is thought to be involved in lignin metabolism. A knockdown mutant line (72E2KD) was obtained using RNAi silencing. A twofold reduction in coniferyl alcohol 4-*O*-glucoside and sinapyl alcohol 4-*O*-glucoside was detected in this line compared to wildtype. In comparison, both knockout and knockdown lines of UGT72E1 and UGT72E3, respectively, failed to display the same reduction in phenylpropanoid 4-*O*-glucosides. |
| 896 | 261806\_at | AT1G30510 | ATRFNR2 |  | -0.4 | -0.3 | -0.1 | -0.1 | -0.4 | -1.2 | -0.4 | -0.3 | -0.2 | 1.2 | -0.1 | -0.2 | 0 | 0.6 | -0.3 | -0.3 | 1.2 | -0.3 | -0.5 | 0.1 | 0.2 | -0.2 | -0.4 | -1.2 | -0.9 | -2 | -1.2 | -0.2 | Encodes a root-type ferredoxin:NADP(H) oxidoreductase. |
| 895 | 262128\_at | AT1G52690 |  | 1 | 0.6 | 0.1 | 0 | 0 | -0.7 | 12.2 | 0.3 | 0 | 0.1 | 12.4 | 2.3 | 7.8 | -0.4 | 0 | 7.2 | 0.6 | 6.4 | -0.1 | -0.4 | -0.3 | 0.3 | 0.1 | 0 | 0.2 | 0.2 | 5 | 0.5 | -0.9 | late embryogenesis abundant protein, putative / LEA protein, putative; similar to late embryogenesis abundant protein, putative / LEA protein, putative [Arabidopsis thaliana] (TAIR:AT3G15670.1); similar to late embryogenesis-abundant protein [Chorispora bungeana] (GB:AAV67892.1); contains InterPro domain Late embryogenesis abundant protein; (InterPro:IPR004238) |
| 894 | 258498\_at | AT3G02480 |  |  | -0.3 | 0.5 | -1.8 | 1.1 | 0 | 8.8 | -0.5 | 0 | 0.1 | 13.1 | 0.4 | 7.3 | -0.6 | 0.2 | 6.4 | 0.9 | 8.3 | 0.4 | -0.4 | -0.4 | 0.4 | -1.6 | -1.7 | -1.2 | 0.4 | 2.6 | 0.6 | -0.5 | ABA-responsive protein-related; similar to unknown protein [Arabidopsis thaliana] (TAIR:AT5G38760.1); similar to hypothetical protein [Cleome spinosa] (GB:ABD96872.1); contains InterPro domain Late embryogenesis abundant protein; (InterPro:IPR004238) |
| 893 | 261149\_s\_at | AT1G19550, AT1G19570 AT1G19550, AT1G19570 | DHAR1, DHAR1 | 8 | -0.2 | 0.1 | -0.3 | -0.1 | -0.1 | 0.4 | 0 | -0.4 | -0.9 | 1.4 | 0.3 | 0.2 | -1.1 | 0.1 | 0.9 | 0.9 | 0.7 | 0.9 | -0.6 | 0.3 | 0 | 0.2 | -0.2 | 1.3 | -0.2 | 0.7 | 0 | -0.2 | dehydroascorbate reductase, putative; similar to DHAR1 (DEHYDROASCORBATE REDUCTASE), glutathione dehydrogenase (ascorbate) [Arabidopsis thaliana] (TAIR:AT1G19570.1); similar to dehydroascorbate reductase [Zinnia elegans] (GB:BAD27392.1); contains InterPro domain Thioredoxin-like fold; (InterPro:IPR012336); contains InterPro domain Glutathione S-transferase, C-terminal-like; (InterPro:IPR010987); contains InterPro domain Thioredoxin fold; (InterPro:IPR012335); contains InterPro domain Glutathione S-transferase, C-terminal; (InterPro:IPR004046), DHAR1 (DEHYDROASCORBATE REDUCTASE); glutathione dehydrogenase (ascorbate); similar to DHAR2, glutathione dehydrogenase (ascorbate) [Arabidopsis thaliana] (TAIR:AT1G75270.1); similar to dehydroascorbate reductase-like protein [Solanum tuberosum] (GB:ABA46750.1); contains InterPro domain Thioredoxin-like fold; (InterPro:IPR012336); contains InterPro domain Glutathione S-transferase, N-terminal; (InterPro:IPR004045); contains InterPro domain Glutathione S-transferase, C-terminal-like; (InterPro:IPR010987); contains InterPro domain Thioredoxin fold; (InterPro:IPR012335); contains InterPro domain Glutathione S-transferase, C-terminal; (InterPro:IPR004046), DHAR1 (DEHYDROASCORBATE REDUCTASE); similar to DHAR2, glutathione dehydrogenase (ascorbate) [Arabidopsis thaliana] (TAIR:AT1G75270.1); similar to dehydroascorbate reductase-like protein [Solanum tuberosum] (GB:ABA46750.1); contains InterPro domain Thioredoxin-like fold; (InterPro:IPR012336); contains InterPro domain Glutathione S-transferase, N-terminal; (InterPro:IPR004045); contains InterPro domain Glutathione S-transferase, C-terminal-like; (InterPro:IPR010987); contains InterPro domain Thioredoxin fold; (InterPro:IPR012335); contains InterPro domain Glutathione S-transferase, C-terminal; (InterPro:IPR004046) |
| 892 | 254232\_at | AT4G23600 | CORI3, JR2 | 8 | 0.6 | -0.1 | -0.8 | -0.7 | -0.7 | 1.1 | -0.5 | -0.5 | -1 | 7.1 | 0.2 | 0.9 | -2.1 | 0.3 | 1.6 | 1.6 | 2.7 | 1 | -1.5 | 2.2 | 2.4 | -0.4 | 0.1 | 2.7 | 0.7 | 1.9 | 0.8 | 0.4 | Encodes cystine lyase which is expected to be involved in amino acid metabolism, providing the plant with cysteine and the generation of precursors of ethylene biosynthesis. mRNA levels are elevated in response to wounding., Encodes cystine lyase which is expected to be involved in amino acid metabolism, providing the plant with cysteine and the generation of precursors of ethylene biosynthesis. mRNA levels are elevated in response to wounding. |
| 891 | 247718\_at | AT5G59310 | LTP4 | 3 | -0.5 | 0 | 0 | 0 | -1.4 | 7.6 | 0 | 0.1 | -0.1 | 10.5 | 2.3 | 6.6 | 1.2 | 2.5 | 7.2 | 1 | 5.8 | -6.3 | -0.5 | 1.1 | 0.8 | 0 | 0 | -0.3 | 0 | 1.2 | 0.2 | 0 | Encodes a member of the lipid transfer protein family. Proteins of this family are generally small (~9 kD), basic, expressed abundantly and contain eight Cys residues. The proteins can bind fatty acids and acylCoA esters and can transfer several different phospholipids. They are localized to the cell wall. The mRNA is present in flowers and siliques, and is strongly up-regulated by abscisic acid. |
| 890 | 247717\_at | AT5G59320 | LTP3 | 3 | -1 | -0.8 | -0.7 | 1.3 | -1.2 | 7.6 | 0 | -1 | -3.1 | 13.4 | 0.2 | 5 | 0.5 | 4.2 | 5.1 | 2.1 | 6 | -6.4 | 0 | 3.3 | 4.5 | -1.1 | 0.6 | -0.3 | -0.6 | -0.3 | -0.1 | 1.1 | lipid transfer protein 3 |
| 889 | 252102\_at | AT3G50970 | LTI30, XERO2 | 9 | -0.4 | 0 | 0 | 0 | -0.5 | 5.6 | 0.2 | -1.1 | -1.4 | 11.2 | 2 | 1.8 | -3.1 | 4.4 | 2.3 | 2.1 | 0.9 | -1.1 | 0 | -0.3 | 3 | 1.1 | 1.2 | 0.2 | 0.7 | 1.6 | 1.8 | 1.3 | Belongs to the dehydrin protein family, which contains highly conserved stretches of 7-17 residues that are repetitively scattered in their sequences, the K-, S-, Y- and lysine rich segments. LTI29 and LTI30 double overexpressors confer freeze tolerance. Located in membranes. mRNA upregulated by water deprivation and abscisic acid., Belongs to the dehydrin protein family, which contains highly conserved stretches of 7-17 residues that are repetitively scattered in their sequences, the K-, S-, Y- and lysine rich segments. LTI29 and LTI30 double overexpressors confer freeze tolerance. Located in membranes. mRNA upregulated by water deprivation and abscisic acid. |
| 888 | 267080\_at | AT2G41190 |  |  | -0.5 | -0.5 | -0.1 | 0.1 | -0.4 | 7.2 | 0 | 0.4 | -1.2 | 8.5 | 1.4 | 1.7 | -1.5 | 1.4 | 3.1 | 1.6 | 3.6 | -0.1 | -0.4 | 0.3 | -0.3 | -0.2 | -0.4 | -0.8 | -0.2 | -0.2 | 0.7 | 0.7 | amino acid transporter family protein; similar to amino acid transporter family protein [Arabidopsis thaliana] (TAIR:AT2G39130.1); similar to Amino acid/polyamine transporter II [Medicago truncatula] (GB:ABE77592.1); contains InterPro domain Amino acid/polyamine transporter II; (InterPro:IPR002422); contains InterPro domain Amino acid transporter, transmembrane; (InterPro:IPR013057) |
| 887 | 255795\_at | AT2G33380 | RD20 | 4 | -0.4 | 0.9 | 0.1 | -0.4 | -0.3 | 11.5 | 0 | -0.7 | -1.3 | 3.6 | 1.7 | 3.7 | -1.2 | 2.5 | 4 | 5.2 | 4.2 | 2.5 | -1.9 | -0.4 | -0.2 | 0 | 0.4 | 5.7 | 2.4 | 9 | 0 | 0 | Encodes a calcium binding protein whose mRNA is induced upon treatment with NaCl, ABA and in response to dessication. mRNA expression under drought conditions is apparent particularly in leaves and flowers. |
| 886 | 247095\_at | AT5G66400 | ATDI8, RAB18 | 33 | -1.7 | -0.2 | 0.1 | 0 | -0.2 | 7.6 | -0.6 | -0.8 | -0.6 | 6.7 | 0.7 | 1.6 | -0.5 | 0.4 | 2.7 | 1.3 | 2 | -1 | -1.1 | -0.3 | -0.8 | 0.1 | -0.2 | 0.6 | 0 | 1 | 0.3 | -0.8 | Belongs to the dehydrin protein family, which contains highly conserved stretches of 7-17 residues that are repetitively scattered in their sequences, the K-, S-, Y- and lysine rich segments. ABA- and drought-induced glycine-rice dehydrin protein. The ABA-induced expression of RAB18 was reduced following ACC application, indicating that ethylene inhibits the ABA signaling pathway. RAB18 is also expressed in response to the formation of the phospholipid diacylglycerol pyrophosphate. COR47 and RAB18 double overexpressor plants are cold tolerant. Expressed in guard cells., Belongs to the dehydrin protein family, which contains highly conserved stretches of 7-17 residues that are repetitively scattered in their sequences, the K-, S-, Y- and lysine rich segments. ABA- and drought-induced glycine-rice dehydrin protein. The ABA-induced expression of RAB18 was reduced following ACC application, indicating that ethylene inhibits the ABA signaling pathway. RAB18 is also expressed in response to the formation of the phospholipid diacylglycerol pyrophosphate. COR47 and RAB18 double overexpressor plants are cold tolerant. Expressed in guard cells. |
| 885 | 263497\_at | AT2G42540 | COR15, COR15A | 34 | -1.1 | -1 | -0.7 | 0 | -0.4 | 9.2 | -0.5 | -0.9 | -1.6 | 5.5 | -0.1 | -3 | -4.8 | 0.7 | -1 | -1.6 | 1.9 | -1.4 | -5.1 | -1.8 | -0.9 | -0.2 | 0.1 | 0.9 | 0.5 | -1.3 | 0.3 | 0.1 | A cold-regulated gene whose product is targeted to the chloroplast and constitutive expression increases freezing tolerance in protoplasts in vitro and chloroplasts in vivo. NMR and x-ray diffraction studies suggest that COR15a alters the intrinsic curvature of the inner membrane of chloroplast envelope., A cold-regulated gene whose product is targeted to the chloroplast and constitutive expression increases freezing tolerance in protoplasts in vitro and chloroplasts in vivo. NMR and x-ray diffraction studies suggest that COR15a alters the intrinsic curvature of the inner membrane of chloroplast envelope. |
| 884 | 262657\_at | AT1G14210 |  |  | 0.2 | 0.2 | -0.1 | 0 | 0.6 | -1.4 | -0.2 | 0.6 | 0.7 | -4.7 | -0.2 | -2.3 | 0.1 | -0.5 | -2.7 | -1 | 1.7 | -1 | 0.1 | -0.4 | -1.3 | 0.2 | -0.1 | -0.1 | -0.6 | -0.2 | -0.3 | 0 | ribonuclease T2 family protein; similar to ribonuclease T2 family protein [Arabidopsis thaliana] (TAIR:AT1G14220.1); similar to wounding-induced ribonuclease gene (GB:AAC49326.1); contains InterPro domain Ribonuclease T2; (InterPro:IPR001568) |
| 883 | 257217\_at | AT3G14940 | ATPPC3 | 4 | -1.2 | 0.1 | 0.2 | 0.1 | 0.7 | -1.2 | 0 | 0.3 | 0.7 | -3.1 | -0.3 | -1.8 | -2 | -1.5 | -2.5 | -0.8 | 1.1 | -2 | -1.8 | -1.1 | -1.9 | 0.4 | 0.2 | -0.9 | -2.2 | -1.9 | -0.2 | 2.2 | One of four genes encoding phosphoenolpyruvate carboxylase, its mRNA is most abundantly expressed in roots and siliques. |
| 882 | 248252\_at | AT5G53250 | AGP22, ATAGP22 | 3 | -0.5 | 0.4 | 0.5 | 0.8 | 1.2 | -1.1 | -0.4 | -0.7 | 0.2 | -2.3 | -0.2 | -2.6 | -0.9 | -1.5 | -1 | 0.1 | 1.1 | -1.1 | -1.1 | -1.3 | -0.2 | -0.6 | -1.1 | -0.3 | -4.2 | -1.1 | -1.6 | -0.3 | AGP22/ATAGP22 (ARABINOGALACTAN PROTEINS 22); Identical to Arabinogalactan peptide 22 precursor (AG-peptide 22) (AGP22) [Arabidopsis Thaliana] (GB:Q9FK16); similar to AGP41 [Arabidopsis thaliana] (TAIR:AT5G24105.1); similar to unknown [Capsicum annuum] (GB:AAM12785.1); similar to Os02g0264800 [Oryza sativa (japonica cultivar-group)] (GB:NP\_001046496.1); similar to Os01g0657000 [Oryza sativa (japonica cultivar-group)] (GB:NP\_001043758.1); contains InterPro domain Protein of unknown function DUF1070; (InterPro:IPR009424), AGP22/ATAGP22 (ARABINOGALACTAN PROTEINS 22); Identical to Arabinogalactan peptide 22 precursor (AG-peptide 22) (AGP22) [Arabidopsis Thaliana] (GB:Q9FK16); similar to AGP41 [Arabidopsis thaliana] (TAIR:AT5G24105.1); similar to unknown [Capsicum annuum] (GB:AAM12785.1); similar to Os02g0264800 [Oryza sativa (japonica cultivar-group)] (GB:NP\_001046496.1); similar to Os01g0657000 [Oryza sativa (japonica cultivar-group)] (GB:NP\_001043758.1); contains InterPro domain Protein of unknown function DUF1070; (InterPro:IPR009424) |
| 881 | 259813\_at | AT1G49860 | ATGSTF14 | 2 | -2.7 | 0.2 | 0.1 | -0.8 | 0.7 | -3.4 | -3.4 | 0.1 | 0 | -0.2 | 0 | -0.2 | 0 | 0.2 | -0.1 | 0 | 1.7 | -0.1 | -0.1 | 0 | 0 | -0.6 | -1 | -4 | -2.7 | -5.4 | -2.9 | -2.3 | Encodes glutathione transferase belonging to the phi class of GSTs. Naming convention according to Wagner et al. (2002). |
| 880 | 246390\_at | AT1G77330 |  |  | -1.7 | 0.4 | 0.6 | -0.8 | 0.7 | -0.5 | 0.4 | 0 | -0.3 | -0.1 | 0.2 | -0.6 | 0.1 | 0.4 | 0.3 | 0 | 2 | 0 | 0 | 0 | 0 | 1.3 | 0.2 | -0.2 | -2.2 | 0 | -1.9 | -1.4 | similar to 1-aminocyclopropane-1-carboxylate oxidase GI:3386565 from (Sorghum bicolor) |
| 879 | 252958\_at | AT4G38620 | ATMYB4, MYB4 | 5 | 0.2 | -0.2 | 0.2 | 0.7 | 0.2 | -1.6 | 0.4 | -0.4 | 0 | -0.6 | -0.7 | -0.6 | 0.2 | -0.4 | -0.3 | 0.1 | 3.1 | -1.2 | -0.3 | 0.1 | -0.1 | 0.3 | -1 | -0.3 | 1.1 | 0.3 | -0.1 | -0.5 | Encodes a R2R3 MYB protein which is involved in the response to UV-B. It functions as a repressor of target gene expression. One of its target genes encodes cinnamate 4-hydroxylase; mutants accumulate sinapate esters in their leaves., Encodes a R2R3 MYB protein which is involved in the response to UV-B. It functions as a repressor of target gene expression. One of its target genes encodes cinnamate 4-hydroxylase; mutants accumulate sinapate esters in their leaves. |
| 878 | 246228\_at | AT4G36430 |  | 1 | -0.2 | 0.4 | 0.2 | 0.1 | 0.7 | -0.5 | -0.2 | 0.7 | -0.5 | -1.7 | 0.7 | -0.7 | 4.9 | -2.7 | -1.1 | 2.3 | 2.3 | 0 | -1.1 | 0.1 | -0.2 | 0.6 | 0.6 | -0.5 | 5.1 | 1.5 | 1.7 | 0.3 | peroxidase, putative; Identical to Peroxidase 49 precursor (EC 1.11.1.7) (Atperox P49) (ATP31) (PER49) [Arabidopsis Thaliana] (GB:O23237); similar to peroxidase, putative [Arabidopsis thaliana] (TAIR:AT2G18150.1); similar to peroxidase [Dimocarpus longan] (GB:ABG49114.1); contains InterPro domain Haem peroxidase; (InterPro:IPR010255); contains InterPro domain Haem peroxidase, plant/fungal/bacterial; (InterPro:IPR002016); contains InterPro domain Plant peroxidase; (InterPro:IPR000823) |
| 877 | 266165\_at | AT2G28190 | CSD2, CZSOD2, CSD2, CZSOD2 | 15 | 0.1 | -1.9 | -0.6 | 0 | 2.1 | -1.9 | 0.2 | -0.1 | 0.4 | -2.9 | -0.5 | -0.3 | -0.1 | -3.5 | -0.7 | -0.4 | 1.6 | 0.8 | 2.4 | -0.9 | -0.8 | 0 | -0.4 | -0.9 | -0.3 | -0.3 | -0.7 | -0.4 | Encodes a chloroplastic copper/zinc superoxide dismutase CSD2 that can detoxify superoxide radicals. Its expression is affected by miR398-directed mRNA cleavage., Encodes a chloroplastic copper/zinc superoxide dismutase CSD2 that can detoxify superoxide radicals. Its expression is affected by miR398-directed mRNA cleavage., Encodes a chloroplastic copper/zinc superoxide dismutase CSD2 that can detoxify superoxide radicals. Its expression is affected by miR398-directed mRNA cleavage., Encodes a chloroplastic copper/zinc superoxide dismutase CSD2 that can detoxify superoxide radicals. Its expression is affected by miR398-directed mRNA cleavage. |
| 876 | 255822\_at | AT2G40610 | ATEXP8, ATEXPA8, ATHEXP, EXP8 | 4 | 0.8 | 0.4 | 1.3 | -1.8 | 0 | -6.9 | -0.8 | 0.1 | -0.7 | -11 | -0.7 | -5.9 | -1.8 | -10.8 | -9.1 | -6.7 | -8.2 | -2.5 | -0.5 | -0.8 | -1.8 | -6.1 | 1.3 | -3.4 | -3.3 | -6.3 | -3.3 | -0.6 | member of Alpha-Expansin Gene Family. Naming convention from the Expansin Working Group (Kende et al, 2004. Plant Mol Bio). Involved in the formation of nematode-induced syncytia in roots of Arabidopsis thaliana., member of Alpha-Expansin Gene Family. Naming convention from the Expansin Working Group (Kende et al, 2004. Plant Mol Bio). Involved in the formation of nematode-induced syncytia in roots of Arabidopsis thaliana., member of Alpha-Expansin Gene Family. Naming convention from the Expansin Working Group (Kende et al, 2004. Plant Mol Bio). Involved in the formation of nematode-induced syncytia in roots of Arabidopsis thaliana., member of Alpha-Expansin Gene Family. Naming convention from the Expansin Working Group (Kende et al, 2004. Plant Mol Bio). Involved in the formation of nematode-induced syncytia in roots of Arabidopsis thaliana. |
| 875 | 252965\_at | AT4G38860 |  |  | 1.2 | 0.4 | 1.5 | -0.2 | 0.3 | -5.3 | -1.3 | 0.1 | -0.2 | -7 | -1.2 | -2.8 | -2.3 | -6.6 | -6.5 | -3.3 | -3 | -3.1 | -0.4 | -0.4 | -0.4 | -0.4 | 0.2 | -2.6 | -2.3 | -3.8 | -0.1 | -1.2 | auxin-responsive protein, putative; similar to auxin-responsive protein, putative [Arabidopsis thaliana] (TAIR:AT2G21220.1); similar to Auxin responsive SAUR protein [Medicago truncatula] (GB:ABE79956.1); contains InterPro domain Auxin responsive SAUR protein; (InterPro:IPR003676) |
| 874 | 256299\_at | AT1G69530 | AT-EXP1, ATEXP1, ATEXPA1, ATHEXP, EXP1 | 13 | 2.3 | 0.5 | 0.2 | -0.4 | 0 | -5.2 | 0 | 0.6 | 1.2 | -5.1 | -0.3 | -0.7 | 0.7 | -7 | -2.4 | -2.1 | -1.6 | -1.4 | 0.1 | -0.3 | -1.3 | 0 | 0.6 | -1.5 | -1.4 | -1.4 | 0.1 | -0.7 | Member of Alpha-Expansin Gene Family. Naming convention from the Expansin Working Group (Kende et al, Plant Mol Bio). Involved in the formation of nematode-induced syncytia in roots of Arabidopsis thaliana., Member of Alpha-Expansin Gene Family. Naming convention from the Expansin Working Group (Kende et al, Plant Mol Bio). Involved in the formation of nematode-induced syncytia in roots of Arabidopsis thaliana., Member of Alpha-Expansin Gene Family. Naming convention from the Expansin Working Group (Kende et al, Plant Mol Bio). Involved in the formation of nematode-induced syncytia in roots of Arabidopsis thaliana., Member of Alpha-Expansin Gene Family. Naming convention from the Expansin Working Group (Kende et al, Plant Mol Bio). Involved in the formation of nematode-induced syncytia in roots of Arabidopsis thaliana., Member of Alpha-Expansin Gene Family. Naming convention from the Expansin Working Group (Kende et al, Plant Mol Bio). Involved in the formation of nematode-induced syncytia in roots of Arabidopsis thaliana. |
| 873 | 267590\_at | AT2G39700 | ATEXP4, ATEXPA4, ATHEXP | 5 | 0.6 | -0.2 | -0.2 | -0.2 | 0.3 | -0.6 | -0.4 | -0.8 | -0.5 | -1 | -0.5 | -0.7 | 2 | -2.1 | -6.8 | -4.9 | 1.5 | -1.1 | -1.2 | -0.5 | 0 | -1.4 | 1.6 | -1.8 | -0.3 | -1.3 | -0.1 | -0.4 | putative expansin. Naming convention from the Expansin Working Group (Kende et al, 2004. Plant Mol Bio). Involved in the formation of nematode-induced syncytia in roots of Arabidopsis thaliana., putative expansin. Naming convention from the Expansin Working Group (Kende et al, 2004. Plant Mol Bio). Involved in the formation of nematode-induced syncytia in roots of Arabidopsis thaliana., putative expansin. Naming convention from the Expansin Working Group (Kende et al, 2004. Plant Mol Bio). Involved in the formation of nematode-induced syncytia in roots of Arabidopsis thaliana. |
| 872 | 262094\_at | AT1G56110 | NOP56 |  | 2.5 | 0.3 | 0 | -0.3 | -0.3 | -1.1 | -0.3 | 0.1 | 1.4 | -2.1 | 0 | -0.4 | 1 | 2.3 | -1.8 | 0.1 | 1.1 | -1 | 0.3 | -0.3 | 0.6 | 0.2 | 0.3 | 0.1 | -0.7 | -1.1 | 0 | 0.2 | NOP56-like protein |
| 871 | 252625\_at | AT3G44750 | ATHD2A, HD2A, HDA3, HDT1 | 5 | 2.5 | 0.1 | 0 | -0.6 | -0.2 | -1 | -0.3 | 1.1 | 2.4 | -3.1 | 1.2 | -0.5 | 0.8 | -0.4 | -4.6 | 0.1 | 1.6 | 0 | -0.4 | -0.7 | 0.4 | 0 | 0.3 | 0.3 | -0.8 | -1.3 | -0.2 | 0.1 | Encodes a histone deacetylase. Controls the development of adaxial/abaxial leaf polarity., Encodes a histone deacetylase. Controls the development of adaxial/abaxial leaf polarity., Encodes a histone deacetylase. Controls the development of adaxial/abaxial leaf polarity., Encodes a histone deacetylase. Controls the development of adaxial/abaxial leaf polarity. |
| 870 | 245106\_at | AT2G41650 |  |  | 1.9 | 0.5 | 0.4 | -0.2 | 0 | -0.2 | -0.2 | 0.3 | 0.9 | -2.1 | -0.1 | -0.2 | 0.4 | -0.6 | -1.1 | -0.2 | 1.2 | -0.8 | -0.3 | -0.4 | -0.1 | 0.6 | 0.6 | 0.4 | -0.6 | -0.6 | -0.2 | 0.2 | unknown protein |
| 869 | 262281\_at | AT1G68570 |  | 1 | 1.1 | -1 | 0 | 1.1 | -0.3 | 3.5 | -0.3 | 0.8 | 0.4 | 2.1 | 0.9 | 2 | -0.4 | -0.1 | 2.6 | -1.5 | 4.9 | -1.8 | -0.5 | -0.2 | -0.2 | -0.6 | -1.3 | -0.7 | -0.3 | -0.5 | 0.8 | -0.4 | proton-dependent oligopeptide transport (POT) family protein; similar to proton-dependent oligopeptide transport (POT) family protein [Arabidopsis thaliana] (TAIR:AT3G54140.1); similar to Os06g0264500 [Oryza sativa (japonica cultivar-group)] (GB:NP\_001057335.1); similar to nitrite transporter [Cucumis sativus] (GB:CAA93316.2); contains InterPro domain TGF-beta receptor, type I/II extracellular region; (InterPro:IPR000109) |
| 868 | 249063\_at | AT5G44110 | ATNAP2, ATPOP1, POP1 | 3 | 0.6 | -0.2 | 0.1 | 0.1 | -0.2 | 0.6 | -0.2 | 0 | 0.8 | -1 | 0.3 | 0.2 | -2.6 | -2 | -1.2 | -1.7 | 1.3 | -0.2 | 0.2 | -0.3 | -0.5 | 0 | -0.4 | -0.6 | -2.5 | -0.5 | -0.9 | -0.4 | Encodes a member of the NAP subfamily of ABC transporters., Encodes a member of the NAP subfamily of ABC transporters., Encodes a member of the NAP subfamily of ABC transporters. |
| 867 | 261684\_at | AT1G47400 |  |  | 1.5 | 1 | -2.1 | 1.8 | 0.6 | -1.1 | -0.6 | 0.5 | 1.5 | -3.3 | 0.4 | -2.7 | -1.2 | -2.9 | -0.6 | -3 | 6.6 | -0.3 | 0.5 | 2 | -3 | -0.6 | 0.3 | 0.4 | 1.2 | 0.6 | 1.2 | 0.2 | similar to unknown protein [Arabidopsis thaliana] (TAIR:AT1G47395.1) |
| 866 | 245296\_at | AT4G16370 | ATOPT3, OPT3 | 8 | 0.8 | 0.2 | -1 | 0.5 | -0.1 | -0.9 | 0 | 0.3 | 1 | -1 | -0.1 | -1.5 | -0.9 | -1.2 | -0.9 | -2.1 | 2 | -0.1 | 0.5 | 1.6 | 0.1 | -0.2 | -0.6 | -1.2 | 0.6 | -0.2 | 0.4 | 0.4 | oligopeptide transporter, oligopeptide transporter |
| 865 | 257062\_at | AT3G18290 | EMB2454 |  | 0 | 0.5 | -0.8 | 0.9 | -0.1 | -0.3 | 0.1 | 0.6 | 0.8 | 0 | 0.5 | -0.2 | -0.3 | -0.9 | 0.6 | -0.7 | 2.6 | 0.2 | 0.5 | 0.8 | 0.1 | -0.4 | -0.4 | 0.2 | 2.2 | 0.7 | 1.3 | 0.6 | EMB2454 (EMBRYO DEFECTIVE 2454); protein binding / zinc ion binding; similar to unknown protein [Arabidopsis thaliana] (TAIR:AT1G74770.1); similar to Zinc finger, RING-type; Zinc finger, CHY-type; RINGv [Medicago truncatula] (GB:ABE78891.1); similar to putative E3 ubiquitin ligase [Lotus japonicus] (GB:BAF38781.1); similar to Zinc finger, RING-type; Zinc finger, CHY-type; RINGv [Medicago truncatula] (GB:ABE81983.1); contains InterPro domain Hemerythrin HHE cation binding region; (InterPro:IPR012312); contains InterPro domain Zinc finger, CHY-type; (InterPro:IPR008913); contains InterPro domain Zinc finger, RING-type; (InterPro:IPR001841) |
| 864 | 248270\_at | AT5G53450 | ORG1, ORG1 | 1 | 0.6 | 0.6 | -1.4 | 0.7 | -0.1 | 0.4 | 0.3 | 0.4 | 0.4 | 2.3 | 0.1 | -0.4 | -0.6 | -0.2 | 0.3 | -0.9 | 2.3 | -0.3 | 0.3 | 1 | -1.2 | 0 | 0.1 | -0.5 | 1.5 | 0 | 0.9 | 0.3 | ORG1 (OBP3-RESPONSIVE GENE 1); kinase; similar to Proteinase inhibitor I4, serpin; Protein kinase [Medicago truncatula] (GB:ABE80838.1); similar to Os01g0655500 [Oryza sativa (japonica cultivar-group)] (GB:NP\_001043752.1); contains InterPro domain Protein kinase-like; (InterPro:IPR011009); contains InterPro domain Protein kinase; (InterPro:IPR000719) |
| 863 | 264751\_at | AT1G23020 | ATFRO3, FRO3 | 4 | 0.3 | 0.4 | -1 | 0.4 | -0.2 | -0.5 | -0.3 | 0.7 | 0.8 | 0 | 0.5 | 0 | 0.6 | -0.3 | 1.1 | -0.3 | 3.7 | -0.6 | 1.5 | 1.8 | -0.3 | -0.1 | -0.2 | -1.4 | 1.1 | 0.3 | -0.1 | 0.4 | Encodes a ferric chelate reductase whose transcription is regulated by FIT1. Expressed in the root, shoot, flower and cotyledon., Encodes a ferric chelate reductase whose transcription is regulated by FIT1. Expressed in the root, shoot, flower and cotyledon. |
| 862 | 267262\_at | AT2G22990 | SCPL8, SNG1 | 11 | -0.4 | 0.2 | -0.2 | 0.4 | 0.1 | 0.1 | 0.2 | -0.1 | -0.6 | -2 | -0.4 | -0.6 | -1 | -4.5 | 0.1 | -1.5 | -3.8 | -0.6 | -0.2 | -0.3 | -0.4 | -0.4 | -0.8 | -0.5 | -0.4 | -1.2 | -0.4 | -0.8 | sinapoylglucose:malate sinapoyltransferase. Catalyzes the formation of sinapoylmalate from sinapoylglucose. Mutants accumulate excess sinapoylglucose., sinapoylglucose:malate sinapoyltransferase. Catalyzes the formation of sinapoylmalate from sinapoylglucose. Mutants accumulate excess sinapoylglucose. |
| 861 | 252950\_at | AT4G38690 |  |  | -2.1 | 0.1 | 0.6 | 0.4 | 0 | 0.8 | 0.1 | -0.4 | -1.6 | -1.3 | -0.8 | -2 | -2.7 | -2.3 | -2 | -4.2 | -5.8 | -2.5 | -1.1 | -0.8 | -0.7 | -2 | -2.7 | -0.4 | -2.6 | -3.3 | -1.6 | -0.9 | 1-phosphatidylinositol phosphodiesterase-related; similar to 1-phosphatidylinositol phosphodiesterase-related [Arabidopsis thaliana] (TAIR:AT4G34920.1); similar to Ki1 protein [Beta vulgaris] (GB:AAO92349.1); contains InterPro domain Phosphatidylinositol-specific phospholipase C, X region; (InterPro:IPR000909) |
| 860 | 257772\_at | AT3G23080 |  |  | -2.1 | 0.7 | -1.8 | 0.8 | 0 | -0.6 | 0 | 0 | -1.1 | -2.9 | -0.3 | -0.2 | -1.6 | -3.3 | -1.3 | -3 | -2.1 | -1.8 | -0.2 | -0.4 | -0.1 | -0.9 | -0.6 | 0.6 | -1 | -1.3 | -0.3 | -0.6 | similar to unknown protein [Arabidopsis thaliana] (TAIR:AT4G14500.1); similar to putative membrane related protein CP5 [Oryza sativa (japonica cultivar-group)] (GB:BAC83004.1); contains InterPro domain Lipid-binding START; (InterPro:IPR002913), similar to unknown protein [Arabidopsis thaliana] (TAIR:AT4G14500.1); similar to putative membrane related protein CP5 [Oryza sativa (japonica cultivar-group)] (GB:BAC83004.1); similar to Os07g0185200 [Oryza sativa (japonica cultivar-group)] (GB:NP\_001059064.1); contains InterPro domain Lipid-binding START; (InterPro:IPR002913) |
| 859 | 256020\_at | AT1G58290 | HEMA1 | 9 | -1.4 | 0 | 0.3 | 0 | 0 | -0.3 | -0.2 | -0.4 | -0.7 | -2.2 | -0.4 | -0.4 | -1 | -2.5 | -0.9 | -1.4 | -2.7 | -0.3 | -0.3 | -0.5 | 0.6 | -0.5 | -0.6 | 0.3 | -1.3 | -1.2 | -0.5 | -0.8 | Encodes a protein with glutamyl-tRNA reductase (GluTR) activity, catalyzing the NADPH-dependent reduction of Glu-tRNA(Glu) to glutamate 1-semialdehyde (GSA) with the release of free tRNA(Glu). It is involved in the early steps of chlorophyll biosynthesis. |
| 858 | 262309\_at | AT1G70820 |  | 1 | -1.3 | -0.6 | -0.5 | -0.3 | 0 | -0.3 | 0.1 | -0.3 | -1 | -3.1 | 0 | -1.4 | -1.3 | -2.5 | -1.6 | -1.6 | -2.3 | -0.5 | -0.3 | -0.1 | -0.7 | -0.4 | -0.5 | 1.4 | -1.6 | -1.2 | -0.3 | -1.1 | phosphoglucomutase, putative / glucose phosphomutase, putative; similar to phosphoglucosamine mutase family protein [Arabidopsis thaliana] (TAIR:AT5G17530.2); similar to Os06g0476200 [Oryza sativa (japonica cultivar-group)] (GB:NP\_001057639.1); similar to phosphoglucomutase [Spinacia oleracea] (GB:CAA53507.1); contains InterPro domain Phosphoglucomutase/phosphomannomutase alpha/beta/alpha domain II; (InterPro:IPR005845); contains InterPro domain Phosphoglucomutase/phosphomannomutase alpha/beta/alpha domain I; (InterPro:IPR005844); contains InterPro domain Phosphoglucomutase/phosphomannomutase alpha/beta/alpha domain III; (InterPro:IPR005846); contains InterPro domain Phosphoglucomutase/phosphomannomutase; (InterPro:IPR005841) |
| 857 | 247899\_at | AT5G57345 |  |  | -0.9 | -0.3 | -0.3 | 0.3 | -0.2 | 0.4 | 0 | 0.1 | -0.5 | -1.6 | -0.2 | -0.9 | -1 | -2.5 | -0.9 | -1.8 | -1.3 | -0.5 | -0.3 | -0.1 | -0.4 | -0.1 | -0.1 | 0.3 | -0.4 | -1 | 0.2 | 0.2 | similar to unknown protein [Arabidopsis thaliana] (TAIR:AT1G54520.1); similar to conserved hypothetical protein [Medicago truncatula] (GB:ABE83998.1); contains domain N-terminal nucleophile aminohydrolases (Ntn hydrolases) (SSF56235) |
| 856 | 260547\_at | AT2G43550 |  | 1 | 0.6 | -0.1 | -0.4 | 0.6 | 0.4 | -0.2 | -0.2 | -0.6 | -1.6 | -3.5 | -0.1 | -1.1 | -3.3 | -3.9 | -2 | -2 | -1.5 | 0.3 | -1.2 | -0.5 | 0 | -0.3 | -0.9 | 3.5 | -0.2 | -0.4 | 0.2 | -1 | Encodes a defensin-like (DEFL) family protein. |
| 855 | 255298\_at | AT4G04840 |  |  | 0.1 | -0.2 | 0.1 | 0.6 | 0.2 | -1.5 | -0.4 | -0.5 | -1.7 | -7.5 | -1 | -3.8 | -3.1 | -7 | -2 | -3.7 | -2.5 | -0.3 | -0.7 | -1.4 | -0.8 | -1.7 | -1.7 | 2.8 | -2.7 | -0.9 | -1 | -1.3 | methionine sulfoxide reductase domain-containing protein / SeIR domain-containing protein; similar to methionine sulfoxide reductase domain-containing protein / SeIR domain-containing protein [Arabidopsis thaliana] (TAIR:AT4G21850.1); similar to Mss4-like [Medicago truncatula] (GB:ABE84787.1); contains InterPro domain Methionine sulfoxide reductase B; (InterPro:IPR002579); contains InterPro domain Mss4-like; (InterPro:IPR011057) |
| 854 | 261518\_at | AT1G71695 |  | 1 | 0 | -0.2 | -0.5 | 0.1 | -0.3 | 0 | 0.4 | -0.4 | -1.5 | 1.7 | -0.5 | -5.6 | -4.2 | -3.5 | -5.6 | -1.9 | -1.1 | -1.3 | -0.4 | -0.2 | -0.5 | -0.2 | -0.4 | -0.1 | -0.5 | -1.5 | 0 | 0.2 | peroxidase 12 (PER12) (P12) (PRXR6); Identical to Peroxidase 12 precursor (EC 1.11.1.7) (Atperox P12) (PRXR6) (ATP4a) (PER12) [Arabidopsis Thaliana] (GB:Q96520;GB:Q43734); similar to peroxidase, putative [Arabidopsis thaliana] (TAIR:AT4G08770.1); similar to peroxidase [Nicotiana tabacum] (GB:AAK52084.1); contains InterPro domain Haem peroxidase; (InterPro:IPR010255); contains InterPro domain Haem peroxidase, plant/fungal/bacterial; (InterPro:IPR002016); contains InterPro domain Plant peroxidase; (InterPro:IPR000823) |
| 853 | 252168\_at | AT3G50440 | ATMES10, MES10 | 1 | -0.3 | 0.2 | -1.1 | 0.3 | -0.1 | -0.9 | 0 | -1 | -1.3 | 2.1 | -1.1 | -1.9 | -1.9 | -1.2 | -1.9 | -1.1 | 1.5 | -1 | -0.8 | -0.4 | -0.5 | -0.5 | -1.3 | 0.5 | -0.8 | -1.4 | -0.4 | -0.6 | hydrolase; similar to esterase, putative [Arabidopsis thaliana] (TAIR:AT2G23620.1); similar to salicylic acid-binding protein 2 [Nicotiana tabacum] (GB:AAR87711.1); contains InterPro domain Esterase/lipase/thioesterase; (InterPro:IPR000379); contains InterPro domain Alpha/beta hydrolase fold-1; (InterPro:IPR000073), hydrolase; similar to esterase, putative [Arabidopsis thaliana] (TAIR:AT2G23620.1); similar to salicylic acid-binding protein 2 [Nicotiana tabacum] (GB:AAR87711.1); contains InterPro domain Esterase/lipase/thioesterase; (InterPro:IPR000379); contains InterPro domain Alpha/beta hydrolase fold-1; (InterPro:IPR000073) |
| 852 | 262682\_at | AT1G75900 |  | 2 | -0.2 | 1 | 1.3 | 1.8 | -0.1 | 2.2 | -3.4 | -0.6 | -2 | 2 | -1 | -1.3 | -3.1 | -3.2 | -5.8 | -4.1 | 3.3 | -1.6 | -0.4 | -0.5 | -0.8 | -2 | -1.6 | 0.2 | -1.8 | -1.9 | -0.2 | -0.3 | family II extracellular lipase 3 (EXL3); similar to family II extracellular lipase 1 (EXL1) [Arabidopsis thaliana] (TAIR:AT1G75880.2); similar to Lipolytic enzyme, G-D-S-L [Medicago truncatula] (GB:ABE88162.1); contains InterPro domain Lipolytic enzyme, G-D-S-L; (InterPro:IPR001087); contains InterPro domain 5' (InterPro:IPR006179); contains InterPro domain Lipolytic enzyme, G-D-S-L, active site; (InterPro:IPR008265) |
| 851 | 260490\_at | AT1G51500 | ABCG12, CER5, D3, WBC12 | 4 | 0 | 0.2 | 0.3 | 0.5 | 0 | 1.5 | -0.2 | -0.3 | -0.9 | 0.7 | -0.4 | -2.1 | -1.8 | -1.6 | -2 | -1.7 | -1.6 | -1.5 | -0.4 | 0.4 | 0 | -0.9 | -0.7 | -0.5 | 0.3 | -1.3 | -0.3 | -0.1 | Encodes an ABC transporter involved in cuticular wax biosynthesis. Lines carrying recessive mutations in this locus have weakly glaucous stem surface, and relative elevated secondary alcohols and ketones., Encodes an ABC transporter involved in cuticular wax biosynthesis. Lines carrying recessive mutations in this locus have weakly glaucous stem surface, and relative elevated secondary alcohols and ketones., Encodes an ABC transporter involved in cuticular wax biosynthesis. Lines carrying recessive mutations in this locus have weakly glaucous stem surface, and relative elevated secondary alcohols and ketones., Encodes an ABC transporter involved in cuticular wax biosynthesis. Lines carrying recessive mutations in this locus have weakly glaucous stem surface, and relative elevated secondary alcohols and ketones. |
| 850 | 259788\_at | AT1G29670 |  |  | 0.8 | 0 | 0.9 | 0.3 | -0.1 | 0.8 | -0.2 | -0.4 | -1.5 | -0.3 | -1 | -3.9 | -3.6 | -5 | -9.1 | -5.2 | -1.9 | -2.4 | -0.7 | 0.3 | -0.5 | -0.6 | -0.4 | -1 | 0.6 | -2 | -0.5 | -0.4 | GDSL-motif lipase/hydrolase family protein; similar to GDSL-motif lipase/hydrolase family protein [Arabidopsis thaliana] (TAIR:AT1G29660.1); similar to GDSL-lipase 1 [Capsicum annuum] (GB:AAZ23955.1); contains InterPro domain Lipolytic enzyme, G-D-S-L; (InterPro:IPR001087) |
| 849 | 260267\_at | AT1G68530 | CER6, CUT1, G2, KCS6, POP1 | 20 | 0.4 | 0.1 | -0.3 | 0.3 | 0 | 1.4 | -0.6 | -0.1 | -0.9 | 1.6 | -0.1 | -1.3 | -1.7 | -0.5 | -1.8 | -1.5 | -1.2 | -1.8 | -0.1 | 0.3 | 0.4 | -0.7 | -1.3 | 0.7 | 0 | -0.6 | -0.2 | -0.1 | involved in wax biosynthesis; required for elongation of C24 very-long-chain fatty acids, involved in wax biosynthesis; required for elongation of C24 very-long-chain fatty acids, involved in wax biosynthesis; required for elongation of C24 very-long-chain fatty acids, involved in wax biosynthesis; required for elongation of C24 very-long-chain fatty acids, involved in wax biosynthesis; required for elongation of C24 very-long-chain fatty acids |
| 848 | 261118\_at | AT1G75460 |  | 1 | 0.5 | -0.1 | -1.5 | 0.4 | -0.2 | 0.1 | 0.3 | -0.2 | -0.9 | -0.8 | -0.3 | -1.8 | -1.3 | -1.6 | -2.1 | -2.1 | -2.7 | -1.2 | -0.3 | -0.2 | 0 | -0.4 | -0.6 | 0.1 | -0.9 | -2 | -0.5 | -0.7 | ATP-dependent protease La (LON) domain-containing protein; similar to ATP-dependent protease La (LON) domain-containing protein [Arabidopsis thaliana] (TAIR:AT1G19740.1); similar to Os07g0509700 [Oryza sativa (japonica cultivar-group)] (GB:NP\_001059749.1); similar to hypothetical protein all4335 [Nostoc sp. PCC 7120] (GB:NP\_488375.1); contains InterPro domain Peptidase S16, lon N-terminal; (InterPro:IPR003111) |
| 847 | 244998\_at | ATCG00180 | RPOC1 | 5 | 0.1 | 0.3 | -0.1 | 0.1 | 0.2 | 0 | 0.1 | 0 | -0.3 | -0.2 | 0.1 | -1.4 | -1.2 | -1 | -0.6 | -0.9 | -1.6 | -1 | 0.1 | 0.3 | 0.1 | 0 | 0 | -0.3 | 0 | -0.3 | 0 | -0.1 | RNA polymerase beta' subunit-1 |
| 846 | 258113\_at | AT3G14650 | CYP72A11 |  | 0.1 | -0.2 | -0.4 | -0.1 | -0.2 | -1 | -0.1 | -0.1 | -0.4 | 0.1 | -0.2 | -1.5 | -1.4 | -0.4 | -1.9 | -1.7 | -1.5 | -0.7 | -0.2 | -0.1 | 0.1 | -0.4 | -0.9 | 0 | -0.9 | -1.7 | -0.5 | -0.5 | putative cytochrome P450 |
| 845 | 247478\_at | AT5G62360 |  |  | 0.9 | 0.3 | -3.8 | -0.1 | 0.4 | -5.1 | -0.4 | -0.3 | -0.8 | 0.2 | -1.6 | -3.1 | -2.6 | -2.3 | -5.9 | -1.6 | -5.2 | -2.6 | 0 | 0.1 | 0.3 | -0.7 | -2.9 | 2.2 | -0.7 | -1.5 | -1 | -1.9 | invertase/pectin methylesterase inhibitor family protein; similar to invertase/pectin methylesterase inhibitor family protein / DC 1.2 homolog (FL5-2I22) [Arabidopsis thaliana] (TAIR:AT5G62350.1); similar to pectin methylesterase inhibitor isoform [Solanum phureja] (GB:ABG49144.1); contains InterPro domain Plant invertase/pectin methylesterase inhibitor; (InterPro:IPR007186); contains InterPro domain Pectinesterase inhibitor; (InterPro:IPR006501) |
| 844 | 256060\_at | AT1G07050 |  |  | 3.6 | -0.1 | -1.6 | 0.5 | 0.4 | -2.6 | -0.2 | -0.1 | -0.5 | -0.9 | 0 | -3.8 | -4.4 | 2 | -7.3 | 0.5 | -1.7 | -3.1 | -0.4 | -0.5 | -0.7 | -1.3 | -0.7 | 1.3 | -0.9 | -1.8 | 0.1 | -1.1 | CONSTANS-like protein-related; similar to CIL [Arabidopsis thaliana] (TAIR:AT4G25990.1); similar to putative stress resistance-related protein [Oryza sativa (japonica cultivar-group)] (GB:ABF00989.1); similar to Os06g0264200 [Oryza sativa (japonica cultivar-group)] (GB:NP\_001057333.1); contains InterPro domain CCT; (InterPro:IPR010402) |
| 843 | 263034\_at | AT1G24020 | MLP423 | 1 | 0.4 | -0.2 | -0.4 | 0.2 | -0.1 | -0.5 | -0.3 | -0.9 | -1.6 | -2.7 | -0.8 | -2.3 | -3.2 | -3.6 | -3.2 | -3.8 | 1.4 | 0.7 | -0.7 | -0.1 | -0.3 | 0 | -0.3 | 0.2 | -0.7 | -0.6 | 0.2 | 0 | Bet v I allergen family protein; Identical to MLP-like protein 423 (MLP423) [Arabidopsis Thaliana] (GB:Q93VR4;GB:Q8L9L8;GB:Q9LR93); similar to MLP34 (MLP-LIKE PROTEIN 34) [Arabidopsis thaliana] (TAIR:AT1G70850.3); similar to Bet v I allergen [Medicago truncatula] (GB:ABE83209.1); contains InterPro domain Bet v I allergen; (InterPro:IPR000916) |
| 842 | 250533\_at | AT5G08640 | FLS | 13 | 0.4 | -0.5 | -0.2 | 0.4 | -0.7 | 0 | -0.3 | -1.6 | -1.8 | -1.7 | -1.5 | -2.6 | -2.9 | -2.5 | -0.5 | -1.3 | 2.6 | 0.4 | 0.3 | -0.1 | -0.3 | -0.6 | -0.9 | -1.3 | -2.8 | -0.9 | -0.5 | 0.2 | Encodes a flavonol synthase that catalyzes formation of flavonols from dihydroflavonols. |
| 841 | 257809\_at | AT3G27060 | TSO2 | 2 | 0.9 | 0.3 | -0.1 | -0.1 | 0 | -0.7 | -0.2 | -0.1 | 0.9 | -3.2 | -0.2 | -1.2 | -1.6 | -3.6 | -1.9 | -0.5 | 1.2 | -0.7 | 0.6 | 3 | 0.1 | 0 | 0.1 | -0.3 | -0.7 | -0.7 | -0.5 | 0 | Encodes one of the 3 ribonucleotide reductase (RNR) small subunit genes. TSO2 transcription occurs predominantly at the S-phase of the cell cycle and its expression pattern is consistent with its role in dNDP biosynthesis during DNA replication in actively dividing cells. Critical for cell cycle progression, DNA damage repair and plant development. |
| 840 | 250517\_at | AT5G08260 | scpl35 | 1 | -0.1 | -0.2 | 0.1 | -0.3 | -0.3 | -0.4 | 0.1 | 0 | -0.5 | -1.5 | 0 | -3.7 | -3.3 | -1.9 | -5 | -0.4 | 3.6 | -1.2 | 0.4 | 0.8 | 0.7 | 0.1 | 0.1 | -0.2 | -0.4 | -1 | 0 | -0.2 | SCPL35 (serine carboxypeptidase-like 35); serine carboxypeptidase; similar to SCPL34, serine carboxypeptidase [Arabidopsis thaliana] (TAIR:AT5G23210.1); similar to OSIGBa0153E02-OSIGBa0093I20.21 [Oryza sativa (indica cultivar-group)] (GB:CAH67892.1); similar to serine carboxypeptidase (carboxypeptidase D), putative [Musa acuminata] (GB:ABF70080.1); similar to OSJNBa0038O10.8 [Oryza sativa (japonica cultivar-group)] (GB:CAE05642.2); contains InterPro domain Esterase/lipase/thioesterase; (InterPro:IPR000379); contains InterPro domain Peptidase S10, serine carboxypeptidase; (InterPro:IPR001563) |
| 839 | 252607\_at | AT3G44990 | ATXTR8, XTR8 |  | 1.3 | -0.2 | 0.2 | -0.1 | -0.2 | -2.8 | -0.1 | -2.4 | -2.2 | -5.9 | -2.6 | -4.7 | -4.3 | -4.7 | -8.4 | -1.4 | 1.8 | -2.9 | -1.5 | -0.5 | -0.1 | 0.5 | -0.4 | -1.1 | -2.3 | -3.8 | -0.1 | -0.7 | xyloglucan endo-transglycosylase, xyloglucan endo-transglycosylase |
| 838 | 266123\_at | AT2G45180 |  | 1 | 0.1 | -0.2 | -0.1 | 0.1 | -0.1 | -0.7 | 0.1 | -0.1 | -0.3 | -2.8 | -0.3 | -0.8 | -0.2 | -1.6 | -2.8 | -1.9 | -1.8 | -0.7 | 0 | -0.3 | -0.3 | -0.2 | -0.2 | -0.4 | 0.1 | -0.7 | 0.1 | 0.2 | protease inhibitor/seed storage/lipid transfer protein (LTP) family protein; similar to protease inhibitor/seed storage/lipid transfer protein (LTP) family protein [Arabidopsis thaliana] (TAIR:AT1G62510.1); similar to 14 kDa polypeptide [Catharanthus roseus] (GB:CAA81526.1); contains InterPro domain Plant lipid transfer/seed storage/trypsin-alpha amylase inhibitor; (InterPro:IPR003612) |
| 837 | 259892\_at | AT1G72610 | ATGER1, GER1, GLP1 | 2 | 0.1 | -0.3 | -0.2 | -0.4 | -0.2 | -1.9 | 0.1 | 0.1 | 0 | -5.8 | 0 | -1.4 | -1.4 | -1.6 | -9.4 | -2.8 | -2.3 | -1.3 | 0.1 | 0.1 | 0 | -0.5 | -0.5 | -1.1 | 0 | -0.8 | 0.1 | 0.1 | germin-like protein (GLP1), germin-like protein (GLP1), germin-like protein (GLP1) |
| 836 | 265894\_at | AT2G15050 | LTP | 2 | 0.7 | -0.5 | -0.3 | 0.6 | -0.2 | -2 | 0.1 | -0.3 | -1.2 | -2.6 | -0.9 | -2.1 | -2.2 | -2.1 | -4.8 | -1.9 | -0.9 | -1.7 | -0.5 | 0.1 | 0.1 | -0.7 | -2.2 | -0.1 | 0.2 | -1.4 | 0.2 | 0.4 | LTP; lipid binding; similar to LP1 (nonspecific lipid transfer protein 1) [Arabidopsis thaliana] (TAIR:AT2G38540.1); similar to Nonspecific lipid-transfer protein A precursor (LTP A) (Wax-associated protein 9A) (GB:Q42641); contains InterPro domain Plant lipid transfer protein/Par allergen; (InterPro:IPR000528); contains InterPro domain Plant lipid transfer/seed storage/trypsin-alpha amylase inhibitor; (InterPro:IPR003612) |
| 835 | 267367\_at | AT2G44210 |  |  | -0.2 | -0.7 | 0.1 | 0.3 | 0 | -1.1 | -0.3 | -0.5 | -1.2 | -2.9 | -0.6 | -1 | -0.6 | -1.4 | -1.1 | -1 | -1.2 | -0.6 | 0.7 | 0.6 | 0.7 | -0.4 | -0.6 | -0.2 | -0.2 | -0.9 | 0.2 | 1.1 | similar to unknown protein [Arabidopsis thaliana] (TAIR:AT1G55360.1); similar to Protein of unknown function DUF239, plant [Medicago truncatula] (GB:ABE82132.1); contains InterPro domain Protein of unknown function DUF239, plant; (InterPro:IPR004314), similar to unknown protein [Arabidopsis thaliana] (TAIR:AT1G55360.1); similar to unknown protein [Arabidopsis thaliana] (TAIR:AT3G13510.1); similar to Protein of unknown function DUF239, plant [Medicago truncatula] (GB:ABE82132.1); contains InterPro domain Protein of unknown function DUF239, plant; (InterPro:IPR004314) |
| 834 | 247444\_at | AT5G62630 | HIPL2 | 1 | 0.5 | 0.1 | -0.4 | 0.5 | -0.3 | -3.1 | -0.4 | 0.1 | -0.2 | -2.2 | -0.1 | -1.6 | 1.8 | -2 | -6.2 | -1.7 | 1.1 | -0.9 | 1.4 | 1.2 | -0.1 | -0.5 | -1.4 | -1.1 | 0.8 | -0.7 | -0.2 | 1.2 | HIPL2 (HIPL2 PROTEIN PRECURSOR); Identical to HIPL2 protein precursor (HIPL2) [Arabidopsis Thaliana] (GB:Q94F08;GB:Q9LV15); similar to unknown protein [Arabidopsis thaliana] (TAIR:AT5G39970.1); similar to unknown protein [Arabidopsis thaliana] (TAIR:AT1G74790.1); similar to hypothetical protien [Hordeum vulgare subsp. vulgare] (GB:AAV49993.1); similar to glucose/sorbosone dehydrogenases-like protein [Oryza sativa (japonica cultivar-group)] (GB:BAD30151.1); similar to unnamed protein product [Triticum tur (GB:CAJ13560.1); contains InterPro domain Soluble quinoprotein glucose dehydrogenase; (InterPro:IPR011041) |
| 833 | 266672\_at | AT2G29650 | ANTR1, PHT4;1, ANTR1, PHT4;1 | 4 | -0.4 | -0.3 | -0.5 | 0.1 | -0.3 | 0 | 0 | -2.1 | -0.4 | -0.6 | 0.6 | -0.9 | -0.9 | -1.7 | -0.7 | -1.6 | -1.9 | -0.3 | -0.7 | 0 | -0.4 | -0.1 | -0.5 | -0.2 | -0.9 | -1 | 0.1 | -0.2 | inorganic phosphate transporter, putative; similar to ANTR2 (anion transporter 2), organic anion transporter [Arabidopsis thaliana] (TAIR:AT4G00370.1); similar to Na+/Pi transporter [Thellun (GB:ABK51380.1); contains InterPro domain Major facilitator superfamily; (InterPro:IPR007114); contains InterPro domain Major facilitator superfamily MFS\_1; (InterPro:IPR011701), inorganic phosphate transporter, putative; similar to ANTR2 (anion transporter 2), organic anion transporter [Arabidopsis thaliana] (TAIR:AT4G00370.1); similar to Na+/Pi transporter [Thellun (GB:ABK51380.1); contains InterPro domain Major facilitator superfamily; (InterPro:IPR007114); contains InterPro domain Major facilitator superfamily MFS\_1; (InterPro:IPR011701), inorganic phosphate transporter, putative; similar to ANTR2 (anion transporter 2), organic anion transporter [Arabidopsis thaliana] (TAIR:AT4G00370.1); similar to Na+/Pi transporter [Thellun (GB:ABK51380.1); similar to Major facilitator superfamily MFS\_1 [Medicago truncatula] (GB:ABE91392.1); similar to Os01g0279700 [Oryza sativa (japonica cultivar-group)] (GB:NP\_001042749.1); contains InterPro domain Major facilitator superfamily; (InterPro:IPR007114); contains InterPro domain Major facilitator superfamily MFS\_1; (InterPro:IPR011701), inorganic phosphate transporter, putative; similar to ANTR2 (anion transporter 2), organic anion transporter [Arabidopsis thaliana] (TAIR:AT4G00370.1); similar to Na+/Pi transporter [Thellun (GB:ABK51380.1); similar to Major facilitator superfamily MFS\_1 [Medicago truncatula] (GB:ABE91392.1); similar to Os01g0279700 [Oryza sativa (japonica cultivar-group)] (GB:NP\_001042749.1); contains InterPro domain Major facilitator superfamily; (InterPro:IPR007114); contains InterPro domain Major facilitator superfamily MFS\_1; (InterPro:IPR011701) |
| 832 | 249355\_at | AT5G40500 |  | 1 | -0.6 | -0.2 | -0.4 | 0.6 | 0.3 | 1.1 | 0 | 0 | -0.5 | -0.4 | 0.3 | -0.6 | -0.7 | -1.8 | -0.3 | -1.3 | -1.9 | 0.1 | -0.9 | -0.6 | -0.5 | -0.2 | -0.1 | -0.6 | -0.4 | -1.1 | 0 | 1 | similar to Os04g0482900 [Oryza sativa (japonica cultivar-group)] (GB:NP\_001053120.1); similar to unnamed protein product [Ostreococcus tauri] (GB:CAL55654.1) |
| 831 | 252412\_at | AT3G47295 |  |  | -0.2 | -0.3 | -1.5 | 0.5 | -0.2 | 2.4 | 0.1 | -0.8 | -1 | -1.4 | -0.4 | -2.1 | -2.4 | -1.6 | -2.6 | -1.5 | -2.3 | 0 | -1.2 | 0 | -0.6 | -1.5 | -0.5 | -0.1 | -0.3 | -0.1 | -0.2 | 0 | similar to unknown protein [Arabidopsis thaliana] (TAIR:AT5G58650.1) |
| 830 | 259880\_at | AT1G76730 |  | 1 | -0.6 | 0 | -0.4 | 0.1 | -0.3 | 0.1 | 0.2 | 0 | -0.4 | -0.8 | -0.1 | -1.5 | -0.8 | -0.9 | -2 | -1.4 | -1.3 | -0.4 | -0.5 | 0 | -0.3 | 0 | -0.3 | -0.3 | 0 | -0.8 | 0.3 | 0 | 5-formyltetrahydrofolate cyclo-ligase family protein; similar to hypothetical protein LOC509897 [Bos taurus] (GB:NP\_001039507.1); similar to Os12g0168000 [Oryza sativa (japonica cultivar-group)] (GB:NP\_001066251.1); contains InterPro domain 5-formyltetrahydrofolate cyclo-ligase; (InterPro:IPR002698) |
| 829 | 258398\_at | AT3G15360 | ATHM4, ATM4, TRX-M4 | 5 | -0.5 | -0.2 | -0.2 | 0 | -0.2 | -0.1 | 0.1 | 0 | -0.2 | -0.6 | 0 | -1.1 | -0.5 | -0.5 | -1.6 | -1.3 | -1.1 | -0.8 | -0.2 | -0.1 | 0 | -0.1 | 0 | -0.3 | 0 | -0.3 | 0 | -0.2 | encodes a prokaryotic thioredoxin, encodes a prokaryotic thioredoxin, encodes a prokaryotic thioredoxin |
| 828 | 251497\_at | AT3G59060 | PIF5, PIL6 | 8 | -0.8 | -0.2 | -1.3 | 0.2 | -0.4 | -0.1 | 0.3 | 0 | -0.3 | -0.5 | 0 | -1 | -0.2 | -0.7 | -1.2 | -1.1 | -1.8 | -1.6 | -0.2 | 0 | -0.4 | -0.3 | -0.2 | -0.7 | 0.4 | -0.5 | 0.3 | 0.1 | encodes a novel Myc-related bHLH transcription factor, which physically associated with APRR1/TOC1 and is a member of PIF3 transcription factor family., encodes a novel Myc-related bHLH transcription factor, which physically associated with APRR1/TOC1 and is a member of PIF3 transcription factor family. |
| 827 | 260837\_at | AT1G43670 |  |  | -1.2 | -0.6 | -0.2 | 0.1 | -0.2 | -0.3 | -0.1 | 0.4 | -0.4 | -2.8 | 0.2 | -1.2 | -1 | -1.5 | -2.1 | -2.3 | -1.8 | -0.8 | -0.2 | -0.2 | -0.6 | -0.3 | -0.5 | -0.5 | -0.1 | -1 | 0.1 | -0.2 | fructose-1,6-bisphosphatase, putative / D-fructose-1,6-bisphosphate 1-phosphohydrolase, putative / FBPase, putative; Identical to Fructose-1,6-bisphosphatase, cytosolic (EC 3.1.3.11) (D-fructose-1,6- bisphosphate 1-phosphohydrolase) (FBPase) [Arabidopsis Thaliana] (GB:Q9MA79); similar to fructose-1,6-bisphosphatase, putative / D-fructose-1,6-bisphosphate 1-phosphohydrolase, putative / FBPase, putative [Arabidopsis thaliana] (TAIR:AT3G54050.1); similar to fructose 1,6-bisphosphatase [Brassica rapa subsp. pekinensis] (GB:AAZ86538.1); similar to Fructose-1,6-bisphosphatase, cytosolic (D-fructose-1,6-bisphosphate 1-phosphohydrolase) (FBPase) (GB:P46267); contains InterPro domain Inositol phosphatase/fructose-1,6-bisphosphatase; (InterPro:IPR000146) |
| 826 | 253337\_at | AT4G33470 | hda14 | 2 | -0.6 | -0.5 | -0.3 | -0.1 | -0.1 | -0.4 | -0.3 | 0.2 | -0.6 | -2.3 | 0 | -2.4 | -2.5 | -1.3 | -3.3 | -2.4 | -1.6 | -2.5 | -0.5 | 0.1 | -0.3 | -0.3 | -0.4 | -0.6 | -0.2 | -1.2 | -0.1 | 0 | HDA14 (histone deacetylase 14); histone deacetylase; similar to HDA15 (histone deacetylase 15), histone deacetylase [Arabidopsis thaliana] (TAIR:AT3G18520.2); similar to Os12g0182700 [Oryza sativa (japonica cultivar-group)] (GB:NP\_001066322.1); similar to Histone deacetylase complex, catalytic component RPD3 (ISS) [Ostreococcus tauri] (GB:CAL55176.1); contains InterPro domain Histone deacetylase superfamily; (InterPro:IPR000286) |
| 825 | 267247\_at | AT2G30170 |  | 1 | -0.7 | -0.3 | -0.2 | 0.1 | 0 | -0.2 | 0 | 0.1 | -0.5 | -0.9 | -0.3 | -2 | -1.3 | -1.1 | -1.6 | -1.9 | -2.1 | -1.7 | -0.5 | -0.3 | 0 | -0.1 | -0.2 | -0.2 | 0.1 | -0.6 | 0.2 | 0.1 | catalytic; similar to 5-azacytidine resistance protein -related [Arabidopsis thaliana] (TAIR:AT5G66720.2); similar to Os01g0164600 [Oryza sativa (japonica cultivar-group)] (GB:NP\_001042103.1); similar to putative senescence-associated protein [Pisum sativum] (GB:BAB33413.1); contains InterPro domain Protein phosphatase 2C-like; (InterPro:IPR001932) |
| 824 | 259296\_at | AT3G05350 |  | 1 | -0.6 | -0.2 | -0.2 | 0.4 | -0.1 | 0 | 0.2 | 0 | -0.5 | -0.3 | -0.1 | -1.2 | -1.3 | -0.4 | -0.9 | 0 | -0.8 | -0.4 | -0.2 | -0.2 | -0.1 | 0 | -0.3 | -0.1 | 0.3 | -0.2 | 0.4 | 0.2 | aminopeptidase; similar to ATAPP1 (aminopeptidase P1) [Arabidopsis thaliana] (TAIR:AT4G36760.1); similar to Os07g0205700 [Oryza sativa (japonica cultivar-group)] (GB:NP\_001059149.1); similar to Peptidase M24; Peptidase aspartic, active site [Medicago truncatula] (GB:ABE90680.1); contains InterPro domain Peptidase M24; (InterPro:IPR000994) |
| 823 | 262988\_at | AT1G23310 | AOAT1, GGT1 | 5 | -0.6 | -0.2 | 0 | 0 | 0 | 0.1 | 0.2 | 0 | 0 | -0.5 | -0.2 | -0.5 | -0.1 | -0.5 | -0.4 | -0.5 | -1.2 | -0.1 | -0.1 | -0.1 | -0.1 | 0 | -0.2 | -0.3 | 0.1 | -0.1 | 0 | 0 | Identified by cloning the gene that corresponded to a purified protein having glyoxylate aminotransferase activity. Localized to the peroxisome and thought to be involved in photorespiration/ metabolic salvage pathway., Identified by cloning the gene that corresponded to a purified protein having glyoxylate aminotransferase activity. Localized to the peroxisome and thought to be involved in photorespiration/ metabolic salvage pathway. |
| 822 | 249996\_at | AT5G18600 |  |  | 0 | -0.6 | -0.6 | -0.7 | 0.1 | 0.7 | 0.9 | -0.5 | -0.1 | -1.9 | -0.3 | -1.2 | -1.6 | -3.8 | -0.9 | -1.1 | -4 | -1.4 | 0 | -0.3 | -0.8 | 0.2 | 0.1 | -0.7 | 0.2 | -0.2 | -0.6 | -0.5 | glutaredoxin family protein; Identical to Monothiol glutaredoxin-S2 (AtGrxS2) (GRXS2) [Arabidopsis Thaliana] (GB:Q8L8Z8); similar to glutaredoxin family protein [Arabidopsis thaliana] (TAIR:AT4G15680.1); similar to Os01g0368900 [Oryza sativa (japonica cultivar-group)] (GB:NP\_001043047.1); similar to Glutaredoxin-like family protein [Oryza sativa (japonica cultivar-group)] (GB:ABA99336.1); contains InterPro domain Thioredoxin-like fold; (InterPro:IPR012336); contains InterPro domain Glutaredoxin; (InterPro:IPR002109); contains InterPro domain Thioredoxin fold; (InterPro:IPR012335); contains InterPro domain Glutaredoxin-like, plant II; (InterPro:IPR011905) |
| 821 | 253335\_at | AT4G33500 |  | 1 | -0.7 | 0 | -0.2 | -0.3 | 0 | 0 | 0 | -0.1 | -0.6 | -2.2 | -0.4 | -1.3 | -0.9 | -1 | -2.1 | -1.3 | -1.8 | -0.9 | -0.5 | -0.2 | -0.5 | 0.1 | -0.2 | -0.2 | -0.3 | -1.2 | -0.2 | -0.3 | protein phosphatase 2C-related / PP2C-related; similar to catalytic [Arabidopsis thaliana] (TAIR:AT4G16580.1); similar to Os03g0192500 [Oryza sativa (japonica cultivar-group)] (GB:NP\_001049240.1); similar to Protein phosphatase 2C containing protein, expressed [Oryza sativa (japonica cultivar-group)] (GB:ABF94415.1); similar to Protein phosphatase 2C containing protein, expressed [Oryza sativa (japonica cultivar-group)] (GB:ABF94416.1); contains InterPro domain Protein phosphatase 2C-like; (InterPro:IPR001932) |
| 820 | 246454\_at | AT5G16710 | DHAR3 | 3 | -0.5 | -0.1 | -0.1 | -0.1 | 0 | 0 | 0 | 0 | -0.3 | -1.7 | -0.3 | -1.8 | -1.4 | -1.5 | -2.4 | -1.6 | -1.1 | -0.7 | -0.5 | -0.4 | -0.6 | -0.1 | 0 | -0.1 | -0.3 | -0.5 | -0.1 | -0.2 | The protein undergoes thiolation following treatment with the oxidant tert-butylhydroperoxide. |
| 819 | 251230\_at |  |  |  | -0.8 | -0.1 | -0.2 | 0 | 0.3 | 0.2 | -0.3 | -0.1 | -1.1 | -1.6 | -0.7 | -1.9 | -2 | -2.2 | -2.6 | -1.6 | -1.9 | -1.8 | -1.1 | -0.2 | -0.8 | 0.1 | -1 | -0.8 | -0.1 | -1.8 | 0 | -0.9 |  |
| 818 | 250170\_at | AT5G14260 |  | 1 | -0.7 | -0.1 | -0.1 | -0.3 | -0.1 | 0.1 | 0.4 | -0.1 | -0.7 | -1.6 | -0.1 | -1.8 | -1.2 | -1.1 | -2.1 | -1.2 | -1.2 | -1.3 | -0.3 | -0.2 | -0.6 | 0 | -0.2 | -0.2 | -0.2 | -1.3 | -0.2 | -0.4 | SET domain-containing protein; similar to SET domain-containing protein [Arabidopsis thaliana] (TAIR:AT3G07670.1); similar to Os12g0236900 [Oryza sativa (japonica cultivar-group)] (GB:NP\_001066466.1); similar to Nuclear protein SET [Medicago truncatula] (GB:ABE81113.1); contains InterPro domain Nuclear protein SET; (InterPro:IPR001214) |
| 817 | 246736\_at | AT5G27560 |  | 2 | -0.7 | -0.3 | -0.1 | -0.3 | 0 | -0.1 | 0 | 0 | -0.5 | -1.3 | 0.1 | -2.6 | -2.1 | -0.7 | -4.1 | -2.1 | -1.4 | -1.7 | -0.2 | -0.5 | -0.6 | 0 | -0.4 | -0.2 | -0.2 | -0.8 | -0.1 | -0.3 | similar to Os06g0146300 [Oryza sativa (japonica cultivar-group)] (GB:NP\_001056793.1); similar to unnamed protein product [Ostreococcus tauri] (GB:CAL54638.1); contains domain FAD/NAD-linked reductases, dimerisation (C-terminal) domain (SSF55424) |
| 816 | 262151\_at | AT1G52510 |  | 1 | -0.7 | -0.4 | -0.1 | 0 | 0.2 | 0.3 | 0.2 | -0.4 | -1.3 | -2.7 | -0.6 | -2.8 | -3.1 | -3.7 | -3.4 | -2.6 | -1.9 | -2.1 | -1.1 | 0 | -0.1 | 0.1 | -0.2 | 0.1 | 0.2 | -0.8 | 0.2 | 0.1 | hydrolase, alpha/beta fold family protein; similar to hydrolase, alpha/beta fold family protein [Arabidopsis thaliana] (TAIR:AT4G12830.1); similar to Alpha/beta hydrolase fold [Medicago truncatula] (GB:ABE77901.1); contains InterPro domain Esterase/lipase/thioesterase; (InterPro:IPR000379); contains InterPro domain Alpha/beta hydrolase fold-1; (InterPro:IPR000073); contains InterPro domain Epoxide hydrolase; (InterPro:IPR000639); contains InterPro domain Alpha/beta hydrolase; (InterPro:IPR003089), hydrolase; similar to hydrolase, alpha/beta fold family protein [Arabidopsis thaliana] (TAIR:AT4G12830.1); similar to Alpha/beta hydrolase fold [Medicago truncatula] (GB:ABE77901.1); contains InterPro domain Esterase/lipase/thioesterase; (InterPro:IPR000379); contains InterPro domain Alpha/beta hydrolase fold-1; (InterPro:IPR000073); contains InterPro domain Epoxide hydrolase; (InterPro:IPR000639); contains InterPro domain Alpha/beta hydrolase; (InterPro:IPR003089) |
| 815 | 251353\_at | AT3G61080 |  | 2 | -0.9 | -0.8 | -0.2 | -0.2 | 0.1 | 0 | 0.1 | -0.5 | -1.3 | -1.9 | -0.8 | -2.5 | -2.6 | -1.3 | -3 | -2.4 | -3 | -1.8 | -1.2 | -0.5 | -0.5 | -0.1 | -0.1 | -0.3 | 0 | -0.9 | 0 | -0.4 | fructosamine kinase family protein; similar to hypothetical protein alr5126 [Nostoc sp. PCC 7120] (GB:NP\_489166.1); similar to Os03g0117800 [Oryza sativa (japonica cultivar-group)] (GB:NP\_001048768.1); contains InterPro domain Protein kinase-like; (InterPro:IPR011009); contains InterPro domain Fructosamine kinase; (InterPro:IPR005581) |
| 814 | 265319\_at | AT2G22670 | IAA8 | 8 | -0.5 | 0 | -0.2 | -0.1 | 0.2 | 0.1 | 0.2 | -0.3 | -1 | -1.6 | -0.5 | -1.8 | -2.1 | -3.2 | -2.8 | -1.8 | -1.2 | -1.3 | -0.7 | -0.3 | -0.4 | -0.2 | 0.1 | -0.2 | -0.4 | -0.6 | -0.1 | -0.2 | IAA8 (IAA8) gene is auxin inducible. |
| 813 | 245730\_at | AT1G73470 |  |  | -0.9 | -0.1 | -0.3 | -0.2 | 0.1 | 0.4 | 0.2 | -0.4 | -0.8 | -0.8 | 0 | -1.7 | -2.1 | -1.4 | -1.9 | -1.3 | -2.3 | -0.3 | -0.5 | -0.4 | -0.7 | -0.2 | -0.1 | -0.1 | -0.2 | -0.6 | -0.2 | -0.1 | similar to hypothetical protein slr0975 [Synechocystis sp. PCC 6803] (GB:NP\_440211.1); similar to Os09g0448900 [Oryza sativa (japonica cultivar-group)] (GB:NP\_001063318.1) |
| 812 | 256130\_at | AT1G18170 |  | 1 | -0.5 | -0.3 | 0 | 0.1 | 0.2 | -0.4 | 0.3 | -0.3 | -0.7 | -0.7 | -0.5 | -2 | -1.7 | -1.1 | -2 | -1.7 | -2.3 | -1.7 | -0.9 | -0.4 | -0.5 | 0 | 0 | 0 | -0.5 | -1.4 | -0.1 | -0.1 | immunophilin / FKBP-type peptidyl-prolyl cis-trans isomerase family protein; similar to immunophilin / FKBP-type peptidyl-prolyl cis-trans isomerase family protein [Arabidopsis thaliana] (TAIR:AT1G73655.1); similar to hypothetical protein MtrDRAFT\_AC139526g24v1 [Medicago truncatula] (GB:ABE80155.1); similar to Os03g0708500 [Oryza sativa (japonica cultivar-group)] (GB:NP\_001051037.1); contains InterPro domain Peptidylprolyl isomerase, FKBP-type; (InterPro:IPR001179) |
| 811 | 245793\_at | AT1G32220 |  | 3 | -0.6 | -0.2 | -0.1 | 0.1 | -0.1 | 0 | 0.1 | -0.3 | -0.5 | -2.3 | -0.5 | -2.5 | -1.4 | -1.7 | -2.3 | -1.9 | -2.2 | -1.7 | -0.7 | -0.4 | -0.3 | 0.1 | 0 | -0.1 | -0.1 | -0.6 | -0.1 | -0.1 | catalytic/ coenzyme binding; similar to catalytic/ coenzyme binding [Arabidopsis thaliana] (TAIR:AT5G10730.1); similar to Os04g0403500 [Oryza sativa (japonica cultivar-group)] (GB:NP\_001052699.1); similar to hypothetical protein [Cleome spinosa] (GB:ABD96868.1); similar to Os02g0556100 [Oryza sativa (japonica cultivar-group)] (GB:NP\_001047127.1); contains InterPro domain NAD-dependent epimerase/dehydratase; (InterPro:IPR001509) |
| 810 | 262955\_at | AT1G54520 |  | 1 | -0.3 | -0.1 | 0.1 | -0.1 | -0.1 | 0 | 0.1 | -0.2 | -0.4 | -0.7 | -0.3 | -0.5 | -0.3 | -0.8 | -0.6 | -0.7 | -1.2 | -0.5 | -0.6 | -0.5 | -0.5 | 0.1 | 0 | -0.2 | -0.2 | -0.6 | 0.1 | 0 | similar to unknown protein [Arabidopsis thaliana] (TAIR:AT5G57345.1); similar to unknown protein [Oryza sativa (japonica cultivar-group)] (GB:AAO19365.1); contains InterPro domain Protein of unknown function DUF1517; (InterPro:IPR010903) |
| 809 | 246007\_at | AT5G08410 | FTRA2 | 2 | -0.6 | -0.1 | 0.1 | 0 | -0.1 | 0.7 | -0.1 | -0.3 | -0.9 | -0.2 | -0.4 | -1.2 | -1.1 | -1.1 | -1.2 | -0.9 | -1.6 | -1.3 | -0.3 | -0.7 | -0.5 | 0 | -0.1 | -0.3 | -0.3 | -1.1 | 0 | -0.4 | FTRA2 (ferredoxin/thioredoxin reductase subunit A (variable subunit) 2); ferredoxin:thioredoxin reductase; similar to FTRA1 (ferredoxin/thioredoxin reductase subunit A (variable subunit) 1), ferredoxin:thioredoxin reductase [Arabidopsis thaliana] (TAIR:AT5G23440.1); similar to Os04g0528800 [Oryza sativa (japonica cultivar-group)] (GB:NP\_001053378.1); similar to ferredoxin-thioredoxin reductase SU A [Spinacia oleracea] (GB:CAA54407.1); similar to OSJNBa0074L08.8 [Oryza sativa (japonica cultivar-group)] (GB:CAD41197.2); contains InterPro domain Ferredoxin thioredoxin reductase, alpha chain; (InterPro:IPR004207); contains InterPro domain Electron transport accessory protein; (InterPro:IPR008990) |
| 808 | 261948\_at | AT1G64680 |  |  | -0.2 | -0.2 | 0 | 0.1 | -0.1 | -0.2 | 0 | -0.3 | -0.7 | -1.7 | -0.4 | -1.8 | -1.9 | -1.4 | -3.6 | -2.6 | -2 | -1.5 | -0.5 | -0.4 | -0.2 | 0 | -0.5 | -0.3 | -0.3 | -0.7 | 0.1 | -0.3 | similar to unknown protein [Arabidopsis thaliana] (TAIR:AT1G03055.1); similar to hypothetical protein LOC\_Os11g37650 [Oryza sativa (japonica cultivar-group)] (GB:ABA94460.1); similar to Os08g0114100 [Oryza sativa (japonica cultivar-group)] (GB:NP\_001060847.1) |
| 807 | 248491\_at | AT5G51010 |  |  | -0.3 | -0.5 | 0.3 | -0.2 | -0.1 | -0.4 | 0 | -0.6 | -0.7 | -1.8 | -0.6 | -1.7 | -1.7 | -1.5 | -3.2 | -2.1 | -2.8 | -0.8 | -0.3 | -1.1 | -0.2 | 0.1 | -0.1 | 0 | -0.6 | -1.1 | -0.3 | -0.6 | rubredoxin family protein; similar to rubredoxin family protein [Arabidopsis thaliana] (TAIR:AT5G17170.1); similar to Rubredoxin-type Fe(Cys)4 protein [Medicago truncatula] (GB:ABE80208.1); contains InterPro domain Rubredoxin-type Fe(Cys)4 protein; (InterPro:IPR004039) |
| 806 | 260044\_at | AT1G73655 |  |  | -0.8 | -0.2 | -0.3 | 0.1 | 0.1 | -0.6 | -0.2 | -0.2 | -0.4 | -2.1 | -0.6 | -1.7 | -1.4 | -2.3 | -3 | -2.3 | -1.9 | -1.9 | 0 | 0 | -0.6 | -0.1 | -0.4 | -0.6 | -0.3 | -1.1 | 0 | 0 | immunophilin / FKBP-type peptidyl-prolyl cis-trans isomerase family protein; similar to immunophilin / FKBP-type peptidyl-prolyl cis-trans isomerase family protein [Arabidopsis thaliana] (TAIR:AT1G18170.1); similar to hypothetical protein MtrDRAFT\_AC139526g24v1 [Medicago truncatula] (GB:ABE80155.1); contains InterPro domain Peptidylprolyl isomerase, FKBP-type; (InterPro:IPR001179) |
| 805 | 258281\_at | AT3G26900 |  | 2 | -0.6 | -0.2 | -0.1 | 0 | 0.1 | -0.2 | 0.1 | -0.1 | -0.1 | -1.1 | -0.2 | -1.3 | -0.7 | -1.3 | -1.7 | -1.8 | -1.2 | -0.8 | 0 | -0.1 | -0.1 | -0.1 | -0.2 | -0.2 | -0.1 | -0.7 | 0 | 0 | shikimate kinase family protein; similar to ATP binding / shikimate kinase [Arabidopsis thaliana] (TAIR:AT2G21940.5); similar to unknown protein [Oryza sativa (japonica cultivar-group)] (GB:BAD45509.1); similar to Os01g0102600 [Oryza sativa (japonica cultivar-group)] (GB:NP\_001041750.1); contains InterPro domain Shikimate kinase; (InterPro:IPR000623) |
| 804 | 252130\_at | AT3G50820 | OEC33, PSBO-2, PSBO2 | 14 | -0.6 | -0.1 | -0.2 | 0 | 0 | 0 | 0 | 0 | -0.3 | -1.5 | -0.2 | -1.4 | -1.1 | -2.8 | -3.2 | -2.5 | -0.7 | -1.1 | -0.3 | 0 | 0 | -0.4 | -0.3 | -0.4 | -0.2 | -0.8 | 0 | -0.1 | Encodes a protein which is an extrinsic subunit of photosystem II and which has been proposed to play a central role in stabilization of the catalytic manganese cluster. In *Arabidopsis thaliana* the PsbO proteins are encoded by two genes: *psbO1* and *psbO2*. PsbO2 is the minor isoform in the wild-type. Mutants defective in this gene have been shown to be affected in the dephosphorylation of the D1 protein of PSII., Encodes a protein which is an extrinsic subunit of photosystem II and which has been proposed to play a central role in stabilization of the catalytic manganese cluster. In *Arabidopsis thaliana* the PsbO proteins are encoded by two genes: *psbO1* and *psbO2*. PsbO2 is the minor isoform in the wild-type. Mutants defective in this gene have been shown to be affected in the dephosphorylation of the D1 protein of PSII., Encodes a protein which is an extrinsic subunit of photosystem II and which has been proposed to play a central role in stabilization of the catalytic manganese cluster. In *Arabidopsis thaliana* the PsbO proteins are encoded by two genes: *psbO1* and *psbO2*. PsbO2 is the minor isoform in the wild-type. Mutants defective in this gene have been shown to be affected in the dephosphorylation of the D1 protein of PSII. |
| 803 | 261218\_at | AT1G20020 | ATLFNR2, FNR2 | 6 | -0.6 | -0.2 | -0.1 | 0 | -0.1 | -0.4 | 0 | 0 | -0.3 | -1.6 | -0.2 | -1.2 | -0.7 | -1.1 | -2.7 | -1.9 | -1.1 | -1.3 | 0.1 | 0.3 | 0.2 | -0.1 | -0.2 | -0.3 | -0.1 | -0.8 | 0 | 0.1 | Encodes a leaf-type ferredoxin:NADP(H) oxidoreductase. It is present in both chloroplast stroma and thylakoid membranes but is more abundant in the stroma, Encodes a leaf-type ferredoxin:NADP(H) oxidoreductase. It is present in both chloroplast stroma and thylakoid membranes but is more abundant in the stroma |
| 802 | 247131\_at | AT5G66190 | ATLFNR1, FNR1 | 5 | -0.7 | -0.2 | -0.1 | 0.2 | -0.1 | -0.2 | -0.2 | -0.1 | -0.2 | -1 | -0.1 | -1.1 | -0.6 | -1.6 | -2.7 | -1.7 | -0.8 | -1.3 | -0.1 | 0 | 0.1 | -0.1 | -0.2 | -0.3 | 0.1 | -0.6 | 0.1 | 0.1 | Encodes a leaf-type ferredoxin:NADP(H) oxidoreductase. It is present in both chloroplast stroma and thylakoid membranes but is more abundant in the thylakoid. The affinity of this enzyme for ferredoxin is slightly, but significantly, higher than AtLFNR2, an isoform of the same enzyme. AtLFNR1 forms a heterodimer with AtFNR2 and is also a prerequisite to attach AtFNR2 to the thylakoid membrane., Encodes a leaf-type ferredoxin:NADP(H) oxidoreductase. It is present in both chloroplast stroma and thylakoid membranes but is more abundant in the thylakoid. The affinity of this enzyme for ferredoxin is slightly, but significantly, higher than AtLFNR2, an isoform of the same enzyme. AtLFNR1 forms a heterodimer with AtFNR2 and is also a prerequisite to attach AtFNR2 to the thylakoid membrane. |
| 801 | 259625\_at | AT1G42970 | GAPB | 12 | -0.6 | -0.2 | -0.1 | -0.1 | 0 | -0.2 | 0 | 0 | -0.1 | -1.1 | -0.2 | -1.5 | -1.1 | -1.5 | -3.4 | -2.5 | -0.9 | -1.4 | -0.3 | 0 | 0 | -0.1 | -0.2 | -0.3 | -0.1 | -0.5 | 0 | 0 | Encodes chloroplast localized glyceraldehyde-3-phosphate dehydrogenase. |
| 800 | 265628\_at | AT2G27290 |  | 1 | -0.9 | -0.5 | -0.4 | 0.2 | -0.2 | 0 | 0.4 | -0.2 | -0.6 | -0.5 | -0.3 | -1.1 | -0.9 | -0.9 | -1.2 | -1.1 | -1.5 | -0.6 | -0.1 | -0.1 | -0.2 | -0.3 | -0.3 | 0 | 0.2 | -0.6 | 0 | 0.1 | similar to unknown protein [Arabidopsis thaliana] (TAIR:AT2G20940.1); similar to Os04g0416000 [Oryza sativa (japonica cultivar-group)] (GB:NP\_001052762.1); similar to Predicted membrane protein (ISS) [Ostreococcus tauri] (GB:CAL57011.1); similar to OSIGBa0092M08.4 [Oryza sativa (indica cultivar-group)] (GB:CAH67592.1); contains InterPro domain Protein of unknown function DUF1279; (InterPro:IPR009688) |
| 799 | 246651\_at | AT5G35170 |  | 1 | -1.1 | -0.3 | -0.2 | -0.1 | 0.1 | 0.1 | 0.2 | -0.1 | -0.8 | -0.7 | -0.6 | -1.8 | -1.4 | -1.6 | -1.9 | -1.8 | -2.4 | -1.6 | -0.1 | 0.2 | 0.2 | 0 | -0.2 | -0.2 | 0 | -1.1 | -0.1 | -0.1 | adenylate kinase family protein; similar to adenylate kinase, chloroplast, putative / ATP-AMP transphosphorylase, putative [Arabidopsis thaliana] (TAIR:AT5G47840.1); similar to Os08g0109300 [Oryza sativa (japonica cultivar-group)] (GB:NP\_001060810.1); similar to Adenylate kinase, chloroplast (ATP-AMP transphosphorylase) (GB:P43188); similar to Os08g0288200 [Oryza sativa (japonica cultivar-group)] (GB:NP\_001061454.1); contains InterPro domain Adenylate kinase, subfamily; (InterPro:IPR006259); contains InterPro domain Adenylate/cytidine kinase, N-terminal; (InterPro:IPR011769); contains InterPro domain Adenylate kinase; (InterPro:IPR000850), nucleotide kinase; similar to adenylate kinase, chloroplast, putative / ATP-AMP transphosphorylase, putative [Arabidopsis thaliana] (TAIR:AT5G47840.1); similar to Os08g0109300 [Oryza sativa (japonica cultivar-group)] (GB:NP\_001060810.1); similar to Adenylate kinase, chloroplast (ATP-AMP transphosphorylase) (GB:P43188); similar to Os08g0288200 [Oryza sativa (japonica cultivar-group)] (GB:NP\_001061454.1); contains InterPro domain Adenylate kinase, subfamily; (InterPro:IPR006259); contains InterPro domain Adenylate/cytidine kinase, N-terminal; (InterPro:IPR011769); contains InterPro domain Adenylate kinase; (InterPro:IPR000850) |
| 798 | 256979\_at | AT3G21055 | PSBTN |  | -0.3 | -0.2 | -0.1 | 0 | -0.1 | -0.2 | 0.1 | -0.1 | -0.3 | -2.3 | -0.4 | -1.2 | -0.9 | -3.3 | -3.3 | -2.3 | -1.4 | -0.6 | -0.2 | -0.1 | -0.2 | -0.2 | 0 | -0.1 | 0 | -0.5 | 0 | 0 | Encodes photosystem II 5 kD protein subunit PSII-T. This is a nuclear-encoded gene (PsbTn) which also has a plastid-encoded paralog (PsbTc). |
| 797 | 255248\_at | AT4G05180 | PSBQ, PSBQ-2, PSII-Q | 6 | -0.3 | -0.1 | 0 | 0.1 | 0 | 0 | 0.1 | -0.1 | -0.1 | -1 | -0.2 | -0.7 | -0.5 | -1.1 | -2.2 | -1.2 | -0.8 | -0.4 | -0.1 | 0 | -0.1 | -0.1 | 0 | 0 | 0 | -0.3 | 0.2 | 0.1 | Encodes the PsbQ subunit of the oxygen evolving complex of photosystem II., Encodes the PsbQ subunit of the oxygen evolving complex of photosystem II., Encodes the PsbQ subunit of the oxygen evolving complex of photosystem II. |
| 796 | 260542\_at | AT2G43560 |  | 3 | -0.6 | -0.2 | 0 | 0 | 0 | -0.2 | 0 | -0.1 | -0.6 | -2.3 | -0.6 | -2.4 | -1.7 | -2.5 | -3.4 | -1.9 | -1.7 | -1.6 | -0.2 | -0.5 | -0.1 | -0.2 | -0.2 | -0.1 | 0.1 | -0.7 | 0.2 | 0 | immunophilin / FKBP-type peptidyl-prolyl cis-trans isomerase family protein; Identical to Probable FKBP-type peptidyl-prolyl cis-trans isomerase 2, chloroplast precursor (EC 5.2.1.8) (PPIase) (Rotamase) [Arabidopsis Thaliana] (GB:O22870;GB:Q940C0); similar to FK506-binding protein 1 (FKBP13) [Arabidopsis thaliana] (TAIR:AT5G45680.1); similar to Os08g0541400 [Oryza sativa (japonica cultivar-group)] (GB:NP\_001062387.1); similar to putative FKBP type peptidyl-prolyl cis-trans isomerase [Pyrus communis] (GB:AAL66192.1); contains InterPro domain Peptidylprolyl isomerase, FKBP-type; (InterPro:IPR001179) |
| 795 | 259838\_at | AT1G52220 |  | 1 | -0.7 | 0 | -0.1 | 0.2 | 0 | 0 | 0.1 | -0.2 | -0.3 | -1.9 | -0.3 | -1.2 | -0.7 | -2.8 | -2.4 | -1.7 | -1.7 | -0.8 | -0.1 | 0 | -0.3 | -0.1 | -0.1 | -0.2 | 0 | -0.7 | 0 | 0 | similar to TMP14 (THYLAKOID MEMBRANE PHOSPHOPROTEIN OF 14 KDA) [Arabidopsis thaliana] (TAIR:AT2G46820.2); similar to P0460E08.25 [Oryza sativa (japonica cultivar-group)] (GB:BAB61215.1); similar to Os01g0761000 [Oryza sativa (japonica cultivar-group)] (GB:NP\_001044320.1) |
| 794 | 249120\_at | AT5G43750 |  | 2 | -1.1 | -0.1 | -0.1 | 0 | 0 | 0.1 | 0 | -0.3 | -0.9 | -2.4 | -0.6 | -2.7 | -2.1 | -3.5 | -3.9 | -3.5 | -1.8 | -1.8 | -0.2 | 0 | -0.3 | -0.1 | -0.3 | -0.1 | -0.1 | -0.9 | 0 | 0 | similar to unknown protein [Oryza sativa (japonica cultivar-group)] (GB:BAD88171.1) |
| 793 | 262010\_at | AT1G35612 |  |  | -0.8 | -0.1 | -0.9 | -0.5 | 0.2 | -0.2 | 1.2 | 0.1 | -0.9 | -1.1 | -0.6 | -1.1 | -1 | -2.3 | -2.3 | -3.1 | -2.2 | -2.5 | -0.1 | -0.3 | -1.3 | 0 | -0.2 | 0.5 | 0.2 | -0.8 | 0.6 | 0.7 | pseudogene of Ulp1 protease family protein |
| 792 | 262368\_at | AT1G73060 |  | 1 | -0.6 | -0.2 | -0.1 | 0.5 | -0.1 | -0.2 | 0 | 0.1 | -0.4 | -1.6 | -0.3 | -1.9 | -1.5 | -1.6 | -3 | -1.8 | -1.4 | -1.8 | 0 | 0 | 0.3 | -0.1 | -0.2 | -0.2 | 0.5 | -0.7 | 0.5 | 0.4 | similar to unknown protein [Arabidopsis thaliana] (TAIR:AT5G48790.1); similar to unnamed protein product [Ostreococcus tauri] (GB:CAL50320.1); similar to Os02g0117100 [Oryza sativa (japonica cultivar-group)] (GB:NP\_001045684.1) |
| 791 | 255435\_at | AT4G03280 | PETC, PGR1 | 12 | -0.3 | -0.1 | -0.1 | 0.1 | 0 | -0.2 | 0 | 0 | -0.1 | -0.6 | -0.2 | -1 | -0.6 | -0.7 | -1.8 | -1.5 | -0.7 | -0.5 | -0.1 | -0.1 | 0 | -0.1 | -0.1 | -0.1 | 0 | -0.2 | 0.1 | 0 | Encodes the Rieske FeS center of cytochrome b6f complex. Gene is expressed in shoot but not in root. Mutant has reduced electron transport at saturating light intensities and Q-cycle activity is hypersensitive to acidification of the thylakoid lumen., Encodes the Rieske FeS center of cytochrome b6f complex. Gene is expressed in shoot but not in root. Mutant has reduced electron transport at saturating light intensities and Q-cycle activity is hypersensitive to acidification of the thylakoid lumen. |
| 790 | 267435\_at | AT2G33800 |  | 2 | -0.4 | -0.1 | 0 | 0.2 | 0.1 | -0.3 | 0 | -0.1 | -0.3 | -1.5 | -0.5 | -1.9 | -1.5 | -1.6 | -3.5 | -2.4 | -0.8 | -1 | 0.1 | 0 | 0 | -0.1 | -0.2 | -0.2 | 0.1 | -0.4 | 0 | 0 | ribosomal protein S5 family protein; Identical to 30S ribosomal protein S5, chloroplast precursor (rps5) [Arabidopsis Thaliana] (GB:P93014); similar to ribosomal protein S5 family protein [Arabidopsis thaliana] (TAIR:AT1G64880.1); similar to 30S ribosomal protein S5, chloroplast precursor (GB:Q9ST69); contains InterPro domain Ribosomal protein S5, bacterial and chloroplast; (InterPro:IPR005712); contains InterPro domain Ribosomal protein S5, C-terminal; (InterPro:IPR005324); contains InterPro domain Ribosomal protein S5; (InterPro:IPR000851) |
| 789 | 245044\_at | AT2G26500 |  | 2 | -0.3 | -0.1 | 0 | -0.1 | 0 | -0.3 | 0.1 | -0.1 | -0.2 | -0.6 | -0.2 | -0.8 | -0.5 | -0.5 | -1.1 | -0.9 | -0.9 | -0.1 | 0.1 | -0.1 | -0.1 | -0.1 | -0.2 | -0.2 | -0.1 | -0.3 | 0 | 0 | cytochrome b6f complex subunit (petM), putative; similar to unknown [Euphorbia esula] (GB:AAF34768.1), cytochrome b6f complex subunit (petM), putative; similar to unknown [Euphorbia esula] (GB:AAF34768.1); contains InterPro domain PetM of cytochrome b6f complex subunit 7; (InterPro:IPR012595) |
| 788 | 267526\_at | AT2G30570 | PSBW | 1 | -0.1 | -0.3 | -0.2 | 0.2 | 0 | -0.1 | 0.1 | 0 | -0.2 | -0.5 | -0.1 | -0.5 | -0.3 | -0.6 | -0.8 | -1.2 | -0.8 | -0.3 | 0 | 0 | -0.1 | -0.3 | -0.1 | -0.2 | 0.1 | -0.2 | 0.1 | 0 | Encodes a protein similar to photosystem II reaction center subunit W. |
| 787 | 255718\_at | AT1G32070 | ATNSI | 3 | -0.4 | -0.3 | -0.2 | 0.2 | 0.1 | -0.3 | 0 | -0.2 | -0.4 | -1.1 | -0.1 | -0.9 | -0.5 | -0.5 | -1.2 | -1.3 | -1.5 | -1 | 0.1 | 0.1 | -0.3 | -0.5 | -0.2 | -0.3 | 0.1 | -1.1 | 0.1 | 0.3 | Encodes a nuclear acetyltransferase (NSI)that interacts with the geminivirus movement protein NSP. This interaction is required for viral infection and systemic spread. Acetylates the viral coat protein (CP) in vitro, but not NSP. NSP inhibits NSI activity in vitro. NSI is highly transcribed in phloem and in xylem parenchyma cells, and in the apical meristem and guard cells, within young tissues in Arabidopsis, and its expression is turned off as tissues mature. |
| 786 | 255567\_at | AT4G01150 |  | 3 | -0.1 | -0.3 | -0.2 | -0.2 | 0 | -0.2 | 0 | 0 | -0.3 | -0.7 | -0.1 | -1.2 | -0.8 | -1.2 | -1.5 | -1.4 | -0.7 | -0.6 | -0.1 | -0.1 | -0.1 | -0.3 | -0.1 | -0.2 | -0.1 | -0.1 | 0 | 0.1 | similar to threonine endopeptidase [Arabidopsis thaliana] (TAIR:AT4G38100.1); similar to unknown (GB:AAB00107.1) |
| 785 | 253738\_at | AT4G28750 | PSAE-1 | 5 | -0.2 | -0.3 | -0.2 | -0.1 | 0 | -0.2 | 0 | 0 | -0.1 | -0.7 | -0.2 | -0.8 | -0.7 | -1.7 | -1.5 | -1.4 | -0.7 | -0.3 | 0 | -0.1 | -0.2 | -0.2 | 0 | -0.2 | 0 | -0.2 | 0.2 | 0 | mutant has Decreased effective quantum yield of photosystem II; Pale green plants; Reduced growth rate; Subunit E of Photosystem I |
| 784 | 259727\_at | AT1G60950 | ATFD2, FED | 6 | -0.1 | -0.2 | -0.2 | -0.3 | -0.1 | -0.2 | 0 | 0 | -0.1 | -0.6 | -0.2 | -0.6 | -0.5 | -0.5 | -1.4 | -1.5 | -1.2 | -0.4 | 0.1 | -0.1 | 0 | -0.2 | 0 | -0.1 | 0 | -0.4 | 0.1 | 0 | encodes a ferredoxin gene, encodes a ferredoxin gene |
| 783 | 251664\_at | AT3G56940 | ACSF, CHL27, CRD1 | 9 | -0.3 | -0.1 | -0.1 | -0.1 | 0 | -0.1 | 0 | 0 | -0.1 | -1 | -0.3 | -0.9 | -0.6 | -3.1 | -1.8 | -2.4 | -1.1 | -0.5 | -0.1 | 0 | -0.1 | -0.1 | -0.1 | -0.2 | -0.1 | -0.5 | 0.1 | 0 | Encodes a putative ZIP protein with varying mRNA accumulation in leaves, stems and roots. Has a consensus carboxylate-bridged di-iron binding site., Encodes a putative ZIP protein with varying mRNA accumulation in leaves, stems and roots. Has a consensus carboxylate-bridged di-iron binding site., Encodes a putative ZIP protein with varying mRNA accumulation in leaves, stems and roots. Has a consensus carboxylate-bridged di-iron binding site. |
| 782 | 248886\_at | AT5G46110 | APE2, TPT | 3 | -0.5 | -0.1 | -0.1 | -0.2 | 0 | -0.1 | 0.1 | 0 | -0.1 | -1 | -0.2 | -0.7 | -0.6 | -1.4 | -2 | -1.6 | -1 | -0.2 | -0.1 | 0 | -0.2 | -0.2 | -0.1 | -0.2 | -0.1 | -0.5 | 0.1 | -0.1 | mutant has Altered acclimation responses; Chloroplast Triose Phosphate Translocator, mutant has Altered acclimation responses; Chloroplast Triose Phosphate Translocator |
| 781 | 261197\_at | AT1G12900 | GAPA-2, GAPA-2 | 3 | -0.5 | 0 | 0 | -0.1 | 0 | -0.1 | 0.1 | 0 | -0.1 | -1.8 | -0.2 | -1.1 | -1.1 | -2 | -4.1 | -2.8 | -1.3 | -0.7 | -0.2 | -0.1 | -0.1 | -0.2 | -0.1 | -0.2 | 0 | -0.7 | 0 | 0.1 | GAPA-2; glyceraldehyde-3-phosphate dehydrogenase; similar to GAPA (GLYCERALDEHYDE 3-PHOSPHATE DEHYDROGENASE A SUBUNIT), glyceraldehyde-3-phosphate dehydrogenase [Arabidopsis thaliana] (TAIR:AT3G26650.1); similar to glyceraldehyde-3-phosphate dehydrogenase A subunit [Glycine max] (GB:ABA86963.1); similar to Glyceraldehyde-3-phosphate dehydrogenase A, chloroplast precursor (NADP-dependent glyceraldehydephosphate dehydrogenase subunit A) (GB:P19866); similar to Glyceraldehyde-3-phosphate dehydrogenase A, chloroplast precursor (NADP-dependent glyceraldehydephosphate dehydrogenase subunit A) (GB:P12858); contains InterPro domain Glyceraldehyde-3-phosphate dehydrogenase, type I; (InterPro:IPR006424); contains InterPro domain Glyceraldehyde 3-phosphate dehydrogenase; (InterPro:IPR000173), GAPA-2; similar to GAPA (GLYCERALDEHYDE 3-PHOSPHATE DEHYDROGENASE A SUBUNIT), glyceraldehyde-3-phosphate dehydrogenase [Arabidopsis thaliana] (TAIR:AT3G26650.1); similar to Glyceraldehyde-3-phosphate dehydrogenase A, chloroplast precursor (NADP-dependent glyceraldehydephosphate dehydrogenase subunit A) (GB:P19866); similar to Chain O, Crystal Structure Of Mutant T33a Of Photosynthetic Glyceraldehyde-3-Phosphate Dehydrogenase A4 Isoform, Complexed With Nadp (GB:1RM3); similar to Chain O, The Dual Coenzyme Specificity Of Photosynthetic Glyceraldehyde-3-Phosphate Dehydrogenase Interpreted By The Crystal Structure Of A4 Isoform Complexed With Nad (GB:1NBO); contains InterPro domain Glyceraldehyde 3-phosphate dehydrogenase; (InterPro:IPR000173) |
| 780 | 262632\_at | AT1G06680 | OEE2, PSBP-1, PSII-P | 14 | -0.4 | -0.2 | -0.2 | -0.1 | 0 | -0.2 | 0 | -0.1 | -0.1 | -0.9 | -0.2 | -0.5 | -0.4 | -1.4 | -1.7 | -1.6 | -0.7 | -0.3 | -0.1 | 0 | -0.2 | -0.2 | -0.1 | -0.1 | 0 | -0.2 | 0.1 | 0.1 | Encodes a 23 kD extrinsic protein that is part of photosystem II and participates in the regulation of oxygen evolution., Encodes a 23 kD extrinsic protein that is part of photosystem II and participates in the regulation of oxygen evolution., Encodes a 23 kD extrinsic protein that is part of photosystem II and participates in the regulation of oxygen evolution. |
| 779 | 259840\_at | AT1G52230 | PSAH-2, PSAH2, PSI-H | 4 | -0.2 | -0.3 | -0.2 | -0.1 | 0 | -0.2 | 0 | 0 | -0.2 | -1.6 | -0.3 | -0.9 | -0.8 | -2.8 | -2.6 | -1.7 | -1.2 | -0.5 | -0.1 | 0 | -0.3 | -0.2 | -0.1 | -0.1 | -0.1 | -0.6 | 0.1 | 0 | photosystem I reaction center subunit VI, chloroplast, putative / PSI-H, putative (PSAH2); Identical to Photosystem I reaction center subunit VI-2, chloroplast precursor (PSI-H1) (PSAH2) [Arabidopsis Thaliana] (GB:Q9SUI6); similar to PSAH-1 (photosystem I subunit H-1) [Arabidopsis thaliana] (TAIR:AT3G16140.1); similar to Photosystem I reaction center subunit VI, chloroplast precursor (PSI-H) (Light-harvesting complex I 11 kDa protein) (GB:O04006); contains InterPro domain Photosystem I reaction centre subunit VI; (InterPro:IPR004928), photosystem I reaction center subunit VI, chloroplast, putative / PSI-H, putative (PSAH2); Identical to Photosystem I reaction center subunit VI-2, chloroplast precursor (PSI-H1) (PSAH2) [Arabidopsis Thaliana] (GB:Q9SUI6); similar to PSAH-1 (photosystem I subunit H-1) [Arabidopsis thaliana] (TAIR:AT3G16140.1); similar to Photosystem I reaction center subunit VI, chloroplast precursor (PSI-H) (Light-harvesting complex I 11 kDa protein) (GB:O04006); contains InterPro domain Photosystem I reaction centre subunit VI; (InterPro:IPR004928), photosystem I reaction center subunit VI, chloroplast, putative / PSI-H, putative (PSAH2); Identical to Photosystem I reaction center subunit VI-2, chloroplast precursor (PSI-H1) (PSAH2) [Arabidopsis Thaliana] (GB:Q9SUI6); similar to PSAH-1 (photosystem I subunit H-1) [Arabidopsis thaliana] (TAIR:AT3G16140.1); similar to Photosystem I reaction center subunit VI, chloroplast precursor (PSI-H) (Light-harvesting complex I 11 kDa protein) (GB:O04006); contains InterPro domain Photosystem I reaction centre subunit VI; (InterPro:IPR004928) |
| 778 | 255290\_at | AT4G04640 | ATPC1 | 6 | -0.3 | -0.1 | 0 | -0.1 | 0 | -0.3 | 0.1 | 0 | -0.1 | -1 | -0.1 | -0.8 | -0.6 | -0.6 | -2 | -1.4 | -0.8 | -0.6 | 0 | -0.1 | -0.1 | -0.2 | 0 | -0.2 | 0 | -0.6 | 0.1 | 0 | One of two genes (with ATPC2) encoding the gamma subunit of Arabidopsis chloroplast ATP synthase. |
| 777 | 265374\_at | AT2G06520 | PSBX | 5 | -0.1 | -0.1 | 0 | -0.1 | 0 | -0.2 | -0.1 | -0.2 | -0.1 | -0.6 | -0.3 | -0.4 | -0.2 | -1 | -1.3 | -1.5 | -0.8 | -0.4 | 0 | 0 | -0.1 | -0.1 | 0 | -0.1 | 0.1 | -0.3 | 0.1 | -0.1 | Encodes a protein with sequence similarity to the spinach photosystem II subunit PsbX. |
| 776 | 260236\_at | AT1G74470 |  | 3 | -0.5 | -0.1 | -0.1 | -0.1 | 0 | -0.4 | 0 | -0.1 | -0.2 | -0.7 | -0.4 | -1.2 | -0.9 | -2.7 | -3.4 | -2.8 | -1.3 | -0.8 | 0 | 0 | 0.1 | -0.1 | -0.1 | -0.2 | -0.2 | -1.3 | 0 | 0 | Encodes for a multifunctional protein with geranylgeranyl reductase activity shown to catalyze the reduction of prenylated geranylgeranyl-chlorophyll a to phytyl-chlorophyll a (chlorophyll a) and free geranylgeranyl pyrophosphate to phytyl pyrophosphate. |
| 775 | 245195\_at | AT1G67740 | PSBY, YCF32 | 4 | -0.5 | -0.1 | -0.1 | -0.3 | -0.1 | -0.2 | 0 | 0 | -0.2 | -1.7 | -0.2 | -1.1 | -0.9 | -1.6 | -2.2 | -1.9 | -1.9 | -0.5 | 0 | 0 | 0 | -0.2 | 0 | -0.2 | -0.1 | -0.7 | 0 | 0 | PsbY precursor (psbY) mRNA. This single nuclear gene is imported into the chloroplasts where it is processed into two integral membrane proteins with identical topology (PsbY-1 and PsbY-2). The protein appears to bind manganese but its role is not well understood., PsbY precursor (psbY) mRNA. This single nuclear gene is imported into the chloroplasts where it is processed into two integral membrane proteins with identical topology (PsbY-1 and PsbY-2). The protein appears to bind manganese but its role is not well understood. |
| 774 | 247073\_at | AT5G66570 | MSP-1, OE33, OEE1, OEE33, PSBO-1, PSBO1 | 21 | -0.4 | -0.3 | -0.2 | -0.2 | 0 | -0.2 | 0.1 | -0.1 | -0.1 | -0.8 | -0.2 | -0.7 | -0.4 | -1.8 | -2.1 | -1.5 | -0.6 | -0.3 | 0.1 | 0.1 | 0.1 | -0.2 | -0.1 | -0.1 | -0.2 | -0.3 | 0 | -0.1 | Encodes a protein which is an extrinsic subunit of photosystem II and which has been proposed to play a central role in stabilization of the catalytic manganese cluster. In *Arabidopsis thaliana* the PsbO proteins are encoded by two genes: *psbO1* and *psbO2*. PsbO1 is the major isoform in the wild-type., Encodes a protein which is an extrinsic subunit of photosystem II and which has been proposed to play a central role in stabilization of the catalytic manganese cluster. In *Arabidopsis thaliana* the PsbO proteins are encoded by two genes: *psbO1* and *psbO2*. PsbO1 is the major isoform in the wild-type., Encodes a protein which is an extrinsic subunit of photosystem II and which has been proposed to play a central role in stabilization of the catalytic manganese cluster. In *Arabidopsis thaliana* the PsbO proteins are encoded by two genes: *psbO1* and *psbO2*. PsbO1 is the major isoform in the wild-type., Encodes a protein which is an extrinsic subunit of photosystem II and which has been proposed to play a central role in stabilization of the catalytic manganese cluster. In *Arabidopsis thaliana* the PsbO proteins are encoded by two genes: *psbO1* and *psbO2*. PsbO1 is the major isoform in the wild-type., Encodes a protein which is an extrinsic subunit of photosystem II and which has been proposed to play a central role in stabilization of the catalytic manganese cluster. In *Arabidopsis thaliana* the PsbO proteins are encoded by two genes: *psbO1* and *psbO2*. PsbO1 is the major isoform in the wild-type., Encodes a protein which is an extrinsic subunit of photosystem II and which has been proposed to play a central role in stabilization of the catalytic manganese cluster. In *Arabidopsis thaliana* the PsbO proteins are encoded by two genes: *psbO1* and *psbO2*. PsbO1 is the major isoform in the wild-type. |
| 773 | 259491\_at | AT1G15820 | CP24, LHCB6 | 14 | -0.3 | -0.1 | -0.2 | 0.1 | -0.2 | -0.1 | 0.2 | 0 | -0.2 | -1.4 | -0.5 | -1 | -0.6 | -3.9 | -3 | -2.3 | -1.5 | -0.6 | 0 | 0.1 | 0 | -0.2 | -0.1 | -0.2 | 0 | -0.9 | 0 | 0 | Lhcb6 protein (Lhcb6), light harvesting complex of photosystem II., Lhcb6 protein (Lhcb6), light harvesting complex of photosystem II. |
| 772 | 253420\_at | AT4G32260 |  | 2 | -0.4 | -0.2 | -0.2 | 0 | -0.1 | -0.2 | 0.1 | 0 | -0.2 | -0.8 | -0.1 | -1.4 | -0.8 | -1.1 | -1.7 | -1.4 | -1.4 | -0.7 | 0.1 | 0.2 | 0 | -0.2 | 0 | -0.3 | -0.1 | -0.5 | -0.1 | -0.1 | ATP synthase family; similar to H+-transporting two-sector ATPase, B/B subunit [Medicago truncatula] (GB:ABE81773.1); contains InterPro domain ATPase, F0 complex, subunit B/B' (InterPro:IPR002146) |
| 771 | 252929\_at | AT4G38970 |  | 5 | -0.3 | -0.2 | -0.1 | -0.1 | -0.1 | -0.2 | 0 | 0 | 0.1 | -0.8 | -0.3 | -1 | -0.6 | -0.8 | -2.7 | -2.2 | -0.9 | -0.7 | 0 | 0 | -0.1 | -0.1 | -0.1 | -0.2 | -0.1 | -0.2 | 0.1 | 0 | fructose-bisphosphate aldolase, putative; similar to fructose-bisphosphate aldolase [Arabidopsis thaliana] (TAIR:AT2G21330.2); similar to chloroplast latex aldolase-like protein [Manihot esculenta] (GB:AAV74407.1); contains InterPro domain Fructose-bisphosphate aldolase, class-I; (InterPro:IPR000741), fructose-bisphosphate aldolase, putative; similar to fructose-bisphosphate aldolase, putative [Arabidopsis thaliana] (TAIR:AT2G21330.1); similar to plastidic aldolase NPALDP1 [Nicotiana paniculata] (GB:BAA77604.1); similar to latex plastidic aldolase-like protein [Hevea brasiliensis] (GB:AAM46780.1); similar to plastidic aldolase [Nicotiana paniculata] (GB:BAA77603.1); contains InterPro domain Fructose-bisphosphate aldolase, class-I; (InterPro:IPR000741) |
| 770 | 246596\_at | AT5G14740 | BETA, CA18, CA2 | 7 | -0.6 | -0.1 | -0.1 | -0.1 | 0 | -0.2 | 0 | 0 | 0 | -2.3 | -0.1 | -1.7 | -0.9 | -3.4 | -4.4 | -3.4 | -1.2 | -1.2 | 0 | -0.1 | 0 | -0.1 | 0 | -0.3 | 0.1 | -0.5 | 0.1 | 0 | Encodes a beta carbonic anhydrase likely to be localized in the cytoplasm. Expression of its mRNA is seen in etiolated seedlings and points to a possible nonphotosynthetic role for this isoform., Encodes a beta carbonic anhydrase likely to be localized in the cytoplasm. Expression of its mRNA is seen in etiolated seedlings and points to a possible nonphotosynthetic role for this isoform., Encodes a beta carbonic anhydrase likely to be localized in the cytoplasm. Expression of its mRNA is seen in etiolated seedlings and points to a possible nonphotosynthetic role for this isoform. |
| 769 | 262418\_at | AT1G50320 | ATHX, ATX | 4 | -0.8 | -0.2 | -0.2 | 0.3 | -0.1 | -0.2 | 0.1 | 0.1 | 0.1 | -1 | -0.2 | -1.1 | -0.7 | -1.1 | -1.3 | -0.9 | -1 | -0.4 | 0 | 0.2 | 0 | 0 | -0.2 | -0.3 | 0.1 | -0.2 | 0.3 | 0.1 | encodes a prokaryotic thioredoxin, encodes a prokaryotic thioredoxin |
| 768 | 254480\_at | AT4G20360 | ATRAB8D, ATRABE1B | 2 | -0.2 | -0.2 | -0.2 | -0.1 | 0.1 | -0.3 | 0 | 0.1 | 0.1 | -1.1 | -0.1 | -0.9 | -0.4 | -0.4 | -1.9 | -1.4 | -0.9 | -0.6 | 0 | 0 | 0.1 | -0.2 | 0 | -0.2 | 0 | -0.3 | 0 | 0 | AtRABE1b/AtRab8D (Arabidopsis Rab GTPase homolog E1b); translation elongation factor; Identical to Elongation factor Tu, chloroplast precursor (EF-Tu) (TUFA) [Arabidopsis Thaliana] (GB:P17745); similar to elongation factor Tu, putative / EF-Tu, putative [Arabidopsis thaliana] (TAIR:AT4G02930.1); similar to chloroplast translational elongation factor Tu [Pelargonium graveolens] (GB:AAK08141.1); similar to Elongation factor Tu, chloroplast precursor (EF-Tu) (GB:Q43467); similar to Elongation factor Tu, chloroplast precursor (EF-Tu) (GB:P46280); contains InterPro domain Translation elongation factor Tu; (InterPro:IPR004541); contains InterPro domain Elongation factor Tu, C-terminal; (InterPro:IPR004160); contains InterPro domain Small GTP-binding protein domain; (InterPro:IPR005225); contains InterPro domain Translation factor; (InterPro:IPR009000); contains InterPro domain Translation elongation factor, GTP-binding; (InterPro:IPR013004); contains InterPro domain Elongation factor Tu, domain 2; (InterPro:IPR004161); contains InterPro domain EF-Tu/eEF-1alpha/eIF2-gamma, C-terminal; (InterPro:IPR009001); contains InterPro domain Protein synthesis factor, GTP-binding; (InterPro:IPR000795), AtRABE1b/AtRab8D (Arabidopsis Rab GTPase homolog E1b); translation elongation factor; Identical to Elongation factor Tu, chloroplast precursor (EF-Tu) (TUFA) [Arabidopsis Thaliana] (GB:P17745); similar to elongation factor Tu, putative / EF-Tu, putative [Arabidopsis thaliana] (TAIR:AT4G02930.1); similar to chloroplast translational elongation factor Tu [Pelargonium graveolens] (GB:AAK08141.1); similar to Elongation factor Tu, chloroplast precursor (EF-Tu) (GB:Q43467); similar to Elongation factor Tu, chloroplast precursor (EF-Tu) (GB:P46280); contains InterPro domain Translation elongation factor Tu; (InterPro:IPR004541); contains InterPro domain Elongation factor Tu, C-terminal; (InterPro:IPR004160); contains InterPro domain Small GTP-binding protein domain; (InterPro:IPR005225); contains InterPro domain Translation factor; (InterPro:IPR009000); contains InterPro domain Translation elongation factor, GTP-binding; (InterPro:IPR013004); contains InterPro domain Elongation factor Tu, domain 2; (InterPro:IPR004161); contains InterPro domain EF-Tu/eEF-1alpha/eIF2-gamma, C-terminal; (InterPro:IPR009001); contains InterPro domain Protein synthesis factor, GTP-binding; (InterPro:IPR000795) |
| 767 | 252430\_at | AT3G47470 | CAB4, LHCA4 | 16 | -0.5 | -0.3 | -0.2 | -0.1 | 0 | -0.1 | 0 | 0 | -0.1 | -0.4 | -0.3 | -0.3 | -0.4 | -1.7 | -0.8 | -1.5 | -0.8 | -0.5 | 0 | 0 | 0 | -0.3 | -0.1 | -0.2 | -0.2 | -0.4 | 0 | 0 | Encodes a chlorophyll a/b-binding protein that is more similar to the PSI Cab proteins than the PSII cab proteins. The predicted protein is about 20 amino acids shorter than most known Cab proteins., Encodes a chlorophyll a/b-binding protein that is more similar to the PSI Cab proteins than the PSII cab proteins. The predicted protein is about 20 amino acids shorter than most known Cab proteins. |
| 766 | 255886\_at | AT1G20340 | DRT112, PETE2 | 5 | -0.2 | -0.4 | -0.3 | 0 | -0.1 | -0.3 | 0 | 0 | -0.1 | -1.3 | -0.2 | -0.7 | -0.5 | -1.6 | -1.9 | -1.3 | -0.9 | -0.4 | 0 | 0 | -0.1 | -0.4 | 0 | -0.2 | -0.1 | -0.2 | -0.1 | 0.1 | recombination and DNA-damage resistance protein (DRT112), recombination and DNA-damage resistance protein (DRT112) |
| 765 | 254398\_at | AT4G21280 | PSBQ, PSBQ-1, PSBQA | 11 | -0.4 | -0.2 | -0.1 | -0.1 | 0 | -0.3 | -0.1 | 0 | -0.2 | -2.2 | -0.3 | -1.1 | -0.8 | -3 | -2.9 | -2.2 | -1.2 | -0.9 | 0 | 0 | -0.1 | -0.2 | -0.1 | -0.2 | -0.2 | -0.5 | -0.1 | -0.1 | Encodes the PsbQ subunit of the oxygen evolving complex of photosystem II., Encodes the PsbQ subunit of the oxygen evolving complex of photosystem II., Encodes the PsbQ subunit of the oxygen evolving complex of photosystem II. |
| 764 | 250752\_at | AT5G05690 | CBB3, CPD, CYP90, CYP90A, CYP90A1, DWF3 | 18 | 0 | -0.2 | -5.4 | -0.1 | -0.1 | -0.4 | 1.2 | 0.1 | -0.2 | -0.5 | 0 | -1.5 | -1.3 | -0.9 | -1.9 | -1.4 | -1.7 | -2.4 | 0.2 | -0.2 | -0.6 | -0.1 | -0.1 | -0.9 | 0.1 | -1.7 | 0 | -0.3 | Encodes a member of the CP90A family, a cytochrome P450 monooxygenase which converts 6-deoxocathasterone to 6-deoxoteasterone in the late C6 oxidation pathway and cathasterone to teasterone in the early C6 oxidation pathway of brassinolide biosynthesis. Expressed in cotyledons and leaves. Mutants display de-etiolation and derepression of light-induced genes in the dark, dwarfism, male sterility and activation of stress-regulated genes in the light. The expression of the gene using a CPD promoter:LUC fusion construct was shown to be under circadian and light control. Additionally, the circadian regulation was shown to be independent of BR levels as it remains unchanged in *bri1* mutant lines., Encodes a member of the CP90A family, a cytochrome P450 monooxygenase which converts 6-deoxocathasterone to 6-deoxoteasterone in the late C6 oxidation pathway and cathasterone to teasterone in the early C6 oxidation pathway of brassinolide biosynthesis. Expressed in cotyledons and leaves. Mutants display de-etiolation and derepression of light-induced genes in the dark, dwarfism, male sterility and activation of stress-regulated genes in the light. The expression of the gene using a CPD promoter:LUC fusion construct was shown to be under circadian and light control. Additionally, the circadian regulation was shown to be independent of BR levels as it remains unchanged in *bri1* mutant lines., Encodes a member of the CP90A family, a cytochrome P450 monooxygenase which converts 6-deoxocathasterone to 6-deoxoteasterone in the late C6 oxidation pathway and cathasterone to teasterone in the early C6 oxidation pathway of brassinolide biosynthesis. Expressed in cotyledons and leaves. Mutants display de-etiolation and derepression of light-induced genes in the dark, dwarfism, male sterility and activation of stress-regulated genes in the light. The expression of the gene using a CPD promoter:LUC fusion construct was shown to be under circadian and light control. Additionally, the circadian regulation was shown to be independent of BR levels as it remains unchanged in *bri1* mutant lines., Encodes a member of the CP90A family, a cytochrome P450 monooxygenase which converts 6-deoxocathasterone to 6-deoxoteasterone in the late C6 oxidation pathway and cathasterone to teasterone in the early C6 oxidation pathway of brassinolide biosynthesis. Expressed in cotyledons and leaves. Mutants display de-etiolation and derepression of light-induced genes in the dark, dwarfism, male sterility and activation of stress-regulated genes in the light. The expression of the gene using a CPD promoter:LUC fusion construct was shown to be under circadian and light control. Additionally, the circadian regulation was shown to be independent of BR levels as it remains unchanged in *bri1* mutant lines., Encodes a member of the CP90A family, a cytochrome P450 monooxygenase which converts 6-deoxocathasterone to 6-deoxoteasterone in the late C6 oxidation pathway and cathasterone to teasterone in the early C6 oxidation pathway of brassinolide biosynthesis. Expressed in cotyledons and leaves. Mutants display de-etiolation and derepression of light-induced genes in the dark, dwarfism, male sterility and activation of stress-regulated genes in the light. The expression of the gene using a CPD promoter:LUC fusion construct was shown to be under circadian and light control. Additionally, the circadian regulation was shown to be independent of BR levels as it remains unchanged in *bri1* mutant lines., Encodes a member of the CP90A family, a cytochrome P450 monooxygenase which converts 6-deoxocathasterone to 6-deoxoteasterone in the late C6 oxidation pathway and cathasterone to teasterone in the early C6 oxidation pathway of brassinolide biosynthesis. Expressed in cotyledons and leaves. Mutants display de-etiolation and derepression of light-induced genes in the dark, dwarfism, male sterility and activation of stress-regulated genes in the light. The expression of the gene using a CPD promoter:LUC fusion construct was shown to be under circadian and light control. Additionally, the circadian regulation was shown to be independent of BR levels as it remains unchanged in *bri1* mutant lines. |
| 763 | 265033\_at | AT1G61520 | LHCA3 | 6 | -0.3 | -0.3 | -0.3 | -0.1 | 0 | -0.2 | 0.1 | -0.1 | 0 | -0.5 | -0.1 | -0.3 | -0.2 | -0.9 | -0.8 | -1.2 | -0.8 | -0.5 | -0.1 | -0.1 | -0.2 | -0.3 | 0 | -0.1 | 0.2 | -0.2 | 0.2 | 0.1 | PSI type III chlorophyll a/b-binding protein (Lhca3\*1) |
| 762 | 245061\_at | AT2G39730 | RCA | 23 | -0.3 | -0.4 | -0.3 | -0.2 | -0.1 | -0.2 | 0 | 0.1 | 0 | -0.4 | 0 | -0.4 | -0.2 | -0.7 | -1.2 | -1.7 | -1 | -0.3 | -0.1 | 0 | -0.1 | -0.3 | 0 | -0.1 | -0.1 | -0.4 | 0 | 0 | Rubisco activase, a nuclear-encoded chloroplast protein that consists of two isoforms arising from alternative splicing in most plants. Required for the light activation of rubisco. |
| 761 | 254970\_at | AT4G10340 | LHCB5 | 15 | -0.4 | -0.4 | -0.4 | -0.3 | 0.1 | -0.3 | 0 | 0 | 0 | -0.8 | -0.2 | -0.2 | -0.2 | -1 | -1.6 | -1.5 | -0.5 | -0.2 | -0.1 | 0 | -0.1 | -0.4 | 0 | -0.2 | -0.1 | -0.3 | 0.1 | 0.1 | photosystem II encoding the light-harvesting chlorophyll a/b binding protein CP26 of the antenna system of the photosynthetic apparatus |
| 760 | 251814\_at | AT3G54890 | LHCA1 | 19 | -0.5 | -0.4 | -0.4 | -0.4 | -0.1 | -0.3 | 0 | -0.2 | 0 | -0.7 | -0.2 | -0.4 | -0.3 | -1.5 | -1.2 | -1.5 | -0.9 | -0.1 | 0 | 0 | 0 | -0.3 | -0.1 | -0.2 | -0.2 | -0.2 | 0.1 | 0 | Encodes a component of the light harvesting complex associated with photosystem I. |
| 759 | 261746\_at | AT1G08380 | PSAO | 3 | -0.5 | -0.2 | -0.3 | -0.1 | -0.1 | -0.2 | 0.1 | 0 | -0.1 | -0.7 | -0.3 | -0.8 | -0.5 | -1.8 | -1.9 | -1.8 | -0.7 | -0.1 | -0.1 | 0 | -0.2 | -0.2 | 0 | -0.1 | 0 | -0.3 | 0 | 0 | Encodes subunit O of photosystem I. |
| 758 | 248151\_at | AT5G54270 | LHCB3, LHCB3\*1 | 5 | -0.4 | -0.3 | -0.3 | -0.1 | -0.1 | -0.3 | 0 | -0.2 | -0.1 | -0.9 | -0.7 | -0.4 | -0.3 | -3.6 | -1.8 | -2.5 | -0.8 | -0.2 | 0 | 0 | -0.1 | -0.4 | 0 | -0.1 | 0 | -0.4 | 0.1 | 0.1 | Lhcb3 protein is a component of the main light harvesting chlorophyll a/b-protein complex of Photosystem II (LHC II)., Lhcb3 protein is a component of the main light harvesting chlorophyll a/b-protein complex of Photosystem II (LHC II). |
| 757 | 262557\_at | AT1G31330 | PSAF | 5 | -0.3 | -0.3 | -0.2 | -0.3 | 0 | -0.2 | 0.1 | -0.1 | 0 | -0.9 | -0.3 | -0.6 | -0.4 | -2.4 | -2 | -1.5 | -1.1 | -0.1 | -0.1 | -0.2 | -0.2 | -0.4 | -0.1 | -0.1 | 0 | -0.2 | 0.1 | 0.1 | Encodes subunit F of photosystem I. |
| 756 | 256309\_at | AT1G30380 | PSAK | 2 | -0.4 | -0.4 | -0.3 | -0.3 | 0 | -0.2 | 0 | -0.1 | -0.1 | -0.8 | -0.1 | -0.5 | -0.4 | -2.6 | -1.2 | -1.7 | -0.6 | -0.1 | -0.1 | -0.1 | -0.2 | -0.3 | 0 | -0.1 | -0.1 | -0.2 | 0.1 | 0 | Encodes subunit K of photosystem I reaction center. |
| 755 | 267002\_s\_at | AT2G34420, AT2G34430 AT2G34420, AT2G34430 | LHB1B2, LHCB1.5, LHB1B1, LHCB1.4 | 14 | -0.4 | -0.2 | -0.3 | -0.1 | 0 | -0.2 | 0.1 | -0.2 | 0.1 | -0.2 | -0.3 | -0.2 | 0 | -1.2 | -0.5 | -1.1 | -0.7 | -0.4 | 0 | 0.1 | 0 | -0.3 | -0.1 | -0.1 | 0 | -0.1 | 0.1 | 0.1 | Photosystem II type I chlorophyll a/b-binding protein, Photosystem II type I chlorophyll a/b-binding protein, Photosystem II type I chlorophyll a/b-binding protein, Photosystem II type I chlorophyll a/b-binding protein |
| 754 | 263345\_s\_at | AT2G05070, AT2G05100 AT2G05070, AT2G05100 | LHCB2, LHCB2.2, LHCB2, LHCB2.1 | 13 | -0.1 | -0.4 | -0.3 | -0.3 | -0.1 | -0.2 | 0 | -0.4 | -0.1 | -0.5 | -1 | -0.2 | -0.2 | -4.2 | -0.8 | -2.8 | -1.4 | -0.3 | 0 | 0 | -0.1 | -0.3 | 0 | -0.1 | -0.1 | -0.2 | 0.1 | 0 | Encodes Lhcb2.2. Belongs to the Lhc super-gene family encodes the light-harvesting chlorophyll a/b-binding (LHC) proteins that constitute the antenna system of the photosynthetic apparatus., Encodes Lhcb2.2. Belongs to the Lhc super-gene family encodes the light-harvesting chlorophyll a/b-binding (LHC) proteins that constitute the antenna system of the photosynthetic apparatus., Lhcb2.1 protein encoding a subunit of the light harvesting complex II. Member of a gene family with high degree of sequence similarity. Initially LHCB2.3 was considered as a separate gene but appears to be an allele of LHCB2.1., Lhcb2.1 protein encoding a subunit of the light harvesting complex II. Member of a gene family with high degree of sequence similarity. Initially LHCB2.3 was considered as a separate gene but appears to be an allele of LHCB2.1. |
| 753 | 251325\_s\_at | AT3G61470, AT5G28450 AT3G61470, AT5G28450 | LHCA2 | 10 | -0.2 | -0.4 | -0.4 | 0 | 0 | -0.3 | -0.1 | 0.1 | -0.1 | -0.4 | -0.2 | -0.2 | -0.2 | -0.8 | -0.9 | -0.7 | -0.4 | 0.1 | 0 | 0 | -0.1 | -0.3 | -0.1 | -0.1 | -0.3 | -0.1 | 0 | -0.1 | Encodes a component of the light harvesting antenna complex of photosystem I., chlorophyll A-B binding protein, chloroplast, putative / LHCI type II CAB, putative; similar to LHCA2 (Photosystem I light harvesting complex gene 2), chlorophyll binding [Arabidopsis thaliana] (TAIR:AT3G61470.1); similar to Chlorophyll a-b binding protein, chloroplast precursor (LHCI type II CAB) (GB:P13869); similar to Chlorophyll a-b binding protein 7, chloroplast precursor (LHCI type II CAB-7) (GB:P10708); contains InterPro domain Chlorophyll A-B binding protein; (InterPro:IPR001344) |
| 752 | 255331\_at | AT4G04330 |  |  | -1.3 | -0.5 | -0.8 | -0.1 | -0.1 | -0.6 | 0.2 | -0.1 | -0.8 | -1.3 | -0.4 | -2.3 | -2.4 | -2 | -4.6 | -2.2 | -1.3 | -2 | 0.3 | -0.4 | -0.6 | 0 | -0.2 | -1.1 | -0.9 | -1.8 | -0.6 | -0.9 | similar to unnamed protein product [Ostreococcus tauri] (GB:CAL56420.1); similar to Os08g0425200 [Oryza sativa (japonica cultivar-group)] (GB:NP\_001061837.1) |
| 751 | 250649\_at | AT5G06690 |  |  | -1.5 | -0.2 | 0 | -0.1 | -0.4 | -1.1 | 0.5 | 0.1 | -1.1 | -3.5 | -0.1 | -2.2 | -2 | -1.2 | -2.7 | -1.9 | -1.8 | -3.1 | 1.3 | 0.5 | -1.3 | 0.2 | 0 | -1.3 | -1.2 | -2.1 | -0.7 | -0.7 | (THIOREDOXIN-LIKE 5); thiol-disulfide exchange intermediate; Identical to Thioredoxin-like 5, chloroplast precursor [Arabidopsis Thaliana] (GB:Q9FG36;GB:Q9SWQ2;GB:Q9XFI2); similar to thioredoxin family protein [Arabidopsis thaliana] (TAIR:AT5G04260.1); similar to Thioredoxin-like 5, chloroplast precursor (GB:Q9FG36); contains InterPro domain Thioredoxin-like fold; (InterPro:IPR012336); contains InterPro domain Thioredoxin-related; (InterPro:IPR006662); contains InterPro domain Thioredoxin domain 2; (InterPro:IPR006663); contains InterPro domain Thioredoxin domain; (InterPro:IPR013766); contains InterPro domain Thioredoxin fold; (InterPro:IPR012335), (THIOREDOXIN-LIKE 5); thiol-disulfide exchange intermediate; Identical to Thioredoxin-like 5, chloroplast precursor [Arabidopsis Thaliana] (GB:Q9FG36;GB:Q9SWQ2;GB:Q9XFI2); similar to thioredoxin family protein [Arabidopsis thaliana] (TAIR:AT5G04260.1); similar to Thioredoxin-like 5, chloroplast precursor (GB:Q9FG36); contains InterPro domain Thioredoxin-like fold; (InterPro:IPR012336); contains InterPro domain Thioredoxin-related; (InterPro:IPR006662); contains InterPro domain Thioredoxin domain 2; (InterPro:IPR006663); contains InterPro domain Thioredoxin fold; (InterPro:IPR012335) |
| 750 | 248409\_at | AT5G51545 | LPA2 | 2 | -0.3 | -0.3 | -0.1 | -0.2 | -0.1 | -0.5 | 0 | 0 | -0.5 | -2 | -0.4 | -2.1 | -1.9 | -1.6 | -3.8 | -2.2 | -1.3 | -2.1 | 0 | 0.2 | -0.3 | -0.3 | -0.1 | -0.4 | -0.7 | -1.2 | -0.4 | -0.5 | similar to Os02g0125000 [Oryza sativa (japonica cultivar-group)] (GB:NP\_001045742.1); contains domain Subunit VIII of photosystem I reaction centre, PsaI (SSF81540) |
| 749 | 245321\_at | AT4G15545 |  |  | -0.3 | -0.2 | 0.1 | -0.4 | -0.2 | -0.2 | 0.1 | -0.1 | -0.2 | -0.6 | -0.2 | -1.1 | -0.5 | -1.2 | -1.5 | -1.5 | -1.4 | -0.4 | 0.1 | 0.1 | -0.4 | 0.2 | 0 | -0.6 | -0.7 | -1.3 | -0.5 | -0.5 | similar to unknown protein [Arabidopsis thaliana] (TAIR:AT1G16520.1); similar to unknown protein [Oryza sativa (japonica cultivar-group)] (GB:AAT85086.1); contains InterPro domain DNA topoisomerases I, dispensable insert, eukaryotic-type; (InterPro:IPR009054) |
| 748 | 266018\_at | AT2G18710 | SCY1 | 2 | -0.3 | 0 | 0.1 | -0.2 | 0.1 | -0.2 | 0 | -0.1 | -0.8 | -1.1 | -0.3 | -2.4 | -1.5 | -1.3 | -2.5 | -1.9 | -1.5 | -1.4 | -0.1 | -0.3 | -0.6 | 0.2 | -0.2 | -0.2 | -0.4 | -0.8 | -0.2 | -0.3 | SCY1 (SECY HOMOLOG 1); protein translocase; Identical to Preprotein translocase secY subunit, chloroplast precursor (CpSecY) (SECY) [Arabidopsis Thaliana] (GB:Q38885;GB:Q9SLG1); similar to EMB2289/SCY2 (EMBRYO DEFECTIVE 2289, SECY HOMOLOG 2), protein translocase [Arabidopsis thaliana] (TAIR:AT2G31530.1); similar to Preprotein translocase secY subunit, chloroplast precursor (CpSecY) (GB:O63066); similar to SecY homolog; targetted to the thylakoid membrane; the protein has a chloroplast targetting signal, but the processing site is not known (GB:AAB60305.1); similar to Preprotein translocase secY subunit, chloroplast precursor (CpSecY) (GB:P93690); contains InterPro domain SecY protein; (InterPro:IPR002208) |
| 747 | 248920\_at | AT5G45930 | CHL, CHLI-2, CHLI2 | 5 | -0.4 | -0.2 | 0 | -0.1 | 0 | -0.3 | 0 | -0.3 | -0.6 | -2.9 | -0.3 | -2.9 | -3 | -3 | -7.6 | -3.9 | -1.7 | -1.9 | 0 | -0.6 | -0.6 | -0.1 | -0.1 | -0.4 | -0.7 | -1.4 | -0.2 | -0.4 | encodes a second Chl I gene (CHLI2), a subunit of magnesium chelatase which is required for chlorophyll biosynthesis. Protein not detectable in wild type and is targeted for rapid degradation in vivo., encodes a second Chl I gene (CHLI2), a subunit of magnesium chelatase which is required for chlorophyll biosynthesis. Protein not detectable in wild type and is targeted for rapid degradation in vivo., encodes a second Chl I gene (CHLI2), a subunit of magnesium chelatase which is required for chlorophyll biosynthesis. Protein not detectable in wild type and is targeted for rapid degradation in vivo. |
| 746 | 248224\_at | AT5G53490 |  | 2 | -0.6 | 0 | 0.2 | -0.1 | 0 | -0.4 | 0.2 | 0 | -0.4 | -2.1 | -0.4 | -2.8 | -2.4 | -2.6 | -4.9 | -2.7 | -1.5 | -2.3 | -0.3 | -0.3 | -0.6 | 0.1 | -0.2 | -0.5 | -0.7 | -1 | -0.1 | -0.4 | thylakoid lumenal 17.4 kDa protein, chloroplast, identical to SP:P81760 Thylakoid lumenal 17.4 kDa protein, chloroplast precursor (P17.4) {Arabidopsis thaliana}. Putative pentapeptide protein. |
| 745 | 261078\_at | AT1G07320 | RPL4 | 6 | -0.4 | -0.1 | 0 | 0 | 0 | -0.4 | 0.1 | -0.2 | -0.4 | -2.8 | -0.4 | -2.4 | -1.9 | -2.6 | -4.2 | -2.8 | -1.3 | -1.8 | 0.1 | -0.1 | -0.3 | 0.1 | -0.1 | -0.1 | -0.2 | -0.6 | 0 | 0 | encodes a plastid ribosomal protein L4 |
| 744 | 256855\_at | AT3G15190 |  | 1 | -0.4 | -0.1 | -0.1 | -0.1 | 0 | -0.2 | 0.1 | -0.3 | -0.4 | -2.4 | -0.5 | -2.4 | -1.9 | -2.9 | -4.3 | -2.6 | -1.4 | -1.1 | 0.2 | -0.1 | -0.4 | 0 | -0.1 | -0.2 | -0.2 | -0.4 | 0 | -0.2 | chloroplast 30S ribosomal protein S20, putative; Identical to 30S ribosomal protein S20, chloroplast precursor (RPS20) [Arabidopsis Thaliana] (GB:Q9ASV6;GB:Q9LIL6); similar to Os01g0678600 [Oryza sativa (japonica cultivar-group)] (GB:NP\_001043859.1); similar to ribosomal protein rpS20 [Bigelowiella natans] (GB:AAP79183.1); contains InterPro domain Ribosomal protein S20; (InterPro:IPR002583); contains InterPro domain Ribosomal protein S20p; (InterPro:IPR010013) |
| 743 | 264383\_at | AT2G25080 | ATGPX1 | 5 | -0.5 | -0.2 | -0.1 | 0.2 | 0.1 | 0 | 0.3 | 0 | -0.3 | -1.1 | -0.3 | -1.8 | -1.2 | -1.6 | -2.6 | -1.8 | -1.2 | -1.2 | 0 | -0.2 | -0.7 | 0 | -0.2 | -0.3 | -0.1 | -0.7 | -0.2 | -0.4 | Encodes glutathione peroxidase. |
| 742 | 251036\_at | AT5G02160 |  | 1 | -0.7 | 0 | -0.1 | 0.1 | 0.1 | 0 | 0.3 | -0.2 | -0.9 | -4.8 | -0.5 | -1.8 | -2.1 | -3.6 | -4.8 | -3 | -2.6 | -1.6 | -0.2 | -0.4 | -0.9 | 0 | -0.2 | -0.2 | -0.2 | -0.8 | -0.4 | -0.6 | similar to hypothetical protein [Solanum lycopersicum] (GB:BAD95796.1) |
| 741 | 263624\_at | AT2G04700 |  |  | -0.5 | 0 | 0.1 | 0.1 | 0 | -0.1 | 0.1 | 0 | -0.1 | -0.4 | -0.2 | -1 | -0.7 | -1.3 | -1.4 | -1 | -0.7 | -0.9 | 0.1 | 0.1 | -0.4 | 0.1 | 0 | -0.3 | 0 | -0.5 | 0 | -0.2 | ferredoxin thioredoxin reductase catalytic beta chain family protein; similar to Ferredoxin-thioredoxin reductase catalytic chain, chloroplast precursor (FTR-C) (GB:O49856); contains InterPro domain Ferredoxin thioredoxin reductase, beta chain; (InterPro:IPR004209) |
| 740 | 259181\_at | AT3G01690 |  |  | -0.6 | 0 | 0 | -0.1 | -0.1 | -0.5 | 0.4 | -0.3 | -0.4 | -0.9 | -0.6 | -2.1 | -1.3 | -1.4 | -2.3 | -0.9 | -1.3 | -1.3 | -0.1 | -0.1 | -0.5 | -0.1 | 0 | -0.2 | -0.2 | -1.2 | -0.9 | -0.4 | similar to unknown protein [Arabidopsis thaliana] (TAIR:AT5G14390.1); similar to Esterase/lipase/thioesterase [Medicago truncatula] (GB:ABD32255.1); contains InterPro domain Esterase/lipase/thioesterase; (InterPro:IPR000379); contains InterPro domain Alpha/beta hydrolase fold-1; (InterPro:IPR000073) |
| 739 | 266813\_at | AT2G44920 |  | 3 | -0.8 | -0.1 | -0.1 | 0.1 | 0 | 0 | 0.4 | 0 | -0.8 | -1.6 | -0.2 | -1.9 | -1.3 | -2.3 | -3 | -2.8 | -2.2 | -1.9 | 0.4 | 0 | -0.2 | -0.1 | -0.2 | -0.3 | -0.2 | -0.9 | -0.1 | -0.2 | thylakoid lumenal 15 kDa protein, chloroplast; Identical to Thylakoid lumenal 15 kDa protein, chloroplast precursor (p15) [Arabidopsis Thaliana] (GB:O22160;GB:Q949R8); similar to FIP2 (FH protein interacting protein 2), voltage-gated potassium channel [Arabidopsis thaliana] (TAIR:AT5G55000.1); similar to hypothetical protein BL107\_13840 [Synechococcus sp. BL107] (GB:ZP\_01468001.1); similar to Os01g0144100 [Oryza sativa (japonica cultivar-group)] (GB:NP\_001042002.1); similar to hypothetical protein NAS141\_07590 [Sulfitobacter sp. NAS-14.1] (GB:ZP\_00964231.1); contains InterPro domain Pentapeptide repeat; (InterPro:IPR001646), thylakoid lumenal 15 kDa protein, chloroplast; Identical to Thylakoid lumenal 15 kDa protein, chloroplast precursor (p15) [Arabidopsis Thaliana] (GB:O22160;GB:Q949R8); similar to thylakoid lumenal protein-related [Arabidopsis thaliana] (TAIR:AT1G12250.1); similar to hypothetical protein BL107\_13840 [Synechococcus sp. BL107] (GB:ZP\_01468001.1); similar to Os01g0144100 [Oryza sativa (japonica cultivar-group)] (GB:NP\_001042002.1); contains InterPro domain Pentapeptide repeat; (InterPro:IPR001646) |
| 738 | 262288\_at | AT1G70760 | CRR23 | 1 | -1.2 | -0.3 | -0.4 | 0.1 | 0 | 0 | 0.3 | -0.1 | -1.2 | -2.5 | -0.4 | -3.4 | -2.2 | -2.3 | -6.1 | -4.9 | -2.1 | -1.7 | -0.3 | -0.1 | -0.5 | -0.2 | -0.3 | -0.6 | -0.4 | -1.7 | -0.2 | -0.4 | inorganic carbon transport protein-related; similar to NADH dehydrogenase subunit [Thermosynechococcus elongatus BP-1] (GB:NP\_681495.1); similar to Os05g0348100 [Oryza sativa (japonica cultivar-group)] (GB:NP\_001055267.1) |
| 737 | 249742\_at | AT5G24490 |  | 1 | -0.8 | 0 | -0.3 | -0.3 | 0 | 0.2 | 0.4 | -0.1 | -0.5 | -2.1 | -0.3 | -1.1 | -1.5 | -3.3 | -3.5 | -2.8 | -1.9 | -1.9 | 0.3 | 0 | -0.6 | 0.2 | 0 | -0.4 | -0.1 | -0.9 | -0.3 | -0.6 | 30S ribosomal protein, putative; similar to chloroplast-specific ribosomal protein [Lycopersicon esculentum] (GB:AAU03364.1); contains InterPro domain Rudiment single hybrid motif; (InterPro:IPR011054); contains InterPro domain Sigma 54 modulation protein/ribosomal protein S30EA; (InterPro:IPR003489) |
| 736 | 265149\_at | AT1G51400 |  | 2 | -0.6 | -0.2 | -0.9 | 0.3 | -0.1 | 0 | 0.4 | -0.1 | -0.5 | -1.3 | -0.3 | -0.7 | -0.7 | -3.9 | -2.3 | -2.6 | -1.5 | -0.6 | 0 | 0 | -0.3 | -0.5 | -0.3 | -0.4 | -0.5 | -1.2 | -0.1 | -0.4 | photosystem II 5 kD protein; similar to PSBTN (photosystem II subunit T) [Arabidopsis thaliana] (TAIR:AT3G21055.1); similar to unknown [Brassica rapa] (GB:ABD91572.1) |
| 735 | 258239\_at | AT3G27690 | LHCB2, LHCB2.3, LHCB2.4 | 4 | -1.3 | 0.2 | -0.7 | 0.7 | -0.1 | 0.3 | 0 | -0.2 | -1 | -3.7 | -0.7 | -2.7 | -3 | -8.2 | -6.6 | -5.1 | -4.3 | -1.3 | -0.4 | 0 | -0.2 | -0.6 | -0.5 | -0.6 | -1.9 | -2.2 | -0.8 | -1.3 | Encodes Lhcb2.4. Belongs to the Lhc super-gene family encodes the light-harvesting chlorophyll a/b-binding (LHC) proteins that constitute the antenna system of the photosynthetic apparatus., Encodes Lhcb2.4. Belongs to the Lhc super-gene family encodes the light-harvesting chlorophyll a/b-binding (LHC) proteins that constitute the antenna system of the photosynthetic apparatus., Encodes Lhcb2.4. Belongs to the Lhc super-gene family encodes the light-harvesting chlorophyll a/b-binding (LHC) proteins that constitute the antenna system of the photosynthetic apparatus. |
| 734 | 266279\_at | AT2G29290 |  |  | -1.3 | -0.3 | -0.1 | -0.3 | 0.1 | -0.7 | 0.4 | -0.3 | -1.5 | -4.4 | -0.9 | -3.1 | -2.2 | -3.9 | -4.9 | -5.4 | -3.5 | -2.8 | -0.4 | -0.1 | -0.5 | 0.5 | 0 | -1.8 | -2.3 | -2.2 | -1.4 | -1.5 | tropinone reductase, putative / tropine dehydrogenase, putative; similar to SAG13 (Senescence-associated gene 13), oxidoreductase [Arabidopsis thaliana] (TAIR:AT2G29350.1); similar to Tropinone reductase, putative, expressed [Oryza sativa (japonica cultivar-group)] (GB:ABF95188.1); contains InterPro domain Short-chain dehydrogenase/reductase SDR; (InterPro:IPR002198); contains InterPro domain Glucose/ribitol dehydrogenase; (InterPro:IPR002347) |
| 733 | 257172\_at | AT3G23700 |  | 2 | -0.5 | -0.1 | 0.1 | -0.1 | 0 | -0.3 | 0.1 | -0.1 | -0.6 | -1.8 | -0.3 | -1.6 | -1.7 | -1.9 | -3.4 | -2.7 | -1.8 | -1.7 | -0.3 | -0.3 | -0.2 | -0.1 | -0.3 | -0.4 | -0.7 | -1.4 | -0.3 | -0.4 | S1 RNA-binding domain-containing protein; similar to RPS1 (ribosomal protein S1), RNA binding [Arabidopsis thaliana] (TAIR:AT5G30510.1); similar to putative ribosomal S1 protein (with alternative splicing) [Oryza sativa (japonica cultivar-group)] (GB:AAS07358.1); contains InterPro domain Nucleic acid-binding, OB-fold, subgroup; (InterPro:IPR012340); contains InterPro domain Nucleic acid-binding, OB-fold; (InterPro:IPR008994); contains InterPro domain RNA binding S1; (InterPro:IPR003029) |
| 732 | 251919\_at | AT3G53800 |  |  | -1.8 | -0.2 | 1.3 | -0.4 | -0.1 | -0.5 | 0 | -0.4 | -1.5 | -2.8 | -0.4 | -2.6 | -2.5 | -6.5 | -4.5 | -4.8 | -4.2 | -3.3 | -2.1 | -1.1 | -1.4 | 0.8 | -0.7 | -1.1 | -1.4 | -2.1 | -1 | -1.3 | armadillo/beta-catenin repeat family protein; similar to armadillo/beta-catenin repeat family protein [Arabidopsis thaliana] (TAIR:AT3G09350.1); similar to Os03g0271400 [Oryza sativa (japonica cultivar-group)] (GB:NP\_001049691.1); similar to putative stress-induced protein [Solanum commersonii] (GB:CAJ19270.1); similar to Os03g0822700 [Oryza sativa (japonica cultivar-group)] (GB:NP\_001051736.1); contains InterPro domain Armadillo-like helical; (InterPro:IPR011989); contains InterPro domain HEAT; (InterPro:IPR000357) |
| 731 | 251701\_at | AT3G56650 |  | 3 | -0.6 | -0.1 | 0.2 | 0.1 | -0.1 | -0.2 | 0 | 0.1 | -0.3 | -2.6 | -0.3 | -1.8 | -2 | -2.8 | -3.3 | -2.4 | -1.9 | -1.7 | -0.5 | -0.1 | -0.6 | 0.3 | -0.1 | -0.3 | -0.4 | -0.9 | -0.2 | -0.2 | thylakoid lumenal 20 kDa protein; similar to thylakoid lumenal 29.8 kDa protein [Arabidopsis thaliana] (TAIR:AT1G77090.1); similar to Os12g0190200 [Oryza sativa (japonica cultivar-group)] (GB:NP\_001066340.1); similar to Os01g0805300 [Oryza sativa (japonica cultivar-group)] (GB:NP\_001044559.1); similar to thylakoid lumenal 29.8 kDa protein, putative, expressed [Oryza sativa (japonica cultivar-group)] (GB:ABA96032.2) |
| 730 | 266277\_at | AT2G29310 |  |  | -1.7 | -0.3 | 0.2 | -0.3 | 0.2 | -0.1 | 0.1 | -0.2 | -1.1 | -2.3 | -0.4 | -1.4 | -1.8 | -3.4 | -3 | -4.6 | -2.9 | -1.2 | -1.1 | -0.3 | -1.2 | 0.4 | 0.2 | -1.8 | -1.5 | -2.2 | -0.6 | -1.3 | tropinone reductase, putative / tropine dehydrogenase, putative; similar to tropinone reductase, putative / tropine dehydrogenase, putative [Arabidopsis thaliana] (TAIR:AT2G29320.1); similar to tropinone reductase, putative / tropine dehydrogenase, putative [Arabidopsis thaliana] (TAIR:AT2G29300.1); similar to Tropinone reductase, putative, expressed [Oryza sativa (japonica cultivar-group)] (GB:ABF95188.1); contains InterPro domain Short-chain dehydrogenase/reductase SDR; (InterPro:IPR002198); contains InterPro domain Glucose/ribitol dehydrogenase; (InterPro:IPR002347) |
| 729 | 264837\_at | AT1G03600 |  | 4 | -0.6 | -0.1 | -0.3 | 0.2 | 0 | -0.2 | 0.1 | -0.1 | -0.1 | -1 | -0.4 | -1.3 | -0.9 | -2.7 | -2.9 | -3 | -1.2 | -0.8 | -0.2 | -0.1 | -0.2 | -0.1 | -0.1 | -0.3 | -0.4 | -0.6 | 0 | -0.3 | photosystem II family protein; similar to photosystem II 11 kD protein [Thermosynechococcus elongatus BP-1] (GB:NP\_683253.1); similar to Os03g0333400 [Oryza sativa (japonica cultivar-group)] (GB:NP\_001050024.1) |
| 728 | 246199\_at | AT4G36530 |  | 1 | -0.8 | -0.3 | -0.4 | -0.1 | -0.2 | -0.2 | 0 | -0.2 | -0.6 | -0.7 | -0.4 | -1.6 | -1.2 | -1.1 | -1.5 | -2.2 | -1.3 | -1 | -0.3 | -0.3 | -0.7 | 0 | -0.2 | -0.6 | -0.6 | -0.9 | -0.1 | -0.1 | hydrolase, alpha/beta fold family protein; similar to hydrolase, alpha/beta fold family protein [Arabidopsis thaliana] (TAIR:AT5G19850.1); similar to hydrolase, alpha/beta fold family [Synechococcus sp. JA-3-3Ab] (GB:YP\_473970.1); similar to Os02g0705100 [Oryza sativa (japonica cultivar-group)] (GB:NP\_001047865.1); contains InterPro domain Esterase/lipase/thioesterase; (InterPro:IPR000379); contains InterPro domain Alpha/beta hydrolase fold-1; (InterPro:IPR000073); contains InterPro domain Epoxide hydrolase; (InterPro:IPR000639); contains InterPro domain Alpha/beta hydrolase; (InterPro:IPR003089) |
| 727 | 260968\_at | AT1G12250 |  | 3 | -0.5 | -0.5 | -1 | 0.1 | 0.1 | -0.2 | 0.1 | -0.3 | -0.7 | -1.7 | -0.1 | -1.4 | -1.4 | -1.5 | -2 | -2.4 | -2.3 | -1.4 | -0.3 | -0.2 | -0.5 | -0.1 | -0.5 | -0.5 | -0.4 | -0.6 | 0 | -0.3 | thylakoid lumenal protein-related; similar to thylakoid lumenal 15 kDa protein, chloroplast [Arabidopsis thaliana] (TAIR:AT2G44920.2); similar to Os02g0643500 [Oryza sativa (japonica cultivar-group)] (GB:NP\_001047560.1); similar to thylakoid lumenal protein-like [Oryza sativa (ISS) [Ostreococcus tauri] (GB:CAL56824.1); contains InterPro domain Pentapeptide repeat; (InterPro:IPR001646) |
| 726 | 264799\_at | AT1G08550 | AVDE1, NPQ1 | 29 | -0.8 | -0.8 | -0.5 | 0 | -0.2 | -0.6 | -0.1 | -0.3 | -0.5 | -1.7 | -0.4 | -2 | -1.9 | -2.6 | -2.5 | -1.9 | -2.1 | -2 | -0.3 | -0.5 | -0.4 | -0.1 | -0.9 | -1.2 | -0.8 | -1.7 | -0.4 | -0.6 | Violaxanthin deepoxidase involved in xanthophyll cycle.Two major consequences of the npq1 mutation are the absence of zeaxanthin formation in strong light and the partial inhibition of the quenching of singlet excited chlorophylls in the photosystem II light-harvesting complex, Violaxanthin deepoxidase involved in xanthophyll cycle.Two major consequences of the npq1 mutation are the absence of zeaxanthin formation in strong light and the partial inhibition of the quenching of singlet excited chlorophylls in the photosystem II light-harvesting complex |
| 725 | 254669\_at | AT4G18370 | DEG5, DEGP5, HHOA | 5 | -0.7 | -0.1 | -0.1 | 0.1 | 0.2 | -0.5 | -0.2 | 0.2 | -0.5 | -3.1 | -0.2 | -2.3 | -1.6 | -1.9 | -4.6 | -2.7 | -1.8 | -0.7 | 0.1 | -0.3 | -0.6 | -0.1 | -0.3 | -0.4 | -0.3 | -1.6 | 0 | -0.2 | Encodes a putative protease HhoA precursor (HhoA)., Encodes a putative protease HhoA precursor (HhoA)., Encodes a putative protease HhoA precursor (HhoA). |
| 724 | 250133\_at | AT5G16400 | ATF2, TRXF2 | 3 | -0.9 | 0 | 0.1 | 0.2 | 0.1 | 0.1 | 0 | 0.4 | -0.5 | -1.5 | 0 | -1.4 | -1.2 | -2.8 | -2.5 | -2.3 | -1.7 | -1.5 | -0.1 | -0.2 | -0.1 | 0 | -0.2 | -0.3 | -0.2 | -1.1 | 0.1 | -0.2 | ATF2/TRXF2 (THIOREDOXIN F2); thiol-disulfide exchange intermediate; Identical to Thioredoxin F-type 2, chloroplast precursor (TRX-F2) [Arabidopsis Thaliana] (GB:Q9XFH9); similar to ATF1/TRXF1 (THIOREDOXIN F-TYPE 1), thiol-disulfide exchange intermediate [Arabidopsis thaliana] (TAIR:AT3G02730.1); similar to Thioredoxin F-type, chloroplast precursor (TRX-F) (GB:O48897); contains InterPro domain Thioredoxin-like fold; (InterPro:IPR012336); contains InterPro domain Thioredoxin-related; (InterPro:IPR006662); contains InterPro domain Thioredoxin domain 2; (InterPro:IPR006663); contains InterPro domain Thioredoxin domain; (InterPro:IPR013766); contains InterPro domain Thioredoxin fold; (InterPro:IPR012335), ATF2/TRXF2 (THIOREDOXIN F2); thiol-disulfide exchange intermediate; Identical to Thioredoxin F-type 2, chloroplast precursor (TRX-F2) [Arabidopsis Thaliana] (GB:Q9XFH9); similar to ATF1/TRXF1 (THIOREDOXIN F-TYPE 1), thiol-disulfide exchange intermediate [Arabidopsis thaliana] (TAIR:AT3G02730.1); similar to Thioredoxin F-type, chloroplast precursor (TRX-F) (GB:O48897); contains InterPro domain Thioredoxin-like fold; (InterPro:IPR012336); contains InterPro domain Thioredoxin-related; (InterPro:IPR006662); contains InterPro domain Thioredoxin domain 2; (InterPro:IPR006663); contains InterPro domain Thioredoxin domain; (InterPro:IPR013766); contains InterPro domain Thioredoxin fold; (InterPro:IPR012335) |
| 723 | 262202\_at | AT2G01110 | APG2, PGA2, TATC, UNE3 | 6 | -0.8 | -0.5 | -0.3 | -0.1 | -0.2 | -0.3 | 0.1 | -0.1 | -0.5 | -0.8 | -0.2 | -1.6 | -1.2 | -0.5 | -1.4 | -1.5 | -1.7 | -1.3 | -0.5 | -0.3 | -0.4 | -0.1 | -0.3 | -0.5 | -0.4 | -1.1 | -0.2 | -0.5 | mutant is Albino and pale green; Chloroplast Protein Translocation (tatC), mutant is Albino and pale green; Chloroplast Protein Translocation (tatC), mutant is Albino and pale green; Chloroplast Protein Translocation (tatC), mutant is Albino and pale green; Chloroplast Protein Translocation (tatC) |
| 722 | 247523\_at | AT5G61410 | RPE, emb2728 | 4 | -0.5 | -0.2 | 0 | 0 | -0.1 | 0 | 0.1 | 0.1 | -0.2 | -0.7 | -0.2 | -1.2 | -0.8 | -0.7 | -1.2 | -1.7 | -1.4 | -0.9 | 0 | -0.2 | -0.1 | -0.1 | -0.1 | -0.2 | -0.1 | -0.4 | 0 | -0.1 | Arabidopsis thaliana ribulose-5-phosphate-3-epimerase mRNA, Arabidopsis thaliana ribulose-5-phosphate-3-epimerase mRNA |
| 721 | 264158\_at | AT1G65260 | PTAC4 | 4 | -0.6 | -0.3 | 0 | 0.1 | 0 | 0 | 0.1 | 0 | -0.4 | -0.9 | -0.2 | -1.3 | -1 | -0.7 | -1 | -1.2 | -1.2 | -1.3 | -0.2 | -0.1 | -0.3 | 0 | -0.2 | -0.1 | -0.1 | -0.4 | 0 | 0.1 | PTAC4 (PLASTID TRANSCRIPTIONALLY ACTIVE4); Identical to Probable membrane-associated 30 kDa protein, chloroplast precursor [Arabidopsis Thaliana] (GB:O80796); similar to Membrane-associated 30 kDa protein, chloroplast precursor (M30) (GB:Q03943); similar to Os01g0895100 [Oryza sativa (japonica cultivar-group)] (GB:NP\_001045073.1); contains InterPro domain PspA/IM30; (InterPro:IPR007157) |
| 720 | 249524\_at | AT5G38520 |  | 1 | -1.3 | -0.3 | -0.1 | 0.2 | -0.2 | -0.2 | 0.2 | 0 | -1 | -2.5 | -0.2 | -2.5 | -1.8 | -2.5 | -3.7 | -4.1 | -2.6 | -1.5 | -0.6 | -0.5 | -0.7 | -0.3 | -0.4 | -0.4 | -0.3 | -1.2 | -0.1 | 0 | hydrolase, alpha/beta fold family protein; similar to hydrolase, alpha/beta fold family protein [Arabidopsis thaliana] (TAIR:AT5G19850.1); similar to Os05g0419000 [Oryza sativa (japonica cultivar-group)] (GB:NP\_001055569.1); similar to Predicted hydrolase/acyltransferase (alpha/beta hydrolase superfamily) (ISS) [Ostreococcus tauri] (GB:CAL57254.1); contains InterPro domain Esterase/lipase/thioesterase; (InterPro:IPR000379); contains InterPro domain Alpha/beta hydrolase fold-1; (InterPro:IPR000073), hydrolase, alpha/beta fold family protein; similar to hydrolase, alpha/beta fold family protein [Arabidopsis thaliana] (TAIR:AT5G19850.1); similar to Os05g0419000 [Oryza sativa (japonica cultivar-group)] (GB:NP\_001055569.1); similar to Predicted hydrolase/acyltransferase (alpha/beta hydrolase superfamily) (ISS) [Ostreococcus tauri] (GB:CAL57254.1); contains InterPro domain Esterase/lipase/thioesterase; (InterPro:IPR000379); contains InterPro domain Alpha/beta hydrolase fold-1; (InterPro:IPR000073); contains InterPro domain Alpha/beta hydrolase; (InterPro:IPR003089) |
| 719 | 252463\_at | AT3G47070 |  | 3 | -0.6 | -0.4 | 0 | 0.3 | 0 | -0.3 | -0.1 | -0.1 | -0.4 | -1.6 | -0.3 | -1.6 | -0.9 | -1.3 | -3.2 | -2.3 | -2.6 | -0.9 | 0 | -0.2 | -0.3 | -0.1 | -0.3 | -0.5 | -0.5 | -1.1 | -0.2 | -0.3 | similar to thylakoid soluble phosphoprotein [Spinacia oleracea] (GB:CAD45559.1) |
| 718 | 249658\_s\_at | AT5G36700, AT5G36790 AT5G36700, AT5G36790 | ATPGLP1, PGLP1 | 2 | -0.8 | -0.2 | 0 | 0.1 | 0 | -0.3 | -0.2 | 0 | -0.4 | -2.3 | -0.3 | -1.8 | -1.2 | -1.1 | -3.2 | -2.5 | -1.3 | -0.7 | 0 | 0.2 | 0 | 0 | -0.3 | -0.3 | -0.1 | -1 | 0.1 | -0.1 | phosphoglycolate phosphatase, putative; similar to phosphoglycolate phosphatase, putative [Arabidopsis thaliana] (TAIR:AT5G36790.1); similar to HAD-superfamily hydrolase, subfamily IIA [Medicago truncatula] (GB:ABE78003.1); similar to OSIGBa0130B08.3 [Oryza sativa (indica cultivar-group)] (GB:CAH67343.1); similar to Os04g0490800 [Oryza sativa (japonica cultivar-group)] (GB:NP\_001053167.1); contains InterPro domain HAD-superfamily hydrolase, subfamily IIA; (InterPro:IPR006357); contains InterPro domain 2-phosphoglycolate phosphatase, eukaryotic; (InterPro:IPR006349); contains InterPro domain Haloacid dehalogenase-like hydrolase; (InterPro:IPR005834), phosphoglycolate phosphatase, putative; similar to phosphoglycolate phosphatase, putative [Arabidopsis thaliana] (TAIR:AT5G36790.1); similar to HAD-superfamily hydrolase, subfamily IIA [Medicago truncatula] (GB:ABE78003.1); similar to OSIGBa0130B08.3 [Oryza sativa (indica cultivar-group)] (GB:CAH67343.1); similar to Os04g0490800 [Oryza sativa (japonica cultivar-group)] (GB:NP\_001053167.1); contains InterPro domain HAD-superfamily hydrolase, subfamily IIA; (InterPro:IPR006357); contains InterPro domain 2-phosphoglycolate phosphatase, eukaryotic; (InterPro:IPR006349); contains InterPro domain Haloacid dehalogenase-like hydrolase; (InterPro:IPR005834), phosphoglycolate phosphatase, putative; similar to phosphoglycolate phosphatase, putative [Arabidopsis thaliana] (TAIR:AT5G36700.1); similar to HAD-superfamily hydrolase, subfamily IIA [Medicago truncatula] (GB:ABE78003.1); similar to OSIGBa0130B08.3 [Oryza sativa (indica cultivar-group)] (GB:CAH67343.1); similar to Os04g0490800 [Oryza sativa (japonica cultivar-group)] (GB:NP\_001053167.1); contains InterPro domain HAD-superfamily hydrolase, subfamily IIA; (InterPro:IPR006357); contains InterPro domain 2-phosphoglycolate phosphatase, eukaryotic; (InterPro:IPR006349); contains InterPro domain Haloacid dehalogenase-like hydrolase; (InterPro:IPR005834) |
| 717 | 262878\_at | AT1G64770 | NDF2 | 1 | -1.2 | -0.5 | 0.1 | 0.2 | -0.1 | -0.5 | -0.2 | -0.1 | -0.6 | -2.7 | -0.2 | -2 | -1.3 | -1.1 | -4.8 | -3.2 | -1.9 | -1.1 | -0.5 | -0.1 | -0.1 | -0.3 | -0.4 | -0.3 | -0.6 | -1.6 | 0 | -0.2 | similar to unknown protein [Arabidopsis thaliana] (TAIR:AT1G55370.2); similar to Os03g0158300 [Oryza sativa (japonica cultivar-group)] (GB:NP\_001049021.1); similar to Hypothetical protein [Oryza sativa (japonica cultivar-group)] (GB:AAN06836.1); contains InterPro domain Galactose mutarotase-like; (InterPro:IPR011013) |
| 716 | 250073\_at | AT5G17170 | ENH1, ENH1 | 4 | -0.7 | -0.7 | 0.3 | -0.1 | 0 | -0.2 | -0.2 | -0.2 | -0.6 | -1.6 | -0.4 | -1.8 | -1.3 | -0.6 | -2.4 | -1.9 | -2.9 | -1 | -0.2 | -0.2 | 0 | 0.1 | -0.3 | -0.3 | -0.6 | -2 | -0.2 | -0.3 | rubredoxin family protein; similar to binding / protein binding [Arabidopsis thaliana] (TAIR:AT1G55480.1); similar to Os08g0162600 [Oryza sativa (japonica cultivar-group)] (GB:NP\_001061060.1); similar to PREDICTED P0577B11.138 gene product [Oryza sativa (ISS) [Ostreococcus tauri] (GB:CAL53651.1); contains InterPro domain PDZ/DHR/GLGF; (InterPro:IPR001478), rubredoxin family protein; similar to rubredoxin family protein [Arabidopsis thaliana] (TAIR:AT5G51010.1); similar to Os08g0162600 [Oryza sativa (japonica cultivar-group)] (GB:NP\_001061060.1); similar to PREDICTED P0577B11.138 gene product [Oryza sativa (ISS) [Ostreococcus tauri] (GB:CAL53651.1); contains InterPro domain PDZ/DHR/GLGF; (InterPro:IPR001478); contains InterPro domain Rubredoxin-type Fe(Cys)4 protein; (InterPro:IPR004039) |
| 715 | 265569\_at | AT2G05620 | PGR5 | 8 | -0.5 | -0.2 | 0 | 0.1 | -0.1 | -0.2 | 0 | -0.2 | -0.7 | -0.4 | -0.5 | -1.1 | -0.6 | -0.9 | -0.7 | -2.2 | -1.7 | -0.2 | -0.2 | -0.1 | -0.1 | 0 | -0.2 | -0.4 | -0.6 | -0.9 | -0.1 | -0.2 | Involved in electron flow in Photosystem I. Essential for photoprotection. |
| 714 | 264845\_at | AT1G03680 | ATHM1, ATM1, TRX-M1 | 6 | -0.5 | -0.2 | -0.2 | 0.2 | -0.1 | 0 | 0 | -0.1 | -0.5 | -1 | -0.2 | -1.9 | -1.1 | -0.7 | -2.6 | -1.6 | -1.4 | -1.2 | 0.1 | 0.1 | 0 | 0 | -0.2 | -0.4 | -0.1 | -0.7 | 0.1 | -0.2 | encodes a chloroplast thioredoxin similar to prokaryotic thioredoxins., encodes a chloroplast thioredoxin similar to prokaryotic thioredoxins., encodes a chloroplast thioredoxin similar to prokaryotic thioredoxins. |
| 713 | 250498\_at | AT5G09660 | PMDH2 | 3 | -0.8 | -0.3 | 0 | 0.1 | -0.1 | -0.1 | 0.1 | 0 | -0.2 | -1.2 | -0.1 | -1.5 | -0.9 | -0.8 | -3.1 | -2.7 | -1.7 | -1.1 | 0 | 0.1 | -0.1 | -0.1 | -0.2 | -0.6 | -0.2 | -0.8 | 0 | 0 | encodes a microbody NAD-dependent malate dehydrogenase encodes an peroxisomal NAD-malate dehydrogenase that is involved in fatty acid beta-oxidation through providing NAD to the process of converting fatty acyl CoA to acetyl CoA. |
| 712 | 251810\_at | AT3G55250 |  | 2 | -0.3 | -0.2 | -0.1 | 0.1 | -0.1 | 0 | 0.2 | -0.2 | -0.4 | -1 | -0.3 | -1.7 | -1.5 | -1.1 | -2.6 | -1.7 | -1.6 | -1.8 | 0 | -0.1 | 0.2 | 0.5 | 0 | -0.7 | -0.3 | -1 | 0.4 | 0 | similar to calcium homeostasis regulator CHoR1 [Solanum tuberosum] (GB:AAQ14193.1) |
| 711 | 262483\_at | AT1G17220 | FUG1 | 2 | 0.1 | -0.1 | 0 | 0.2 | -0.1 | -0.6 | 0 | -0.1 | -0.4 | -1.2 | -0.1 | -1.3 | -0.8 | -0.7 | -2.3 | -1.6 | -1.3 | -1.8 | 0 | 0 | -0.5 | 0 | -0.3 | -0.3 | -0.2 | -1.2 | 0.3 | 0 | translation initiation factor IF-2, chloroplast, putative; Identical to Translation initiation factor IF-2, chloroplast precursor [Arabidopsis Thaliana] (GB:Q9SHI1); similar to translation initiation factor IF-2, mitochondrial, putative [Arabidopsis thaliana] (TAIR:AT4G11160.1); similar to translation initiation factor IF-2 Small GTP-binding protein domain [Synechococcus elongatus PCC 7942] (GB:YP\_401037.1); similar to Os05g0575300 [Oryza sativa (japonica cultivar-group)] (GB:NP\_001056396.1); similar to Translation initiation factor IF-2, chloroplast precursor (PvIF2cp) (GB:P57997); contains InterPro domain Small GTP-binding protein domain; (InterPro:IPR005225); contains InterPro domain Translation factor; (InterPro:IPR009000); contains InterPro domain Elongation factor Tu, domain 2; (InterPro:IPR004161); contains InterPro domain Translation initiation factor IF-2, N-terminal; (InterPro:IPR006847); contains InterPro domain Initiation factor 2; (InterPro:IPR000178); contains InterPro domain Protein synthesis factor, GTP-binding; (InterPro:IPR000795) |
| 710 | 261338\_at | AT1G44920 |  | 1 | 0.1 | -0.3 | 0 | 0 | -0.2 | -0.4 | 0.1 | -0.2 | -0.6 | -1.3 | -0.4 | -2 | -1.5 | -1.7 | -2.8 | -2 | -2.1 | -1.5 | 0 | 0 | -0.3 | -0.1 | -0.2 | -0.4 | -0.5 | -1.5 | -0.1 | -0.2 | similar to unknown protein [Oryza sativa (japonica cultivar-group)] (GB:AAO38479.1) |
| 709 | 267130\_at | AT2G23390 |  |  | -0.4 | 0 | -0.2 | 0.2 | 0.1 | -0.2 | -0.3 | 0 | -0.7 | -0.9 | -0.3 | -1 | -0.7 | -0.3 | -1.3 | -1.2 | -1.2 | -1.5 | -0.5 | -0.4 | -0.6 | -0.1 | -0.4 | -0.3 | -0.7 | -1.5 | 0.1 | -0.5 | similar to Os04g0442200 [Oryza sativa (japonica cultivar-group)] (GB:NP\_001052881.1); similar to P0076O17.10 [Oryza sativa (japonica cultivar-group)] (GB:CAE02512.1); contains InterPro domain Protein of unknown function DUF482; (InterPro:IPR007434) |
| 708 | 249247\_at | AT5G42310 |  | 1 | -0.1 | -0.3 | 0 | 0 | -0.3 | -0.4 | -0.2 | -0.1 | -0.2 | -1.1 | 0 | -1.6 | -1.1 | -0.3 | -1.8 | -1.7 | -1.8 | -0.8 | -0.4 | -0.4 | -0.8 | -0.2 | -0.5 | -0.5 | -0.8 | -1.7 | -0.3 | -0.5 | pentatricopeptide (PPR) repeat-containing protein; similar to pentatricopeptide (PPR) repeat-containing protein [Arabidopsis thaliana] (TAIR:AT3G22470.1); similar to CRP1 [Zea mays] (GB:AAC25599.1); similar to Os07g0548300 [Oryza sativa (japonica cultivar-group)] (GB:NP\_001059927.1); contains InterPro domain Pentatricopeptide repeat; (InterPro:IPR002885); contains InterPro domain Protein prenyltransferase; (InterPro:IPR008940); contains InterPro domain Tetratricopeptide-like helical; (InterPro:IPR011990) |
| 707 | 252543\_at | AT3G45780 | JK224, NPH1, PHOT1, RPT1 | 72 | 0.2 | 0 | -0.7 | 0 | 0 | -0.6 | 0.2 | -0.2 | -0.1 | -1.7 | -0.3 | -1.7 | -1.4 | -1 | -2.4 | -2.4 | -2 | -1.8 | -0.4 | -0.4 | -0.7 | -0.4 | 0 | -0.8 | -1.3 | -2.6 | -0.6 | -1 | Blue-light photoreceptor. Contains a light activated serine-threonine kinase domain and LOV1 and LOV2 repeats. Mutants are defective in blue-light response. Mediates blue light-induced growth enhancements. PHOT1 and PHOT2 mediate blue light-dependent activation of the plasma membrane H+-ATPase in guard cell protoplasts., Blue-light photoreceptor. Contains a light activated serine-threonine kinase domain and LOV1 and LOV2 repeats. Mutants are defective in blue-light response. Mediates blue light-induced growth enhancements. PHOT1 and PHOT2 mediate blue light-dependent activation of the plasma membrane H+-ATPase in guard cell protoplasts., Blue-light photoreceptor. Contains a light activated serine-threonine kinase domain and LOV1 and LOV2 repeats. Mutants are defective in blue-light response. Mediates blue light-induced growth enhancements. PHOT1 and PHOT2 mediate blue light-dependent activation of the plasma membrane H+-ATPase in guard cell protoplasts., Blue-light photoreceptor. Contains a light activated serine-threonine kinase domain and LOV1 and LOV2 repeats. Mutants are defective in blue-light response. Mediates blue light-induced growth enhancements. PHOT1 and PHOT2 mediate blue light-dependent activation of the plasma membrane H+-ATPase in guard cell protoplasts. |
| 706 | 252092\_at | AT3G51420 | ATSSL4, SSL4 |  | 0.2 | -0.6 | -0.6 | 0.1 | 0.2 | -0.3 | 0 | 0 | -0.5 | -1.1 | -0.2 | -0.9 | -0.6 | -0.3 | -0.8 | -1.7 | -2.2 | -0.7 | -0.2 | -0.4 | -0.4 | 0 | -0.3 | -0.6 | -0.7 | -1.7 | 0 | -0.5 | strictosidine synthase family protein; similar to YLS2 (yellow-leaf-specific gene 2), strictosidine synthase [Arabidopsis thaliana] (TAIR:AT3G51430.1); similar to mucin-like protein [Oryza sativa] (GB:AAG13594.1); contains InterPro domain Strictosidine synthase; (InterPro:IPR004141), strictosidine synthase family protein; similar to YLS2 (yellow-leaf-specific gene 2), strictosidine synthase [Arabidopsis thaliana] (TAIR:AT3G51430.1); similar to mucin-like protein [Oryza sativa] (GB:AAG13594.1); contains InterPro domain Strictosidine synthase; (InterPro:IPR004141) |
| 705 | 267471\_at | AT2G30390 | FC2 | 2 | 0.1 | -0.4 | -0.7 | 0 | -0.2 | -0.1 | -0.2 | -0.1 | -0.1 | -1.1 | -0.2 | -1.8 | -1.3 | -0.5 | -2.4 | -1.7 | -1.4 | -0.8 | -0.5 | -0.4 | -0.8 | -0.2 | -0.3 | -0.4 | -0.8 | -1.5 | -0.1 | -0.4 | ferrochelatase II; Identical to Ferrochelatase-2, chloroplast precursor (EC 4.99.1.1) (Ferrochelatase II) (Protoheme ferro-lyase 2) (Heme synthetase 2) [Arabidopsis Thaliana] (GB:O04921;GB:O23623;GB:Q7GB39); similar to ferrochelatase [Arabidopsis thaliana] (TAIR:AT5G26030.2); similar to ferrochelatase [Nicotiana tabacum] (GB:CAC50871.1); similar to ferrochelatase [Oryza sativa] (GB:BAA22284.1); similar to ferrochelatase [Cucumis sativus] (GB:BAB20760.1); contains InterPro domain Ferrochelatase; (InterPro:IPR001015) |
| 704 | 264195\_at | AT1G22690 |  |  | -0.2 | -0.6 | -0.5 | 0.6 | -0.3 | -0.1 | -0.7 | 0 | 0.2 | -8.6 | -0.3 | -2.6 | -1.6 | -5.3 | -8.9 | -2.3 | -4.6 | -1.1 | -0.9 | -0.3 | -0.5 | -0.7 | -0.3 | -0.4 | -1.2 | -0.6 | 0.5 | -0.3 | gibberellin-responsive protein, putative; similar to GASA2 (GAST1 PROTEIN HOMOLOG 2) [Arabidopsis thaliana] (TAIR:AT4G09610.1); similar to snakin2 [Solanum tuberosum] (GB:CAC44012.1); contains InterPro domain Gibberellin regulated protein; (InterPro:IPR003854) |
| 703 | 251155\_at | AT3G63160 |  | 3 | -0.2 | 0 | 0.1 | 0.5 | -0.3 | -0.3 | -0.2 | -0.1 | -1 | -1.4 | -0.5 | -2.8 | -2.9 | -2.4 | -5.8 | -1.8 | -0.6 | -3.1 | -0.2 | 0.2 | 0 | 0 | -0.2 | -0.1 | 0.3 | -1.7 | 0.2 | 0.2 | similar to outer envelope membrane protein, putative [Arabidopsis thaliana] (TAIR:AT3G52420.1); similar to chloroplast outer envelope membrane protein [Erysimum cheiri] (GB:AAK52964.1) |
| 702 | 247101\_at | AT5G66530 |  | 1 | -0.5 | 0 | -0.1 | 0.1 | -0.3 | -0.3 | -0.2 | -0.4 | -0.4 | -3.9 | -0.7 | -3.2 | -2.4 | -3.1 | -5.1 | -1.9 | -1.6 | -1.9 | -0.6 | 0 | -0.3 | 0.1 | -0.1 | -0.3 | 0.1 | -0.8 | 0.2 | 0.1 | aldose 1-epimerase family protein; similar to aldose 1-epimerase family protein [Arabidopsis thaliana] (TAIR:AT5G57330.1); similar to Os09g0327400 [Oryza sativa (japonica cultivar-group)] (GB:NP\_001062890.1); similar to apospory-associated protein C; APOC [Chlamydomonas reinhardtii] (GB:AAF34174.1); contains InterPro domain Galactose mutarotase-like; (InterPro:IPR011013); contains InterPro domain Aldose 1-epimerase; (InterPro:IPR008183) |
| 701 | 250867\_at | AT5G03880 |  | 2 | -0.4 | 0.1 | 0.2 | 0.3 | -0.1 | 0 | 0.1 | 0 | -0.5 | -1.1 | -0.4 | -2.1 | -1.4 | -0.2 | -1.7 | -1.1 | -1.1 | -1.6 | -0.6 | -0.1 | -0.3 | 0.1 | 0 | 0.1 | 0.5 | -0.4 | 0.3 | 0.2 | similar to unknown protein [Arabidopsis thaliana] (TAIR:AT4G10000.2); similar to Os08g0558200 [Oryza sativa (japonica cultivar-group)] (GB:NP\_001062492.1); similar to unnamed protein product [Ostreococcus tauri] (GB:CAL56846.1); contains InterPro domain Thioredoxin-like fold; (InterPro:IPR012336); contains InterPro domain Thioredoxin fold; (InterPro:IPR012335) |
| 700 | 245291\_at | AT4G16155 |  | 3 | -0.1 | -0.3 | -0.1 | 0.2 | -0.1 | -0.2 | -0.1 | -0.5 | -0.5 | -2.5 | -1 | -2.2 | -3.2 | -3.1 | -4.6 | -1.2 | -2 | -1.3 | -0.1 | -0.3 | -0.2 | 0 | -0.1 | 0.1 | 0.1 | -0.5 | 0.1 | 0.1 | dihydrolipoamide dehydrogenase 2, plastidic / lipoamide dehydrogenase 2 (PTLPD2); similar to LPD1 (LIPOAMIDE DEHYDROGENASE 1) [Arabidopsis thaliana] (TAIR:AT3G16950.1); similar to Pyridine nucleotide-disulphide oxidoreductase, class I [Medicago truncatula] (GB:ABD32316.1); similar to Pyridine nucleotide-disulphide oxidoreductase, class I [Medicago truncatula] (GB:ABE83195.1); similar to Pyridine nucleotide-disulphide oxidoreductase (ISS) [Ostreococcus tauri] (GB:CAL51905.1); contains InterPro domain Dihydrolipoamide dehydrogenase; (InterPro:IPR006258); contains InterPro domain Pyridine nucleotide-disulphide oxidoreductase, class I; (InterPro:IPR001100); contains InterPro domain Pyridine nucleotide-disulphide oxidoreductase, NAD-binding region; (InterPro:IPR001327); contains InterPro domain Mercuric reductase; (InterPro:IPR000815); contains InterPro domain Pyridine nucleotide-disulphide oxidoreductase, class I, active site; (InterPro:IPR012999); contains InterPro domain Pyridine nucleotide-disulphide oxidoreductase dimerisation region; (InterPro:IPR004099); contains InterPro domain FAD-dependent pyridine nucleotide-disulphide oxidoreductase; (InterPro:IPR013027) |
| 699 | 246547\_at | AT5G14970 |  |  | -0.5 | -0.3 | -0.2 | 0.1 | 0.1 | -0.6 | -0.3 | -0.3 | -0.5 | -1 | -0.3 | -1.8 | -1.1 | -0.5 | -1.6 | -1 | -2.5 | -1.2 | -0.4 | -0.1 | -0.1 | 0.1 | -0.4 | -0.1 | -0.1 | -1.5 | 0 | -0.2 | similar to unknown protein [Arabidopsis thaliana] (TAIR:AT2G14910.1); similar to seed maturation-like protein, putative [Medicago truncatula] (GB:ABE86960.1) |
| 698 | 259896\_at | AT1G71500 |  | 1 | -0.6 | -0.3 | -0.1 | 0.1 | 0.1 | -0.4 | 0 | -0.2 | -0.1 | -1.9 | -0.4 | -1.9 | -1.5 | -1.6 | -2.8 | -1.7 | -1.6 | -1 | -0.5 | -0.4 | 0.1 | 0 | -0.3 | -0.3 | -0.1 | -0.7 | 0.1 | 0 | Rieske (2Fe-2S) domain-containing protein; similar to Rieske [2Fe-2S] domain, putative [Oryza sativa (ISS) [Ostreococcus tauri] (GB:CAL51406.1); similar to Os11g0242400 [Oryza sativa (japonica cultivar-group)] (GB:NP\_001067589.1); contains InterPro domain Rieske [2Fe-2S] region; (InterPro:IPR005806) |
| 697 | 254126\_at | AT4G24770 | ATRBP31, ATRBP33, CP31, RBP31 | 7 | -0.3 | 0 | 0.1 | 0.1 | 0.1 | -0.5 | -0.1 | 0 | -0.3 | -2.2 | -0.4 | -1.8 | -1.6 | -2.5 | -3.2 | -2.5 | -1.1 | -1.3 | -0.3 | -0.2 | 0.3 | 0.1 | -0.1 | 0 | 0 | -0.3 | 0.1 | 0 | encodes a chloroplast RNA-binding protein, encodes a chloroplast RNA-binding protein, encodes a chloroplast RNA-binding protein, encodes a chloroplast RNA-binding protein |
| 696 | 263678\_at | AT1G04420 |  | 1 | -0.6 | -0.2 | 0.1 | 0.1 | -0.1 | -0.1 | -0.1 | -0.1 | -0.9 | -2.3 | -0.6 | -2.2 | -2.7 | -2.2 | -3.2 | -2.2 | -1.3 | -1.9 | -0.3 | 0 | 0.3 | 0.1 | -0.2 | 0 | 0.1 | -0.9 | 0.1 | 0.1 | aldo/keto reductase family protein; similar to KAB1 (POTASSIUM CHANNEL BETA SUBUNIT), potassium channel [Arabidopsis thaliana] (TAIR:AT1G04690.1); similar to Aldo/keto reductase [Medicago truncatula] (GB:ABE91742.1); contains InterPro domain Aldo/keto reductase; (InterPro:IPR001395) |
| 695 | 259161\_at | AT3G01500 | ATBCA1, CA1, SABP3, ATBCA1, CA1, SABP3 | 22 | -0.4 | -0.2 | 0 | 0 | 0 | -0.2 | 0 | 0 | -0.3 | -3.1 | -0.2 | -1.6 | -0.8 | -5.3 | -3.4 | -3 | -1.4 | -0.6 | -0.1 | -0.1 | 0 | 0 | -0.2 | -0.3 | 0 | -0.1 | 0.1 | 0 | CA1 (CARBONIC ANHYDRASE 1); carbonate dehydratase/ zinc ion binding; Identical to Carbonic anhydrase, chloroplast precursor (EC 4.2.1.1) (Carbonate dehydratase) (CA1) [Arabidopsis Thaliana] (GB:P27140;GB:Q8RWW2;GB:Q93VR8); similar to CA2 (BETA CARBONIC ANHYDRASE 2), carbonate dehydratase/ zinc ion binding [Arabidopsis thaliana] (TAIR:AT5G14740.2); similar to chloroplast carbonic anhydrase precursor [Thlaspi caerulescens] (GB:AAS65454.1); contains InterPro domain Carbonic anhydrase, prokaryotic and plant; (InterPro:IPR001765), CA1 (CARBONIC ANHYDRASE 1); carbonate dehydratase/ zinc ion binding; Identical to Carbonic anhydrase, chloroplast precursor (EC 4.2.1.1) (Carbonate dehydratase) (CA1) [Arabidopsis Thaliana] (GB:P27140;GB:Q8RWW2;GB:Q93VR8); similar to CA2 (BETA CARBONIC ANHYDRASE 2), carbonate dehydratase/ zinc ion binding [Arabidopsis thaliana] (TAIR:AT5G14740.2); similar to chloroplast carbonic anhydrase precursor [Thlaspi caerulescens] (GB:AAS65454.1); contains InterPro domain Carbonic anhydrase, prokaryotic and plant; (InterPro:IPR001765), CA1 (CARBONIC ANHYDRASE 1); carbonate dehydratase/ zinc ion binding; Identical to Carbonic anhydrase, chloroplast precursor (EC 4.2.1.1) (Carbonate dehydratase) (CA1) [Arabidopsis Thaliana] (GB:P27140;GB:Q8RWW2;GB:Q93VR8); similar to CA2 (BETA CARBONIC ANHYDRASE 2), carbonate dehydratase/ zinc ion binding [Arabidopsis thaliana] (TAIR:AT5G14740.2); similar to chloroplast carbonic anhydrase precursor [Thlaspi caerulescens] (GB:AAS65454.1); contains InterPro domain Carbonic anhydrase, prokaryotic and plant; (InterPro:IPR001765) |
| 694 | 266716\_at | AT2G46820 | PSAP, PSI-P, PTAC8, TMP14 | 3 | -0.5 | 0 | -0.1 | 0.1 | 0 | -0.1 | 0 | -0.1 | -0.2 | -1.5 | -0.3 | -1.2 | -0.9 | -1.5 | -2.4 | -2.1 | -1.1 | -0.6 | -0.1 | 0 | 0 | 0 | -0.1 | -0.1 | 0 | -0.2 | -0.1 | -0.1 | Encodes the P subunit of Photosystem I. About 25% of the TMP14 pool appeared to be phosphorylated, and this ratio is not affected by light. Contains seven phosphorylation sites on threonine residue and chloroplast targeting signal. Located in the proximity of PSI-L, -H and -O subunits., Encodes the P subunit of Photosystem I. About 25% of the TMP14 pool appeared to be phosphorylated, and this ratio is not affected by light. Contains seven phosphorylation sites on threonine residue and chloroplast targeting signal. Located in the proximity of PSI-L, -H and -O subunits., Encodes the P subunit of Photosystem I. About 25% of the TMP14 pool appeared to be phosphorylated, and this ratio is not affected by light. Contains seven phosphorylation sites on threonine residue and chloroplast targeting signal. Located in the proximity of PSI-L, -H and -O subunits., Encodes the P subunit of Photosystem I. About 25% of the TMP14 pool appeared to be phosphorylated, and this ratio is not affected by light. Contains seven phosphorylation sites on threonine residue and chloroplast targeting signal. Located in the proximity of PSI-L, -H and -O subunits. |
| 693 | 253871\_at | AT4G27440 | PORB | 14 | -0.5 | 0.2 | 0 | 0.2 | -0.2 | -0.4 | 0.1 | -0.2 | -0.4 | -2.8 | -0.7 | -1.6 | -2.1 | -4.3 | -4.5 | -2.5 | -0.9 | -1.6 | -0.3 | 0.4 | 0.7 | -0.1 | -0.2 | -0.3 | -0.1 | -1 | -0.1 | -0.1 | light-dependent NADPH:protochlorophyllide oxidoreductase B |
| 692 | 256441\_at | AT3G10940 |  | 1 | -1.7 | -0.4 | -0.3 | 0.5 | 0.1 | 0.5 | 0 | -1 | -1.7 | -1.2 | -1.1 | -2.1 | -3.3 | -2.1 | -3.6 | -2.4 | -1.2 | -2.2 | -1.5 | -0.4 | -0.3 | -0.3 | -0.6 | -0.5 | -0.7 | -1 | 0.3 | -0.4 | protein phosphatase-related; similar to SEX4 (STARCH-EXCESS 4), protein tyrosine/serine/threonine phosphatase [Arabidopsis thaliana] (TAIR:AT3G52180.2); similar to Os12g0112500 [Oryza sativa (japonica cultivar-group)] (GB:NP\_001065974.1); similar to dual-specificity protein-like phosphatase 3 [Zea mays] (GB:ABA02564.1); similar to Os11g0113100 [Oryza sativa (japonica cultivar-group)] (GB:NP\_001065571.1); contains InterPro domain Dual specificity protein phosphatase; (InterPro:IPR000340); contains InterPro domain Tyrosine specific protein phosphatase and dual specificity protein phosphatase; (InterPro:IPR000387) |
| 691 | 253530\_at | AT4G31530 |  | 1 | -0.7 | -0.1 | -0.1 | 0 | 0.1 | 0.3 | 0.2 | -0.3 | -0.7 | -0.2 | -0.4 | -1.3 | -0.9 | -0.7 | -1.3 | -1.4 | -1.8 | -1.1 | -0.3 | -0.1 | -0.2 | 0.1 | -0.2 | -0.3 | -0.1 | -0.5 | 0 | 0.1 | catalytic/ coenzyme binding; similar to catalytic/ coenzyme binding [Arabidopsis thaliana] (TAIR:AT2G37660.1); similar to Male sterility-like [Trichodesmium erythraeum IMS101] (GB:YP\_724032.1); similar to Os03g0355900 [Oryza sativa (japonica cultivar-group)] (GB:NP\_001050138.1); contains InterPro domain NAD-dependent epimerase/dehydratase; (InterPro:IPR001509) |
| 690 | 259707\_at | AT1G77490 | TAPX | 6 | -0.5 | -0.2 | -0.1 | 0.2 | 0 | 0.1 | 0.1 | -0.4 | -1.3 | -2.4 | -0.6 | -4 | -3.1 | -2.7 | -3.6 | -3 | -3 | -1.4 | -0.5 | -0.1 | 0.2 | 0 | -0.3 | -0.1 | 0 | -0.8 | -0.1 | 0 | Encodes a chloroplastic thylakoid ascorbate peroxidase tAPX. Ascorbate peroxidases are enzymes that scavenge hydrogen peroxide in plant cells. Eight types of APX have been described for Arabidopsis: three cytosolic (APX1, APX2, APX6), two chloroplastic types (stromal sAPX, thylakoid tAPX), and three microsomal (APX3, APX4, APX5) isoforms. |
| 689 | 249875\_at | AT5G23120 | HCF136 | 10 | -0.6 | -0.1 | 0.1 | 0.1 | 0.1 | 0.1 | 0.2 | -0.3 | -0.8 | -1.1 | -0.3 | -2 | -1.7 | -1.6 | -1.9 | -2.1 | -1.1 | -1.5 | -0.1 | 0 | 0.2 | 0.1 | -0.1 | -0.3 | -0.2 | -0.4 | -0.1 | -0.1 | encodes a stability and/or assembly factor of photosystem II |
| 688 | 251243\_at | AT3G61870 |  | 3 | -1 | -0.3 | 0 | 0.1 | 0.1 | 0.2 | 0 | -0.2 | -0.8 | -2.2 | -0.5 | -2.1 | -1.7 | -2 | -3.7 | -2.6 | -2.4 | -1 | 0 | -0.2 | 0.4 | 0.1 | -0.2 | -0.1 | -0.2 | -0.8 | 0 | -0.2 | similar to conserved hypothetical protein [Medicago truncatula] (GB:ABE93513.1) |
| 687 | 254298\_at | AT4G22890 | PGR5-LIKE | 5 | -1.2 | -0.2 | -0.3 | 0.5 | -0.1 | -0.1 | 0 | -0.2 | -0.3 | -1.4 | -0.3 | -1 | -0.9 | -1.1 | -2.1 | -2.3 | -1.4 | -0.8 | -0.2 | 0 | -0.1 | -0.1 | -0.4 | -0.5 | 0 | -0.7 | 0 | -0.2 | similar to unknown protein [Arabidopsis thaliana] (TAIR:AT4G11960.1); similar to Os03g0857400 [Oryza sativa (japonica cultivar-group)] (GB:NP\_001051955.1); similar to Os08g0526300 [Oryza sativa (japonica cultivar-group)] (GB:NP\_001062297.1); similar to hypothetical protein OSJNBa0003M24.13 [Oryza sativa (japonica cultivar-group)] (GB:AAQ56398.1); contains domain no description (G3D.2.20.25.10); contains domain Zinc beta-ribbon (SSF57783) |
| 686 | 245701\_at | AT5G04140 | GLS1, GLU1, GLUS | 3 | -1.1 | -0.2 | -0.1 | -0.1 | 0 | -0.1 | 0.1 | -0.2 | -0.3 | -1.6 | -0.2 | -1.8 | -0.9 | -1.1 | -2.5 | -1.6 | -1.6 | -0.8 | -0.3 | -0.1 | 0 | 0.1 | -0.2 | -0.4 | -0.1 | -0.6 | 0.1 | -0.1 | Encodes a gene whose sequence is similar to ferredoxin dependent glutamate synthase (Fd-GOGAT). Expression in leaves is induced by light and sucrose. Proposed to be involved in photorespiration and nitrogen assimilation., Encodes a gene whose sequence is similar to ferredoxin dependent glutamate synthase (Fd-GOGAT). Expression in leaves is induced by light and sucrose. Proposed to be involved in photorespiration and nitrogen assimilation., Encodes a gene whose sequence is similar to ferredoxin dependent glutamate synthase (Fd-GOGAT). Expression in leaves is induced by light and sucrose. Proposed to be involved in photorespiration and nitrogen assimilation. |
| 685 | 253823\_at | AT4G28030 |  | 1 | -0.8 | 0 | -0.1 | 0.4 | 0.2 | 0.5 | 0.2 | 0.1 | -0.3 | -0.8 | -0.1 | -1.1 | -0.4 | -0.8 | -1.4 | -1.4 | -1.6 | -0.5 | -0.2 | -0.1 | 0.3 | 0.1 | -0.2 | -0.4 | 0.2 | -1.1 | 0.2 | 0.2 | GCN5-related N-acetyltransferase (GNAT) family protein; similar to GCN5-related N-acetyltransferase (GNAT) family protein [Arabidopsis thaliana] (TAIR:AT2G06025.1); similar to Os08g0260000 [Oryza sativa (japonica cultivar-group)] (GB:NP\_001061388.1); similar to Predicted N-acetyltransferase (ISS) [Ostreococcus tauri] (GB:CAL55572.1); contains InterPro domain GCN5-related N-acetyltransferase; (InterPro:IPR000182), N-acetyltransferase; similar to GCN5-related N-acetyltransferase (GNAT) family protein [Arabidopsis thaliana] (TAIR:AT2G06025.1); similar to Os08g0260000 [Oryza sativa (japonica cultivar-group)] (GB:NP\_001061388.1); similar to Predicted N-acetyltransferase (ISS) [Ostreococcus tauri] (GB:CAL55572.1); contains InterPro domain GCN5-related N-acetyltransferase; (InterPro:IPR000182) |
| 684 | 266224\_at | AT2G28800 | ABL3, ALB3 | 11 | -0.6 | -0.1 | -0.2 | 0.2 | 0 | -0.1 | 0.1 | -0.1 | -0.3 | -0.2 | -0.2 | -1.1 | -1.1 | -0.7 | -1.1 | -1.2 | -1.1 | -0.9 | -0.1 | -0.1 | 0 | -0.1 | -0.2 | -0.2 | 0 | -0.7 | 0 | 0 | member of Chloroplast membrane protein ALBINO3 family, member of Chloroplast membrane protein ALBINO3 family |
| 683 | 250668\_at | AT5G07020 |  | 2 | -0.7 | -0.4 | -0.3 | 0.2 | 0 | -0.1 | 0 | -0.2 | -0.4 | -0.8 | -0.3 | -1.9 | -1.7 | -2.4 | -2.5 | -2 | -1.3 | -1.2 | -0.3 | -0.3 | -0.2 | -0.3 | -0.2 | -0.4 | -0.2 | -0.7 | 0.1 | 0.1 | proline-rich family protein; similar to unknown protein [Lycopersicon esculentum] (GB:AAU03360.1) |
| 682 | 249691\_at | AT5G36170 | ATPRFB, HCF109 | 2 | -0.7 | -0.3 | -0.3 | 0.3 | 0.1 | 0 | -0.1 | -0.2 | -0.9 | -1.5 | -0.3 | -1.9 | -1.5 | -1 | -2.8 | -2 | -1.2 | -0.8 | -0.1 | -0.3 | 0 | -0.1 | -0.4 | -0.4 | -0.3 | -1.3 | 0 | -0.1 | Required for normal processing of polycistronic plastidial transcripts, Required for normal processing of polycistronic plastidial transcripts |
| 681 | 261788\_at | AT1G15980 | NDF1 | 1 | -1.4 | -0.2 | 0 | 0.1 | 0.2 | 0 | 0.1 | -0.2 | -0.6 | -2.1 | -0.6 | -2 | -1.5 | -2.5 | -4.6 | -3.5 | -2 | -1.7 | -0.4 | -0.2 | -0.1 | 0.1 | -0.2 | -0.5 | -0.3 | -1.1 | 0.1 | -0.1 | similar to Os08g0276100 [Oryza sativa (japonica cultivar-group)] (GB:NP\_001061432.1); similar to COG0859: ADP-heptose:LPS heptosyltransferase [Nostoc punctiforme PCC 73102] (GB:ZP\_00111141.1); contains domain UDP-Glycosyltransferase/glycogen phosphorylase (SSF53756) |
| 680 | 253197\_at | AT4G35250 |  | 1 | -1.2 | -0.3 | -0.1 | 0.1 | 0 | 0.1 | 0.2 | -0.4 | -0.6 | -2 | -0.7 | -2.2 | -1.9 | -2.4 | -2.6 | -3 | -1.9 | -1.3 | -0.6 | -0.5 | -0.3 | 0 | -0.3 | -0.4 | -0.5 | -0.7 | -0.1 | -0.2 | vestitone reductase-related; similar to DVR (PALE-GREEN AND CHLOROPHYLL B REDUCED 2), 3,8-divinyl protochlorophyllide a 8-vinyl reductase [Arabidopsis thaliana] (TAIR:AT5G18660.1); similar to hypothetical protein MtrDRAFT\_AC121239g27v1 [Medicago truncatula] (GB:ABE78447.1); contains InterPro domain NmrA-like; (InterPro:IPR008030) |
| 679 | 263048\_s\_at | AT2G05310, AT4G13500 AT2G05310, AT4G13500 |  | 1 | -0.6 | -0.1 | -0.1 | 0.3 | 0.2 | -0.2 | 0.1 | -0.2 | -0.7 | -1 | -0.6 | -1.8 | -1.6 | -2.1 | -2.4 | -2.4 | -1.4 | -0.7 | -0.2 | -0.2 | -0.4 | 0 | -0.5 | -0.6 | -0.4 | -1.3 | -0.1 | -0.2 | similar to unknown protein [Arabidopsis thaliana] (TAIR:AT4G13500.1); similar to Os03g0595300 [Oryza sativa (japonica cultivar-group)] (GB:NP\_001050596.1); similar to hypothetical protein MA4\_112I10.24 [Musa acuminata] (GB:ABF70151.1), similar to unknown protein [Arabidopsis thaliana] (TAIR:AT2G05310.1); similar to Os03g0595300 [Oryza sativa (japonica cultivar-group)] (GB:NP\_001050596.1); similar to hypothetical protein MA4\_112I10.24 [Musa acuminata] (GB:ABF70151.1) |
| 678 | 263142\_at | AT1G65230 |  | 1 | -1.3 | -0.3 | -0.1 | 0.3 | 0.1 | -0.1 | 0.2 | -0.5 | -0.9 | -3.4 | -0.6 | -2 | -1.8 | -2.3 | -4 | -3.9 | -3 | -1.6 | -0.8 | -0.6 | -0.3 | -0.2 | -0.5 | -0.6 | -0.6 | -1.4 | -0.2 | -0.5 | similar to hypothetical protein MtrDRAFT\_AC148171g2v1 [Medicago truncatula] (GB:ABE89789.1) |
| 677 | 262612\_at | AT1G14150 |  | 3 | -1.2 | -0.6 | -0.1 | 0.1 | 0 | -0.3 | 0.1 | -0.2 | -1 | -2.7 | -0.5 | -2.7 | -2.3 | -2.8 | -4.9 | -3.9 | -2.2 | -1.8 | -0.8 | -0.5 | -0.7 | -0.3 | -0.7 | -1.1 | -1 | -2.1 | -0.4 | -0.7 | oxygen evolving enhancer 3 (PsbQ) family protein; similar to PSBQ/PSBQ-2 (photosystem II subunit Q-2), calcium ion binding [Arabidopsis thaliana] (TAIR:AT4G05180.1); similar to Putative oxygen evolving enhancer protein 3, identical [Solanum demissum] (GB:AAW28572.1); contains InterPro domain Photosystem II oxygen evolving complex protein PsbQ; (InterPro:IPR008797), oxygen evolving enhancer 3 (PsbQ) family protein; similar to oxygen evolving enhancer 3 (PsbQ) family protein [Arabidopsis thaliana] (TAIR:AT3G01440.1); similar to Putative oxygen evolving enhancer protein 3, identical [Solanum demissum] (GB:AAW28572.1); contains InterPro domain Photosystem II oxygen evolving complex protein PsbQ; (InterPro:IPR008797) |
| 676 | 256015\_at | AT1G19150 | LHCA2\*1, LHCA6 | 2 | -1.5 | -0.2 | -0.2 | 0.2 | -0.1 | -0.2 | -0.1 | -0.2 | -1.3 | -4 | -0.7 | -2.4 | -2.7 | -8.2 | -8.7 | -6.6 | -2.2 | -2.4 | -0.6 | -0.3 | -0.3 | -0.2 | -0.5 | -1 | -1.8 | -1.6 | -0.2 | -1 | PSI type II chlorophyll a/b-binding protein (Lhca2\*1) mRNA,, PSI type II chlorophyll a/b-binding protein (Lhca2\*1) mRNA, |
| 675 | 252181\_at | AT3G50685 |  |  | -0.6 | -0.1 | -0.2 | 0.1 | 0 | 0 | 0.1 | -0.2 | -0.9 | -1.1 | -0.5 | -1.3 | -1.7 | -2 | -2.9 | -2.5 | -1 | -1 | -0.4 | -0.3 | -0.2 | -0.2 | -0.2 | -0.5 | -0.6 | -1.2 | 0 | -0.4 | similar to p-166-4\_1 [Pinus resinosa] (GB:AAY17046.1); similar to Os10g0213700 [Oryza sativa (japonica cultivar-group)] (GB:NP\_001064326.1) |
| 674 | 246154\_at | AT5G19940 |  | 2 | -0.6 | 0 | -0.2 | 0.2 | 0 | 0 | 0.2 | -0.2 | -0.5 | -1.6 | -0.4 | -1.4 | -1.1 | -1.6 | -2.3 | -2.1 | -1.8 | -1.6 | -0.2 | -0.1 | -0.1 | -0.1 | -0.1 | -0.2 | -0.3 | -1 | 0.1 | -0.2 | plastid-lipid associated protein PAP-related / fibrillin-related; similar to plastid-lipid associated protein PAP / fibrillin family protein [Arabidopsis thaliana] (TAIR:AT3G26070.1); similar to PAP fibrillin [Medicago truncatula] (GB:ABE82162.1); similar to OSIGBa0113I13.8 [Oryza sativa (indica cultivar-group)] (GB:CAH66782.1); similar to Os04g0607000 [Oryza sativa (japonica cultivar-group)] (GB:NP\_001053805.1); contains InterPro domain PAP fibrillin; (InterPro:IPR006843) |
| 673 | 264185\_at | AT1G54780 |  | 4 | -0.8 | 0 | 0 | 0.4 | 0 | 0.1 | 0 | -0.1 | -0.2 | -0.8 | -0.2 | -1 | -0.7 | -1.5 | -2 | -2.2 | -1.2 | -0.5 | -0.1 | -0.1 | -0.1 | 0 | -0.1 | -0.3 | -0.3 | -0.8 | 0.1 | 0 | thylakoid lumen 18.3 kDa protein; similar to Os05g0401100 [Oryza sativa (japonica cultivar-group)] (GB:NP\_001055486.1); similar to unnamed protein product [Ostreococcus tauri] (GB:CAL57312.1); contains InterPro domain Protein of unknown function DUF477; (InterPro:IPR007621) |
| 672 | 263880\_at | AT2G21960 |  | 1 | -0.7 | -0.3 | 0 | 0 | 0 | 0.1 | 0.1 | -0.1 | -0.8 | -0.4 | -0.3 | -1.7 | -1.7 | -1.8 | -2.4 | -2.4 | -1.2 | -0.7 | -0.6 | -0.4 | -0.6 | -0.1 | -0.3 | -0.2 | -0.1 | -1.1 | 0 | -0.1 | similar to unknown protein [Arabidopsis thaliana] (TAIR:AT1G56180.1); similar to Os04g0639300 [Oryza sativa (japonica cultivar-group)] (GB:NP\_001054033.1); similar to OSJNBb0003B01.9 [Oryza sativa (japonica cultivar-group)] (GB:CAE03618.3); similar to unnamed protein product [Ostreococcus tauri] (GB:CAL58559.1) |
| 671 | 245806\_at | AT1G45474 | LHCA5 | 7 | -1.1 | -0.3 | -0.1 | 0.2 | -0.1 | 0.2 | 0.1 | 0 | -0.7 | -0.4 | -0.3 | -2.1 | -1.8 | -1.6 | -2.2 | -2.2 | -1.2 | -1.4 | -0.3 | -0.2 | -0.3 | 0 | -0.3 | -0.3 | -0.6 | -1.2 | 0.1 | -0.3 | Encodes a component of the light harvesting complex of photosystem I. |
| 670 | 265415\_at | AT2G20890 | PSB29, THF1 | 5 | -0.4 | 0 | 0 | 0.1 | 0.1 | 0.1 | 0 | 0 | -0.3 | -1.3 | -0.4 | -1.5 | -1.2 | -1.2 | -2.4 | -1.8 | -1.4 | -0.8 | -0.2 | -0.1 | -0.1 | 0.1 | 0 | -0.1 | -0.2 | -0.6 | 0.1 | -0.1 | Chloroplast-localized Thylakoid formation1 gene product involved in vesicle-mediated formation of thylakoid membranes. Thf1 antisense lines contain abnormal chloroplasts early in leaf development (chloroplasts have loosely stacked thylakoid membranes). Expression was induced in the light and decreased under dark conditions. G-alpha interaction partner that functions downstream of the plasma membrane<96>delimited heterotrimeric G-protein (GPA1) in a D-glucose signaling pathway. Localized to both the outer plastid membrane and the stroma. Probably involved in the metabolic pathway that controls the assembly of the PS II complex., Chloroplast-localized Thylakoid formation1 gene product involved in vesicle-mediated formation of thylakoid membranes. Thf1 antisense lines contain abnormal chloroplasts early in leaf development (chloroplasts have loosely stacked thylakoid membranes). Expression was induced in the light and decreased under dark conditions. G-alpha interaction partner that functions downstream of the plasma membrane<96>delimited heterotrimeric G-protein (GPA1) in a D-glucose signaling pathway. Localized to both the outer plastid membrane and the stroma. Probably involved in the metabolic pathway that controls the assembly of the PS II complex. |
| 669 | 252116\_at | AT3G51510 |  |  | -0.7 | -0.1 | -0.2 | 0.1 | 0 | 0.1 | 0 | -0.4 | -0.8 | -1.3 | -0.5 | -1.7 | -1.7 | -2 | -2 | -2.7 | -1.7 | -0.6 | -0.8 | -0.5 | -0.5 | -0.2 | -0.2 | -0.4 | -0.5 | -1.1 | -0.1 | -0.4 | similar to unknown [Hordeum vulgare] (GB:ABK56720.1) |
| 668 | 264959\_at | AT1G77090 |  | 3 | -0.6 | -0.2 | -0.2 | 0.1 | 0 | 0.3 | -0.1 | -0.2 | -0.5 | -0.5 | -0.5 | -1.7 | -1.7 | -1.9 | -1.9 | -2.3 | -1.5 | -1.4 | -0.7 | -0.4 | -0.5 | 0 | -0.4 | -0.4 | -0.8 | -1.3 | 0.2 | -0.1 | thylakoid lumenal 29.8 kDa protein; Identical to Thylakoid lumenal 29.8 kDa protein, chloroplast precursor [Arabidopsis Thaliana] (GB:O49292); similar to thylakoid lumenal 20 kDa protein [Arabidopsis thaliana] (TAIR:AT3G56650.1); similar to Os12g0190200 [Oryza sativa (japonica cultivar-group)] (GB:NP\_001066340.1); similar to thylakoid lumenal 29.8 kDa protein, putative, expressed [Oryza sativa (japonica cultivar-group)] (GB:ABA96032.2); contains InterPro domain Photosystem II oxygen evolving complex protein PsbP; (InterPro:IPR002683) |
| 667 | 267430\_at | AT2G34860 | EDA3 | 2 | -0.7 | -0.3 | -0.1 | -0.1 | -0.1 | -0.2 | 0.1 | 0 | -0.3 | -1.7 | -0.2 | -1.8 | -1.3 | -1.5 | -2.3 | -1.8 | -1.3 | -1.8 | -0.2 | -0.3 | -0.3 | -0.1 | -0.3 | -0.2 | -0.2 | -0.5 | 0 | -0.1 | EDA3 (embryo sac development arrest 3); similar to conserved hypothetical protein [Medicago truncatula] (GB:ABE91339.1); contains domain Cysteine-rich domain of the chaperone protein DnaJ. (SSF57938); contains domain no description (G3D.2.10.230.10) |
| 666 | 261206\_at | AT1G12800 |  | 1 | -0.5 | -0.2 | -0.1 | -0.1 | -0.1 | -0.2 | 0 | 0 | -0.3 | -1.5 | -0.1 | -1.8 | -1.4 | -2 | -2.6 | -2 | -1.4 | -0.8 | -0.3 | -0.3 | -0.5 | -0.1 | -0.3 | 0 | -0.4 | -1.1 | 0 | 0 | S1 RNA-binding domain-containing protein; similar to S1 RNA-binding domain-containing protein [Arabidopsis thaliana] (TAIR:AT3G23700.1); similar to putative ribosomal S1 protein (with alternative splicing) [Oryza sativa (japonica cultivar-group)] (GB:AAS07358.1); similar to Os02g0115600 [Oryza sativa (japonica cultivar-group)] (GB:NP\_001045672.1); contains InterPro domain Nucleic acid-binding, OB-fold, subgroup; (InterPro:IPR012340); contains InterPro domain Nucleic acid-binding, OB-fold; (InterPro:IPR008994); contains InterPro domain RNA binding S1; (InterPro:IPR003029) |
| 665 | 256036\_at | AT1G07110 | ATF2KP, F2KP, FKFBP | 8 | -0.5 | -0.3 | -0.1 | -0.1 | -0.1 | -0.4 | 0.1 | -0.1 | -0.4 | -1.2 | -0.2 | -1.1 | -1.1 | -0.6 | -1.5 | -1.8 | -1.4 | -0.8 | -0.2 | -0.3 | -0.6 | -0.2 | -0.2 | -0.2 | -0.4 | -1.3 | 0.1 | -0.2 | Encodes the bifunctional enzyme fructose-6-phosphate 2-kinase/fructose-2,6-bisphosphatase., Encodes the bifunctional enzyme fructose-6-phosphate 2-kinase/fructose-2,6-bisphosphatase., Encodes the bifunctional enzyme fructose-6-phosphate 2-kinase/fructose-2,6-bisphosphatase. |
| 664 | 261695\_at | AT1G08520 | CHLD, PDE166 | 6 | -0.7 | 0 | 0.1 | -0.1 | 0.1 | -0.2 | 0.3 | 0.1 | -0.4 | -1.6 | -0.3 | -1.6 | -1.4 | -2 | -2.4 | -1.8 | -1.3 | -0.9 | -0.2 | -0.5 | -0.7 | 0.1 | -0.2 | 0 | -0.4 | -1.1 | 0.1 | 0 | CHLD/PDE166 (PIGMENT DEFECTIVE 166); ATP binding / magnesium chelatase/ nucleoside-triphosphatase/ nucleotide binding; Identical to Magnesium-chelatase subunit chlD, chloroplast precursor (EC 6.6.1.1) (Mg-protoporphyrin IX chelatase) (Mg-chelatase subunit D) (CHLD) [Arabidopsis Thaliana] (GB:Q9SJE1;GB:Q9SWY5); similar to CHLI1 (CHLORINA 42), magnesium chelatase [Arabidopsis thaliana] (TAIR:AT4G18480.1); similar to Magnesium-chelatase subunit chlD, chloroplast precursor (Mg-protoporphyrin IX chelatase) (Mg-chelatase subunit D) (GB:O22437); similar to Magnesium-chelatase subunit chlD, chloroplast precursor (Mg-protoporphyrin IX chelatase) (Mg-chelatase subunit D) (GB:O24133); similar to Magnesium-chelatase subunit chlD, chloroplast precursor (Mg-protoporphyrin IX chelatase) (Mg-chelatase subunit D) (GB:Q9SJE1); contains InterPro domain von Willebrand factor, type A; (InterPro:IPR002035); contains InterPro domain Magnesium chelatase ATPase subunit D; (InterPro:IPR011776); contains InterPro domain AAA ATPase; (InterPro:IPR003593); contains InterPro domain Magnesium chelatase, ChlI subunit; (InterPro:IPR000523); contains InterPro domain Magnesium chelatase, subunit ChlD; (InterPro:IPR012174), CHLD/PDE166 (PIGMENT DEFECTIVE 166); ATP binding / magnesium chelatase/ nucleoside-triphosphatase/ nucleotide binding; Identical to Magnesium-chelatase subunit chlD, chloroplast precursor (EC 6.6.1.1) (Mg-protoporphyrin IX chelatase) (Mg-chelatase subunit D) (CHLD) [Arabidopsis Thaliana] (GB:Q9SJE1;GB:Q9SWY5); similar to CHLI1 (CHLORINA 42), magnesium chelatase [Arabidopsis thaliana] (TAIR:AT4G18480.1); similar to Magnesium-chelatase subunit chlD, chloroplast precursor (Mg-protoporphyrin IX chelatase) (Mg-chelatase subunit D) (GB:O22437); similar to Magnesium-chelatase subunit chlD, chloroplast precursor (Mg-protoporphyrin IX chelatase) (Mg-chelatase subunit D) (GB:O24133); similar to Magnesium-chelatase subunit chlD, chloroplast precursor (Mg-protoporphyrin IX chelatase) (Mg-chelatase subunit D) (GB:Q9SJE1); contains InterPro domain von Willebrand factor, type A; (InterPro:IPR002035); contains InterPro domain Magnesium chelatase ATPase subunit D; (InterPro:IPR011776); contains InterPro domain AAA ATPase; (InterPro:IPR003593); contains InterPro domain Magnesium chelatase, ChlI subunit; (InterPro:IPR000523); contains InterPro domain Magnesium chelatase, subunit ChlD; (InterPro:IPR012174) |
| 663 | 252409\_at | AT3G47650 |  | 1 | -0.5 | -0.1 | 0.2 | -0.1 | 0.2 | -0.4 | 0 | 0.1 | -0.2 | -1.9 | -0.1 | -1.9 | -1.6 | -1.3 | -3.5 | -2.3 | -0.8 | -0.7 | 0 | -0.3 | -0.4 | 0 | -0.1 | 0.1 | -0.3 | -0.5 | -0.1 | -0.1 | bundle-sheath defective protein 2 family / bsd2 family; similar to putative chaperon P13.9 [Castanea sativa] (GB:AAK25754.1); contains domain Cysteine-rich domain of the chaperone protein DnaJ. (SSF57938); contains domain no description (G3D.2.10.230.10) |
| 662 | 248094\_at | AT5G55220 |  | 1 | -0.2 | 0 | 0.2 | -0.1 | 0 | -0.5 | -0.2 | -0.1 | -0.6 | -2.8 | -0.6 | -2.9 | -2.9 | -2 | -5.6 | -3.1 | -1.2 | -2.1 | -0.1 | -0.6 | -0.6 | 0.1 | -0.2 | 0 | -0.1 | -0.9 | 0 | 0.1 | trigger factor type chaperone family protein; similar to Os06g0308000 [Oryza sativa (japonica cultivar-group)] (GB:NP\_001057472.1); similar to unnamed protein product [Ostreococcus tauri] (GB:CAL52285.1); contains InterPro domain Bacterial trigger factor, C-terminal; (InterPro:IPR008880); contains InterPro domain Peptidylprolyl isomerase, FKBP-type; (InterPro:IPR001179); contains InterPro domain Bacterial trigger factor, N-terminal; (InterPro:IPR008881) |
| 661 | 262235\_at | AT1G48350 |  | 1 | -0.3 | -0.2 | 0 | 0.1 | -0.1 | -0.4 | 0.4 | 0 | -0.2 | -2.7 | -0.3 | -2.2 | -1.9 | -2.8 | -4.3 | -2.4 | -1 | -1.2 | 0.1 | -0.3 | -0.6 | 0 | -0.2 | -0.2 | 0 | -0.6 | -0.1 | 0 | ribosomal protein L18 family protein; Identical to 50S ribosomal protein L18, chloroplast precursor (CL18) (RPL18) [Arabidopsis Thaliana] (GB:Q9SX68); similar to ribosomal protein L18 family protein [Arabidopsis thaliana] (TAIR:AT1G14205.1); similar to Os03g0828100 [Oryza sativa (japonica cultivar-group)] (GB:NP\_001051775.1); similar to 50S ribosomal protein L18, chloroplast precursor, putative, expressed [Oryza sativa (japonica cultivar-group)] (GB:ABF99674.1); contains InterPro domain Ribosomal protein L18; (InterPro:IPR004389); contains InterPro domain Ribosomal protein L18P/L5E; (InterPro:IPR005484) |
| 660 | 250058\_at | AT5G17870 | PSRP6 |  | -0.3 | -0.1 | -0.1 | 0.1 | 0 | -0.4 | 0.1 | 0 | -0.4 | -2.2 | -0.3 | -1.8 | -1.3 | -2.1 | -3.6 | -2.2 | -0.9 | -1.1 | 0 | -0.2 | -0.4 | 0.1 | -0.2 | -0.2 | 0 | -0.4 | 0 | -0.1 | plastid-specific ribosomal protein 6 precursor (Psrp-6) - like |
| 659 | 257190\_at | AT3G13120 |  | 1 | -0.3 | -0.1 | 0 | 0.1 | 0 | -0.6 | 0.2 | 0.1 | -0.3 | -2.6 | -0.3 | -2.1 | -1.7 | -2.5 | -3.5 | -2.2 | -1.3 | -1.3 | 0.2 | 0 | -0.4 | -0.1 | -0.1 | -0.2 | 0.1 | -0.8 | 0.2 | 0.1 | 30S ribosomal protein S10, chloroplast, putative; Identical to 30S ribosomal protein S10, chloroplast precursor (RPS10) [Arabidopsis Thaliana] (GB:Q9LK61); similar to 30S ribosomal protein S10-like [Brassica napus] (GB:AAW22623.1); contains InterPro domain Ribosomal protein S10; (InterPro:IPR001848); contains InterPro domain Ribosomal protein S10, bacterial form; (InterPro:IPR005731) |
| 658 | 252853\_at | AT4G39710 |  | 3 | -1.4 | -0.5 | 0.1 | 0.1 | -0.2 | -0.5 | 0.1 | -0.2 | -0.9 | -5.1 | -0.4 | -3.6 | -2.6 | -2.5 | -6.8 | -4.6 | -2.7 | -1.9 | -0.5 | -0.4 | -0.9 | -0.2 | -0.6 | -0.7 | -0.6 | -1.5 | -0.3 | -0.4 | immunophilin, putative / FKBP-type peptidyl-prolyl cis-trans isomerase, putative; Identical to Probable FKBP-type peptidyl-prolyl cis-trans isomerase 4, chloroplast precursor (EC 5.2.1.8) (PPIase) (Rotamase) (FKBP23I) [Arabidopsis Thaliana] (GB:Q9SCY3;GB:O65658;GB:Q941D9); similar to FK506-binding protein 1 (FKBP13) [Arabidopsis thaliana] (TAIR:AT5G45680.1); similar to Os06g0663800 [Oryza sativa (japonica cultivar-group)] (GB:NP\_001058294.1); similar to Os02g0751600 [Oryza sativa (japonica cultivar-group)] (GB:NP\_001048136.1); similar to Chain A, Crystal Structure Of Atfkbp13 (GB:1U79); contains InterPro domain Peptidylprolyl isomerase, FKBP-type; (InterPro:IPR001179) |
| 657 | 247816\_at | AT5G58260 |  | 2 | -0.9 | -0.6 | -0.1 | -0.1 | -0.2 | -0.5 | -0.1 | -0.3 | -1 | -3.5 | -0.4 | -2.8 | -2.4 | -2.2 | -6.9 | -5.2 | -2.4 | -1.7 | -0.8 | -0.3 | -0.8 | -0.1 | -0.8 | -0.9 | -1 | -1.7 | -0.3 | -0.6 | Encodes subunit NDH-N of NAD(P)H:plastoquinone dehydrogenase complex (Ndh complex) present in the thylakoid membrane of chloroplasts. This subunit is thought to be required for Ndh complex assembly. |
| 656 | 266979\_at | AT2G39470 | PPL2, PPL2 | 5 | -1.1 | -0.2 | -0.2 | -0.1 | 0 | -0.2 | 0 | -0.1 | -0.8 | -3.5 | -0.5 | -2.6 | -2.4 | -4 | -6.3 | -3.9 | -2.3 | -1.6 | -0.4 | -0.2 | -0.5 | 0 | -0.5 | -0.4 | -0.5 | -1.3 | -0.3 | -0.2 | calcium ion binding; similar to photosystem II reaction center PsbP family protein [Arabidopsis thaliana] (TAIR:AT3G55330.1); similar to kinase binding protein [Oryza sativa (japonica cultivar-group)] (GB:BAF30961.1); contains InterPro domain Photosystem II oxygen evolving complex protein PsbP; (InterPro:IPR002683), photosystem II reaction center PsbP family protein; similar to photosystem II reaction center PsbP family protein [Arabidopsis thaliana] (TAIR:AT3G55330.1); similar to kinase binding protein [Oryza sativa (japonica cultivar-group)] (GB:BAF30961.1); contains InterPro domain Photosystem II oxygen evolving complex protein PsbP; (InterPro:IPR002683) |
| 655 | 260704\_at | AT1G32470 |  | 1 | -0.6 | -0.2 | 0.1 | 0 | -0.1 | -0.2 | 0 | -0.1 | -0.9 | -2.7 | -0.4 | -2.7 | -2.4 | -2.3 | -4.3 | -3.2 | -1.1 | -1.1 | -0.2 | -0.1 | -0.2 | 0 | -0.1 | -0.1 | -0.1 | -0.7 | 0 | -0.1 | glycine cleavage system H protein, mitochondrial, putative; Identical to Probable glycine cleavage system H protein 2, mitochondrial precursor [Arabidopsis Thaliana] (GB:Q9LQL0); similar to GDCH (Glycine decarboxylase complex H) [Arabidopsis thaliana] (TAIR:AT2G35370.1); similar to Glycine cleavage system H protein, mitochondrial precursor (GB:P16048); contains InterPro domain 2-oxo acid dehydrogenase, lipoyl-binding site; (InterPro:IPR003016); contains InterPro domain Glycine cleavage H-protein; (InterPro:IPR002930); contains InterPro domain Single hybrid motif; (InterPro:IPR011053) |
| 654 | 255719\_at | AT1G32080 |  | 2 | -1.2 | -0.4 | 0.1 | 0.1 | -0.1 | -0.3 | 0.1 | -0.3 | -0.6 | -2.4 | -0.6 | -3.3 | -2.2 | -2.5 | -5.4 | -3.2 | -1.6 | -2.6 | -0.5 | -0.2 | -0.4 | -0.1 | -0.3 | -0.4 | -0.3 | -0.9 | 0 | -0.2 | membrane protein, putative; similar to Os10g0578800 [Oryza sativa (japonica cultivar-group)] (GB:NP\_001065502.1); similar to LrgB-like family protein, expressed [Oryza sativa (japonica cultivar-group)] (GB:ABB48034.1); contains InterPro domain Duffy antigen/chemokine receptor; (InterPro:IPR005384); contains InterPro domain LrgB-like protein; (InterPro:IPR007300) |
| 653 | 264394\_at | AT1G11860 |  | 1 | -0.5 | 0 | 0.1 | 0 | 0 | -0.3 | -0.1 | -0.1 | -0.6 | -2.4 | -0.6 | -2.5 | -1.8 | -2.3 | -4.5 | -2.9 | -1.8 | -1.4 | -0.2 | 0 | 0 | 0 | -0.3 | -0.3 | -0.1 | -1.1 | -0.1 | -0.1 | aminomethyltransferase, putative; Identical to Aminomethyltransferase, mitochondrial precursor (EC 2.1.2.10) (Glycine cleavage system T protein) (GCVT) (GDCST) [Arabidopsis Thaliana] (GB:O65396;GB:Q42123); similar to unknown protein [Arabidopsis thaliana] (TAIR:AT1G60990.2); similar to Aminomethyltransferase, mitochondrial precursor (Glycine cleavage system T protein) (GCVT) (GB:P93256); similar to Aminomethyltransferase, mitochondrial precursor (Glycine cleavage system T protein) (GCVT) (GB:O49849); similar to Aminomethyltransferase, mitochondrial precursor (Glycine cleavage system T protein) (GCVT) (GB:P49363); contains InterPro domain Glycine cleavage T protein (aminomethyl transferase); (InterPro:IPR006222); contains InterPro domain Glycine cleavage system T protein; (InterPro:IPR006223) |
| 652 | 251784\_at | AT3G55330 | PPL1 | 4 | -0.6 | -0.1 | 0 | -0.1 | 0 | -0.6 | -0.2 | 0 | -0.5 | -2.8 | -0.3 | -3.1 | -2.2 | -2.6 | -5.4 | -2.9 | -1.5 | -1.5 | -0.2 | -0.2 | -0.1 | 0 | -0.3 | -0.3 | -0.4 | -0.8 | -0.1 | -0.3 | photosystem II reaction center PsbP family protein; Identical to Thylakoid lumenal 25.6 kDa protein, chloroplast precursor [Arabidopsis Thaliana] (GB:P82538); similar to photosystem II reaction center PsbP family protein [Arabidopsis thaliana] (TAIR:AT2G39470.1); similar to PsbP family protein, expressed [Oryza sativa (japonica cultivar-group)] (GB:ABG66119.1); similar to Os10g0461100 [Oryza sativa (japonica cultivar-group)] (GB:NP\_001064776.1); similar to Os08g0347500 [Oryza sativa (japonica cultivar-group)] (GB:NP\_001061609.1); contains InterPro domain Photosystem II oxygen evolving complex protein PsbP; (InterPro:IPR002683) |
| 651 | 255720\_at | AT1G32060 | PRK | 7 | -0.7 | 0 | 0.1 | 0 | -0.1 | -0.1 | 0 | 0.1 | -0.2 | -1.1 | -0.2 | -1.4 | -0.7 | -0.9 | -3.5 | -2.5 | -1.6 | -0.8 | 0 | 0 | -0.2 | 0 | -0.2 | -0.5 | -0.3 | -1.1 | 0 | -0.2 | PRK (PHOSPHORIBULOKINASE); ATP binding / phosphoribulokinase/ protein binding; Identical to Phosphoribulokinase, chloroplast precursor (EC 2.7.1.19) (Phosphopentokinase) (PRKase) (PRK) [Arabidopsis Thaliana] (GB:P25697); similar to uracil phosphoribosyltransferase, putative / UMP pyrophosphorylase, putative / UPRTase, putative [Arabidopsis thaliana] (TAIR:AT3G27440.1); similar to Phosphoribulokinase, chloroplast precursor (Phosphopentokinase) (PRKase) (PRK) (GB:P26302); similar to phosphoribulokinase; ribulose-5-phosphate kinase [Triticum aestivum] (GB:CAA41020.1); similar to phosphoribulokinase (EC 2.7.1.19) - wheat (GB:S16585); contains InterPro domain Phosphoribulokinase; (InterPro:IPR006082); contains InterPro domain Phosphoribulokinase/uridine kinase; (InterPro:IPR006083) |
| 650 | 251762\_at | AT3G55800 | SBPASE | 4 | -0.8 | 0 | 0 | 0 | -0.1 | -0.1 | 0 | 0.1 | -0.4 | -1.3 | -0.2 | -2.1 | -1.4 | -1.1 | -4 | -3.1 | -1.3 | -1 | -0.2 | 0 | -0.1 | 0 | -0.1 | -0.3 | -0.2 | -1 | 0.1 | -0.1 | Encodes the chloroplast enzyme sedoheptulose-1,7-bisphosphatase (SBPase), involved in the carbon reduction of the Calvin cycle. Increase in SBPase activity in transgenic lines accumulate up to 50% more sucrose and starch than wild-type. |
| 649 | 262645\_at | AT1G62750 | ATSCO1, ATSCO1/CPEF-G, SCO1 | 3 | -0.8 | 0 | 0.2 | 0 | -0.3 | -0.8 | 0.1 | 0 | -0.2 | -2 | -0.3 | -1.9 | -1.2 | -0.7 | -3 | -1.8 | -1.3 | -1.2 | -0.1 | 0 | -0.3 | 0.1 | -0.2 | -0.2 | -0.5 | -1.1 | -0.1 | -0.2 | Nuclear encoded protein consists of the five domains conserved in EF-G proteins, with two GTP-binding sites in the first domain, and an additional transit peptide at the N-terminus. Localized in chloroplasts. Point mutation results in a delay in the onset of germination. At early developmental stage embryos still contain undifferentiated proplastids. The greening of cotyledons is severely impaired in light-grown mutant sco1 seedlings, whereas the following true leaves develop normally as in wild-type plants., Nuclear encoded protein consists of the five domains conserved in EF-G proteins, with two GTP-binding sites in the first domain, and an additional transit peptide at the N-terminus. Localized in chloroplasts. Point mutation results in a delay in the onset of germination. At early developmental stage embryos still contain undifferentiated proplastids. The greening of cotyledons is severely impaired in light-grown mutant sco1 seedlings, whereas the following true leaves develop normally as in wild-type plants., Nuclear encoded protein consists of the five domains conserved in EF-G proteins, with two GTP-binding sites in the first domain, and an additional transit peptide at the N-terminus. Localized in chloroplasts. Point mutation results in a delay in the onset of germination. At early developmental stage embryos still contain undifferentiated proplastids. The greening of cotyledons is severely impaired in light-grown mutant sco1 seedlings, whereas the following true leaves develop normally as in wild-type plants. |
| 648 | 256115\_at | AT1G16880 |  | 3 | -0.5 | -0.1 | 0.2 | 0.2 | -0.2 | -0.5 | 0.1 | 0 | -0.3 | -1.9 | -0.1 | -1.4 | -1 | -0.7 | -2.6 | -1.5 | -1.1 | -0.8 | -0.2 | -0.1 | -0.3 | -0.1 | -0.2 | -0.3 | -0.2 | -1 | -0.2 | -0.2 | uridylyltransferase-related; similar to ACT domain-containing protein [Arabidopsis thaliana] (TAIR:AT5G04740.1); similar to unnamed protein product [Ostreococcus tauri] (GB:CAL58487.1); similar to Os08g0242700 [Oryza sativa (japonica cultivar-group)] (GB:NP\_001061342.1); similar to Os12g0152700 [Oryza sativa (japonica cultivar-group)] (GB:NP\_001066177.1); contains domain Regulatory domain in the aminoacid metabolism (SSF55021) |
| 647 | 253858\_at | AT4G27600 |  |  | -0.3 | -0.3 | -0.1 | -0.1 | -0.1 | -0.7 | -0.1 | -0.1 | -0.5 | -1.8 | -0.3 | -1.3 | -0.9 | -1 | -1.9 | -1.3 | -1.2 | -1.3 | -0.3 | -0.6 | -0.7 | -0.1 | -0.4 | -0.5 | -0.6 | -1.1 | -0.1 | -0.1 | pfkB-type carbohydrate kinase family protein; similar to pfkB-type carbohydrate kinase family protein [Arabidopsis thaliana] (TAIR:AT1G19600.1); similar to carbohydrate kinase, putative, expressed [Oryza sativa (japonica cultivar-group)] (GB:ABG66284.1); similar to Os10g0573000 [Oryza sativa (japonica cultivar-group)] (GB:NP\_001065468.1); contains InterPro domain PfkB; (InterPro:IPR011611); contains InterPro domain Carbohydrate kinase, PfkB; (InterPro:IPR002173); contains InterPro domain Ribokinase; (InterPro:IPR002139) |
| 646 | 252481\_at | AT3G46630 |  | 1 | 0.2 | 0 | -0.2 | 0.1 | 0 | -0.6 | 0.2 | -0.1 | -0.5 | -2 | -0.2 | -2 | -0.9 | -0.7 | -2.4 | -1 | -1.6 | -1.5 | -0.3 | -0.3 | -0.9 | 0 | -0.3 | -0.3 | -0.4 | -1.1 | 0.2 | -0.1 | similar to defective chloroplasts and leaves protein-related / DCL protein-related [Arabidopsis thaliana] (TAIR:AT1G45230.1); similar to putative defective chloroplasts and leaves (DCL) protein [Oryza sativa (japonica cultivar-group)] (GB:AAQ56405.1) |
| 645 | 266857\_at | AT2G26900 |  |  | -0.3 | -0.1 | 0.1 | 0.2 | 0 | -0.2 | 0 | -0.1 | -0.4 | -1.3 | -0.1 | -1.1 | -1 | -0.9 | -2.8 | -1.5 | -1.2 | -0.6 | -0.3 | -0.4 | -0.6 | -0.3 | -0.3 | -0.1 | -0.2 | -1 | 0 | -0.2 | bile acid:sodium symporter family protein; similar to bile acid:sodium symporter family protein [Arabidopsis thaliana] (TAIR:AT1G78560.1); similar to putative anion:sodium symporter [Lycopersicon esculentum] (GB:AAU03362.1); contains InterPro domain Bile acid:sodium symporter; (InterPro:IPR002657); contains InterPro domain Bile acid transporter; (InterPro:IPR004710) |
| 644 | 249685\_at |  |  |  | 0.6 | -0.1 | 0 | 0.2 | 0 | -1.4 | -0.1 | -0.1 | -0.3 | -2.3 | -0.3 | -1.9 | -1.6 | -2 | -3.3 | -1.8 | -1.4 | -1 | -0.3 | -0.2 | -0.4 | -0.1 | -0.5 | -0.4 | -0.9 | -1.4 | 0 | -0.2 |  |
| 643 | 245347\_at | AT4G14890 |  | 1 | 0.1 | -0.3 | -0.1 | 0.2 | 0.1 | -1.9 | 0.1 | -0.2 | -0.4 | -2.6 | -0.3 | -2 | -1.1 | -1.9 | -4.1 | -2.4 | -1.9 | -0.5 | -0.4 | -0.3 | -0.2 | -0.1 | -0.4 | -0.4 | -1 | -1.5 | -0.3 | -0.3 | ferredoxin family protein; similar to ATFD3 (FERREDOXIN 3), electron carrier [Arabidopsis thaliana] (TAIR:AT2G27510.1); similar to 2Fe-2S ferredoxin, iron-sulfur binding site [Medicago truncatula] (GB:ABE82976.1); contains InterPro domain 2Fe-2S Ferredoxin-like fold; (InterPro:IPR012675); contains InterPro domain Ferredoxin [2Fe-2S], plant; (InterPro:IPR010241); contains InterPro domain Ferredoxin; (InterPro:IPR001041) |
| 642 | 258956\_at | AT3G01440 |  | 3 | -0.3 | -0.7 | 0.2 | -0.1 | 0 | -1.5 | -0.2 | -0.2 | -1.2 | -5.7 | -0.4 | -3.7 | -2.4 | -1.2 | -7.3 | -3.8 | -2.3 | -1.9 | -0.7 | -0.4 | -0.9 | -0.1 | -0.9 | -1.3 | -1.2 | -2.9 | -0.6 | -1 | oxygen evolving enhancer 3 (PsbQ) family protein; similar to PSBQ/PSBQ-1/PSBQA, calcium ion binding [Arabidopsis thaliana] (TAIR:AT4G21280.2); similar to Os07g0105600 [Oryza sativa (japonica cultivar-group)] (GB:NP\_001058698.1); similar to photosystem II oxygen-evolving complex protein 3 [Lycopersicon esculentum] (GB:AAU03361.1); contains InterPro domain Photosystem II oxygen evolving complex protein PsbQ; (InterPro:IPR008797) |
| 641 | 245388\_at | AT4G16410 |  |  | -0.3 | -0.2 | 0 | 0.2 | 0.2 | -1.2 | 0 | 0 | -0.5 | -2 | -0.3 | -1.6 | -1.3 | -1.2 | -2.6 | -2.4 | -1.1 | -0.7 | -0.2 | -0.2 | -0.4 | -0.2 | -0.4 | -0.4 | -0.8 | -1.6 | -0.2 | -0.3 | similar to Os07g0607200 [Oryza sativa (japonica cultivar-group)] (GB:NP\_001060233.1); contains InterPro domain Protein of unknown function DUF751; (InterPro:IPR008470) |
| 640 | 258495\_at | AT3G02690 |  | 1 | -0.6 | -0.2 | -0.1 | 0.3 | 0.2 | -0.5 | 0 | 0.1 | -0.6 | -1.4 | -0.3 | -1.4 | -1.1 | -1.2 | -1.4 | -2.6 | -2.3 | -0.7 | -0.3 | -0.2 | -0.8 | 0 | -0.6 | -0.6 | -1.1 | -1.7 | -0.2 | -0.5 | integral membrane family protein; similar to unknown protein [Oryza sativa (japonica cultivar-group)] (GB:AAM92827.1); contains InterPro domain ssDNA-binding transcriptional regulator; (InterPro:IPR009044); contains InterPro domain Protein of unknown function DUF6, transmembrane; (InterPro:IPR000620) |
| 639 | 253860\_at | AT4G27700 |  | 1 | -0.6 | -0.2 | -0.4 | 0.2 | 0 | -0.6 | 0.1 | -0.2 | -0.4 | -1.2 | -0.3 | -1.9 | -1.5 | -2.6 | -2.9 | -2.9 | -2.1 | -0.9 | -0.6 | -0.3 | -0.5 | -0.3 | -0.6 | -0.5 | -1.3 | -1.6 | -0.2 | -0.4 | rhodanese-like domain-containing protein; similar to rhodanese-like domain-containing protein [Arabidopsis thaliana] (TAIR:AT3G08920.1); similar to unnamed protein product [Ostreococcus tauri] (GB:CAL52773.1); similar to Os09g0530000 [Oryza sativa (japonica cultivar-group)] (GB:NP\_001063746.1); contains InterPro domain Rhodanese-like; (InterPro:IPR001763) |
| 638 | 247936\_at | AT5G57030 | LUT2 | 13 | -0.3 | -0.2 | -0.3 | 0.2 | -0.1 | 1 | 0.1 | -0.2 | -0.5 | -1.9 | -0.4 | -2.4 | -1.7 | -1.8 | -2.8 | -2 | -1.1 | -0.9 | -0.7 | -0.3 | -0.6 | -0.3 | -0.5 | -0.8 | -1.2 | -1.5 | 0 | -0.4 | Lutein-deficient 2 (LUT2) required for lutein biosynthesis, member of the xanthophyll class of carotenoids. Encodes lycopene epsilon cyclase |
| 637 | 260412\_at | AT1G69830 | AMY3, ATAMY3 | 7 | -0.4 | -0.1 | -0.3 | 0.1 | 0 | -0.1 | -0.1 | -0.4 | -0.7 | -2 | -0.4 | -1.4 | -0.9 | -1 | -2.2 | -0.9 | -1.3 | -1.4 | -0.8 | -0.6 | -0.6 | -0.1 | -0.2 | -0.2 | -0.1 | -0.7 | 0 | 0 | Encodes a plastid-localized α-amylase. Expression is reduced in the SEX4 mutant. Loss of function mutations show normal diurnal pattern of starch accumulation/degradation. Expression follows circadian rhythms., Encodes a plastid-localized α-amylase. Expression is reduced in the SEX4 mutant. Loss of function mutations show normal diurnal pattern of starch accumulation/degradation. Expression follows circadian rhythms. |
| 636 | 248748\_at | AT5G47840 | AMK2 | 2 | -0.6 | 0 | -0.1 | 0.1 | 0 | -0.1 | 0 | -0.1 | -0.2 | -1.6 | -0.3 | -1.2 | -1.1 | -2.1 | -1.4 | -1 | -1.2 | -0.6 | -0.3 | -0.3 | -0.2 | 0 | -0.2 | -0.1 | -0.1 | -0.7 | 0.1 | -0.1 | adenylate kinase, chloroplast, putative / ATP-AMP transphosphorylase, putative; Identical to Probable adenylate kinase 2, chloroplast precursor (EC 2.7.4.3) (ATP- AMP transphosphorylase) [Arabidopsis Thaliana] (GB:Q9FIJ7); similar to nucleotide kinase [Arabidopsis thaliana] (TAIR:AT5G35170.2); similar to Os08g0109300 [Oryza sativa (japonica cultivar-group)] (GB:NP\_001060810.1); similar to Adenylate kinase, chloroplast (ATP-AMP transphosphorylase) (GB:P43188); contains InterPro domain Adenylate kinase, subfamily; (InterPro:IPR006259); contains InterPro domain Adenylate/cytidine kinase, N-terminal; (InterPro:IPR011769); contains InterPro domain Adenylate kinase; (InterPro:IPR000850) |
| 635 | 267220\_at | AT2G02500 | ATMEPCT, ISPD, MCT | 6 | -0.3 | -0.2 | -0.1 | 0.3 | 0.2 | -0.1 | 0 | -0.1 | -0.7 | -3 | -1.1 | -3 | -2.5 | -2.4 | -4.1 | -2.7 | -1.1 | -2.6 | -0.1 | -0.2 | -0.5 | 0.1 | -0.2 | -0.2 | 0 | -0.8 | 0.4 | 0 | Encodes a protein with 4-Diphosphocytidyl-2C-methyl-D-erythritol synthase activity. The enzyme has an absolute requirement for divalent cations (Mg2+ reaches the highest catalytic activity)., Encodes a protein with 4-Diphosphocytidyl-2C-methyl-D-erythritol synthase activity. The enzyme has an absolute requirement for divalent cations (Mg2+ reaches the highest catalytic activity)., Encodes a protein with 4-Diphosphocytidyl-2C-methyl-D-erythritol synthase activity. The enzyme has an absolute requirement for divalent cations (Mg2+ reaches the highest catalytic activity). |
| 634 | 256753\_at | AT3G27160 | GHS1 | 1 | -0.2 | 0.1 | 0.1 | 0.3 | 0.1 | -0.1 | -0.1 | -0.1 | -0.6 | -2.7 | -0.4 | -2.3 | -1.9 | -3.4 | -3.3 | -2.3 | -0.9 | -1 | 0 | -0.2 | -0.4 | 0.1 | 0 | -0.1 | 0.1 | -0.4 | 0.2 | 0.1 | GHS1 encodes plastid ribosomal protein S21 |
| 633 | 259633\_at | AT1G56500 |  | 1 | -0.3 | 0 | 0.4 | 0.3 | 0 | 0.2 | 0 | -0.1 | -0.9 | -1.8 | -0.3 | -1.8 | -1.4 | -0.8 | -2.7 | -1.4 | -2.2 | -1.1 | -0.4 | -0.3 | -0.6 | 0.3 | 0 | 0 | 0.2 | -1.2 | 0.4 | 0.1 | haloacid dehalogenase-like hydrolase family protein; similar to haloacid dehalogenase-like hydrolase family protein [Arabidopsis thaliana] (TAIR:AT4G39970.1); similar to HAD-superfamily hydrolase, subfamily IA, variant 3 containing protein, expressed [Oryza sativa (japonica cultivar-group)] (GB:ABF95582.1); similar to HAD-superfamily hydrolase, subfamily IA, variant 3 containing protein, expressed [Oryza sativa (japonica cultivar-group)] (GB:ABF95581.1); similar to Os03g0311300 [Oryza sativa (japonica cultivar-group)] (GB:NP\_001049921.1); contains InterPro domain HAD-superfamily hydrolase subfamily IA, variant 3; (InterPro:IPR006402); contains InterPro domain Low-density lipoprotein receptor, YWTD repeat; (InterPro:IPR000033); contains InterPro domain Thioredoxin-like fold; (InterPro:IPR012336); contains InterPro domain Redoxin; (InterPro:IPR013740); contains InterPro domain Thioredoxin domain 2; (InterPro:IPR006663); contains InterPro domain Quinoprotein amine dehydrogenase, beta chain-like; (InterPro:IPR011044); contains InterPro domain Thioredoxin fold; (InterPro:IPR012335); contains InterPro domain Thioredoxin-like; (InterPro:IPR011594); contains InterPro domain Haloacid dehalogenase-like hydrolase; (InterPro:IPR005834); contains InterPro domain Haloacid dehalogenase/epoxide hydrolase; (InterPro:IPR005833); contains InterPro domain NHL repeat; (InterPro:IPR001258) |
| 632 | 249899\_at | AT5G22620 |  |  | -0.6 | -0.3 | -0.3 | 0.1 | -0.1 | -0.1 | 0.2 | -0.3 | -0.7 | -2 | -0.3 | -2.3 | -1.7 | -1.6 | -2.6 | -1.7 | -1.1 | -0.8 | -0.5 | -0.5 | -0.6 | 0 | -0.3 | -0.1 | 0.2 | -0.7 | 0.2 | 0.1 | phosphoglycerate/bisphosphoglycerate mutase family protein; similar to phosphoglycerate/bisphosphoglycerate mutase family protein [Arabidopsis thaliana] (TAIR:AT3G50520.1); similar to phosphoglycerate mutase family, putative [Oryza sativa (japonica cultivar-group)] (GB:AAX92756.1); similar to Os11g0150100 [Oryza sativa (japonica cultivar-group)] (GB:NP\_001065757.1); contains InterPro domain Phosphoglycerate/bisphosphoglycerate mutase; (InterPro:IPR001345); contains InterPro domain Phosphoglycerate mutase; (InterPro:IPR013078) |
| 631 | 253009\_at | AT4G37930 | SHM1, SHMT1, STM | 12 | -0.5 | 0 | 0 | 0.2 | -0.2 | 0 | -0.1 | -0.1 | -0.2 | -1.1 | -0.2 | -1 | -0.6 | -0.6 | -1.6 | -1.4 | -0.7 | -0.3 | -0.2 | -0.1 | -0.2 | 0.1 | -0.1 | -0.2 | 0 | -0.2 | 0 | 0 | Encodes a protein with mitochondrial serine hydroxymethyltransferase activity, which functions in the photorespiratory pathway, catalyzes the conversion of serine and tetrahydrofolate to glycine and 5,10-methylene tetrahydrofolate. Involved in controlling cell damage caused by abiotic stress, such as high light and salt and the hypersensitive defense response of plants., Encodes a protein with mitochondrial serine hydroxymethyltransferase activity, which functions in the photorespiratory pathway, catalyzes the conversion of serine and tetrahydrofolate to glycine and 5,10-methylene tetrahydrofolate. Involved in controlling cell damage caused by abiotic stress, such as high light and salt and the hypersensitive defense response of plants., Encodes a protein with mitochondrial serine hydroxymethyltransferase activity, which functions in the photorespiratory pathway, catalyzes the conversion of serine and tetrahydrofolate to glycine and 5,10-methylene tetrahydrofolate. Involved in controlling cell damage caused by abiotic stress, such as high light and salt and the hypersensitive defense response of plants. |
| 630 | 249927\_at | AT5G19220 | ADG2, APL1 | 12 | -1.2 | -0.1 | -0.1 | 0.1 | -0.1 | -0.3 | -0.3 | -0.1 | -0.6 | -2.4 | -0.5 | -2.6 | -1.4 | -2.6 | -3 | -2.6 | -1.4 | -1.3 | -0.8 | -0.4 | -0.2 | 0 | -0.1 | -0.2 | 0.1 | -0.6 | 0.1 | 0.1 | Encodes the large subunit of ADP-glucose pyrophosphorylase which catalyzes the first, rate limiting step in starch biosynthesis. The large subunit plays a regulatory role whereas the small subunit (ApS) is the catalytic isoform. Four isoforms (ApL1-4) have been identified. ApL1 is the major large subunit isoform present in leaves., Encodes the large subunit of ADP-glucose pyrophosphorylase which catalyzes the first, rate limiting step in starch biosynthesis. The large subunit plays a regulatory role whereas the small subunit (ApS) is the catalytic isoform. Four isoforms (ApL1-4) have been identified. ApL1 is the major large subunit isoform present in leaves. |
| 629 | 258607\_at | AT3G02730 | ATF1, TRXF1 | 3 | -0.7 | 0 | 0 | 0.1 | 0 | -0.1 | -0.1 | -0.3 | -0.3 | -1.3 | -0.5 | -1.5 | -1.1 | -1.2 | -2.7 | -1.8 | -1.3 | -1.1 | -0.4 | -0.1 | 0 | 0 | -0.1 | -0.3 | -0.1 | -1 | 0.2 | -0.1 | ATF1/TRXF1 (THIOREDOXIN F-TYPE 1); thiol-disulfide exchange intermediate; Identical to Thioredoxin F-type 1, chloroplast precursor (TRX-F1) [Arabidopsis Thaliana] (GB:Q9XFH8;GB:Q9M8R5); similar to ATF2/TRXF2 (THIOREDOXIN F2), thiol-disulfide exchange intermediate [Arabidopsis thaliana] (TAIR:AT5G16400.1); similar to Thioredoxin F-type, chloroplast precursor (TRX-F) (GB:O48897); contains InterPro domain Thioredoxin-like fold; (InterPro:IPR012336); contains InterPro domain Thioredoxin-related; (InterPro:IPR006662); contains InterPro domain Thioredoxin domain 2; (InterPro:IPR006663); contains InterPro domain Thioredoxin domain; (InterPro:IPR013766); contains InterPro domain Thioredoxin fold; (InterPro:IPR012335), ATF1/TRXF1 (THIOREDOXIN F-TYPE 1); thiol-disulfide exchange intermediate; Identical to Thioredoxin F-type 1, chloroplast precursor (TRX-F1) [Arabidopsis Thaliana] (GB:Q9XFH8;GB:Q9M8R5); similar to ATF2/TRXF2 (THIOREDOXIN F2), thiol-disulfide exchange intermediate [Arabidopsis thaliana] (TAIR:AT5G16400.1); similar to Thioredoxin F-type, chloroplast precursor (TRX-F) (GB:O48897); contains InterPro domain Thioredoxin-like fold; (InterPro:IPR012336); contains InterPro domain Thioredoxin-related; (InterPro:IPR006662); contains InterPro domain Thioredoxin domain 2; (InterPro:IPR006663); contains InterPro domain Thioredoxin domain; (InterPro:IPR013766); contains InterPro domain Thioredoxin fold; (InterPro:IPR012335) |
| 628 | 250531\_at | AT5G08650 |  | 1 | -1 | 0 | 0 | 0 | 0 | -0.2 | -0.1 | -0.4 | -0.8 | -1.9 | -0.6 | -2 | -1.5 | -1.2 | -2.6 | -2.1 | -2.3 | -1.1 | -0.7 | -0.5 | -0.2 | 0.1 | -0.2 | -0.2 | -0.1 | -0.9 | 0.1 | -0.2 | GTP-binding protein LepA, putative; similar to GTP binding / translation elongation factor [Arabidopsis thaliana] (TAIR:AT5G39900.1); similar to GTP-binding protein LepA [Synechococcus elongatus PCC 7942] (GB:YP\_399498.1); similar to putative LepA protein [Oryza sativa (japonica cultivar-group)] (GB:BAD28023.1); similar to GTP-binding protein LepA [Synechococcus elongatus PCC 6301] (GB:YP\_171750.1); contains InterPro domain Small GTP-binding protein domain; (InterPro:IPR005225); contains InterPro domain Elongation factor G, III and V; (InterPro:IPR009022); contains InterPro domain Elongation factor Tu, domain 2; (InterPro:IPR004161); contains InterPro domain Elongation factor G, C-terminal; (InterPro:IPR000640); contains InterPro domain GTP-binding protein LepA; (InterPro:IPR006297); contains InterPro domain Protein synthesis factor, GTP-binding; (InterPro:IPR000795) |
| 627 | 260284\_at | AT1G80380 |  | 3 | -0.5 | -0.1 | 0.2 | 0.1 | -0.1 | -0.2 | -0.1 | -0.2 | -0.5 | -2.1 | -0.5 | -2 | -1.8 | -1.5 | -2.2 | -1.2 | -1.1 | -0.9 | -0.4 | -0.3 | -0.2 | -0.1 | -0.1 | -0.1 | 0.2 | -0.3 | 0.1 | -0.2 | encodes a glycerate kinase which catalyzes the last step of photorespiration C2 cycle. |
| 626 | 255623\_at | AT4G01310 |  | 2 | -0.2 | -0.1 | 0 | 0.3 | 0 | -0.1 | 0.1 | -0.1 | -0.5 | -2.8 | -0.4 | -2.5 | -2.4 | -2.8 | -5.4 | -3 | -1.1 | -1.4 | -0.1 | -0.4 | 0 | 0.1 | 0 | -0.1 | 0.1 | -0.4 | 0.1 | -0.1 | ribosomal protein L5 family protein; Identical to 50S ribosomal protein L5, chloroplast precursor (RPL5) [Arabidopsis Thaliana] (GB:O04603); similar to 50S ribosomal protein L5, chloroplast (GB:P82192); contains InterPro domain Ribosomal protein L5; (InterPro:IPR002132); contains InterPro domain Mitochondrial ribosomal protein L5; (InterPro:IPR003236) |
| 625 | 261422\_at | AT1G18730 | NDF6 | 3 | -0.6 | -0.1 | -0.1 | 0.2 | 0 | 0 | -0.3 | -0.1 | -1 | -1.3 | -0.4 | -2.2 | -1.9 | -1.9 | -3.2 | -2.4 | -1.3 | -0.8 | -0.3 | -0.3 | -0.1 | 0.1 | -0.1 | -0.1 | 0 | -0.6 | 0.1 | 0 | similar to Os02g0744000 [Oryza sativa (japonica cultivar-group)] (GB:NP\_001048099.1) |
| 624 | 252366\_at | AT3G48420 |  | 2 | -1.2 | -0.3 | -0.1 | 0.1 | 0.1 | -0.1 | -0.1 | -0.3 | -1.4 | -2.8 | -0.8 | -2.7 | -2.4 | -3.5 | -4.4 | -3.7 | -1.9 | -1.7 | -0.7 | -0.4 | -0.3 | -0.1 | -0.2 | -0.2 | 0.1 | -0.8 | 0 | 0 | haloacid dehalogenase-like hydrolase family protein; similar to haloacid dehalogenase-like hydrolase family protein [Arabidopsis thaliana] (TAIR:AT4G39970.1); similar to Os03g0565200 [Oryza sativa (japonica cultivar-group)] (GB:NP\_001050499.1); similar to Putative hydrolase [Oryza sativa (japonica cultivar-group)] (GB:AAK53865.1); contains InterPro domain HAD-superfamily hydrolase subfamily IA, variant 3; (InterPro:IPR006402); contains InterPro domain Haloacid dehalogenase-like hydrolase; (InterPro:IPR005834); contains InterPro domain Haloacid dehalogenase/epoxide hydrolase; (InterPro:IPR005833) |
| 623 | 259603\_at | AT1G56500 |  | 1 | -0.5 | -0.1 | 0 | 0.1 | 0.1 | -0.1 | -0.1 | -0.4 | -0.7 | -1.8 | -0.5 | -1.9 | -1.4 | -1.8 | -2.5 | -2 | -2 | -1.3 | -0.4 | -0.1 | -0.3 | 0.2 | -0.2 | -0.2 | -0.1 | -0.8 | 0.2 | -0.1 | haloacid dehalogenase-like hydrolase family protein; similar to haloacid dehalogenase-like hydrolase family protein [Arabidopsis thaliana] (TAIR:AT4G39970.1); similar to HAD-superfamily hydrolase, subfamily IA, variant 3 containing protein, expressed [Oryza sativa (japonica cultivar-group)] (GB:ABF95582.1); similar to HAD-superfamily hydrolase, subfamily IA, variant 3 containing protein, expressed [Oryza sativa (japonica cultivar-group)] (GB:ABF95581.1); similar to Os03g0311300 [Oryza sativa (japonica cultivar-group)] (GB:NP\_001049921.1); contains InterPro domain HAD-superfamily hydrolase subfamily IA, variant 3; (InterPro:IPR006402); contains InterPro domain Low-density lipoprotein receptor, YWTD repeat; (InterPro:IPR000033); contains InterPro domain Thioredoxin-like fold; (InterPro:IPR012336); contains InterPro domain Redoxin; (InterPro:IPR013740); contains InterPro domain Thioredoxin domain 2; (InterPro:IPR006663); contains InterPro domain Quinoprotein amine dehydrogenase, beta chain-like; (InterPro:IPR011044); contains InterPro domain Thioredoxin fold; (InterPro:IPR012335); contains InterPro domain Thioredoxin-like; (InterPro:IPR011594); contains InterPro domain Haloacid dehalogenase-like hydrolase; (InterPro:IPR005834); contains InterPro domain Haloacid dehalogenase/epoxide hydrolase; (InterPro:IPR005833); contains InterPro domain NHL repeat; (InterPro:IPR001258) |
| 622 | 251157\_at | AT3G63140 | CSP41A | 4 | -0.6 | -0.2 | 0 | 0.2 | -0.1 | -0.1 | 0 | -0.3 | -0.9 | -2.6 | -0.6 | -2.7 | -2.1 | -2.7 | -4.6 | -2.8 | -2.1 | -1.5 | -0.6 | -0.1 | -0.3 | 0.1 | -0.2 | -0.3 | 0.1 | -0.8 | 0.2 | 0.1 | mRNA-binding protein, putative; similar to catalytic/ coenzyme binding [Arabidopsis thaliana] (TAIR:AT1G09340.1); similar to mRNA-binding protein precursor [Nicotiana tabacum] (GB:AAP87140.1); contains InterPro domain NAD-dependent epimerase/dehydratase; (InterPro:IPR001509) |
| 621 | 265073\_at | AT1G55480 |  | 1 | -1.2 | -0.1 | 0.2 | 0.4 | 0 | 0 | -0.1 | -0.3 | -0.5 | -1.5 | -0.4 | -1.8 | -1.4 | -1.8 | -2.5 | -2.8 | -1.1 | -1.4 | -0.3 | 0 | -0.1 | 0 | -0.2 | -0.3 | 0.1 | -0.5 | 0.3 | 0.1 | binding / protein binding; similar to rubredoxin family protein [Arabidopsis thaliana] (TAIR:AT5G17170.2); similar to PDZ/DHR/GLGF; Tetratricopeptide-like helical [Medicago truncatula] (GB:ABE87465.1); contains InterPro domain Tetratricopeptide region; (InterPro:IPR013026); contains InterPro domain PDZ/DHR/GLGF; (InterPro:IPR001478); contains InterPro domain Tetratricopeptide-like helical; (InterPro:IPR011990) |
| 620 | 265247\_at | AT2G43030 |  | 1 | -0.3 | 0 | 0.2 | 0.2 | 0 | -0.2 | 0 | -0.1 | -0.5 | -1.7 | -0.4 | -2.2 | -1.6 | -2.8 | -4 | -2.3 | -1 | -1.1 | -0.1 | -0.2 | -0.3 | 0.1 | 0 | 0 | 0.1 | -0.4 | 0.1 | 0 | ribosomal protein L3 family protein; Identical to 50S ribosomal protein L3-1, chloroplast precursor (RPL3A) [Arabidopsis Thaliana] (GB:Q9SKX4;GB:Q8LEQ9); similar to RPL3P (ribosomal protein large subunit 3P), structural constituent of ribosome [Arabidopsis thaliana] (TAIR:AT3G17465.1); similar to 50S ribosomal protein L3, chloroplast precursor (GB:O80360); contains InterPro domain Translation factor; (InterPro:IPR009000); contains InterPro domain Ribosomal protein L3; (InterPro:IPR000597) |
| 619 | 260898\_at | AT1G29070 |  |  | -0.3 | -0.1 | 0.1 | 0.1 | -0.1 | -0.2 | 0 | 0 | -0.4 | -2.7 | -0.5 | -2.4 | -2.1 | -3.5 | -3.8 | -2.9 | -1.1 | -1.3 | -0.2 | -0.4 | -0.3 | 0 | -0.1 | -0.2 | 0 | -0.4 | 0 | 0 | ribosomal protein L34 family protein; Identical to 50S ribosomal protein L34, chloroplast precursor (CL34) (RPL34) [Arabidopsis Thaliana] (GB:Q9LP37); similar to 50S ribosomal protein L34, chloroplast precursor (CL34) (GB:P82244); contains InterPro domain Ribosomal protein L34; (InterPro:IPR000271) |
| 618 | 251883\_at | AT3G54210 |  | 1 | -0.3 | -0.1 | 0 | 0.1 | 0 | -0.3 | 0 | -0.1 | -0.3 | -1.9 | -0.5 | -1.5 | -1.2 | -2 | -2.5 | -2 | -1 | -0.9 | -0.1 | -0.3 | -0.1 | 0 | -0.1 | -0.1 | 0 | -0.4 | 0 | 0 | ribosomal protein L17 family protein; Identical to 50S ribosomal protein L17, chloroplast precursor (CL17) (RPL17) [Arabidopsis Thaliana] (GB:Q9M385;GB:Q8LCR3); similar to ribosomal protein L17 family protein [Arabidopsis thaliana] (TAIR:AT5G09770.1); similar to 50S ribosomal protein L17, chloroplast precursor (CL17) (GB:O80363); contains InterPro domain Ribosomal protein L17; (InterPro:IPR000456) |
| 617 | 262172\_at | AT1G74970 | RPS9, TWN3 | 3 | -0.3 | -0.2 | 0.2 | 0 | -0.3 | -0.2 | 0.2 | -0.2 | -0.5 | -2.1 | -0.6 | -2.4 | -2.1 | -2.4 | -3 | -2.6 | -1.4 | -1.3 | -0.6 | -0.9 | -0.3 | 0.2 | 0 | 0 | -0.1 | -0.4 | 0 | -0.1 | ribosomal protein S9, nuclear encoded component of the chloroplast ribosome, ribosomal protein S9, nuclear encoded component of the chloroplast ribosome |
| 616 | 247201\_at | AT5G65220 |  | 1 | -0.2 | 0 | 0.2 | 0.1 | -0.1 | -0.1 | 0.3 | -0.1 | -0.6 | -1.3 | -0.2 | -2.2 | -1.7 | -1.6 | -3.3 | -2.5 | -0.7 | -0.9 | -0.2 | -0.4 | -0.5 | 0.1 | 0 | 0 | 0.1 | -0.3 | 0.1 | 0 | ribosomal protein L29 family protein; Identical to 50S ribosomal protein L29, chloroplast precursor (CL29) (RPL29) [Arabidopsis Thaliana] (GB:Q9FJP3); similar to 50S ribosomal protein L29, chloroplast precursor (CL29) (GB:Q9SWI6); contains InterPro domain Ribosomal protein L29; (InterPro:IPR001854) |
| 615 | 258929\_at | AT3G10060 |  | 3 | -0.6 | -0.2 | 0 | 0 | 0 | -0.3 | 0 | 0 | -0.6 | -3.5 | -0.3 | -2.6 | -2 | -3.4 | -4.4 | -2.9 | -2.4 | -1.9 | -0.6 | -0.4 | -0.3 | -0.1 | -0.2 | -0.2 | 0 | -0.6 | 0 | 0.1 | immunophilin, putative / FKBP-type peptidyl-prolyl cis-trans isomerase, putative; similar to immunophilin / FKBP-type peptidyl-prolyl cis-trans isomerase family protein [Arabidopsis thaliana] (TAIR:AT1G20810.1); similar to immunophilin / FKBP-type peptidyl-prolyl cis-trans isomerase-like [Oryza sativa (japonica cultivar-group)] (GB:BAC83911.1); contains InterPro domain Peptidylprolyl isomerase, FKBP-type; (InterPro:IPR001179) |
| 614 | 257168\_at | AT3G24430 | HCF101 | 3 | -0.3 | -0.2 | 0 | -0.1 | -0.1 | -0.3 | 0.1 | -0.1 | -0.7 | -2.2 | -0.2 | -2.3 | -2.1 | -1.4 | -5.8 | -2.9 | -1.5 | -1.5 | -0.4 | -0.3 | -0.3 | 0 | -0.3 | -0.3 | -0.2 | -1.2 | 0.1 | 0 | encodes a MRP-like protein with a nucleotide-binding domain. |
| 613 | 263763\_at | AT2G21385 |  | 2 | -0.6 | -0.3 | -0.2 | -0.1 | -0.1 | -0.7 | -0.1 | 0.1 | -0.6 | -2.8 | -0.3 | -2.2 | -2 | -1.4 | -3.1 | -1.9 | -1.4 | -0.6 | -0.7 | -0.7 | -0.5 | -0.1 | -0.4 | 0 | -0.1 | -1 | 0.1 | 0.2 | similar to Os09g0531100 [Oryza sativa (japonica cultivar-group)] (GB:NP\_001063753.1); similar to conserved hypothetical protein [Medicago truncatula] (GB:ABE89529.1) |
| 612 | 252876\_at | AT4G39970 |  | 1 | -1 | -0.3 | -0.1 | 0.1 | 0 | -0.2 | 0 | -0.2 | -0.7 | -4 | -0.2 | -2.7 | -2.1 | -2 | -2.8 | -1.1 | -3.4 | -0.5 | -1 | -0.5 | 0 | 0 | -0.4 | 0.6 | -0.2 | -1.3 | 0.1 | -0.2 | haloacid dehalogenase-like hydrolase family protein; similar to haloacid dehalogenase-like hydrolase family protein [Arabidopsis thaliana] (TAIR:AT3G48420.1); similar to Os08g0485900 [Oryza sativa (japonica cultivar-group)] (GB:NP\_001062091.1); similar to Predicted haloacid-halidohydrolase and related hydrolases (ISS) [Ostreococcus tauri] (GB:CAL53722.1); contains InterPro domain HAD-superfamily hydrolase subfamily IA, variant 3; (InterPro:IPR006402); contains InterPro domain Haloacid dehalogenase-like hydrolase; (InterPro:IPR005834) |
| 611 | 252132\_at | AT3G50790 |  |  | -0.8 | -0.2 | -0.1 | 0 | 0 | -0.6 | 0 | -0.2 | -0.6 | -2.1 | -0.1 | -1.8 | -1.4 | -2.6 | -2.9 | -1.6 | -1.8 | -1 | -1.1 | -0.6 | -0.5 | -0.1 | -0.5 | 0.2 | -0.4 | -0.8 | 0 | -0.1 | late embryogenesis abundant protein, putative / LEA protein, putative; similar to esterase/lipase/thioesterase family protein [Arabidopsis thaliana] (TAIR:AT1G34340.1); similar to putative late embryonic abundant protein EMB8 [Oryza sativa (japonica cultivar-group)] (GB:BAD25578.1); contains InterPro domain Protein of unknown function UPF0017, hydrolase-like; (InterPro:IPR000952); contains InterPro domain Esterase/lipase/thioesterase; (InterPro:IPR000379); contains InterPro domain AB-hydrolase YheT, putative; (InterPro:IPR012020); contains InterPro domain Alpha/beta hydrolase fold-1; (InterPro:IPR000073) |
| 610 | 250733\_at | AT5G06290 | 2-Cys | 5 | -0.6 | 0 | 0.1 | 0.1 | 0.2 | 0 | -0.2 | -0.3 | -0.1 | -2.1 | -0.7 | -2.2 | -1.6 | -3.4 | -4.2 | -2.4 | -1.3 | -1 | -0.3 | -0.4 | -0.1 | 0.1 | -0.1 | -0.3 | 0 | -0.5 | 0.2 | 0.1 | 2-cys peroxiredoxin, chloroplast, putative; similar to 2-cys peroxiredoxin, chloroplast (BAS1) [Arabidopsis thaliana] (TAIR:AT3G11630.1); similar to 2-Cys peroxiredoxin [Brassica napus] (GB:AAG30570.1); contains InterPro domain Thioredoxin-like fold; (InterPro:IPR012336); contains InterPro domain Thioredoxin fold; (InterPro:IPR012335); contains InterPro domain Alkyl hydroperoxide reductase/ Thiol specific antioxidant/ Mal allergen; (InterPro:IPR000866) |
| 609 | 246792\_at | AT5G27290 |  | 1 | -1 | -0.3 | -0.1 | -0.3 | 0 | -0.1 | -0.3 | -0.3 | -1 | -4.2 | -0.7 | -3 | -2.8 | -4 | -6.6 | -3.3 | -3.9 | -1.9 | -0.6 | -0.2 | -0.3 | -0.1 | -0.5 | -0.7 | -0.6 | -1.5 | 0 | -0.2 | similar to unknown protein [Arabidopsis thaliana] (TAIR:AT1G54680.1); similar to stress regulated protein [Lycopersicon esculentum] (GB:AAW65813.1) |
| 608 | 255440\_at | AT4G02530 |  | 4 | -0.6 | -0.2 | 0.2 | 0.1 | 0 | -0.1 | -0.2 | -0.1 | -0.3 | -1.9 | -0.5 | -2.2 | -2.2 | -3.5 | -3.9 | -1.7 | -1.2 | -1.3 | -0.3 | -0.3 | -0.5 | -0.1 | -0.2 | -0.1 | 0 | -0.8 | 0.1 | 0 | chloroplast thylakoid lumen protein; Identical to Thylakoid lumenal 16.5 kDa protein, chloroplast precursor [Arabidopsis Thaliana] (GB:O22773;GB:Q8L985;GB:Q94BU6;GB:Q9M110); similar to Os06g0705100 [Oryza sativa (japonica cultivar-group)] (GB:NP\_001058514.1) |
| 607 | 248624\_at | AT5G48790 |  | 1 | -1 | -0.2 | 0 | 0 | 0.1 | -0.3 | -0.3 | -0.1 | -0.7 | -1.6 | -0.6 | -2 | -1.8 | -2.5 | -2.7 | -2.4 | -2 | -1 | -0.6 | -0.4 | -0.5 | 0 | -0.2 | -0.1 | -0.4 | -1.2 | 0 | -0.1 | similar to unknown protein [Arabidopsis thaliana] (TAIR:AT1G73060.1); similar to hypothetical protein SBB1\_14t00013 [Solanum bulbocastanum] (GB:AAP45177.2); similar to hypothetical protein SBB1\_21t00009 [Solanum bulbocastanum] (GB:ABI34553.1) |
| 606 | 255078\_at | AT4G09010 | APX4 | 6 | -1.2 | -0.1 | 0 | -0.1 | 0.1 | 0 | 0 | 0 | -0.3 | -2 | -0.4 | -2.3 | -2.7 | -4.5 | -5.5 | -3.8 | -1.6 | -1.3 | -0.4 | -0.3 | -0.4 | 0 | -0.1 | 0 | -0.4 | -0.8 | 0 | -0.2 | Encodes a microsomal ascorbate peroxidase APX4. Ascorbate peroxidases are enzymes that scavenge hydrogen peroxide in plant cells. Eight types of APX have been described for Arabidopsis: three cytosolic (APX1, APX2, APX6), two chloroplastic types (stromal sAPX, thylakoid tAPX), and three microsomal (APX3, APX4, APX5) isoforms. |
| 605 | 263676\_at | AT1G09340 | CRB, CSP41B | 5 | -0.7 | -0.3 | 0 | 0.1 | 0 | -0.2 | -0.1 | 0 | -0.5 | -2.1 | -0.2 | -2.2 | -1.4 | -2.2 | -4.2 | -3 | -1.5 | -1 | -0.2 | -0.3 | -0.3 | 0 | -0.1 | -0.3 | -0.1 | -0.5 | 0.1 | -0.2 | catalytic/ coenzyme binding; similar to mRNA-binding protein, putative [Arabidopsis thaliana] (TAIR:AT3G63140.1); similar to 38 kDa ribosome-associated protein precursor [Chlamydomonas reinhardtii] (GB:AAO22242.1); similar to Os12g0420200 [Oryza sativa (japonica cultivar-group)] (GB:NP\_001066657.1); contains InterPro domain NAD-dependent epimerase/dehydratase; (InterPro:IPR001509), catalytic/ coenzyme binding; similar to mRNA-binding protein, putative [Arabidopsis thaliana] (TAIR:AT3G63140.1); similar to 38 kDa ribosome-associated protein precursor [Chlamydomonas reinhardtii] (GB:AAO22242.1); similar to Os12g0420200 [Oryza sativa (japonica cultivar-group)] (GB:NP\_001066657.1); contains InterPro domain NAD-dependent epimerase/dehydratase; (InterPro:IPR001509) |
| 604 | 251885\_at | AT3G54050 |  | 2 | -1 | -0.2 | 0.1 | 0.1 | -0.1 | 0 | 0 | -0.1 | -0.4 | -2.6 | -0.4 | -2.3 | -1.7 | -1.8 | -4.9 | -2.9 | -1.5 | -1.7 | -0.4 | -0.2 | -0.1 | 0 | -0.2 | -0.4 | -0.1 | -1 | 0.1 | -0.2 | fructose-1,6-bisphosphatase, putative / D-fructose-1,6-bisphosphate 1-phosphohydrolase, putative / FBPase, putative; Identical to Fructose-1,6-bisphosphatase, chloroplast precursor (EC 3.1.3.11) (D- fructose-1,6-bisphosphate 1-phosphohydrolase) (FBPase) (FBP) [Arabidopsis Thaliana] (GB:P25851;GB:Q9M398); similar to fructose-1,6-bisphosphatase, putative / D-fructose-1,6-bisphosphate 1-phosphohydrolase, putative / FBPase, putative [Arabidopsis thaliana] (TAIR:AT1G43670.1); similar to Fructose-1,6-bisphosphatase, chloroplast precursor (D-fructose-1,6-bisphosphate 1-phosphohydrolase) (FBPase) (GB:P22418); similar to fructose-1,6-bisphosphatase precursor [Brassica napus] (GB:AAD12243.1); similar to Fructose-1,6-bisphosphatase, chloroplast precursor (D-fructose-1,6-bisphosphate 1-phosphohydrolase) (FBPase) (GB:Q07204); contains InterPro domain Inositol phosphatase/fructose-1,6-bisphosphatase; (InterPro:IPR000146) |
| 603 | 245198\_at | AT1G67700 |  | 1 | -0.6 | 0.1 | 0 | 0.2 | -0.1 | 0 | -0.1 | 0 | -0.6 | -1.5 | -0.3 | -2.1 | -1.9 | -2 | -3.2 | -2.5 | -1.1 | -1.5 | -0.3 | -0.1 | -0.3 | 0.1 | -0.1 | -0.2 | -0.2 | -0.5 | 0.1 | 0 | similar to At1g67700/F12A21\_30 [Medicago truncatula] (GB:ABE84614.1); contains domain OLIGOPEPTIDASE (PTHR11804:SF2); contains domain PROTEASE M3 THIMET OLIGOPEPTIDASE-RELATED (PTHR11804) |
| 602 | 253547\_at | AT4G30950 | FAD6, FADC, SFD4 | 12 | -0.5 | -0.1 | 0 | 0 | -0.1 | -0.1 | 0.1 | -0.2 | -0.4 | -0.6 | -0.4 | -1.5 | -1.4 | -2.1 | -1.5 | -2 | -1.1 | -0.6 | -0.4 | -0.3 | -0.3 | 0 | -0.1 | -0.1 | -0.2 | -0.6 | -0.1 | -0.1 | Chloroplastic enzyme responsible for the synthesis of 16:2 and 18:2 fatty acids from galactolipids, sulpholipids and phosphatidylglycerol. Uses ferredoxin as electron donor. Gene mutation resulted in reduced level of unsaturated fatty acids leading to susceptibility to photoinhibition., Chloroplastic enzyme responsible for the synthesis of 16:2 and 18:2 fatty acids from galactolipids, sulpholipids and phosphatidylglycerol. Uses ferredoxin as electron donor. Gene mutation resulted in reduced level of unsaturated fatty acids leading to susceptibility to photoinhibition., Chloroplastic enzyme responsible for the synthesis of 16:2 and 18:2 fatty acids from galactolipids, sulpholipids and phosphatidylglycerol. Uses ferredoxin as electron donor. Gene mutation resulted in reduced level of unsaturated fatty acids leading to susceptibility to photoinhibition. |
| 601 | 250006\_at | AT5G18660 | PCB2 | 7 | -0.4 | 0 | 0.3 | 0.2 | 0.1 | 0 | 0 | -0.6 | -1.1 | -3.3 | -1.1 | -3.6 | -3.8 | -6.8 | -7.3 | -4 | -1.9 | -1.9 | -0.5 | -0.7 | -0.5 | 0.2 | -0.2 | 0 | -0.2 | -0.8 | -0.1 | -0.2 | Encodes a protein with 3,8-divinyl protochlorophyllide a 8-vinyl reductase activity. Mutants accumulate divinyl chlorophyll rather than monovinyl chlorophyll. |
| 600 | 267088\_at | AT2G38140 | PSRP4 | 1 | -0.1 | 0.1 | 0.2 | 0 | 0.1 | 0 | 0 | 0 | -0.5 | -2.6 | -0.6 | -2.1 | -1.7 | -3.1 | -3.2 | -2.4 | -1.1 | -0.7 | -0.3 | -0.6 | -0.6 | 0.1 | 0.1 | 0 | -0.2 | -0.6 | 0 | -0.2 | plastid-specific ribosomal protein 4 (PSRP4) mRNA, complete |
| 599 | 266509\_at | AT2G47940 | DEGP2 | 6 | -0.2 | -0.1 | 0.1 | -0.1 | -0.1 | -0.1 | 0 | -0.6 | -0.8 | -2.7 | -0.8 | -2.6 | -2.5 | -2.8 | -3 | -1.8 | -1.2 | -1.8 | -0.4 | -0.4 | -0.4 | -0.1 | -0.1 | -0.2 | -0.2 | -0.9 | 0 | -0.1 | Encodes DegP2 protease (DEGP2); nuclear gene for chloroplast product. |
| 598 | 258897\_at | AT3G05730 |  | 1 | -0.2 | 0 | 0.3 | 0.2 | 0.2 | 0 | 0 | -0.3 | -1.4 | -3.3 | -0.4 | -7 | -2.9 | -7.7 | -4.5 | -2.3 | -2.6 | -0.5 | -0.3 | -0.1 | 0 | 0.2 | 0 | 0.2 | 0.2 | -0.6 | 0.1 | 0 | Encodes a defensin-like (DEFL) family protein. |
| 597 | 249785\_at | AT5G24300 | ATSS1, SSI, SSI1 | 9 | -0.3 | -0.1 | 0.1 | 0 | -0.2 | 0 | -0.2 | -0.3 | -0.7 | -1.7 | -0.6 | -2.4 | -2 | -1.3 | -3.3 | -2 | -1.7 | -1.1 | -0.7 | -0.2 | -0.3 | 0.1 | -0.1 | 0.1 | -0.4 | -1.2 | 0.1 | -0.2 | SSI is a plastidial enzyme and crucial for the synthesis of normal amylopectin in the leaves of Arabidopsis. The absence of SSI results in a deficiency in the number of shorter glucans which in turn affect the formation and connection of the amylopectin clusters in starch., SSI is a plastidial enzyme and crucial for the synthesis of normal amylopectin in the leaves of Arabidopsis. The absence of SSI results in a deficiency in the number of shorter glucans which in turn affect the formation and connection of the amylopectin clusters in starch., SSI is a plastidial enzyme and crucial for the synthesis of normal amylopectin in the leaves of Arabidopsis. The absence of SSI results in a deficiency in the number of shorter glucans which in turn affect the formation and connection of the amylopectin clusters in starch. |
| 596 | 266570\_at | AT2G24090 |  | 1 | -0.2 | 0 | 0.2 | 0.1 | 0 | -0.1 | 0.2 | -0.2 | -0.3 | -2.7 | -0.5 | -2.8 | -1.9 | -2.4 | -4 | -2.6 | -1.1 | -1.1 | 0 | 0 | -0.4 | 0.1 | -0.1 | -0.1 | -0.1 | -0.5 | -0.2 | -0.1 | ribosomal protein L35 family protein; similar to 50S ribosomal protein L35, chloroplast precursor (CL35) (GB:P23326); contains InterPro domain Ribosomal protein L35; (InterPro:IPR001706) |
| 595 | 259981\_at | AT1G76450 |  | 3 | -0.7 | 0 | 0.1 | -0.2 | -0.1 | -0.4 | -0.1 | -0.1 | -0.3 | -2 | -0.5 | -2.2 | -1.9 | -2.6 | -2.6 | -1.9 | -1.7 | -1.4 | -0.3 | -0.2 | -0.3 | 0.1 | -0.4 | -0.1 | -0.2 | -0.8 | -0.3 | -0.4 | oxygen-evolving complex-related; Identical to Unknown thylakoid lumen protein, chloroplast precursor [Arabidopsis Thaliana] (GB:Q9S720); similar to thylakoid lumen protein, chloroplast precursor-related [Medicago truncatula] (GB:ABE82597.1); similar to Os08g0512500 [Oryza sativa (japonica cultivar-group)] (GB:NP\_001062219.1) |
| 594 | 259193\_at | AT3G01480 | CYP38 | 4 | -0.4 | 0 | 0.1 | 0 | -0.1 | -0.4 | -0.1 | -0.4 | -0.3 | -2.9 | -0.7 | -2.6 | -2 | -3.1 | -4.6 | -1.9 | -1.5 | -1.6 | -0.3 | -0.6 | -0.8 | 0 | -0.2 | -0.1 | -0.4 | -1 | -0.2 | -0.3 | peptidyl-prolyl cis-trans isomerase, putative / cyclophilin, putative / rotamase, putative; similar to peptidyl-prolyl cis-trans isomerase TLP38, chloroplast / thylakoid lumen PPIase of 38 kDa / cyclophilin / rotamase [Arabidopsis thaliana] (TAIR:AT3G15520.1); similar to Peptidyl-prolyl cis-trans isomerase, chloroplast precursor (40 kDa thylakoid lumen PPIase) (40 kDa thylakoid lumen rotamase) (GB:O49939); contains InterPro domain Peptidyl-prolyl cis-trans isomerase, cyclophilin type; (InterPro:IPR002130) |
| 593 | 252032\_at | AT3G52150 |  | 1 | -0.3 | 0.1 | 0 | 0 | 0.1 | -0.3 | 0 | -0.1 | -0.1 | -2.1 | -0.4 | -1.8 | -1.7 | -2.7 | -3.7 | -2.6 | -1 | -1.4 | 0 | -0.2 | -0.3 | 0 | -0.1 | -0.1 | -0.2 | -0.6 | -0.1 | -0.2 | RNA binding; similar to CP33 (PIGMENT DEFECTIVE 322), RNA binding [Arabidopsis thaliana] (TAIR:AT3G52380.1); similar to Plastid-specific 30S ribosomal protein 2, chloroplast precursor (PSRP-2) (GB:P82277); contains InterPro domain Paraneoplastic encephalomyelitis antigen; (InterPro:IPR002343); contains InterPro domain Nucleotide-binding, alpha-beta plait; (InterPro:IPR012677); contains InterPro domain Ankyrin; (InterPro:IPR002110); contains InterPro domain RNA-binding region RNP-1 (RNA recognition motif); (InterPro:IPR000504), RNA recognition motif (RRM)-containing protein; similar to CP33 (PIGMENT DEFECTIVE 322), RNA binding [Arabidopsis thaliana] (TAIR:AT3G52380.1); similar to Plastid-specific 30S ribosomal protein 2, chloroplast precursor (PSRP-2) (GB:P82277); contains InterPro domain Paraneoplastic encephalomyelitis antigen; (InterPro:IPR002343); contains InterPro domain Nucleotide-binding, alpha-beta plait; (InterPro:IPR012677); contains InterPro domain Ankyrin; (InterPro:IPR002110); contains InterPro domain RNA-binding region RNP-1 (RNA recognition motif); (InterPro:IPR000504) |
| 592 | 256215\_at | AT1G50900 |  | 1 | -0.4 | 0 | 0.1 | -0.1 | 0 | -0.6 | -0.2 | -0.1 | -0.2 | -2.7 | -0.3 | -2 | -1.4 | -2 | -3.4 | -2.5 | -1.7 | -1.1 | -0.1 | -0.1 | -0.3 | 0 | -0.1 | -0.1 | -0.6 | -1.1 | -0.2 | -0.3 | similar to unnamed protein product [Ostreococcus tauri] (GB:CAL57548.1); similar to Os07g0520800 [Oryza sativa (japonica cultivar-group)] (GB:NP\_001059804.1); contains InterPro domain Ankyrin; (InterPro:IPR002110) |
| 591 | 245852\_at | AT5G13510 |  | 2 | -0.3 | -0.1 | 0 | -0.1 | 0 | -0.3 | 0.1 | 0 | -0.2 | -2.1 | -0.4 | -2 | -1.6 | -2.1 | -3.1 | -2 | -1.3 | -0.8 | -0.1 | -0.3 | -0.3 | -0.1 | -0.1 | -0.2 | -0.4 | -0.9 | -0.3 | -0.2 | ribosomal protein L10 family protein; Identical to 50S ribosomal protein L10, chloroplast precursor (CL10) (RPL10) [Arabidopsis Thaliana] (GB:Q9FY50); similar to ribosomal protein L10 family protein [Arabidopsis thaliana] (TAIR:AT3G12370.1); similar to 50S ribosomal protein L10, chloroplast precursor (CL10) (GB:O80362); contains InterPro domain Ribosomal protein L10; (InterPro:IPR001790) |
| 590 | 253283\_at | AT4G34090 |  | 3 | -0.6 | 0 | 0.3 | 0.1 | 0.1 | -0.1 | -0.3 | -0.2 | -0.9 | -2.2 | -0.5 | -2.5 | -2 | -2.2 | -3.5 | -3.7 | -1.7 | -1.1 | -0.3 | -0.4 | -0.4 | 0.1 | -0.3 | -0.3 | -0.6 | -1.9 | -0.3 | -0.6 | similar to unknown protein [Arabidopsis thaliana] (TAIR:AT2G23370.1); similar to Hypothetical protein [Oryza sativa (japonica cultivar-group)] (GB:AAN61482.1); similar to Os03g0109700 [Oryza sativa (japonica cultivar-group)] (GB:NP\_001048710.1); contains domain TPR-like (SSF48452) |
| 589 | 248287\_at | AT5G52970 |  | 4 | -0.5 | -0.1 | 0 | 0 | 0.1 | -0.2 | 0 | -0.1 | -1.1 | -3 | -0.5 | -2.5 | -2.7 | -4.4 | -5.2 | -3.6 | -2.6 | -1.6 | -0.4 | -0.5 | -0.3 | 0 | -0.2 | -0.2 | -0.4 | -0.9 | -0.2 | -0.4 | thylakoid lumen 15.0 kDa protein; similar to unnamed protein product [Ostreococcus tauri] (GB:CAL58595.1) |
| 588 | 261190\_at | AT1G32990 | PRPL11 | 7 | -0.2 | -0.1 | 0.2 | 0.1 | 0.1 | -0.1 | 0.1 | 0 | -0.4 | -2.9 | -0.4 | -2.1 | -2.2 | -2.5 | -3.9 | -2.7 | -1.2 | -1.1 | -0.1 | -0.4 | -0.6 | 0.1 | -0.1 | -0.1 | -0.1 | -0.5 | 0 | -0.1 | mutant has Decreased effective quantum yield of photosystem II; Pale green plants; Reduced growth rate; Plastid Ribosomal Protein L11 |
| 587 | 245357\_at | AT4G17560 |  | 1 | -0.4 | -0.1 | 0.1 | 0 | 0 | -0.1 | -0.3 | 0 | -0.6 | -2.9 | -0.2 | -2.4 | -2.5 | -2.5 | -5.8 | -3 | -1.1 | -2.1 | -0.3 | -0.5 | -0.6 | 0 | -0.1 | -0.1 | -0.3 | -0.7 | 0 | -0.3 | ribosomal protein L19 family protein; Identical to 50S ribosomal protein L19-1, chloroplast precursor [Arabidopsis Thaliana] (GB:Q8W463;GB:O23597); similar to ribosomal protein L19 family protein [Arabidopsis thaliana] (TAIR:AT5G47190.1); similar to plastid ribosomal protein L19, putative [Brassica oleracea] (GB:ABD65070.1); contains InterPro domain Ribosomal protein L19; (InterPro:IPR001857) |
| 586 | 262029\_at | AT1G35680 |  | 2 | -0.2 | 0.1 | 0.1 | 0 | 0 | -0.1 | 0 | 0 | -0.4 | -2.6 | -0.3 | -1.7 | -1.6 | -2.8 | -4.4 | -2.5 | -1.2 | -1.4 | -0.2 | -0.3 | -0.2 | 0 | -0.1 | -0.1 | -0.3 | -0.4 | -0.1 | -0.1 | 50S ribosomal protein L21, chloroplast / CL21 (RPL21); Identical to 50S ribosomal protein L21, chloroplast precursor (CL21) (RPL21) [Arabidopsis Thaliana] (GB:P51412;GB:Q9SMM2); similar to NFD1 (NUCLEAR FUSION DEFECTIVE 1), structural constituent of ribosome [Arabidopsis thaliana] (TAIR:AT4G30930.1); similar to Os02g0259600 [Oryza sativa (japonica cultivar-group)] (GB:NP\_001046479.1); similar to 50S ribosomal protein L21, chloroplast precursor (CL21) (CS-L7) (GB:P24613); contains InterPro domain Ribosomal protein L21; (InterPro:IPR001787) |
| 585 | 260165\_at | AT1G79850 | CS17, PRPS17, RPS17 | 5 | -0.2 | 0 | 0.1 | 0.2 | -0.1 | -0.2 | 0 | 0 | -0.3 | -2.5 | -0.5 | -2.2 | -1.7 | -2.1 | -4 | -2.1 | -1.1 | -1.1 | -0.2 | -0.5 | -0.6 | 0.2 | -0.1 | -0.1 | -0.2 | -0.5 | -0.1 | -0.2 | nuclear-encoded 30S chloroplast ribosomal protein S17, nuclear-encoded 30S chloroplast ribosomal protein S17, nuclear-encoded 30S chloroplast ribosomal protein S17 |
| 584 | 261954\_at | AT1G64510 |  | 1 | -0.3 | 0 | 0 | 0.1 | 0 | -0.1 | 0 | 0 | -0.4 | -2.9 | -0.5 | -2.3 | -2 | -3.5 | -4.3 | -2.8 | -1.3 | -0.9 | -0.2 | -0.4 | -0.5 | 0.1 | 0 | -0.1 | -0.1 | -0.4 | -0.1 | 0 | ribosomal protein S6 family protein; Identical to 30S ribosomal protein S6 alpha, chloroplast precursor (RPS6) [Arabidopsis Thaliana] (GB:Q8VY91;GB:Q9SGW4); similar to 30S ribosomal protein S6 alpha, chloroplast precursor [Contains: 30S ribosomal protein S6 beta; 30S ribosomal protein S6 gamma; 30S ribosomal protein S6 delta; 30S ribosomal protein S6 epsilon] (GB:P82403); contains InterPro domain Ribosomal protein S6; (InterPro:IPR000529) |
| 583 | 267172\_at | AT2G37660 |  | 1 | -0.4 | -0.2 | 0 | -0.1 | 0 | -0.4 | 0 | -0.1 | 0.2 | -2.6 | -0.3 | -1.6 | -1.4 | -2.7 | -3.9 | -1.8 | -1 | -0.6 | 0.1 | -0.2 | -0.2 | -0.1 | -0.1 | -0.2 | -0.2 | -0.7 | 0 | -0.1 | catalytic/ coenzyme binding; Identical to Protein At2g37660, chloroplast precursor [Arabidopsis Thaliana] (GB:O80934;GB:Q94B61); similar to catalytic/ coenzyme binding [Arabidopsis thaliana] (TAIR:AT5G02240.1); similar to Os05g0110300 [Oryza sativa (japonica cultivar-group)] (GB:NP\_001054439.1); similar to Os03g0822200 [Oryza sativa (japonica cultivar-group)] (GB:NP\_001051733.1); similar to similar to Nucleoside-diphosphate-sugar epimerases [Crocosphaera watsonii WH 8501] (GB:ZP\_00516553.1); contains InterPro domain NAD-dependent epimerase/dehydratase; (InterPro:IPR001509) |
| 582 | 246033\_at | AT5G08280 | HEMC | 4 | -0.3 | 0 | 0.2 | 0 | 0 | -0.4 | 0.1 | 0 | 0.2 | -1.9 | -0.2 | -1.5 | -1.1 | -2.1 | -2.4 | -1.1 | -1.1 | -1.4 | -0.1 | -0.5 | -0.2 | -0.1 | 0.1 | 0 | -0.5 | -1.1 | 0 | 0 | Encodes a protein with porphobilinogen deaminase activity. This protein is targeted to the chloroplast. |
| 581 | 251118\_at | AT3G63410 | APG1, VTE3 | 5 | -0.2 | -0.1 | -0.1 | 0 | 0 | -0.2 | 0.2 | 0 | 0 | -1.7 | -0.3 | -1.8 | -1.4 | -2.2 | -3.4 | -2 | -1.1 | -0.7 | -0.3 | 0 | 0.4 | -0.1 | 0 | -0.1 | -0.2 | -0.8 | 0 | -0.1 | Encodes a MPBQ/MSBQ methyltransferase located in the chloroplast inner envelope membrane. Mutant plants lack plastoquinone (PQ), suggesting that the APG1 protein is involved in the methylation step of PQ biosynthesis. The gene product is also involved in tocopherol (vitamin E) biosynthesis., Encodes a MPBQ/MSBQ methyltransferase located in the chloroplast inner envelope membrane. Mutant plants lack plastoquinone (PQ), suggesting that the APG1 protein is involved in the methylation step of PQ biosynthesis. The gene product is also involved in tocopherol (vitamin E) biosynthesis. |
| 580 | 248449\_at | AT5G51110 |  | 1 | -0.7 | -0.2 | -0.1 | 0.2 | 0 | -0.7 | 0 | 0.1 | 0 | -2.3 | -0.3 | -2.3 | -1.8 | -1.3 | -3.5 | -2.5 | -1.4 | -1.3 | -0.2 | -0.1 | -0.1 | -0.1 | -0.4 | -0.2 | -0.4 | -0.9 | 0 | -0.1 | similar to dehydratase family [Arabidopsis thaliana] (TAIR:AT1G29810.1); similar to expressed protein [Oryza sativa (japonica cultivar-group)] (GB:ABF93463.1); contains InterPro domain Transcriptional coactivator/pterin dehydratase; (InterPro:IPR001533) |
| 579 | 260877\_at | AT1G21500 |  | 1 | -0.7 | 0 | 0.1 | 0.1 | 0.1 | -0.3 | 0 | -0.2 | -0.8 | -3.8 | -0.9 | -1.9 | -2.2 | -6.7 | -4.3 | -3.2 | -2.5 | -1.4 | -0.4 | -0.1 | 0 | -0.2 | -0.2 | -0.2 | -0.8 | -1.3 | -0.2 | -0.4 | similar to conserved hypothetical protein [Medicago truncatula] (GB:ABE83904.1) |
| 578 | 255046\_at | AT4G09650 | ATPD | 6 | -0.3 | -0.2 | 0 | 0.1 | 0 | -0.3 | -0.1 | -0.2 | -0.3 | -2 | -0.7 | -1.5 | -1.5 | -2.9 | -3.4 | -2.4 | -1.4 | -1.4 | -0.3 | -0.1 | -0.1 | -0.1 | -0.1 | -0.2 | -0.5 | -1.1 | -0.2 | -0.2 | ATP synthase delta chain, chloroplast, putative / H(+)-transporting two-sector ATPase, delta (OSCP) subunit, putative; similar to ATP synthase delta chain, mitochondrial, putative / H(+)-transporting two-sector ATPase, delta (OSCP) subunit, putative [Arabidopsis thaliana] (TAIR:AT5G13450.1); similar to ATP synthase delta chain, chloroplast precursor (GB:P32980); contains InterPro domain ATPase, F1 complex, OSCP/delta subunit; (InterPro:IPR000711) |
| 577 | 258989\_at | AT3G08920 |  | 1 | -1.2 | -0.2 | -0.1 | 0 | -0.1 | -0.4 | 0.2 | 0 | -1.1 | -4 | -0.4 | -1.8 | -2.9 | -5.2 | -5.3 | -3.2 | -2.6 | -1.7 | -0.3 | -0.2 | 0.1 | -0.1 | -0.3 | -0.2 | -0.4 | -1.3 | -0.2 | -0.2 | rhodanese-like domain-containing protein; similar to rhodanese-like domain-containing protein [Arabidopsis thaliana] (TAIR:AT2G42220.1); similar to Os09g0279400 [Oryza sativa (japonica cultivar-group)] (GB:NP\_001062759.1); similar to Os03g0289400 [Oryza sativa (japonica cultivar-group)] (GB:NP\_001049793.1); similar to Os02g0596000 [Oryza sativa (japonica cultivar-group)] (GB:NP\_001047318.1); contains InterPro domain Rhodanese-like; (InterPro:IPR001763) |
| 576 | 266329\_at | AT2G01590 | CRR3 | 1 | -1.5 | -0.2 | 0 | 0.2 | 0 | -0.6 | 0 | -0.2 | -1.1 | -3.6 | -0.5 | -2.7 | -2.3 | -3.2 | -4.9 | -4.1 | -1.6 | -1.2 | -0.8 | -0.3 | -0.5 | 0 | -0.2 | -0.4 | -0.9 | -1.5 | 0 | -0.4 | unknown protein |
| 575 | 258055\_at | AT3G16250 | NDF4 |  | -0.8 | -0.2 | 0.1 | 0.1 | 0.1 | -0.7 | -0.1 | -0.2 | -1 | -4.8 | -0.7 | -2.9 | -3.1 | -3.9 | -8.7 | -5.5 | -2.4 | -1.9 | -0.6 | -0.5 | -0.7 | -0.2 | -0.7 | -0.4 | -1.1 | -1.9 | -0.3 | -0.7 | ferredoxin-related; similar to ferredoxin-related [Arabidopsis thaliana] (TAIR:AT4G32590.1); similar to Ferredoxin [Medicago truncatula] (GB:ABE80924.1); contains InterPro domain 2Fe-2S Ferredoxin-like fold; (InterPro:IPR012675); contains InterPro domain Ferredoxin; (InterPro:IPR001041) |
| 574 | 262168\_at | AT1G74730 |  | 1 | -0.9 | -0.1 | 0.1 | 0.1 | -0.2 | -0.4 | 0 | -0.2 | -0.6 | -1.9 | -0.4 | -1.7 | -2 | -2.7 | -3 | -2 | -1.2 | -0.6 | -0.4 | -0.2 | -0.1 | -0.1 | -0.3 | -0.1 | -0.5 | -0.9 | 0 | -0.3 | similar to unknown protein [Arabidopsis thaliana] (TAIR:AT5G08050.1); similar to Os12g0575000 [Oryza sativa (japonica cultivar-group)] (GB:NP\_001067105.1); similar to Os01g0585300 [Oryza sativa (japonica cultivar-group)] (GB:NP\_001043425.1); contains InterPro domain Protein of unknown function DUF1118; (InterPro:IPR009500) |
| 573 | 257219\_at | AT3G14930 | HEME1 | 3 | -0.7 | -0.1 | -0.2 | 0.1 | -0.1 | -0.5 | -0.1 | -0.5 | -0.2 | -2.9 | -0.8 | -2.4 | -1.9 | -4 | -3.6 | -2.2 | -1.4 | -1.2 | -0.2 | -0.4 | -0.1 | -0.3 | -0.4 | -0.1 | -0.5 | -1.1 | -0.3 | 0 | HEME1; uroporphyrinogen decarboxylase; similar to HEME2, uroporphyrinogen decarboxylase [Arabidopsis thaliana] (TAIR:AT2G40490.1); similar to Uroporphyrinogen decarboxylase (ISS) [Ostreococcus tauri] (GB:CAL57745.1); similar to Os01g0622300 [Oryza sativa (japonica cultivar-group)] (GB:NP\_001043610.1); contains InterPro domain Uroporphyrinogen decarboxylase (URO-D); (InterPro:IPR000257); contains InterPro domain Uroporphyrinogen decarboxylase HemE; (InterPro:IPR006361) |
| 572 | 250563\_at | AT5G08050 |  | 2 | -0.4 | -0.1 | 0 | 0.3 | 0 | -0.4 | 0.2 | -0.2 | -0.3 | -2.4 | -0.7 | -2.6 | -2.1 | -3.9 | -4.5 | -3.4 | -1.3 | -1.3 | -0.2 | -0.4 | -0.4 | 0 | -0.2 | -0.2 | -0.4 | -1 | 0.2 | -0.1 | similar to unknown protein [Arabidopsis thaliana] (TAIR:AT1G74730.1); similar to Os12g0575000 [Oryza sativa (japonica cultivar-group)] (GB:NP\_001067105.1); similar to hypothetical protein LOC\_Os11g27300 [Oryza sativa (japonica cultivar-group)] (GB:ABA93451.1); contains InterPro domain Protein of unknown function DUF1118; (InterPro:IPR009500) |
| 571 | 248798\_at | AT5G47190 |  | 1 | -0.3 | 0 | 0.1 | 0.1 | 0.1 | -0.2 | 0 | -0.2 | -0.6 | -2.9 | -0.6 | -2.8 | -2.2 | -3.3 | -5.1 | -3.4 | -1.5 | -0.9 | -0.2 | -0.5 | -0.4 | -0.1 | -0.2 | -0.3 | -0.2 | -0.7 | 0 | -0.1 | ribosomal protein L19 family protein; Identical to 50S ribosomal protein L19-2, chloroplast precursor [Arabidopsis Thaliana] (GB:Q8RXX5;GB:Q8LBV7;GB:Q9LVU0); similar to ribosomal protein L19 family protein [Arabidopsis thaliana] (TAIR:AT4G17560.1); similar to plastid ribosomal protein L19, putative [Brassica oleracea] (GB:ABD64915.1); contains InterPro domain Ribosomal protein L19; (InterPro:IPR001857) |
| 570 | 248962\_at | AT5G45680 |  | 2 | -0.6 | -0.2 | 0.1 | 0.2 | 0 | -0.4 | 0 | 0.1 | -0.7 | -2.7 | -0.4 | -2.6 | -2.2 | -3.6 | -6.9 | -3.4 | -2 | -1.5 | -0.1 | -0.3 | -0.5 | -0.1 | -0.2 | -0.4 | -0.3 | -1.2 | -0.1 | -0.2 | FK506-binding protein 1 (FKBP13); Identical to FKBP-type peptidyl-prolyl cis-trans isomerase 3, chloroplast precursor (EC 5.2.1.8) (PPIase) (Rotamase) (AtFKBP13) (FK506-binding protein 1) (FKBP13) [Arabidopsis Thaliana] (GB:Q9SCY2;GB:Q8M8T4); similar to immunophilin, putative / FKBP-type peptidyl-prolyl cis-trans isomerase, putative [Arabidopsis thaliana] (TAIR:AT4G39710.1); similar to Chain A, Crystal Structure Of Atfkbp13 (GB:1U79); contains InterPro domain Peptidylprolyl isomerase, FKBP-type; (InterPro:IPR001179) |
| 569 | 246548\_at | AT5G14910 |  | 1 | -0.4 | -0.1 | 0 | 0.1 | 0 | -0.3 | 0.1 | 0 | -0.4 | -2.1 | -0.3 | -1.9 | -1.5 | -2.4 | -3.4 | -2.4 | -1.3 | -1.5 | -0.1 | -0.4 | -0.4 | 0 | -0.2 | -0.2 | -0.2 | -0.7 | 0 | -0.2 | heavy-metal-associated domain-containing protein; similar to Os05g0337400 [Oryza sativa (japonica cultivar-group)] (GB:NP\_001055238.1); similar to unknown protein [Oryza sativa (japonica cultivar-group)] (GB:AAV32180.1); contains InterPro domain Heavy metal transport/detoxification protein; (InterPro:IPR006121) |
| 568 | 267635\_at | AT2G42220 |  | 1 | -0.6 | 0.1 | 0.1 | 0.3 | 0 | -0.3 | 0 | -0.1 | -0.9 | -3.7 | -0.7 | -2.7 | -2.4 | -5.2 | -4.9 | -3.6 | -2.2 | -1.5 | -0.4 | -0.3 | -0.3 | 0.1 | -0.1 | 0.1 | -0.3 | -1.2 | 0.1 | -0.1 | rhodanese-like domain-containing protein; similar to rhodanese-like domain-containing protein [Arabidopsis thaliana] (TAIR:AT3G08920.1); similar to Os09g0279400 [Oryza sativa (japonica cultivar-group)] (GB:NP\_001062759.1); similar to Os03g0289400 [Oryza sativa (japonica cultivar-group)] (GB:NP\_001049793.1); similar to Os02g0596000 [Oryza sativa (japonica cultivar-group)] (GB:NP\_001047318.1); contains InterPro domain Rhodanese-like; (InterPro:IPR001763) |
| 567 | 258076\_at | AT3G25920 | RPL15 | 2 | -0.5 | 0.1 | 0.1 | 0.1 | 0 | -0.2 | 0.1 | -0.1 | -0.4 | -2.9 | -0.6 | -2.1 | -1.8 | -3.4 | -4.4 | -2.6 | -1.2 | -1.2 | -0.1 | -0.3 | -0.3 | 0 | 0 | -0.1 | 0 | -0.5 | 0.1 | -0.2 | encodes a plastid ribosomal protein CL15, a constituent of the large subunit of the ribosomal complex |
| 566 | 254105\_at | AT4G25080 | CHLM | 4 | -1 | 0 | -0.2 | 0.2 | 0 | -0.7 | 0.2 | -0.2 | -0.4 | -3.5 | -0.5 | -1.8 | -1.8 | -3.3 | -4.1 | -2.9 | -2 | -1.7 | -0.6 | -0.4 | -0.5 | -0.2 | -0.3 | -0.5 | -0.7 | -1.3 | -0.1 | -0.2 | Encodes a protein with methyltransferase activity responsible for the methylation of magnesium protoporphyrin IX. Mutants defective in this gene are affected in chlorophyll biosynthesis and show a reduction in the accumulation of a number of major thylakoid-associated proteins including components of PSI (LHCI), PSII (LHCII, D1, CP43) and the cytochrome b6f complex (Cytf). By contrast, no significant changes were detected for the proteins of the stroma and the chloroplast envelope. |
| 565 | 246294\_at | AT3G56910 | PSRP5 | 1 | -0.3 | 0 | 0.1 | 0.1 | 0 | -0.3 | 0.1 | 0 | -0.1 | -2.2 | -0.2 | -1.8 | -1.4 | -2.1 | -3.2 | -2.1 | -1.1 | -0.7 | -0.1 | -0.2 | -0.2 | -0.1 | 0 | -0.1 | -0.1 | -0.5 | 0 | -0.1 | PSRP5 (PLASTID-SPECIFIC 50S RIBOSOMAL PROTEIN 5); Identical to Plastid-specific 50S ribosomal protein 5, chloroplast precursor (PSRP- 5) (PSRP5) [Arabidopsis Thaliana] (GB:Q9LER7;GB:Q94B31); similar to Os01g0278900 [Oryza sativa (japonica cultivar-group)] (GB:NP\_001042743.1); similar to 50s ribosomal protein l18, chloroplast precursor (cl18). [Medicago truncatula] (GB:ABE79862.1) |
| 564 | 255457\_at | AT4G02770 | PSAD-1 | 5 | -0.1 | 0 | -0.1 | 0.2 | 0 | -0.2 | 0.1 | -0.1 | -0.2 | -0.9 | -0.2 | -0.9 | -0.4 | -1 | -1.7 | -1.5 | -0.7 | -0.7 | -0.2 | -0.1 | -0.1 | -0.1 | -0.1 | -0.2 | -0.1 | -0.4 | 0.1 | 0.1 | Encodes a protein predicted by sequence similarity with spinach PsaD to be photosystem I reaction center subunit II (PsaD1) |
| 563 | 245354\_at | AT4G17600 | LIL3:1 | 4 | -0.2 | -0.1 | 0 | 0 | 0.1 | -0.5 | -0.1 | -0.3 | -0.3 | -2.1 | -0.6 | -1.8 | -1.5 | -2 | -3.1 | -2.3 | -1.7 | -1.4 | -0.2 | -0.3 | -0.4 | 0.1 | -0.2 | -0.3 | -0.5 | -1.1 | -0.2 | 0 | Encodes Lil3:1 (light-harvesting-like) protein. Belongs to the Lhc super-gene family encodes the light-harvesting chlorophyll a/b-binding (LHC) proteins that constitute the antenna system of the photosynthetic apparatus. A generic LHC motif is present in Lil3:1. |
| 562 | 265966\_at | AT2G37220 |  | 4 | -0.3 | 0 | 0.1 | 0 | 0 | -0.1 | 0 | -0.1 | -0.3 | -0.5 | -0.2 | -1.7 | -1.3 | -1.1 | -2.9 | -1.6 | -0.9 | -1.7 | 0.2 | -0.3 | 0.1 | 0.1 | -0.1 | -0.3 | -0.3 | -0.8 | -0.1 | -0.3 | 29 kDa ribonucleoprotein, chloroplast, putative / RNA-binding protein cp29, putative; Identical to Putative ribonucleoprotein At2g37220, chloroplast precursor [Arabidopsis Thaliana] (GB:Q9ZUU4); similar to CP29 (chloroplast 29 kDa ribonucleoprotein), RNA binding [Arabidopsis thaliana] (TAIR:AT3G53460.2); similar to 29 kDa ribonucleoprotein A, chloroplast precursor (CP29A) (GB:Q08935); contains InterPro domain Nucleotide-binding, alpha-beta plait; (InterPro:IPR012677); contains InterPro domain RNA-binding region RNP-1 (RNA recognition motif); (InterPro:IPR000504) |
| 561 | 264575\_at | AT1G05190 | emb2394 | 1 | -0.3 | -0.2 | 0.1 | -0.2 | -0.1 | -0.1 | -0.2 | -0.1 | -0.9 | -1.4 | -0.3 | -2.4 | -1.8 | -2.1 | -3 | -2.3 | -1.2 | -1.5 | 0.1 | -0.2 | 0 | 0.1 | -0.2 | -0.2 | -0.4 | -0.6 | -0.1 | -0.4 | EMB2394 (EMBRYO DEFECTIVE 2394); structural constituent of ribosome; Identical to 50S ribosomal protein L6, chloroplast precursor (RPL6) [Arabidopsis Thaliana] (GB:O23049); similar to ribosomal protein L6 family protein [Arabidopsis thaliana] (TAIR:AT2G18400.1); similar to Os03g0751400 [Oryza sativa (japonica cultivar-group)] (GB:NP\_001051292.1); similar to putative ribosomal protein CtrL6e, 5'-partial [Oryza sativa (japonica cultivar-group)] (GB:AAP68351.1); similar to Os03g0356300 [Oryza sativa (japonica cultivar-group)] (GB:NP\_001050139.1); contains InterPro domain Ribosomal protein L6; (InterPro:IPR000702); contains InterPro domain Ribosomal protein L6, signature 1; (InterPro:IPR002358) |
| 560 | 248380\_at | AT5G51820 | ATPGMP, PGM, PGM1, STF1 | 36 | -0.6 | 0.1 | 0.1 | -0.2 | -0.2 | -0.2 | -0.4 | -0.1 | -0.5 | -0.9 | -0.2 | -2.7 | -2 | -1.8 | -5.6 | -1.9 | -1.3 | -1 | -0.1 | -0.1 | 0.2 | 0.3 | -0.1 | 0.1 | -0.3 | -1.3 | 0.1 | -0.3 | Encodes a plastid isoform of the enzyme phosphoglucomutase involved in controlling photosynthetic carbon flow. Effective petiole movement against the direction of the gravity requires functional PGM activity that is required for full development of amyloplasts., Encodes a plastid isoform of the enzyme phosphoglucomutase involved in controlling photosynthetic carbon flow. Effective petiole movement against the direction of the gravity requires functional PGM activity that is required for full development of amyloplasts., Encodes a plastid isoform of the enzyme phosphoglucomutase involved in controlling photosynthetic carbon flow. Effective petiole movement against the direction of the gravity requires functional PGM activity that is required for full development of amyloplasts., Encodes a plastid isoform of the enzyme phosphoglucomutase involved in controlling photosynthetic carbon flow. Effective petiole movement against the direction of the gravity requires functional PGM activity that is required for full development of amyloplasts. |
| 559 | 255540\_at | AT4G01800 |  | 1 | -0.9 | -0.1 | -0.1 | 0 | -0.2 | 0 | 0.1 | 0 | -0.3 | -0.5 | -0.4 | -1.7 | -1.2 | -0.6 | -2.1 | -1.6 | -1.9 | -1.2 | -0.3 | -0.1 | 0.1 | 0 | -0.2 | -0.1 | -0.1 | -0.9 | 0.1 | -0.1 | preprotein translocase secA subunit, putative; Identical to Preprotein translocase secA subunit, chloroplast precursor (SECA) [Arabidopsis Thaliana] (GB:Q9SYI0); similar to preprotein translocase secA family protein [Arabidopsis thaliana] (TAIR:AT1G21650.1); similar to preprotein translocase secA precursor - spinach (GB:A57386); similar to Preprotein translocase secA subunit, chloroplast precursor (GB:Q36795); similar to Preprotein translocase secA subunit, chloroplast precursor (GB:Q41062); contains InterPro domain SecA Wing and Scaffold; (InterPro:IPR011116); contains InterPro domain SecA preprotein cross-linking region; (InterPro:IPR011130); contains InterPro domain SecA protein; (InterPro:IPR000185); contains InterPro domain SecA DEAD-like; (InterPro:IPR011115) |
| 558 | 251120\_at | AT3G63490 |  | 1 | -0.4 | 0 | 0 | -0.1 | -0.3 | 0 | 0.2 | 0 | -0.4 | -1.1 | -0.1 | -1.7 | -1.3 | -0.9 | -2.5 | -1.9 | -1.3 | -1.3 | -0.1 | -0.2 | 0.1 | 0.1 | 0 | -0.1 | -0.1 | -0.6 | -0.1 | -0.1 | ribosomal protein L1 family protein; Identical to 50S ribosomal protein L1, chloroplast precursor (CL1) (RPL1) [Arabidopsis Thaliana] (GB:Q9LY66;GB:Q9LWB4); similar to ribosomal protein L1 family protein [Arabidopsis thaliana] (TAIR:AT2G42710.1); similar to 50S ribosomal protein L1, chloroplast precursor (CL1) (GB:Q9LE95); contains InterPro domain Ribosomal protein L1; (InterPro:IPR002143); contains InterPro domain Ribosomal protein L1, bacterial and chloroplast form; (InterPro:IPR005878), ribosomal protein L1 family protein; similar to ribosomal protein L1 family protein [Arabidopsis thaliana] (TAIR:AT2G42710.1); similar to 50S ribosomal protein L1, chloroplast precursor (CL1) (GB:Q9LE95); contains InterPro domain Ribosomal protein L1; (InterPro:IPR002143); contains InterPro domain Ribosomal protein L1, bacterial and chloroplast form; (InterPro:IPR005878) |
| 557 | 252441\_at | AT3G46780 | PTAC16 | 2 | -1 | -0.1 | 0 | -0.2 | -0.2 | -0.1 | 0 | -0.2 | -0.6 | -2.8 | -0.7 | -2.5 | -2.4 | -6.7 | -4.7 | -3.3 | -2.8 | -2.8 | -0.3 | -0.1 | 0.2 | 0 | -0.2 | -0.1 | -0.4 | -1 | 0 | -0.3 | PTAC16 (PLASTID TRANSCRIPTIONALLY ACTIVE18); similar to catalytic/ coenzyme binding [Arabidopsis thaliana] (TAIR:AT3G18890.1); similar to Os05g0291700 [Oryza sativa (japonica cultivar-group)] (GB:NP\_001055100.1); similar to Tic62 protein [Pisum sativum] (GB:CAC87810.2); contains domain NAD(P)-binding Rossmann-fold domains (SSF51735); contains domain no description (G3D.3.40.50.720) |
| 556 | 250243\_at | AT5G13630 | CCH, CCH1, CHLH, GUN5 | 15 | -1.2 | -0.1 | 0 | 0.1 | -0.2 | 0.1 | 0.1 | -0.1 | -0.3 | -1.5 | -0.6 | -1.1 | -1.1 | -3.8 | -3.2 | -3.3 | -2.4 | -1.1 | -0.2 | -0.2 | 0 | -0.1 | -0.1 | -0.2 | -0.5 | -1 | -0.1 | -0.2 | Encodes magnesium chelatase involved in plastid-to-nucleus signal transduction., Encodes magnesium chelatase involved in plastid-to-nucleus signal transduction., Encodes magnesium chelatase involved in plastid-to-nucleus signal transduction., Encodes magnesium chelatase involved in plastid-to-nucleus signal transduction. |
| 555 | 248377\_at | AT5G51720 |  | 1 | -2.2 | -1.5 | 0.6 | -0.2 | -0.3 | 0.2 | -0.2 | -0.8 | -2.4 | -4.3 | -0.7 | -2.3 | -2.5 | -4.9 | -4.2 | -4.9 | -6.6 | -1 | -0.6 | -1.7 | 0 | 0.3 | -0.4 | 0 | -1 | -1.5 | 0.1 | -0.5 | similar to zinc finger, CDGSH-type domain 2 [Homo sapiens] (GB:NP\_001008389.1); similar to Os07g0467200 [Oryza sativa (japonica cultivar-group)] (GB:NP\_001059590.1); similar to hypothetical protein [Homo sapiens] (GB:CAD97935.1); contains InterPro domain Zinc finger, CDGSH-type; (InterPro:IPR006622) |
| 554 | 245745\_at | AT1G51110 |  | 2 | -0.9 | -0.1 | 0.2 | -0.2 | 0.3 | 0.2 | -0.3 | -0.3 | -0.8 | -2 | -0.7 | -2.3 | -2 | -3.5 | -2.7 | -1.9 | -1.9 | -1.1 | -0.9 | -0.2 | -0.1 | 0.3 | 0 | 0.1 | -0.5 | -0.6 | -0.2 | 0 | plastid-lipid associated protein PAP / fibrillin family protein; similar to plastid-lipid associated protein PAP / fibrillin family protein [Arabidopsis thaliana] (TAIR:AT3G26070.1); similar to PAP fibrillin [Medicago truncatula] (GB:ABE81557.1); contains InterPro domain PAP fibrillin; (InterPro:IPR006843) |
| 553 | 245744\_at | AT1G51110 |  | 2 | -1.1 | -0.1 | 0 | -0.2 | 0 | 0.2 | 0 | -0.1 | -0.8 | -1.9 | -0.3 | -2.7 | -2 | -5.1 | -3 | -2.3 | -2 | -2.2 | -0.3 | 0.3 | 0.4 | 0.2 | -0.2 | 0 | -0.1 | -0.4 | 0 | 0 | plastid-lipid associated protein PAP / fibrillin family protein; similar to plastid-lipid associated protein PAP / fibrillin family protein [Arabidopsis thaliana] (TAIR:AT3G26070.1); similar to PAP fibrillin [Medicago truncatula] (GB:ABE81557.1); contains InterPro domain PAP fibrillin; (InterPro:IPR006843) |
| 552 | 258151\_at | AT3G18080 | BGLU44 | 1 | -0.4 | 0.1 | -0.7 | -0.3 | -0.1 | -0.2 | 0.1 | 0 | -0.4 | -2 | 0.2 | -0.4 | -1.1 | -2.6 | -1.9 | -1 | -1.1 | -0.6 | -0.5 | 0 | 0.3 | 0.2 | 0.1 | -0.2 | 0.1 | -0.6 | 0 | -0.6 | glycosyl hydrolase family 1 protein; similar to glycosyl hydrolase family 1 protein [Arabidopsis thaliana] (TAIR:AT3G18070.1); similar to beta-mannosidase enzyme [Lycopersicon esculentum] (GB:AAL37714.1); similar to beta-mannosidase 3 [Oncidium Gower Ramsey] (GB:ABC55716.1); similar to beta-mannosidase 2 [Oncidium Gower Ramsey] (GB:ABC55717.1); contains InterPro domain 6-phospho-beta-galactosidase, eukaryotic; (InterPro:IPR011580); contains InterPro domain Glycoside hydrolase, family 1; (InterPro:IPR001360) |
| 551 | 255826\_at | AT2G40490 | HEME2 | 3 | 0 | 0 | 0.2 | 0.3 | -0.1 | -0.2 | -0.1 | 0.1 | 0 | -2.8 | -0.4 | -1.7 | -1.4 | -2.3 | -2.9 | -1.2 | -1.2 | -1.3 | -0.3 | -0.4 | -0.7 | 0.1 | -0.1 | -0.1 | -0.2 | -0.9 | 0.1 | 0 | HEME2; uroporphyrinogen decarboxylase; Identical to Uroporphyrinogen decarboxylase, chloroplast precursor (EC 4.1.1.37) (URO-D) (UPD) [Arabidopsis Thaliana] (GB:O22886); similar to HEME1, uroporphyrinogen decarboxylase [Arabidopsis thaliana] (TAIR:AT3G14930.2); similar to Uroporphyrinogen decarboxylase, chloroplast precursor (URO-D) (UPD) (GB:Q42967); contains InterPro domain Uroporphyrinogen decarboxylase (URO-D); (InterPro:IPR000257); contains InterPro domain Uroporphyrinogen decarboxylase HemE; (InterPro:IPR006361) |
| 550 | 248285\_at | AT5G52960 |  |  | -0.3 | 0 | 0 | 0.1 | 0.2 | -0.5 | -0.1 | 0.1 | 0 | -2.5 | -0.3 | -1.7 | -1.5 | -2.3 | -2.1 | -1.7 | -1.4 | -1 | -0.5 | -0.6 | -0.8 | 0 | -0.2 | -0.4 | -0.5 | -1.2 | 0.1 | -0.3 | similar to hypothetical protein asr2994 [Nostoc sp. PCC 7120] (GB:NP\_487034.1); similar to Os05g0569200 [Oryza sativa (japonica cultivar-group)] (GB:NP\_001056361.1) |
| 549 | 257773\_at | AT3G29185 |  |  | 0.1 | -0.1 | 0.1 | -0.2 | 0 | -0.3 | -0.3 | -0.2 | -0.9 | -2.3 | -0.6 | -2 | -1.5 | -2.6 | -4.4 | -2.6 | -1.2 | -1.2 | -0.7 | -1 | -0.6 | 0 | -0.3 | -0.3 | -0.7 | -1.4 | -0.1 | -0.2 | similar to hypothetical protein MtrDRAFT\_AC147482g2v1 [Medicago truncatula] (GB:ABD32485.1) |
| 548 | 255447\_at | AT4G02790 |  |  | 0.5 | -0.1 | 0.3 | 0.1 | 0.1 | -0.5 | -0.2 | 0 | -0.4 | -2.5 | -0.1 | -1.9 | -1.3 | -1.4 | -5.8 | -2.1 | -1.3 | -1.5 | -0.4 | -0.5 | -0.5 | 0.1 | -0.1 | 0 | -0.6 | -1.3 | 0.1 | -0.1 | GTP-binding family protein; similar to GTP-binding family protein [Arabidopsis thaliana] (TAIR:AT2G41670.1); similar to Os05g0430400 [Oryza sativa (japonica cultivar-group)] (GB:NP\_001055624.1); similar to COG1161: Predicted GTPases [Nostoc punctiforme PCC 73102] (GB:ZP\_00109854.1); contains InterPro domain GTP-binding protein, HSR1-related; (InterPro:IPR002917); contains InterPro domain GTP1/OBG; (InterPro:IPR006073); contains InterPro domain GTP-binding; (InterPro:IPR005289) |
| 547 | 251461\_at |  |  |  | -0.1 | -0.2 | -0.1 | 0 | 0.2 | -0.3 | 0 | -0.3 | -0.3 | -1.9 | -0.5 | -1.7 | -1.5 | -1.5 | -3.6 | -2.2 | -0.8 | -0.7 | -0.7 | -0.6 | -0.4 | -0.1 | -0.2 | -0.2 | -0.2 | -0.6 | 0 | 0 |  |
| 546 | 246202\_at | AT4G37040 | MAP1D | 3 | -0.1 | -0.1 | 0.2 | 0 | 0.1 | -0.4 | -0.2 | -0.1 | -0.4 | -1.9 | -0.2 | -1.4 | -1 | -1 | -1.6 | -0.8 | -1.3 | -1 | -0.4 | -0.4 | -0.5 | 0.1 | -0.1 | -0.1 | -0.1 | -0.9 | 0.1 | 0 | encodes a methionine aminopeptidase |
| 545 | 259098\_at | AT3G04790 |  | 2 | -0.1 | -0.1 | 0.1 | -0.1 | 0 | -0.4 | -0.1 | -0.1 | 0.3 | -2 | -0.3 | -1.8 | -0.9 | -1 | -3.4 | -1.3 | -1.1 | -1 | -0.3 | -0.3 | -0.2 | 0.1 | -0.1 | -0.2 | -0.2 | -0.7 | 0.1 | -0.2 | ribose 5-phosphate isomerase-related; similar to ribose-5-phosphate isomerase [Arabidopsis thaliana] (TAIR:AT2G01290.1); similar to putative ribose-5-phosphate isomerase [Oryza sativa (japonica cultivar-group)] (GB:BAC83440.1); similar to Os07g0176900 [Oryza sativa (japonica cultivar-group)] (GB:NP\_001059023.1); contains InterPro domain Ribose 5-phosphate isomerase; (InterPro:IPR004788) |
| 544 | 257831\_at | AT3G26710 | CCB1 | 2 | -0.1 | -0.1 | 0.1 | -0.2 | 0.1 | -1 | 0 | -0.2 | -0.1 | -2.4 | -0.6 | -1.9 | -1.4 | -2.1 | -2.6 | -2.1 | -0.6 | -1.3 | -0.7 | -0.6 | -0.5 | 0.2 | -0.4 | -0.1 | -0.7 | -0.7 | -0.2 | -0.2 | similar to Os11g0526200 [Oryza sativa (japonica cultivar-group)] (GB:NP\_001067997.1); similar to unnamed protein product [Ostreococcus tauri] (GB:CAL53542.1) |
| 543 | 262693\_at | AT1G62780 |  | 1 | -0.7 | -0.1 | 0.2 | -0.1 | -0.2 | -0.6 | -0.3 | 0 | 0.1 | -2.8 | -0.3 | -2 | -1.9 | -1.8 | -3.8 | -1.5 | -1.3 | -2.2 | -0.3 | -0.3 | -0.7 | 0.1 | -0.2 | 0 | -0.4 | -1 | -0.1 | -0.1 | similar to B0616E02-H0507E05.5 [Oryza sativa (indica cultivar-group)] (GB:CAH67829.1) |
| 542 | 245118\_at | AT2G41680 | NTRC | 3 | -0.6 | -0.2 | 0 | 0.1 | -0.1 | -0.4 | -0.1 | 0.1 | 0.1 | -1.8 | -0.1 | -1.4 | -1.4 | -1.2 | -1.8 | -1.1 | -1.2 | -0.9 | -0.4 | -0.4 | -0.4 | 0 | -0.2 | -0.2 | -0.2 | -0.7 | -0.1 | 0 | thioredoxin reductase, putative / NADPH-dependent thioredoxin reductase, putative; similar to NTRA (NADPH-dependent thioredoxin reductase 2) [Arabidopsis thaliana] (TAIR:AT2G17420.1); similar to Os07g0657900 [Oryza sativa (japonica cultivar-group)] (GB:NP\_001060515.1); similar to thioredoxin reductase [Nostoc sp. PCC 7120] (GB:NP\_484780.1); contains InterPro domain Thioredoxin-like fold; (InterPro:IPR012336); contains InterPro domain Pyridine nucleotide-disulphide oxidoreductase, NAD-binding region; (InterPro:IPR001327); contains InterPro domain Adrenodoxin reductase; (InterPro:IPR000759); contains InterPro domain Thioredoxin-related; (InterPro:IPR006662); contains InterPro domain Thioredoxin domain 2; (InterPro:IPR006663); contains InterPro domain Glutaredoxin; (InterPro:IPR002109); contains InterPro domain Thioredoxin reductase; (InterPro:IPR005982); contains InterPro domain Thioredoxin fold; (InterPro:IPR012335); contains InterPro domain Pyridine nucleotide-disulphide oxidoreductase, class-II; (InterPro:IPR000103); contains InterPro domain FAD-dependent pyridine nucleotide-disulphide oxidoreductase; (InterPro:IPR013027) |
| 541 | 247252\_at | AT5G64770 |  |  | 0.7 | 0.1 | 2.1 | -0.4 | 0 | -1.5 | -2.2 | -0.2 | 0.3 | -3.5 | -0.7 | -2.7 | -1.8 | -1.5 | -4.6 | -3.5 | -1.7 | -1.5 | -0.7 | -0.1 | -0.5 | -0.5 | 1.8 | 0.3 | -1.5 | -1.5 | 1 | 0.1 | similar to 80C09\_10 [Brassica rapa subsp. pekinensis] (GB:AAZ41821.1) |
| 540 | 246004\_at | AT5G20630 | ATGER3, GER3, GLP3, GLP3A, GLP3B | 5 | 0 | -0.1 | -0.1 | 0.1 | 0.1 | -0.7 | 0.1 | -0.9 | 0.7 | -8.5 | -1.4 | -10.7 | -7 | -9.5 | -10.8 | -4.6 | -1 | -1.6 | -0.3 | -0.6 | -1 | -0.2 | -0.1 | -0.5 | 0.1 | -0.5 | 0.1 | 0.3 | Encodes a germin-like protein., Encodes a germin-like protein., Encodes a germin-like protein., Encodes a germin-like protein., Encodes a germin-like protein. |
| 539 | 245397\_at | AT4G14560 | AXR5, IAA1 | 18 | -0.5 | 0 | 0 | -1.2 | 0 | -1.5 | -0.1 | -0.5 | 1.5 | -1.9 | -1.1 | -2.1 | -1.8 | -1.2 | -2 | -1.8 | -1.6 | -1.2 | -0.6 | -0.4 | -0.9 | -0.4 | 4.2 | -1.2 | 0 | -0.7 | 0.2 | -0.3 | auxin (indole-3-acetic acid) induced gene (IAA1) encoding a short-lived nuclear-localized transcriptional regulator protein., auxin (indole-3-acetic acid) induced gene (IAA1) encoding a short-lived nuclear-localized transcriptional regulator protein. |
| 538 | 258764\_at | AT3G10720 |  | 1 | -0.6 | 0.7 | 1 | 0 | 0.5 | -0.6 | -0.1 | 0 | -1.8 | -3.1 | -0.3 | -4.7 | -3.3 | -6.7 | -9 | -4.6 | -1.6 | -0.8 | -0.4 | -0.3 | -0.4 | 0.5 | 1.9 | -1.3 | 2 | 0.2 | 0.4 | 1 | pectinesterase, putative; similar to pectinesterase, putative [Arabidopsis thaliana] (TAIR:AT5G04970.1); similar to papillar cell-specific pectin methylesterase-like protein [Brassica napus] (GB:AAK84428.1); similar to Os01g0311800 [Oryza sativa (japonica cultivar-group)] (GB:NP\_001042865.1); similar to putative pectinesterase [Oryza sativa (japonica cultivar-group)] (GB:AAT38097.1); contains InterPro domain Virulence factor, pectin lyase fold; (InterPro:IPR011050); contains InterPro domain Plant invertase/pectin methylesterase inhibitor; (InterPro:IPR007186); contains InterPro domain Pectinesterase; (InterPro:IPR000070), pectinesterase, putative; similar to pectinesterase, putative [Arabidopsis thaliana] (TAIR:AT5G04970.1); similar to pectin methylesterase isoform alpha [Vigna radiata] (GB:AAF35897.1); contains InterPro domain Virulence factor, pectin lyase fold; (InterPro:IPR011050); contains InterPro domain Pectinesterase; (InterPro:IPR000070) |
| 537 | 261141\_at | AT1G19740 |  |  | -0.1 | 0 | 0.3 | 0 | 0 | -0.4 | 0 | -0.3 | -0.4 | -0.7 | -0.2 | -0.9 | -0.6 | 0.2 | -1.2 | -1.2 | -3.7 | -1.7 | -0.1 | -0.4 | -0.4 | 0.4 | 0.1 | -0.2 | -0.5 | -0.9 | -0.1 | -0.3 | ATP-dependent protease La (LON) domain-containing protein; similar to ATP-dependent protease La (LON) domain-containing protein [Arabidopsis thaliana] (TAIR:AT1G75460.1); similar to Os07g0509700 [Oryza sativa (japonica cultivar-group)] (GB:NP\_001059749.1); similar to hypothetical protein all4335 [Nostoc sp. PCC 7120] (GB:NP\_488375.1); similar to COG2802: Uncharacterized protein, similar to the N-terminal domain of Lon protease [Nostoc punctiforme PCC 73102] (GB:ZP\_00107090.1); contains InterPro domain Peptidase S16, lon N-terminal; (InterPro:IPR003111) |
| 536 | 248283\_at | AT5G52920 | PKP-BETA1, PKP1, PKP2 | 4 | 0.6 | 0.2 | 0.3 | 0.2 | -0.1 | 0 | -0.1 | -0.4 | -0.4 | -0.9 | -0.9 | -2.4 | -1.5 | 0.2 | -3.3 | -0.8 | -0.8 | -0.9 | -0.5 | 0.1 | -0.7 | 0.2 | 0.2 | 0.1 | -0.1 | -0.4 | 0.1 | 0 | pyruvate kinase, putative; similar to pyruvate kinase, putative [Arabidopsis thaliana] (TAIR:AT1G32440.1); similar to Pyruvate kinase isozyme G, chloroplast precursor (GB:Q40546); similar to Pyruvate kinase [Medicago truncatula] (GB:ABE87102.1); similar to Os10g0571200 [Oryza sativa (japonica cultivar-group)] (GB:NP\_001065454.1); contains InterPro domain Pyruvate kinase, beta-barrel-like; (InterPro:IPR011037); contains InterPro domain Pyruvate kinase; (InterPro:IPR001697), pyruvate kinase, putative; similar to pyruvate kinase, putative [Arabidopsis thaliana] (TAIR:AT1G32440.1); similar to Pyruvate kinase isozyme G, chloroplast precursor (GB:Q40546); similar to Pyruvate kinase [Medicago truncatula] (GB:ABE87102.1); similar to Os10g0571200 [Oryza sativa (japonica cultivar-group)] (GB:NP\_001065454.1); contains InterPro domain Pyruvate kinase, beta-barrel-like; (InterPro:IPR011037); contains InterPro domain Pyruvate kinase; (InterPro:IPR001697), pyruvate kinase, putative; similar to pyruvate kinase, putative [Arabidopsis thaliana] (TAIR:AT1G32440.1); similar to Pyruvate kinase isozyme G, chloroplast precursor (GB:Q40546); similar to Pyruvate kinase [Medicago truncatula] (GB:ABE87102.1); similar to Os10g0571200 [Oryza sativa (japonica cultivar-group)] (GB:NP\_001065454.1); contains InterPro domain Pyruvate kinase, beta-barrel-like; (InterPro:IPR011037); contains InterPro domain Pyruvate kinase; (InterPro:IPR001697) |
| 535 | 263906\_at | AT2G36250 | ATFTSZ2-1, FTSZ2-1 | 10 | 0 | -0.3 | -0.3 | -0.2 | 0.2 | -0.1 | 0.1 | -0.3 | -0.7 | -1.6 | -0.4 | -1.9 | -1.6 | -0.1 | -2.7 | -1 | -1.1 | -0.9 | -0.6 | -0.4 | 0 | 0 | -0.1 | 0 | -0.1 | -0.7 | 0 | 0 | Encodes one of two FtsZ proteins, tubulin-like proteins, in Arabidopsis. It is involved in chloroplast division., Encodes one of two FtsZ proteins, tubulin-like proteins, in Arabidopsis. It is involved in chloroplast division. |
| 534 | 261536\_at | AT1G01790 | ATKEA1, KEA1 | 2 | -0.6 | -0.2 | 0 | -0.2 | -0.1 | 0 | 0.2 | -0.3 | -1 | -2 | -0.3 | -2.6 | -2 | 0 | -3 | -1.9 | -1.9 | -1.2 | -0.6 | -0.3 | -0.6 | 0.1 | -0.3 | -0.1 | -0.4 | -0.9 | -0.1 | -0.1 | K efflux antiporter KEA1, K efflux antiporter KEA1 |
| 533 | 255456\_at | AT4G02920 |  |  | -0.2 | -0.3 | -0.4 | 0.1 | -0.2 | -1.9 | 0 | 0 | -0.2 | -0.7 | -0.4 | -1.1 | -0.8 | -1.4 | -0.9 | -2.1 | -1.7 | -0.7 | -0.2 | 0 | -0.1 | -0.1 | -0.6 | -0.9 | -1.1 | -1.2 | -0.2 | -0.1 | similar to unknown protein [Arabidopsis thaliana] (TAIR:AT1G03340.1); similar to IMP dehydrogenase/GMP reductase [Medicago truncatula] (GB:ABE86673.1) |
| 532 | 245117\_at | AT2G41560 | ACA4 | 6 | -0.1 | -0.6 | -0.5 | -0.2 | -0.2 | -1.7 | 0.1 | -0.2 | -0.3 | -0.7 | -0.3 | -2.1 | -0.5 | -0.8 | -3.4 | -1.2 | -1.4 | -1.3 | 0.3 | 0.2 | -0.1 | 0 | -0.2 | -0.8 | -0.6 | -1.6 | -0.2 | 0.1 | encodes a calmodulin-regulated Ca(2+)-ATPase that improves salt tolerance in yeast. localized to the vacuole. |
| 531 | 253160\_at | AT4G35760 |  | 2 | 0.2 | 0 | -0.1 | -0.1 | 0.1 | -0.7 | 0.2 | 0 | -0.1 | -0.5 | -0.1 | -1.5 | -0.9 | -0.6 | -2.3 | -1.2 | -1.3 | -2.1 | -0.1 | 0.1 | -0.4 | 0.1 | 0 | -0.2 | -0.4 | -1.1 | -0.1 | 0 | electron carrier/ protein disulfide oxidoreductase; similar to unnamed protein product [Ostreococcus tauri] (GB:CAL55978.1); similar to Os03g0131500 [Oryza sativa (japonica cultivar-group)] (GB:NP\_001048864.1); contains InterPro domain Thioredoxin-related; (InterPro:IPR006662); contains InterPro domain Vitamin K epoxide reductase; (InterPro:IPR012932); contains InterPro domain Thioredoxin fold; (InterPro:IPR012335) |
| 530 | 250366\_at | AT5G11420 |  |  | 1 | 0 | -0.7 | -0.2 | -0.2 | -0.6 | 0 | -0.2 | -1.2 | -3 | -0.8 | -3.4 | -2.9 | -4.4 | -6.8 | -4.2 | -1.5 | -1.5 | -0.4 | -0.2 | -0.5 | -0.6 | -0.3 | -1.5 | -2.7 | -3 | -1.1 | -0.1 | similar to unknown protein [Arabidopsis thaliana] (TAIR:AT5G25460.1); similar to unknown [Ricinus communis] (GB:CAB02653.1); contains InterPro domain Galactose-binding like; (InterPro:IPR008979); contains InterPro domain Protein of unknown function DUF642; (InterPro:IPR006946) |
| 529 | 264014\_at | AT2G21210 |  | 1 | 0.9 | 0 | -0.7 | 0.2 | 0 | -2.5 | -0.1 | -0.7 | 0.1 | -1.7 | -1.6 | -2.2 | -1.8 | -3.1 | -3.2 | -5.1 | -2.4 | -1.5 | -0.3 | -0.4 | -0.6 | 0.1 | -0.2 | -0.2 | -0.4 | -1.4 | -0.2 | -0.6 | Putative auxin-regulated protein whose expression is downregulated in response to chitin oligomers. |
| 528 | 247218\_at | AT5G65010 | ASN2 | 4 | 0.2 | -0.2 | -0.9 | -0.1 | -0.1 | -1.7 | 0 | -0.4 | -0.1 | -0.9 | -1.2 | -2.1 | -1.3 | -4 | -2.2 | -1.5 | -1.3 | -1.4 | -0.2 | 0.1 | 0.1 | 0.3 | -0.3 | -0.7 | -0.6 | -0.8 | -0.1 | -0.2 | Encodes asparagine synthetase (ASN2). |
| 527 | 263737\_at | AT1G60010 |  |  | -0.4 | -0.1 | -0.1 | 0.1 | 0.1 | -2 | 0.2 | -0.3 | -0.7 | -2.2 | -0.3 | -1.8 | -1.4 | -0.4 | -1.8 | -2.2 | -1.8 | -1.9 | -0.2 | 0.1 | -0.1 | 0.1 | 1.2 | 0.5 | -0.1 | -1.1 | 0 | -0.3 | similar to unknown protein [Arabidopsis thaliana] (TAIR:AT1G10530.1); similar to T10O24.15, related [Lycopersicon esculentum] (GB:AAX95759.1) |
| 526 | 247450\_at | AT5G62350 |  | 1 | 0.5 | -0.1 | -0.5 | 0.1 | -0.1 | -1.1 | 0 | -0.1 | -0.8 | -1.7 | -0.5 | -0.6 | -0.7 | -1.5 | -2.7 | -1.9 | -0.8 | -0.5 | 0 | -0.3 | -0.3 | -0.2 | 0 | 0.7 | -0.8 | -0.9 | -0.8 | -0.8 | invertase/pectin methylesterase inhibitor family protein / DC 1.2 homolog (FL5-2I22); similar to invertase/pectin methylesterase inhibitor family protein [Arabidopsis thaliana] (TAIR:AT3G47380.1); similar to putative ripening-related protein [Vitis vinifera] (GB:CAB85625.1); contains InterPro domain Plant invertase/pectin methylesterase inhibitor; (InterPro:IPR007186); contains InterPro domain Pectinesterase inhibitor; (InterPro:IPR006501) |
| 525 | 266551\_at | AT2G35260 |  |  | -0.4 | -0.2 | -0.6 | -0.1 | -0.1 | -1.6 | 0.2 | -0.2 | -0.5 | -1.2 | -0.6 | -1.4 | -1.2 | -4.4 | -3.1 | -3.2 | -1.7 | -2.5 | -0.3 | 0 | 0 | -0.4 | -0.7 | -0.5 | -0.9 | -1.7 | -0.4 | -0.3 | similar to unknown protein [Arabidopsis thaliana] (TAIR:AT4G17840.1); similar to hypothetical protein 40.t00061 [Brassica oleracea] (GB:ABD65174.1) |
| 524 | 245123\_at | AT2G47450 | CAO, CPSRP43 | 7 | -0.2 | -0.1 | -0.1 | 0.1 | -0.1 | -1.2 | -0.1 | -0.3 | -0.4 | -0.7 | -0.5 | -1.7 | -1.4 | -1.2 | -3 | -2.3 | -1 | -2.2 | -0.2 | 0.2 | 0.6 | 0 | -0.3 | -0.2 | -0.6 | -1 | -0.2 | -0.1 | A component of the chloroplast signal recognition particle pathway that is involved in LHCP targeting. Downregulated in response to high light., A component of the chloroplast signal recognition particle pathway that is involved in LHCP targeting. Downregulated in response to high light. |
| 523 | 253040\_at | AT4G37800 |  |  | -0.5 | -0.2 | 0.1 | 0.1 | 0 | 0.1 | 0.2 | -0.6 | -2.2 | -4.2 | -1.4 | -1.9 | -2.8 | -11 | -8.7 | -6.8 | -1.6 | -1.8 | -0.6 | 0.4 | 0.5 | 0 | -0.8 | -0.7 | -1.7 | -2.4 | -0.1 | -0.4 | xyloglucan:xyloglucosyl transferase, putative / xyloglucan endotransglycosylase, putative / endo-xyloglucan transferase, putative; Identical to Probable xyloglucan endotransglucosylase/hydrolase protein 7 precursor (EC 2.4.1.207) (At-XTH7) (XTH-7) (XTH7) [Arabidopsis Thaliana] (GB:Q8LER3;GB:Q9T067); similar to xyloglucan:xyloglucosyl transferase, putative / xyloglucan endotransglycosylase, putative / endo-xyloglucan transferase, putative [Arabidopsis thaliana] (TAIR:AT5G65730.1); similar to endo-xyloglucan transferase [Gossypium hirsutum] (GB:BAA21107.1); contains InterPro domain Beta-glucanase; (InterPro:IPR008264); contains InterPro domain Glycoside hydrolase, family 16, active site; (InterPro:IPR008263); contains InterPro domain Concanavalin A-like lectin/glucanase, subgroup; (InterPro:IPR013320); contains InterPro domain Concanavalin A-like lectin/glucanase; (InterPro:IPR008985); contains InterPro domain Glycoside hydrolase, family 16; (InterPro:IPR000757); contains InterPro domain Xyloglucan endo-transglycosylase, C-terminal; (InterPro:IPR010713) |
| 522 | 252971\_at | AT4G38770 | ATPRP4, PRP4 | 2 | -0.3 | 0 | 0 | -0.1 | -0.2 | -0.2 | 0 | -0.4 | -0.8 | -3 | -0.8 | -3.3 | -2.7 | -5.1 | -4.7 | -2.3 | -2.1 | -0.6 | -0.5 | 0 | 0 | -0.3 | -0.4 | -0.4 | -0.9 | -1.5 | -0.2 | -0.1 | Encodes one of four proline-rich proteins in Arabidopsis which are predicted to localize to the cell wall. Transcripts are most abundant in aerial organs of the plant., Encodes one of four proline-rich proteins in Arabidopsis which are predicted to localize to the cell wall. Transcripts are most abundant in aerial organs of the plant. |
| 521 | 254553\_at | AT4G19530 |  |  | -1.9 | -0.2 | -0.2 | 0.5 | -0.2 | -1.7 | -0.3 | -0.4 | -0.9 | -1.7 | -0.8 | -2.3 | -2.6 | -2.7 | -4.4 | -2.5 | -1.6 | -2.7 | 0.2 | -0.1 | -0.1 | -0.4 | -1.3 | -1.5 | -2.1 | -2.8 | -1.3 | -0.4 | disease resistance protein (TIR-NBS-LRR class), putative; similar to disease resistance protein (TIR-NBS-LRR class), putative [Arabidopsis thaliana] (TAIR:AT3G51570.1); similar to RPS4 (RESISTANT TO P. SYRINGAE 4) [Arabidopsis thaliana] (TAIR:AT5G45250.1); similar to disease resistance protein (TIR-NBS-LRR class), putative [Arabidopsis thaliana] (TAIR:AT5G45060.1); similar to TIR-NBS-LRR type disease resistance protein [Populus trichocarpa] (GB:ABF81419.1); contains InterPro domain NB-ARC; (InterPro:IPR002182); contains InterPro domain Disease resistance protein; (InterPro:IPR000767); contains InterPro domain Toll-Interleukin receptor; (InterPro:IPR000157); contains InterPro domain Leucine-rich repeat; (InterPro:IPR001611); contains InterPro domain Leucine-rich repeat 3; (InterPro:IPR011713) |
| 520 | 249008\_at | AT5G44680 |  |  | -0.9 | 0.5 | 1.7 | 1.2 | 0.1 | -2.5 | -1 | -0.6 | -0.8 | -3.8 | -1.3 | -4.9 | -3.4 | -5.1 | -7.1 | -4.2 | -2.7 | -4.3 | -0.3 | -0.2 | -0.5 | -1.2 | -1.3 | -1.7 | -2.3 | -3.5 | -1.9 | -0.9 | methyladenine glycosylase family protein; similar to methyladenine glycosylase family protein [Arabidopsis thaliana] (TAIR:AT3G12710.1); similar to Methyladenine glycosylase [Medicago truncatula] (GB:ABE81339.1); contains InterPro domain Methyladenine glycosylase; (InterPro:IPR005019); contains InterPro domain DNA glycosylase; (InterPro:IPR011257) |
| 519 | 258156\_at | AT3G18050 |  | 1 | -0.3 | -0.2 | -0.6 | 0.3 | 0 | -1.7 | 0 | -0.4 | -1 | -5 | -1 | -3.2 | -2.7 | -5.5 | -8.6 | -4.5 | -5.5 | -2 | -0.2 | -0.1 | 0.7 | -0.7 | -0.6 | -1.3 | -1.3 | -2.5 | -0.4 | -0.3 | similar to unknown protein [Arabidopsis thaliana] (TAIR:AT4G28100.1); similar to hypothetical protein MtrDRAFT\_AC139526g8v1 [Medicago truncatula] (GB:ABE80139.1) |
| 518 | 246487\_at | AT5G16030 |  |  | -0.3 | 0.2 | -0.2 | 0.3 | -0.1 | -1.4 | -0.1 | -0.5 | -1.2 | -3.8 | -1.1 | -3.5 | -2.2 | -6.3 | -9.2 | -6.4 | -4.7 | -3 | 0 | 0.1 | -0.1 | -0.6 | -1 | -2.8 | -2.3 | -3.2 | -1.3 | -1.1 | similar to unknown protein [Arabidopsis thaliana] (TAIR:AT3G02500.1); similar to hypothetical protein [Cleome spinosa] (GB:ABD96956.1) |
| 517 | 245318\_at | AT4G16980 |  |  | -0.2 | -0.2 | 0.1 | 0.1 | 0.1 | -0.2 | -0.2 | -0.2 | -0.5 | -4 | -0.7 | -1.6 | -1 | -4.8 | -5 | -3.2 | -4.2 | -0.5 | 0 | 0 | -0.1 | -0.4 | -0.6 | -0.7 | -1 | -2 | -0.5 | -0.1 | arabinogalactan-protein family; similar to AGP19/ATAGP19 (ARABINOGALACTAN-PROTEIN 19) [Arabidopsis thaliana] (TAIR:AT1G68725.1); similar to hypothetical protein [Capsella rubella] (GB:CAC82613.1); contains InterPro domain Glutelin; (InterPro:IPR000480) |
| 516 | 253650\_at | AT4G30020 |  |  | 0.6 | -0.5 | 0.4 | -0.5 | -0.5 | -1.8 | -0.5 | -0.7 | -1.4 | -3.1 | -1.1 | -3.4 | -3 | -4.8 | -7 | -4.5 | -1.5 | -1.2 | -0.7 | 0.8 | -0.1 | 0.1 | -0.1 | -1.3 | -1.4 | -2.2 | 0 | -0.3 | subtilase family protein; similar to subtilase family protein [Arabidopsis thaliana] (TAIR:AT5G44530.1); similar to subtilase family protein [Arabidopsis thaliana] (TAIR:AT4G20430.1); similar to SLP3 (Subtilisin-like serine protease 3), subtilase [Arabidopsis thaliana] (TAIR:AT2G19170.1); similar to proteinase TMP [Lycopersicon esculentum] (GB:AAB38743.1); similar to meiotic serine proteinase [Lycopersicon esculentum] (GB:AAF13299.1); similar to subtilisin-like protease [Arachis hypogaea] (GB:AAY54007.1); contains InterPro domain Protease-associated PA; (InterPro:IPR003137); contains InterPro domain Peptidase S8 and S53, subtilisin, kexin, sedolisin; (InterPro:IPR000209); contains InterPro domain Proteinase inhibitor I9, subtilisin propeptide; (InterPro:IPR010259); contains InterPro domain Protein of unknown function DUF1034; (InterPro:IPR010435); contains InterPro domain Proteinase inhibitor, propeptide; (InterPro:IPR009020) |
| 515 | 247377\_at | AT5G63180 |  |  | 0.5 | -0.1 | 0.2 | 0 | 0 | -5.3 | 0.2 | -0.4 | -0.9 | -9.4 | -0.6 | -5.7 | -3.1 | -10.6 | -9.8 | -5.9 | -3.8 | -1 | -0.4 | 0.7 | 0 | -1.6 | -1.4 | -0.8 | -1.5 | -3.8 | -0.3 | 0.1 | pectate lyase family protein; Identical to Probable pectate lyase 22 precursor (EC 4.2.2.2) [Arabidopsis Thaliana] (GB:Q93Z25;GB:Q9FMK5); similar to pectate lyase family protein [Arabidopsis thaliana] (TAIR:AT4G24780.1); similar to pectate lyase family protein [Arabidopsis thaliana] (TAIR:AT1G67750.1); similar to pectate lyase [Gossypium hirsutum] (GB:AAY85180.1); similar to pectate lyase [Malus x domestica] (GB:AAQ84042.1); similar to Pectate lyase [Prunus mume] (GB:BAE48664.1); contains InterPro domain Virulence factor, pectin lyase fold; (InterPro:IPR011050); contains InterPro domain Pectate lyase/Amb allergen; (InterPro:IPR002022); contains InterPro domain Pectolytic enzyme, Pectin lyase fold; (InterPro:IPR012334) |
| 514 | 266500\_at | AT2G06925 | ATSPLA2-ALPHA, PLA2-ALPHA | 2 | 0 | -0.1 | 0.3 | -0.1 | 0.1 | -1.1 | -0.2 | -0.5 | -0.6 | -1.1 | -0.8 | -2.9 | -1.9 | -2.4 | -4.3 | -0.7 | -1.3 | -0.6 | 1.1 | 0.1 | 1.2 | -0.1 | -0.3 | 0 | -0.4 | -0.8 | -0.2 | -0.3 | Encodes a secretory phospholipase A2 enzyme, which specifically hydrolyzes the sn-2 position of phospholipids. The enzyme has a preference towards linoleoyl acyl chain over palmitoyl acyl chain. It also has a slight preference for phosphatidylcholine over phosphatidylethanolamine., Encodes a secretory phospholipase A2 enzyme, which specifically hydrolyzes the sn-2 position of phospholipids. The enzyme has a preference towards linoleoyl acyl chain over palmitoyl acyl chain. It also has a slight preference for phosphatidylcholine over phosphatidylethanolamine. |
| 513 | 253624\_at | AT4G30580 | ATS2, EMB1995 | 5 | -0.1 | -0.2 | -0.1 | 0 | -0.1 | -0.6 | 0 | -0.1 | -0.4 | -1.1 | -0.5 | -1.5 | -1.1 | -1.5 | -1.9 | -1.2 | -1.4 | -1.1 | 0.2 | 0 | 0.3 | -0.2 | -0.3 | -0.2 | -0.4 | -0.7 | -0.1 | -0.4 | Encodes a plastidic lysophosphatidic acid acyltransferase (LPAAT). Is critical for chloroplasts phosphatidic acid biosynthesis. The null allele is embryo lethal., Encodes a plastidic lysophosphatidic acid acyltransferase (LPAAT). Is critical for chloroplasts phosphatidic acid biosynthesis. The null allele is embryo lethal. |
| 512 | 246011\_at | AT5G08330 |  | 1 | -1.4 | 0.4 | -0.7 | 0.3 | 0.3 | -2.2 | -0.1 | -0.2 | -0.9 | -4.2 | -0.6 | -2.2 | -2.7 | -4.9 | -6.6 | -3.8 | -2.8 | -2 | 0.2 | 0.5 | 0.4 | -1.3 | -0.8 | -0.9 | -1.5 | -1.5 | -0.9 | 0.1 | TCP family transcription factor, putative; similar to TCP family transcription factor, putative [Arabidopsis thaliana] (TAIR:AT5G23280.1); similar to unknown [Brassica rapa] (GB:ABB97033.1); contains InterPro domain TCP transcription factor; (InterPro:IPR005333) |
| 511 | 245845\_at | AT1G26150 |  |  | -0.8 | -0.1 | -0.3 | 0.3 | 0 | -1.2 | 0 | 0 | -0.6 | -1.7 | -0.3 | -1.6 | -1.4 | -1.3 | -3.2 | -2.4 | -1.6 | -1.4 | -0.1 | 0.4 | 0 | -0.5 | -0.6 | -0.1 | -0.8 | -2 | 0.1 | -0.2 | protein kinase; similar to protein kinase family protein [Arabidopsis thaliana] (TAIR:AT5G38560.1); similar to protein kinase family protein [Musa balbisiana] (GB:ABF70139.1); similar to Os01g0110500 [Oryza sativa (japonica cultivar-group)] (GB:NP\_001041798.1); similar to protein kinase family protein [Musa acuminata] (GB:ABF70054.1); contains InterPro domain Serine/threonine protein kinase; (InterPro:IPR002290); contains InterPro domain Protein kinase-like; (InterPro:IPR011009); contains InterPro domain Protein kinase; (InterPro:IPR000719); contains InterPro domain Serine/threonine protein kinase, active site; (InterPro:IPR008271) |
| 510 | 261769\_at | AT1G76100 | PETE1 | 3 | -0.9 | 0.1 | -0.1 | 0.4 | -0.2 | -0.4 | -0.1 | 0 | -0.4 | -2.3 | -0.5 | -1.4 | -1.9 | -3.8 | -4.3 | -2.7 | -1.3 | -1.2 | -0.3 | -0.2 | -0.1 | -0.5 | -0.6 | -0.1 | -0.7 | -1 | -0.3 | -0.4 | plastocyanin; Identical to Plastocyanin minor isoform, chloroplast precursor (PETE) [Arabidopsis Thaliana] (GB:P11490;GB:P93656;GB:Q52K91;GB:Q9SGS3); similar to DRT112 (DNA-damage-repair/toleration protein 112), copper ion binding / electron carrier [Arabidopsis thaliana] (TAIR:AT1G20340.1); similar to plastocyanin (GB:AAA32834.1); contains InterPro domain Plastocyanin; (InterPro:IPR002387); contains InterPro domain Blue (type 1) copper protein; (InterPro:IPR001235); contains InterPro domain Cupredoxin; (InterPro:IPR008972); contains InterPro domain Blue (type 1) copper domain; (InterPro:IPR000923); contains InterPro domain Blue (type 1) copper subtype; (InterPro:IPR011572) |
| 509 | 263761\_at | AT2G21330 |  | 4 | -0.4 | -0.4 | -0.1 | 0.1 | -0.2 | -0.4 | 0 | 0.3 | -0.2 | -1.4 | -0.2 | -1.3 | -0.8 | -1.6 | -3.5 | -3.5 | -1.8 | -0.7 | 0 | 0.1 | 0 | -0.3 | -0.4 | -0.7 | -0.4 | -1.4 | -0.2 | -0.4 | fructose-bisphosphate aldolase, putative; similar to fructose-bisphosphate aldolase, putative [Arabidopsis thaliana] (TAIR:AT4G38970.1); similar to plastidic aldolase NPALDP1 [Nicotiana paniculata] (GB:BAA77604.1); similar to latex plastidic aldolase-like protein [Hevea brasiliensis] (GB:AAM46780.1); similar to plastidic aldolase [Nicotiana paniculata] (GB:BAA77603.1); contains InterPro domain Fructose-bisphosphate aldolase, class-I; (InterPro:IPR000741), fructose-bisphosphate aldolase; similar to fructose-bisphosphate aldolase, putative [Arabidopsis thaliana] (TAIR:AT4G38970.1); similar to plastidic aldolase NPALDP1 [Nicotiana paniculata] (GB:BAA77604.1); similar to latex plastidic aldolase-like protein [Hevea brasiliensis] (GB:AAM46780.1); similar to plastidic aldolase [Nicotiana paniculata] (GB:BAA77603.1); contains InterPro domain Fructose-bisphosphate aldolase, class-I; (InterPro:IPR000741), fructose-bisphosphate aldolase; similar to fructose-bisphosphate aldolase, putative [Arabidopsis thaliana] (TAIR:AT4G38970.2); similar to chloroplast latex aldolase-like protein [Manihot esculenta] (GB:AAV74407.1); contains InterPro domain Fructose-bisphosphate aldolase, class-I; (InterPro:IPR000741) |
| 508 | 253849\_at | AT4G28080 |  |  | -0.7 | -0.2 | -0.2 | 0.1 | -0.3 | -0.5 | 0.1 | 0 | 0.2 | -1.9 | -0.5 | -1.6 | -1.5 | -1.7 | -3.3 | -3 | -1.9 | -0.8 | 0 | -0.1 | 0.1 | -0.4 | -0.6 | -0.9 | -1.2 | -1.8 | -0.1 | -0.7 | binding; similar to binding [Arabidopsis thaliana] (TAIR:AT1G15290.1); similar to tetratricopeptide repeat (TPR)-containing protein [Arabidopsis thaliana] (TAIR:AT1G01320.1); similar to putative tetratricopeptide repeat(TPR)-containing protein [Oryza sativa (japonica cultivar-group)] (GB:BAC84544.1); similar to TPR repeat [Medicago truncatula] (GB:ABE77904.1); similar to H0811D08.1 [Oryza sativa (indica cultivar-group)] (GB:CAJ86110.1); contains InterPro domain Tetratricopeptide region; (InterPro:IPR013026); contains InterPro domain Tetratricopeptide TPR\_2; (InterPro:IPR013105); contains InterPro domain Tetratricopeptide-like helical; (InterPro:IPR011990) |
| 507 | 262970\_at | AT1G75690 |  | 1 | -1.2 | -0.1 | 0.1 | 0.4 | -0.1 | -0.2 | 0.1 | -0.2 | -0.8 | -2.8 | -0.7 | -2.5 | -2 | -4.5 | -4.4 | -4 | -2.9 | -1.4 | -0.1 | 0.5 | -0.2 | -0.1 | -0.3 | -0.4 | -0.2 | -1.1 | 0.1 | -0.2 | chaperone protein dnaJ-related; similar to Os03g0244000 [Oryza sativa (japonica cultivar-group)] (GB:NP\_001049532.1); contains domain Cysteine-rich domain of the chaperone protein DnaJ. (SSF57938); contains domain no description (G3D.2.10.230.10) |
| 506 | 247709\_at | AT5G59250 |  | 2 | -0.8 | -0.1 | 0 | 0.1 | -0.1 | 0 | 0.3 | -0.5 | -0.7 | -1.3 | -0.7 | -2 | -1.7 | -3.2 | -3.1 | -2.8 | -2 | -0.7 | -0.5 | 0.2 | -0.2 | 0.1 | -0.1 | -0.3 | -0.2 | -1 | 0.1 | 0 | sugar transporter family protein; Identical to D-xylose-proton symporter-like 3 [Arabidopsis Thaliana] (GB:Q0WWW9;GB:Q9FIF2); similar to sugar transporter family protein [Arabidopsis thaliana] (TAIR:AT5G17010.3); similar to putative sugar transporter [Nicotiana langsdorffii x Nicotiana sanderae] (GB:ABB17074.1); contains InterPro domain Sugar transporter; (InterPro:IPR003663); contains InterPro domain Sugar transporter superfamily; (InterPro:IPR005829); contains InterPro domain Major facilitator superfamily; (InterPro:IPR007114); contains InterPro domain General substrate transporter; (InterPro:IPR005828) |
| 505 | 248828\_at | AT5G47110 |  | 2 | -0.5 | -0.1 | 0 | 0 | -0.1 | -0.2 | 0.1 | -0.3 | -0.4 | -0.5 | -0.5 | -1.7 | -1.2 | -0.9 | -1.8 | -2.3 | -1.3 | -1.3 | -0.2 | 0 | -0.2 | -0.1 | -0.1 | -0.3 | -0.2 | -0.8 | 0 | -0.2 | lil3 protein, putative; similar to LIL3:1, transcription factor [Arabidopsis thaliana] (TAIR:AT4G17600.1); similar to Lil3 protein, putative [Brassica oleracea] (GB:ABD64919.1) |
| 504 | 263114\_at | AT1G03130 | PSAD-2 | 1 | -0.6 | -0.1 | -0.1 | 0.5 | 0.1 | -0.2 | 0.2 | -0.1 | -0.3 | -1.1 | -0.4 | -0.8 | -0.7 | -2.8 | -2.5 | -2.1 | -1.6 | -0.8 | -0.1 | 0.1 | -0.1 | -0.1 | -0.2 | -0.3 | -0.4 | -1.2 | 0.1 | -0.2 | Encodes a protein predicted by sequence similarity with spinach PsaD to be photosystem I reaction center subunit II (PsaD2) |
| 503 | 258993\_at | AT3G08940 | LHCB4.2 | 5 | -0.5 | 0 | -0.3 | 0.1 | -0.1 | -0.1 | 0.1 | -0.1 | -0.3 | -0.8 | -0.6 | -0.7 | -0.6 | -3.8 | -2.7 | -2.2 | -1.8 | -0.6 | -0.1 | 0 | 0 | -0.1 | -0.2 | -0.4 | -0.4 | -1.1 | -0.1 | -0.2 | Lhcb4.2 protein (Lhcb4.2, protein involved in the light harvesting complex of photosystem II |
| 502 | 255572\_at | AT4G01050 |  | 2 | -0.7 | 0 | -0.2 | -0.1 | -0.1 | -0.2 | 0.1 | -0.1 | -0.3 | -1.5 | -0.5 | -1.9 | -1.3 | -2 | -2.8 | -2.4 | -1.7 | -0.5 | -0.1 | -0.1 | -0.1 | -0.1 | -0.2 | -0.2 | -0.3 | -0.8 | 0.1 | 0.2 | hydroxyproline-rich glycoprotein family protein, contains a rhodanese homology domain. |
| 501 | 258285\_at | AT3G16140 | PSAH-1 | 2 | -0.4 | 0 | 0 | -0.1 | -0.1 | 0 | 0 | -0.1 | -0.3 | -1 | -0.3 | -1.2 | -0.7 | -1.8 | -2.3 | -1.5 | -1.3 | -0.4 | 0 | 0 | -0.2 | -0.1 | -0.1 | -0.2 | -0.1 | -0.3 | 0 | -0.1 | Encodes subunit H of photosystem I reaction center subunit VI. |
| 500 | 253597\_at | AT4G30690 |  | 1 | -1.2 | 0.1 | 0.1 | 0 | -0.1 | 0 | 0.4 | -0.1 | -0.8 | -1.7 | -0.2 | -1.4 | -1.4 | -2.1 | -2.4 | -2.1 | -1.8 | -2.1 | 0.5 | -0.1 | 0 | 0 | 0 | -0.4 | -0.3 | -1.4 | 0.1 | 0.1 | translation initiation factor 3 (IF-3) family protein; similar to translation initiation factor 3 (IF-3) family protein [Arabidopsis thaliana] (TAIR:AT2G24060.1); similar to Initiation factor 3 [Medicago truncatula] (GB:ABE79490.1); contains InterPro domain Initiation factor 3; (InterPro:IPR001288) |
| 499 | 265287\_at | AT2G20260 | PSAE-2 | 4 | -0.4 | 0.1 | 0 | 0.2 | 0 | 0.1 | 0.3 | -0.1 | -0.3 | -1 | -0.2 | -1.7 | -1.2 | -1.8 | -2.5 | -2 | -1.4 | -1.2 | 0.2 | 0.2 | 0 | -0.1 | 0.1 | -0.1 | 0.1 | -0.3 | 0 | 0 | Encodes subunit E of photosystem I. |
| 498 | 247320\_at | AT5G64040 | PSAN | 9 | -0.6 | -0.2 | -0.2 | 0 | 0 | -0.1 | 0.1 | -0.2 | -0.5 | -1.5 | -0.4 | -1.2 | -1 | -3.1 | -3.3 | -2.8 | -2.1 | -0.7 | 0 | -0.1 | 0 | -0.2 | 0 | -0.2 | -0.1 | -0.4 | 0 | -0.1 | Encodes the only subunit of photosystem I located entirely in the thylakoid lumen. May be involved in the interaction between plastocyanin and the photosystem I complex. |
| 497 | 254790\_at | AT4G12800 | PSAL | 4 | -0.3 | 0 | -0.1 | 0 | -0.1 | -0.2 | 0 | -0.1 | -0.1 | -0.5 | -0.3 | -0.7 | -0.4 | -1.1 | -1.4 | -1.2 | -0.9 | -0.1 | -0.1 | 0 | 0 | -0.2 | 0 | -0.1 | 0 | -0.3 | 0 | 0 | Encodes subunit L of photosystem I reaction center. |
| 496 | 254737\_at | AT4G13840 |  |  | 1 | 0.2 | -0.1 | -0.2 | -0.2 | -0.7 | -0.1 | 0 | -0.9 | -0.7 | -0.2 | -3.6 | -3.1 | -1.8 | -6.3 | -2 | -2.3 | -1.7 | 0.2 | 0.6 | 0 | 1.2 | 0.6 | -1.1 | -0.5 | -1.4 | 0 | 0 | transferase family protein; similar to transferase family protein [Arabidopsis thaliana] (TAIR:AT3G23840.1); similar to Os04g0611200 [Oryza sativa (japonica cultivar-group)] (GB:NP\_001053836.1); similar to orf [Zea mays] (GB:CAA61258.1); contains InterPro domain Transferase; (InterPro:IPR003480) |
| 495 | 246550\_at | AT5G14920 |  |  | 0.4 | 0 | -0.1 | -0.2 | -0.1 | -0.9 | 0 | -0.2 | -1 | -2.4 | -0.9 | -2.4 | -2.5 | -2.9 | -4.7 | -1.5 | -1.9 | -1 | 0.4 | 0.1 | 0.7 | 0.6 | 0.3 | -0.4 | -0.3 | -1.4 | 0 | -0.2 | gibberellin-regulated family protein; similar to ATEPR1 (Arabidopsis thaliana extensin proline-rich 1) [Arabidopsis thaliana] (TAIR:AT2G27380.1); similar to hypothetical protein [Brassica napus var. napus] (GB:AAS68179.1); contains InterPro domain Pistil-specific extensin-like protein; (InterPro:IPR003882); contains InterPro domain Gibberellin regulated protein; (InterPro:IPR003854), structural constituent of cell wall; similar to ATEPR1 (Arabidopsis thaliana extensin proline-rich 1) [Arabidopsis thaliana] (TAIR:AT2G27380.1); similar to hypothetical protein [Brassica napus var. napus] (GB:AAS68179.1); contains InterPro domain Pistil-specific extensin-like protein; (InterPro:IPR003882) |
| 494 | 259839\_at | AT1G52190 |  |  | 0.5 | 0.8 | -4.1 | -1.1 | 0.2 | -4.7 | 1.2 | -0.6 | -1.2 | -4.3 | -1.2 | -6.5 | -3 | -3.1 | -8.3 | -3.5 | -6.6 | -2.8 | -0.5 | -0.2 | -0.4 | 0.3 | 1.2 | -4.9 | -0.6 | -2.6 | -0.7 | -1.1 | proton-dependent oligopeptide transport (POT) family protein; similar to proton-dependent oligopeptide transport (POT) family protein [Arabidopsis thaliana] (TAIR:AT3G16180.1); similar to TGF-beta receptor, type I/II extracellular re (GB:ABD32296.1); similar to TGF-beta receptor, type I/II extracellular re (GB:ABD32298.1); contains InterPro domain TGF-beta receptor, type I/II extracellular region; (InterPro:IPR000109) |
| 493 | 260070\_at | AT1G73830 | BEE3 | 3 | -0.7 | -0.3 | 1.3 | -1.9 | -0.2 | -3.6 | 0.6 | -0.8 | -0.8 | -2.6 | -1.8 | -3 | -1.2 | -4.3 | -3.2 | -2.8 | -2.8 | -4 | -0.2 | -0.4 | 0 | 0.7 | 0.4 | -3.1 | -1.6 | -0.6 | 0 | -0.7 | BEE3 (BR ENHANCED EXPRESSION 3); DNA binding / transcription factor; similar to BEE1 (BR ENHANCED EXPRESSION 1), transcription factor [Arabidopsis thaliana] (TAIR:AT1G18400.1); similar to putative bZIPtranscription factor protein [Brassica oleracea] (GB:AAW81732.1); contains InterPro domain Helix-loop-helix DNA-binding; (InterPro:IPR011598); contains InterPro domain Basic helix-loop-helix dimerisation region bHLH; (InterPro:IPR001092) |
| 492 | 257769\_at | AT3G23050 | AXR2, IAA7 | 38 | 0.4 | 0.2 | -0.8 | -0.2 | 0.1 | 0 | 0.2 | -0.1 | -0.6 | -1.2 | -0.7 | -2.9 | -1.7 | -3.3 | -1.5 | -2.7 | -1.8 | -1.6 | 0.1 | 0 | -0.3 | 0.6 | 1.5 | -0.5 | -0.1 | -1.1 | 0.1 | 0 | Transcription regulator acting as repressor of auxin-inducible gene expression. Plays role in the control of gravitropic growth and development in light-grown seedlings. Auxin induces the degradation of the protein in a dosage-dependent manner in a process mediated by AtRac1. Auxin induced the relocalization of the protein within the nucleus from a diffused nucleoplasmic pattern to a discrete particulated pattern named nuclear protein bodies or NPB in a process also mediated by Rac1. Colocalizes with SCF, CSN and 26S proteasome components., Transcription regulator acting as repressor of auxin-inducible gene expression. Plays role in the control of gravitropic growth and development in light-grown seedlings. Auxin induces the degradation of the protein in a dosage-dependent manner in a process mediated by AtRac1. Auxin induced the relocalization of the protein within the nucleus from a diffused nucleoplasmic pattern to a discrete particulated pattern named nuclear protein bodies or NPB in a process also mediated by Rac1. Colocalizes with SCF, CSN and 26S proteasome components. |
| 491 | 249894\_at | AT5G22580 |  | 1 | 0.3 | 0.4 | -1.2 | 0 | 0.1 | -0.6 | 1.2 | -1.8 | -1.2 | -7.2 | -2.3 | -5 | -2.6 | -6.4 | -6.4 | -3.7 | -3.3 | -1.1 | -0.1 | 0.2 | -0.7 | 0.6 | 0.5 | -0.4 | 0.4 | -0.8 | 0.6 | 1.3 | Identical to Protein At5g22580 [Arabidopsis Thaliana] (GB:Q9FK81); similar to stable protein 1-related [Arabidopsis thaliana] (TAIR:AT3G17210.1); similar to hypothetical protein [Cucumis melo] (GB:BAD93607.1); contains InterPro domain Stress responsive alpha-beta barrel; (InterPro:IPR013097); contains InterPro domain Dimeric alpha-beta barrel; (InterPro:IPR011008) |
| 490 | 252391\_at | AT3G47860 |  | 1 | -0.2 | -0.3 | -0.1 | -0.1 | -0.3 | -0.2 | -0.1 | -0.4 | -0.9 | -0.9 | -0.6 | -2.2 | -2.6 | -2.7 | -2.8 | -0.3 | -1.1 | -1.6 | -0.3 | -0.2 | -0.4 | -0.2 | -0.1 | -0.5 | -0.5 | -1.3 | 0 | -0.2 | apolipoprotein D-related; similar to chloroplast lipocalin [Glycine max] (GB:ABB02412.1); contains InterPro domain Lipocalin-related protein and Bos/Can/Equ allergen; (InterPro:IPR000566); contains InterPro domain Lipocalin; (InterPro:IPR002345); contains InterPro domain Calycin-like; (InterPro:IPR011038); contains InterPro domain Calycin; (InterPro:IPR012674) |
| 489 | 263656\_at | AT1G04240 | IAA3, SHY2 | 13 | 0.3 | 0.1 | 0.6 | -0.2 | 0.4 | 0.4 | -0.5 | -0.4 | -1 | -1.3 | -1.1 | -2.7 | -2.7 | -1.6 | -3.3 | -3 | -1.4 | -2.8 | -0.9 | -0.3 | -0.5 | -0.7 | 1.7 | -0.7 | -1 | -2.1 | -0.9 | -0.5 | SHY2/IAA3 regulates multiple auxin responses in roots. It is induced rapidly by IAA, and has been shown to be phosphorylated by oat phytochrome A in vitro., SHY2/IAA3 regulates multiple auxin responses in roots. It is induced rapidly by IAA, and has been shown to be phosphorylated by oat phytochrome A in vitro. |
| 488 | 259375\_at | AT3G16370 |  |  | 0.5 | 0.3 | 0.4 | 0.2 | 0 | 0.2 | -0.4 | -0.3 | -1.7 | -1.6 | -0.9 | -4 | -3.5 | -6.8 | -9.4 | -5.7 | -2.2 | -1.3 | -0.7 | -0.3 | -0.8 | -0.9 | -0.3 | -0.6 | -1.2 | -1.7 | -0.4 | -0.3 | GDSL-motif lipase/hydrolase family protein; similar to GDSL-motif lipase, putative [Arabidopsis thaliana] (TAIR:AT5G22810.1); similar to Lipolytic enzyme, G-D-S-L [Medicago truncatula] (GB:ABE90349.1); contains InterPro domain Lipolytic enzyme, G-D-S-L; (InterPro:IPR001087) |
| 487 | 257974\_at | AT3G20820 |  | 2 | 0.9 | 0 | 0.3 | -0.3 | 0 | 0.8 | 0.1 | -0.7 | -1.7 | -0.7 | -0.9 | -2.9 | -3.1 | -3.5 | -2.9 | -3.6 | -1.8 | -1.5 | -0.4 | 0.1 | -0.6 | -0.1 | -0.2 | -1.5 | -1.8 | -2.2 | -1 | -1.2 | leucine-rich repeat family protein; similar to leucine-rich repeat family protein [Arabidopsis thaliana] (TAIR:AT5G12940.1); similar to polygalacturonase inhibitor-like protein [Cicer arietinum] (GB:CAD56505.1); contains InterPro domain Rudiment single hybrid motif; (InterPro:IPR011054); contains InterPro domain Leucine-rich repeat; (InterPro:IPR001611); contains InterPro domain Leucine-rich repeat, plant specific; (InterPro:IPR007090); contains InterPro domain Leucine rich repeat, N-terminal; (InterPro:IPR013210) |
| 486 | 262954\_at | AT1G54500 |  | 2 | -0.2 | -0.2 | 0.1 | 0.2 | 0.1 | 0.2 | 0 | -0.3 | -0.5 | -1.3 | -0.4 | -2 | -1.6 | -2 | -2.8 | -2.1 | -1.8 | -0.8 | -0.2 | -0.4 | -0.4 | -0.2 | -0.4 | -0.5 | -1 | -1.9 | -0.5 | -0.7 | rubredoxin family protein; similar to putative rubredoxin [Musa acuminata] (GB:ABI23729.1); similar to Os08g0323400 [Oryza sativa (japonica cultivar-group)] (GB:NP\_001061541.1); contains InterPro domain Rubredoxin; (InterPro:IPR001052); contains InterPro domain Rubredoxin-type Fe(Cys)4 protein; (InterPro:IPR004039) |
| 485 | 261488\_at | AT1G14345 |  | 1 | 0.1 | -0.1 | 0.4 | 0.4 | 0.2 | -0.7 | -0.2 | -0.4 | -0.7 | -2 | -0.7 | -2 | -2.3 | -3 | -3.2 | -5.4 | -2.5 | -0.9 | -0.6 | -0.6 | -0.4 | -0.4 | -0.8 | -0.9 | -1.7 | -2 | -0.5 | -0.8 | similar to hypothetical protein 9 [Ipomoea batatas] (GB:AAV88073.1); contains domain ALDO/KETO REDUCTASE (PTHR11732); contains domain ALDO/KETO REDUCTASE RELATED/NUCLEIC ACID BINDING (PTHR11732:SF1) |
| 484 | 267061\_at | AT2G32480 |  | 2 | 0.2 | 0 | 0.1 | 0.2 | 0.3 | -0.1 | -0.3 | -0.3 | -0.3 | -0.9 | -0.3 | -1.3 | -1 | -0.8 | -1.5 | -1.7 | -1.4 | -1 | -0.3 | -0.2 | -0.5 | 0.1 | -0.4 | -0.5 | -0.8 | -1.3 | -0.1 | -0.3 | membrane-associated zinc metalloprotease, putative; similar to membrane-associated zinc metalloprotease, putative [Arabidopsis thaliana] (TAIR:AT1G05140.1); similar to hypothetical protein slr1821 [Synechocystis sp. PCC 6803] (GB:NP\_441081.1); similar to Os03g0579000 [Oryza sativa (japonica cultivar-group)] (GB:NP\_001050547.1); contains InterPro domain Peptidase M50, putative membrane-associated zinc metallopeptidase; (InterPro:IPR004387); contains InterPro domain PDZ/DHR/GLGF; (InterPro:IPR001478); contains InterPro domain Peptidase M50; (InterPro:IPR008915) |
| 483 | 265400\_at | AT2G10940 |  | 2 | 0.3 | -0.2 | 0.1 | -0.1 | -0.1 | -0.7 | -0.2 | -1.5 | -2.2 | -8.6 | -2.2 | -5.2 | -3.4 | -9.2 | -8.5 | -7.5 | -4.4 | -0.9 | -0.4 | -0.6 | 0.8 | -0.4 | -0.2 | -0.4 | -0.5 | -2 | -0.2 | -0.1 | protease inhibitor/seed storage/lipid transfer protein (LTP) family protein; similar to unknown protein [Arabidopsis thaliana] (TAIR:AT3G22142.1); similar to hypothetical protein [Trifolium pratense] (GB:BAE71274.1); contains InterPro domain Plant lipid transfer/seed storage/trypsin-alpha amylase inhibitor; (InterPro:IPR003612) |
| 482 | 258054\_at | AT3G16240 | AQP1, ATTIP2;1, DELTA-TIP, DELTA-TIP1, TIP2;1 | 12 | 0.1 | 0 | 0.2 | 0.2 | 0.1 | 0 | 0 | -0.9 | -0.5 | -3.2 | -1.8 | -2.8 | -2.9 | -10 | -3.1 | -4.6 | -0.9 | -1.3 | -0.3 | -0.6 | -0.4 | -0.2 | -0.1 | -0.5 | -0.2 | -1.1 | 0 | 0 | Delta tonoplast intrinsic protein, functions as a water channel and ammonium (NH3) transporter. Highly expressed in flower, shoot, and stem. Expression shows diurnal regulation and is induced by ammonium (NH3). Protein localized to vacuolar membrane., Delta tonoplast intrinsic protein, functions as a water channel and ammonium (NH3) transporter. Highly expressed in flower, shoot, and stem. Expression shows diurnal regulation and is induced by ammonium (NH3). Protein localized to vacuolar membrane., Delta tonoplast intrinsic protein, functions as a water channel and ammonium (NH3) transporter. Highly expressed in flower, shoot, and stem. Expression shows diurnal regulation and is induced by ammonium (NH3). Protein localized to vacuolar membrane., Delta tonoplast intrinsic protein, functions as a water channel and ammonium (NH3) transporter. Highly expressed in flower, shoot, and stem. Expression shows diurnal regulation and is induced by ammonium (NH3). Protein localized to vacuolar membrane., Delta tonoplast intrinsic protein, functions as a water channel and ammonium (NH3) transporter. Highly expressed in flower, shoot, and stem. Expression shows diurnal regulation and is induced by ammonium (NH3). Protein localized to vacuolar membrane. |
| 481 | 264371\_at | AT1G12090 | ELP |  | -0.1 | -0.1 | 0 | 0 | 0 | 0 | -0.1 | -0.7 | -0.9 | -0.1 | -1.5 | -1.4 | -1.9 | -5.5 | -6.6 | -3.7 | -1.3 | -1 | -0.9 | -0.4 | 0 | -0.2 | -0.1 | -0.2 | -0.4 | -0.9 | -0.2 | -0.1 | extensin-like protein (ELP) |
| 480 | 261049\_at | AT1G01430 |  |  | 0.1 | 0.1 | 0 | 0.2 | -0.1 | -0.6 | 0.1 | -0.3 | -1 | -2.7 | -0.8 | -1.6 | -1.4 | -2.8 | -2.5 | -2 | -1.3 | -1.7 | -0.2 | -0.2 | -0.4 | -0.4 | -1.1 | 0 | -0.9 | -2.3 | -0.7 | -0.9 | similar to unknown protein [Arabidopsis thaliana] (TAIR:AT4G01080.1); similar to unknown protein Cr17 [Brassica napus] (GB:AAX51387.1); contains InterPro domain Protein of unknown function DUF231, plant; (InterPro:IPR004253) |
| 479 | 254239\_at | AT4G23400 | PIP1;5, PIP1D | 3 | -0.2 | 0.4 | 0.1 | 0.6 | 0.1 | -0.4 | 0.4 | -0.7 | -1.2 | -3.1 | -1.1 | -1.1 | -1.1 | -2.8 | -2.2 | -3 | -1 | -1.2 | -0.5 | -0.7 | -0.6 | -0.6 | -0.8 | -0.4 | -2.2 | -1.7 | -0.7 | -0.5 | PIP1;5/PIP1D (plasma membrane intrinsic protein 1;5); water channel; Identical to Probable aquaporin PIP1.5 (Plasma membrane intrinsic protein 1d) (PIP1d) (PIP1.5) [Arabidopsis Thaliana] (GB:Q8LAA6;GB:O81736); similar to TMP-C (plasma membrane intrinsic protein 1,4), water channel [Arabidopsis thaliana] (TAIR:AT4G00430.1); similar to PIP1C (PLASMA MEMBRANE INTRINSIC PROTEIN 1,3), water channel [Arabidopsis thaliana] (TAIR:AT1G01620.1); similar to plasma membrane aquaporin (PAQ1) [Raphanus sativus] (GB:BAA32777.1); contains InterPro domain Aquaporin; (InterPro:IPR012269); contains InterPro domain Major intrinsic protein; (InterPro:IPR000425), PIP1;5/PIP1D (plasma membrane intrinsic protein 1;5); water channel; Identical to Probable aquaporin PIP1.5 (Plasma membrane intrinsic protein 1d) (PIP1d) (PIP1.5) [Arabidopsis Thaliana] (GB:Q8LAA6;GB:O81736); similar to TMP-C (plasma membrane intrinsic protein 1,4), water channel [Arabidopsis thaliana] (TAIR:AT4G00430.1); similar to PIP1C (PLASMA MEMBRANE INTRINSIC PROTEIN 1,3), water channel [Arabidopsis thaliana] (TAIR:AT1G01620.1); similar to plasma membrane aquaporin (PAQ1) [Raphanus sativus] (GB:BAA32777.1); contains InterPro domain Aquaporin; (InterPro:IPR012269); contains InterPro domain Major intrinsic protein; (InterPro:IPR000425) |
| 478 | 255332\_at | AT4G04340 |  |  | 0.2 | 0.1 | -0.1 | 0.1 | -0.1 | -0.2 | 0.2 | -0.3 | -1 | -1.9 | -0.6 | -1.6 | -1.7 | -0.6 | -3.1 | -1.4 | -1.8 | -1.7 | -0.4 | -0.3 | -0.6 | -0.2 | -0.6 | -0.6 | -1.3 | -1.8 | -0.5 | -0.8 | early-responsive to dehydration protein-related / ERD protein-related; similar to early-responsive to dehydration protein-related / ERD protein-related [Arabidopsis thaliana] (TAIR:AT3G21620.1); similar to early-responsive to dehydration protein-related / ERD protein-related [Arabidopsis thaliana] (TAIR:AT1G62320.1); similar to early-responsive to dehydration protein-related / ERD protein-related [Arabidopsis thaliana] (TAIR:AT4G22120.2); similar to Protein of unknown function DUF221 [Medicago truncatula] (GB:ABE86757.1); similar to Os05g0594700 [Oryza sativa (japonica cultivar-group)] (GB:NP\_001056508.1); similar to Os01g0534900 [Oryza sativa (japonica cultivar-group)] (GB:NP\_001043256.1); contains InterPro domain Protein of unknown function DUF221; (InterPro:IPR003864) |
| 477 | 247162\_at | AT5G65730 |  |  | 0.1 | 0.2 | -0.3 | 1.3 | 0 | -1.1 | -0.4 | -0.8 | -4.1 | -9.4 | -1.1 | -2.4 | -4.4 | -8.7 | -3.6 | -3.3 | -5.9 | -3.5 | -0.9 | -0.6 | -0.1 | -3 | -4.2 | -2.4 | -5.3 | -2.8 | -2.6 | -2.4 | xyloglucan:xyloglucosyl transferase, putative / xyloglucan endotransglycosylase, putative / endo-xyloglucan transferase, putative; Identical to Probable xyloglucan endotransglucosylase/hydrolase protein 6 precursor (EC 2.4.1.207) (At-XTH6) (XTH-6) (XTH6) [Arabidopsis Thaliana] (GB:Q8LF99;GB:O49542;GB:Q93ZF8); similar to xyloglucan:xyloglucosyl transferase, putative / xyloglucan endotransglycosylase, putative / endo-xyloglucan transferase, putative [Arabidopsis thaliana] (TAIR:AT4G37800.1); similar to xyloglucan endotransglucosylase/hydrolase 2 [Rosa x borboniana] (GB:ABC55454.2); contains InterPro domain Beta-glucanase; (InterPro:IPR008264); contains InterPro domain Glycoside hydrolase, family 16, active site; (InterPro:IPR008263); contains InterPro domain Concanavalin A-like lectin/glucanase, subgroup; (InterPro:IPR013320); contains InterPro domain Concanavalin A-like lectin/glucanase; (InterPro:IPR008985); contains InterPro domain Glycoside hydrolase, family 16; (InterPro:IPR000757); contains InterPro domain Xyloglucan endo-transglycosylase, C-terminal; (InterPro:IPR010713) |
| 476 | 259786\_at | AT1G29660 |  |  | 0.7 | 0.4 | 1.5 | 0.8 | 0 | -0.8 | -2.4 | -0.3 | -0.3 | -6.2 | -1.1 | -3.2 | -3.6 | -5.8 | -10.8 | -3.3 | -2.7 | -2.1 | -0.2 | -0.2 | -0.2 | -3.2 | -1.1 | -1.3 | -2.5 | -3.4 | -2.8 | -0.5 | GDSL-motif lipase/hydrolase family protein; similar to GDSL-motif lipase/hydrolase family protein [Arabidopsis thaliana] (TAIR:AT1G29670.1); similar to GDSL-lipase 1 [Capsicum annuum] (GB:AAZ23955.1); contains InterPro domain Lipolytic enzyme, G-D-S-L; (InterPro:IPR001087) |
| 475 | 252711\_at | AT3G43720 |  | 2 | 0.3 | 0.4 | 1 | 0.3 | -0.1 | 0.1 | -0.7 | -0.6 | -1.4 | -1.4 | -1 | -2.8 | -3.3 | -6.5 | -6.3 | -2.2 | -1.8 | -1.8 | -0.5 | 0 | -0.4 | -1.4 | -0.5 | -0.2 | -0.5 | -1.4 | -0.8 | -0.4 | lipid binding; similar to protease inhibitor/seed storage/lipid transfer protein (LTP) family protein [Arabidopsis thaliana] (TAIR:AT2G27130.1); similar to Plant lipid transfer protein/Par allergen [Medicago truncatula] (GB:ABE89509.1); contains InterPro domain Plant lipid transfer/seed storage/trypsin-alpha amylase inhibitor; (InterPro:IPR003612), protease inhibitor/seed storage/lipid transfer protein (LTP) family protein; similar to protease inhibitor/seed storage/lipid transfer protein (LTP) family protein [Arabidopsis thaliana] (TAIR:AT2G27130.1); similar to Plant lipid transfer protein/Par allergen [Medicago truncatula] (GB:ABE89509.1); contains InterPro domain Plant lipid transfer/seed storage/trypsin-alpha amylase inhibitor; (InterPro:IPR003612) |
| 474 | 249037\_at | AT5G44130 | FLA13 | 3 | -0.3 | 0.2 | 0.3 | 0 | -0.1 | -1.4 | -0.3 | -0.7 | -1.4 | -4.7 | -1.4 | -1.8 | -1.2 | -3.8 | -4.6 | -4.3 | -2.1 | -3.1 | 0.5 | -0.1 | 0.6 | 0.5 | 0.3 | -1.5 | -1.7 | -1.6 | -1.1 | -1.2 | fasciclin-like arabinogalactan-protein, putative; Identical to Fasciclin-like arabinogalactan protein 13 precursor (FLA13) [Arabidopsis Thaliana] (GB:Q9FFH6); similar to FLA9 (FLA9) [Arabidopsis thaliana] (TAIR:AT1G03870.1); similar to unknown [Brassica rapa] (GB:ABK78690.1); contains InterPro domain Beta-Ig-H3/fasciclin; (InterPro:IPR000782) |
| 473 | 246371\_at | AT1G51940 |  |  | -0.1 | 0.1 | 0.9 | -0.1 | -0.2 | -2.6 | -0.2 | -0.1 | -0.9 | -4.2 | -0.7 | -1.5 | -2.4 | -2.4 | -6.1 | -2.2 | -1.8 | -2.4 | 0.3 | -0.8 | -0.1 | 0.2 | 0 | -0.3 | -1.8 | -2.3 | -1 | -0.9 | protein kinase family protein / peptidoglycan-binding LysM domain-containing protein; similar to protein kinase family protein [Arabidopsis thaliana] (TAIR:AT3G21630.1); similar to Os01g0741200 [Oryza sativa (japonica cultivar-group)] (GB:NP\_001044206.1); similar to protein kinase family protein [Musa balbisiana] (GB:ABF70119.1); contains InterPro domain Serine/threonine protein kinase; (InterPro:IPR002290); contains InterPro domain Protein kinase-like; (InterPro:IPR011009); contains InterPro domain Protein kinase; (InterPro:IPR000719); contains InterPro domain Tyrosine protein kinase; (InterPro:IPR001245); contains InterPro domain Serine/threonine protein kinase, active site; (InterPro:IPR008271); contains InterPro domain Peptidoglycan-binding LysM; (InterPro:IPR002482) |
| 472 | 254563\_at | AT4G19120 | ERD3 | 2 | 1.6 | 0.2 | 1.2 | -0.4 | -0.3 | -0.9 | -0.4 | -0.7 | -0.9 | -4 | -1 | -1.4 | -1.8 | -2.7 | -2.8 | -0.5 | -1.3 | -0.6 | 0.7 | 1.2 | 0.6 | 0.2 | 0.7 | -0.2 | -0.9 | -1.7 | -0.6 | -0.6 | ERD3 (EARLY-RESPONSIVE TO DEHYDRATION 3); similar to dehydration-responsive protein, putative [Arabidopsis thaliana] (TAIR:AT1G31850.2); similar to dehydration-responsive protein-like [Oryza sativa (japonica cultivar-group)] (GB:BAD67956.1); similar to Os06g0103900 [Oryza sativa (japonica cultivar-group)] (GB:NP\_001056546.1); contains InterPro domain Protein of unknown function DUF248, methyltransferase putative; (InterPro:IPR004159) |
| 471 | 264672\_at | AT1G09750 |  | 1 | 0.5 | -0.3 | 0.4 | 0.1 | -0.1 | -0.8 | -0.3 | -0.5 | -1.8 | -7 | -1 | -4.3 | -3.9 | -10.6 | -10.7 | -6.7 | -2.3 | -1.1 | 0 | -0.3 | -0.4 | -0.5 | -0.6 | -1.2 | -1.3 | -2.4 | -0.6 | -1 | chloroplast nucleoid DNA-binding protein-related; similar to aspartyl protease family protein [Arabidopsis thaliana] (TAIR:AT3G54400.1); similar to hypothetical protein [Trifolium pratense] (GB:BAE71208.1); contains InterPro domain Peptidase A1, pepsin; (InterPro:IPR001461); contains InterPro domain Peptidase aspartic, catalytic; (InterPro:IPR009007) |
| 470 | 251395\_at | AT2G45470 | AGP8, FLA8, AGP8, FLA8 | 6 | 0.3 | 0.2 | 0.5 | 0.2 | 0.1 | -0.7 | -0.1 | -0.7 | -1.1 | -5.5 | -0.8 | -3.2 | -4.2 | -4.9 | -6.9 | -2.5 | -1.3 | -1 | -1 | -0.6 | -0.1 | -0.3 | -0.3 | 0.2 | -1.4 | -2 | -0.8 | -0.9 | FLA8 (Arabinogalactan protein 8); Identical to Fasciclin-like arabinogalactan protein 8 precursor (AtAGP8) (FLA8) [Arabidopsis Thaliana] (GB:O22126;GB:Q56ZW9;GB:Q8L9B1;GB:Q9FR46); similar to FLA10 (fasciclin-like arabinogalactan-protein 10) [Arabidopsis thaliana] (TAIR:AT3G60900.1); similar to fasciclin-like protein FLA5 [Triticum aestivum] (GB:ABI95395.1); similar to Os04g0574200 [Oryza sativa (japonica cultivar-group)] (GB:NP\_001053617.1); contains InterPro domain Beta-Ig-H3/fasciclin; (InterPro:IPR000782), FLA8 (Arabinogalactan protein 8); Identical to Fasciclin-like arabinogalactan protein 8 precursor (AtAGP8) (FLA8) [Arabidopsis Thaliana] (GB:O22126;GB:Q56ZW9;GB:Q8L9B1;GB:Q9FR46); similar to FLA10 (fasciclin-like arabinogalactan-protein 10) [Arabidopsis thaliana] (TAIR:AT3G60900.1); similar to fasciclin-like protein FLA5 [Triticum aestivum] (GB:ABI95395.1); similar to Os04g0574200 [Oryza sativa (japonica cultivar-group)] (GB:NP\_001053617.1); contains InterPro domain Beta-Ig-H3/fasciclin; (InterPro:IPR000782) |
| 469 | 256516\_at | AT1G66150 | TMK1 | 5 | -0.2 | 0 | 0.3 | 0.2 | -0.1 | -0.3 | -0.1 | -0.4 | -1.1 | -2 | -0.8 | -1.1 | -2 | -2.9 | -1.5 | -1.7 | -1.3 | -1.6 | -0.4 | -0.4 | -0.3 | -0.3 | -0.2 | -0.7 | -0.9 | -1.5 | -0.4 | -0.4 | receptor-like transmembrane kinase I (TMK1) |
| 468 | 254221\_at | AT4G23820 |  |  | -0.4 | -0.1 | 0.2 | 0.1 | -0.2 | -0.7 | -0.1 | -0.4 | -1.9 | -8.9 | -1.1 | -3 | -3.7 | -8.6 | -8.1 | -4 | -6.3 | -2.2 | -0.8 | -0.6 | -0.2 | -0.3 | 0.1 | -0.5 | -1.2 | -2.1 | -0.6 | -0.3 | glycoside hydrolase family 28 protein / polygalacturonase (pectinase) family protein; similar to glycoside hydrolase family 28 protein / polygalacturonase (pectinase) family protein [Arabidopsis thaliana] (TAIR:AT5G41870.1); similar to Os05g0587000 [Oryza sativa (japonica cultivar-group)] (GB:NP\_001056466.1); similar to Os02g0256100 [Oryza sativa (japonica cultivar-group)] (GB:NP\_001046468.1); similar to putative polygalacturonase [Oryza sativa (japonica cultivar-group)] (GB:AAT44156.1); contains InterPro domain Virulence factor, pectin lyase fold; (InterPro:IPR011050); contains InterPro domain Glycoside hydrolase, family 28; (InterPro:IPR000743); contains InterPro domain Pectolytic enzyme, Pectin lyase fold; (InterPro:IPR012334) |
| 467 | 261230\_at | AT1G20010 | TUB5 | 2 | 0.4 | 0 | 0.4 | 0 | 0.1 | -1.3 | -0.4 | -0.3 | -1.1 | -7 | -1 | -3.2 | -3.1 | -3.6 | -7 | -4.4 | -2 | -2.4 | -0.6 | -0.1 | 0 | 0.3 | 0.2 | -0.6 | -1.2 | -1.6 | -0.5 | -0.6 | beta tubulin |
| 466 | 256237\_at | AT3G12610 | DRT100 | 4 | 0.5 | 0.4 | 0.5 | -0.2 | 0.1 | -1.4 | -0.6 | -0.6 | -2 | -4.1 | -2 | -4.4 | -4.4 | -8.4 | -9 | -5 | -1.5 | -3.1 | -1.3 | -0.3 | -1 | -0.2 | 0.4 | -1.4 | -1.7 | -3.1 | -0.8 | -0.6 | Plays role in DNA-damage repair/toleration. Partially complements RecA- phenotypes. |
| 465 | 246275\_at | AT4G36540 | BEE2, BEE2 | 3 | 1 | 0 | 0.4 | -0.4 | 0.1 | -2.8 | -0.1 | -0.3 | -0.7 | -9 | -0.9 | -4.7 | -3 | -5.4 | -8.1 | -5.5 | -2.5 | -2 | -0.4 | -0.4 | -1.1 | -0.3 | 0.4 | -1.4 | -2.3 | -2.5 | -1.1 | -0.5 | BEE2 (BR ENHANCED EXPRESSION 2); DNA binding / transcription factor; similar to basic helix-loop-helix (bHLH) family protein [Arabidopsis thaliana] (TAIR:AT2G18300.1); similar to Helix-loop-helix DNA-binding [Medicago truncatula] (GB:ABE85937.1); contains InterPro domain Helix-loop-helix DNA-binding; (InterPro:IPR011598); contains InterPro domain Basic helix-loop-helix dimerisation region bHLH; (InterPro:IPR001092), BEE2 (BR ENHANCED EXPRESSION 2); DNA binding / transcription factor; similar to basic helix-loop-helix (bHLH) family protein [Arabidopsis thaliana] (TAIR:AT2G18300.2); similar to Helix-loop-helix DNA-binding [Medicago truncatula] (GB:ABE85937.1); contains InterPro domain Helix-loop-helix DNA-binding; (InterPro:IPR011598); contains InterPro domain Basic helix-loop-helix dimerisation region bHLH; (InterPro:IPR001092) |
| 464 | 257137\_at | AT3G28860 | ABCB19, ATMDR1, ATMDR11, ATPGP19, MDR1, MDR11, PGP19 | 16 | 0.5 | 0.2 | -0.3 | -0.3 | -0.2 | -1.6 | 0 | -1.1 | -1.3 | -3.5 | -1.4 | -2.9 | -2.9 | -4.7 | -5.7 | -3.4 | -1.5 | -1.9 | -0.6 | -0.7 | -0.7 | -0.4 | 0.4 | -0.6 | -0.6 | -1 | -0.5 | -0.8 | Belongs to the family of ATP-binding cassette (ABC) transporters. Also known as AtMDR11 and PGP19. Possibly regulates auxin-dependent responses by influencing basipetal auxin transport in the root. Acts upstream of phyA in regulating hypocotyl elongation and gravitropic response. Exerts nonredundant, partially overlapping functions with the ABC transporter encoded by AtPGP1., Belongs to the family of ATP-binding cassette (ABC) transporters. Also known as AtMDR11 and PGP19. Possibly regulates auxin-dependent responses by influencing basipetal auxin transport in the root. Acts upstream of phyA in regulating hypocotyl elongation and gravitropic response. Exerts nonredundant, partially overlapping functions with the ABC transporter encoded by AtPGP1., Belongs to the family of ATP-binding cassette (ABC) transporters. Also known as AtMDR11 and PGP19. Possibly regulates auxin-dependent responses by influencing basipetal auxin transport in the root. Acts upstream of phyA in regulating hypocotyl elongation and gravitropic response. Exerts nonredundant, partially overlapping functions with the ABC transporter encoded by AtPGP1., Belongs to the family of ATP-binding cassette (ABC) transporters. Also known as AtMDR11 and PGP19. Possibly regulates auxin-dependent responses by influencing basipetal auxin transport in the root. Acts upstream of phyA in regulating hypocotyl elongation and gravitropic response. Exerts nonredundant, partially overlapping functions with the ABC transporter encoded by AtPGP1., Belongs to the family of ATP-binding cassette (ABC) transporters. Also known as AtMDR11 and PGP19. Possibly regulates auxin-dependent responses by influencing basipetal auxin transport in the root. Acts upstream of phyA in regulating hypocotyl elongation and gravitropic response. Exerts nonredundant, partially overlapping functions with the ABC transporter encoded by AtPGP1., Belongs to the family of ATP-binding cassette (ABC) transporters. Also known as AtMDR11 and PGP19. Possibly regulates auxin-dependent responses by influencing basipetal auxin transport in the root. Acts upstream of phyA in regulating hypocotyl elongation and gravitropic response. Exerts nonredundant, partially overlapping functions with the ABC transporter encoded by AtPGP1., Belongs to the family of ATP-binding cassette (ABC) transporters. Also known as AtMDR11 and PGP19. Possibly regulates auxin-dependent responses by influencing basipetal auxin transport in the root. Acts upstream of phyA in regulating hypocotyl elongation and gravitropic response. Exerts nonredundant, partially overlapping functions with the ABC transporter encoded by AtPGP1. |
| 463 | 259783\_at | AT1G29510 | SAUR68 | 1 | -0.4 | 0.2 | 2.3 | -1.2 | 0.5 | -1.6 | -1.3 | -1 | -1.4 | -5.1 | -2.4 | -5.7 | -4.2 | -7.4 | -6.7 | -5.4 | -3.1 | -1.6 | -1.3 | -0.7 | -0.5 | -0.4 | 2.7 | -0.2 | -2.2 | -3.4 | -0.9 | -1.9 | auxin-responsive protein, putative; similar to auxin-responsive protein, putative [Arabidopsis thaliana] (TAIR:AT1G29450.1); similar to auxin-induced SAUR-like protein [Capsicum annuum] (GB:AAM12781.1); contains InterPro domain Auxin responsive SAUR protein; (InterPro:IPR003676) |
| 462 | 248681\_at | AT5G48900 |  |  | 0.7 | 0.3 | 0.2 | -0.5 | -0.1 | 0.2 | -0.4 | -0.5 | -1.5 | -2.3 | -2.3 | -5.2 | -4.5 | -7.6 | -6.7 | -3.7 | -1.1 | -1.3 | -1.4 | -0.2 | -1 | -0.1 | 1 | -0.5 | -1.8 | -2.1 | -0.6 | -0.9 | pectate lyase family protein; Identical to Probable pectate lyase 20 precursor (EC 4.2.2.2) [Arabidopsis Thaliana] (GB:Q93WF1;GB:Q8LDB4;GB:Q9FI81); similar to pectate lyase family protein [Arabidopsis thaliana] (TAIR:AT3G24670.1); similar to pectate lyase family protein [Arabidopsis thaliana] (TAIR:AT4G13210.1); similar to pectate lyase family protein [Arabidopsis thaliana] (TAIR:AT3G07010.1); similar to pectate lyase [Fragaria x ananassa] (GB:AAK66161.1); similar to ripening-related pectate lyase [Man (GB:AAX88800.1); similar to pectate lyase B [Fragaria x ananassa] (GB:AAK66160.1); contains InterPro domain Virulence factor, pectin lyase fold; (InterPro:IPR011050); contains InterPro domain Pectate lyase/Amb allergen; (InterPro:IPR002022); contains InterPro domain Pectolytic enzyme, Pectin lyase fold; (InterPro:IPR012334) |
| 461 | 254609\_at | AT4G18970 |  |  | -0.1 | 0.3 | 1.5 | -0.4 | 0.2 | -0.1 | -1.3 | -0.7 | -1 | -6.7 | -2.7 | -7 | -5 | -7.1 | -7.7 | -4.2 | -5.4 | -0.8 | -1.4 | -1.4 | -0.6 | -0.2 | 0.4 | -1.6 | -1.4 | -2.9 | -1 | -0.6 | GDSL-motif lipase/hydrolase family protein; similar to GDSL-motif lipase/hydrolase family protein [Arabidopsis thaliana] (TAIR:AT5G45670.1); similar to GDSL-lipase protein [Capsicum annuum] (GB:AAX20033.1); similar to GDSL-lipase 1 [Capsicum annuum] (GB:AAZ23955.1); contains InterPro domain Lipolytic enzyme, G-D-S-L; (InterPro:IPR001087) |
| 460 | 261226\_at | AT1G20190 | ATEXP11, ATEXPA11, ATHEXP, EXP11 | 2 | 1.8 | -0.4 | 0 | -1 | 0 | -0.9 | -0.2 | -1 | -1.6 | -2.5 | -1.7 | -5.5 | -4.1 | -8 | -8.3 | -4.4 | -3.8 | -4.1 | -2.3 | -0.6 | -2.2 | -0.1 | 1.2 | -2.9 | -2.3 | -3.5 | -1.6 | -0.7 | member of Alpha-Expansin Gene Family. Naming convention from the Expansin Working Group (Kende et al, 2004. Plant Mol Bio), member of Alpha-Expansin Gene Family. Naming convention from the Expansin Working Group (Kende et al, 2004. Plant Mol Bio), member of Alpha-Expansin Gene Family. Naming convention from the Expansin Working Group (Kende et al, 2004. Plant Mol Bio), member of Alpha-Expansin Gene Family. Naming convention from the Expansin Working Group (Kende et al, 2004. Plant Mol Bio) |
| 459 | 251573\_at | AT3G58140 |  | 2 | 0.2 | 0.1 | 0.1 | 0 | 0.2 | 0 | 0.1 | 0 | 0 | -1.4 | -0.1 | -1.3 | -1 | -1.4 | -1.9 | -0.8 | -0.9 | -1 | -0.2 | -0.1 | 0.3 | 0.1 | 0.1 | 0 | -0.1 | -0.9 | 0 | 0 | phenylalanyl-tRNA synthetase class IIc family protein; similar to phenylalanyl-tRNA synthetase, putative / phenylalanine--tRNA ligase, putative [Arabidopsis thaliana] (TAIR:AT4G39280.1); similar to Os12g0533700 [Oryza sativa (japonica cultivar-group)] (GB:NP\_001066926.1); similar to phenylalanyl-tRNA synthetase class IIc family protein, putative, expressed [Oryza sativa (japonica cultivar-group)] (GB:ABA98813.1); contains InterPro domain Phenylalanyl-tRNA synthetase, class IIc; (InterPro:IPR002319); contains InterPro domain Phenylalanyl-tRNA synthetase, mitochondrial; (InterPro:IPR004530); contains InterPro domain Ferredoxin-fold anticodon-binding; (InterPro:IPR005121); contains InterPro domain Aminoacyl-transfer RNA synthetase, class II; (InterPro:IPR006195) |
| 458 | 264781\_at | AT1G08540 | ATSIG1, SIG1, SIG2, SIGA, SIGB | 10 | 0 | -0.1 | 0.2 | 0 | 0 | -0.2 | -0.2 | -0.2 | -0.6 | -1 | -0.6 | -1.9 | -1.5 | -1.3 | -2.3 | -2.3 | -0.9 | -1.4 | -0.4 | -0.3 | -0.3 | 0.2 | -0.3 | -0.3 | -0.8 | -1.8 | 0 | -0.5 | Subunit of chloroplast RNA polymerase, confers the ability to recognize promoter sequences on the core enzyme, Subunit of chloroplast RNA polymerase, confers the ability to recognize promoter sequences on the core enzyme, Subunit of chloroplast RNA polymerase, confers the ability to recognize promoter sequences on the core enzyme, Subunit of chloroplast RNA polymerase, confers the ability to recognize promoter sequences on the core enzyme, Subunit of chloroplast RNA polymerase, confers the ability to recognize promoter sequences on the core enzyme |
| 457 | 251744\_at | AT3G56010 |  |  | 0 | 0.2 | 0.2 | 0.2 | -0.1 | -0.3 | 0 | -0.1 | -0.4 | -1.9 | -0.4 | -1.7 | -1.5 | -1 | -2.4 | -1.9 | -1.1 | -1.7 | -0.4 | -0.3 | -0.3 | 0.3 | -0.2 | -0.1 | -0.4 | -1.4 | 0.2 | -0.2 | similar to conserved hypothetical protein [Medicago truncatula] (GB:ABE94028.1) |
| 456 | 245952\_at | AT5G28500 |  | 1 | 0.1 | 0.1 | 0.2 | 0.2 | 0.1 | -0.5 | -0.1 | -0.2 | -0.4 | -1.5 | -0.4 | -1.4 | -1.1 | -1.1 | -3.1 | -2.2 | -0.9 | -2.2 | 0 | -0.2 | -0.3 | 0.3 | -0.2 | -0.1 | -0.8 | -1.4 | -0.2 | -0.5 | similar to unknown protein [Arabidopsis thaliana] (TAIR:AT3G04550.1); similar to Os10g0445600 [Oryza sativa (japonica cultivar-group)] (GB:NP\_001064712.1); similar to hypothetical protein Npun02004305 [Nostoc punctiforme PCC 73102] (GB:ZP\_00109308.1) |
| 455 | 266293\_at | AT2G29360 |  |  | 0.4 | 0.6 | 0.2 | 0.3 | 0.1 | 0.2 | -0.4 | -0.1 | -0.7 | -2.2 | -0.6 | -1.3 | -1.2 | -2 | -1.1 | -1.9 | -2.1 | -1.1 | -0.6 | -0.3 | -0.5 | 0.5 | -0.3 | -0.1 | -1.5 | -1.5 | -0.1 | -0.5 | tropinone reductase, putative / tropine dehydrogenase, putative; similar to tropinone reductase, putative / tropine dehydrogenase, putative [Arabidopsis thaliana] (TAIR:AT2G29150.1); similar to Tropinone reductase, putative, expressed [Oryza sativa (japonica cultivar-group)] (GB:ABF95188.1); contains InterPro domain Short-chain dehydrogenase/reductase SDR; (InterPro:IPR002198); contains InterPro domain Glucose/ribitol dehydrogenase; (InterPro:IPR002347) |
| 454 | 251183\_at | AT3G62630 |  |  | -0.1 | 0.4 | 0.3 | -0.2 | -0.1 | -0.4 | -0.4 | -0.3 | -0.4 | -1.6 | -0.9 | -1.7 | -1.6 | -2.2 | -3.3 | -2.5 | -2.5 | -2.1 | -0.8 | -0.7 | -1 | -0.2 | 0.1 | -0.1 | -0.7 | -1.4 | -0.8 | -0.6 | similar to calmodulin-binding protein [Arabidopsis thaliana] (TAIR:AT2G15760.1); similar to conserved hypothetical protein [Medicago truncatula] (GB:ABE84267.1); contains InterPro domain Protein of unknown function DUF1645; (InterPro:IPR012442) |
| 453 | 246237\_at | AT4G36390 |  |  | 0 | 0 | 0 | 0 | 0 | -0.4 | 0 | -0.1 | -0.5 | -1.2 | -0.2 | -1.5 | -1 | -1.2 | -1.6 | -1.4 | -1.4 | -1.3 | -0.4 | -0.4 | -0.5 | 0.2 | -0.1 | 0 | -0.4 | -1 | -0.1 | -0.2 | radical SAM domain-containing protein / TRAM domain-containing protein; Identical to CDK5RAP1-like protein [Arabidopsis Thaliana] (GB:Q8H0V1;GB:O23241); similar to radical SAM domain-containing protein / TRAM domain-containing protein [Arabidopsis thaliana] (TAIR:AT1G72090.1); similar to Os11g0592800 [Oryza sativa (japonica cultivar-group)] (GB:NP\_001068196.1); similar to similar to CDK5 regulatory subunit-associated protein [Nicotiana tabacum] (GB:BAD15110.2); contains InterPro domain Protein of unknown function UPF0004; (InterPro:IPR005839); contains InterPro domain Radical SAM; (InterPro:IPR007197); contains InterPro domain tRNA-i(6)A37 modification enzyme MiaB; (InterPro:IPR006463); contains InterPro domain Deoxyribonuclease/rho motif-related TRAM; (InterPro:IPR002792); contains InterPro domain Elongator protein 3/MiaB/NifB; (InterPro:IPR006638) |
| 452 | 257717\_at | AT3G18390 | EMB1865 | 1 | 0.1 | 0.1 | 0.3 | -0.3 | 0 | -0.2 | -0.2 | -0.1 | -0.1 | -2 | -0.3 | -1.5 | -1.4 | -1.7 | -2.5 | -1.9 | -1.1 | -2 | -0.1 | -0.5 | -0.4 | 0.2 | -0.1 | 0.2 | -0.7 | -1.5 | -0.3 | -0.3 | EMB1865 (EMBRYO DEFECTIVE 1865); similar to unknown protein [Arabidopsis thaliana] (TAIR:AT3G23070.1); similar to Protein of unknown function UPF0044 [Medicago truncatula] (GB:ABE83057.1); similar to Os05g0551900 [Oryza sativa (japonica cultivar-group)] (GB:NP\_001056251.1); contains InterPro domain CRS1/YhbY; (InterPro:IPR001890) |
| 451 | 260370\_at | AT1G69740 | HEMB1, HEMB1 | 3 | 0.3 | 0.1 | 0.2 | 0.1 | -0.1 | -0.2 | 0 | -0.3 | -0.2 | -1.9 | -0.3 | -0.3 | -0.2 | -1.6 | -1.6 | -0.6 | -1.1 | -0.9 | 0 | -0.3 | -0.3 | 0.1 | -0.1 | -0.1 | 0.2 | -0.1 | 0.1 | -0.1 | HEMB1; Identical to Delta-aminolevulinic acid dehydratase, chloroplast precursor (EC 4.2.1.24) (Porphobilinogen synthase) (ALADH) [Arabidopsis Thaliana] (GB:Q9SFH9); similar to HEMB2, porphobilinogen synthase [Arabidopsis thaliana] (TAIR:AT1G44318.1); similar to Delta-aminolevulinic acid dehydratase, chloroplast precursor (Porphobilinogen synthase) (ALADH) (GB:P43210); similar to aminolevulinate dehydratase (GB:2114378A); similar to aminolevulinate dehydratase [Raphanus sativus] (GB:AAK15323.1); contains InterPro domain Delta-aminolevulinic acid dehydratase; (InterPro:IPR001731), HEMB1; porphobilinogen synthase; Identical to Delta-aminolevulinic acid dehydratase, chloroplast precursor (EC 4.2.1.24) (Porphobilinogen synthase) (ALADH) [Arabidopsis Thaliana] (GB:Q9SFH9); similar to HEMB2, porphobilinogen synthase [Arabidopsis thaliana] (TAIR:AT1G44318.1); similar to Delta-aminolevulinic acid dehydratase, chloroplast precursor (Porphobilinogen synthase) (ALADH) (GB:P43210); similar to aminolevulinate dehydratase (GB:2114378A); similar to aminolevulinate dehydratase [Raphanus sativus] (GB:AAK15323.1); contains InterPro domain Delta-aminolevulinic acid dehydratase; (InterPro:IPR001731) |
| 450 | 250281\_at | AT5G13240 |  |  | 0.1 | -0.1 | 0 | 0.6 | 0.3 | 0 | 0.2 | -0.4 | -0.8 | -0.7 | -0.1 | -0.7 | -0.5 | 0 | -0.5 | -0.5 | -1.6 | -1.4 | -0.2 | 0 | 0 | 0.1 | -0.1 | -0.1 | 0.3 | -0.1 | -0.1 | 0.1 | similar to conserved hypothetical protein [Medicago truncatula] (GB:ABE92223.1); contains domain FAMILY NOT NAMED (PTHR22504); contains domain gb def: Hypothetical protein T31B5\_60 (PTHR22504:SF5) |
| 449 | 244988\_s\_at | ATCG00840, ATCG01300 ATCG00840, ATCG01300 | RPL23, RPL23.1, RPL23.2 | 1 | 0.3 | -0.1 | 0 | 0 | 0.1 | 0 | 0 | -0.1 | -0.3 | 0 | -0.2 | -1.7 | -1 | -1.4 | -0.6 | -0.6 | -1 | -0.8 | 0 | 0.2 | 0.1 | 0 | 0 | -0.2 | 0.1 | 0.1 | -0.1 | 0 | One of two chloroplast genes that encode chloroplast ribosomal protein L23, a constituent of the large subunit of the ribosomal complex, One of two chloroplast genes that encode chloroplast ribosomal protein L23, a constituent of the large subunit of the ribosomal complex, One of two chloroplast genes that encode chloroplast ribosomal protein L23, a constituent of the large subunit of the ribosomal complex |
| 448 | 253421\_at | AT4G32340 |  |  | -0.8 | 0.1 | -0.8 | 0.1 | 0.2 | -1.1 | 0.3 | -0.1 | -1.3 | -3 | -0.1 | -1.4 | -0.6 | 0.2 | -2.1 | -1.2 | -1.9 | -2.5 | -0.6 | -0.1 | -1.2 | 0.1 | -0.3 | -1 | -0.8 | -1.5 | -0.1 | -0.6 | binding; similar to binding [Arabidopsis thaliana] (TAIR:AT1G80130.1); similar to Os06g0606700 [Oryza sativa (japonica cultivar-group)] (GB:NP\_001058025.1); similar to Tetratricopeptide-like helical [Medicago truncatula] (GB:ABE92034.1); contains InterPro domain Tetratricopeptide-like helical; (InterPro:IPR011990) |
| 447 | 245362\_at | AT4G17460 | HAT1 | 3 | -1.6 | 0.9 | -3.2 | -0.2 | 0.9 | -3.9 | 0.5 | 0 | -0.5 | -5.2 | 0.1 | -4.5 | -3.1 | 0.7 | -5.8 | -4.9 | -2.9 | -4.3 | 0.1 | -0.1 | -0.5 | -1.2 | 0.9 | -2 | -0.5 | -2.1 | -0.5 | -0.2 | Encodes homeobox protein HAT1. |
| 446 | 264054\_at | AT2G22540 | AGL22, SVP | 10 | -0.2 | 0.2 | -0.2 | 0.5 | 0.1 | -0.1 | 0.7 | -0.3 | -0.6 | -0.9 | -0.2 | -0.7 | -0.4 | 0.3 | -0.9 | -1.6 | -1.4 | -0.7 | -0.1 | 0.1 | 0.2 | -0.1 | -0.2 | -0.6 | -0.3 | -1.2 | -0.1 | 0.2 | Encodes a nuclear protein that acts as a floral repressor and that functions within the thermosensory pathway. It represses FT expression via direct binding to the vCArG III motif in the FT promoter., Encodes a nuclear protein that acts as a floral repressor and that functions within the thermosensory pathway. It represses FT expression via direct binding to the vCArG III motif in the FT promoter. |
| 445 | 245795\_at | AT1G32160 |  |  | -0.1 | -0.1 | -0.2 | -0.1 | 0 | -0.5 | 0 | -0.2 | -0.4 | -0.6 | -0.3 | -1.1 | -0.6 | -0.1 | -0.8 | -1.2 | -1.5 | -0.8 | -0.3 | -0.1 | -0.5 | -0.1 | -0.2 | -0.4 | -0.2 | -0.8 | -0.2 | 0 | similar to unknown protein [Arabidopsis thaliana] (TAIR:AT3G17800.2); similar to unknown protein [Oryza sativa (japonica cultivar-group)] (GB:AAO38836.1); similar to Os03g0717900 [Oryza sativa (japonica cultivar-group)] (GB:NP\_001051084.1); similar to Os04g0595100 [Oryza sativa (japonica cultivar-group)] (GB:NP\_001053731.1); contains InterPro domain Protein of unknown function DUF760; (InterPro:IPR008479) |
| 444 | 262162\_at | AT1G78020 |  |  | -0.6 | 0 | 0.7 | 1 | -0.1 | -1.1 | -0.4 | -0.3 | -0.9 | -1.4 | -0.3 | -2.4 | -1.7 | 1.1 | -2.8 | -1.4 | -1.5 | -1 | -0.4 | 0.6 | -0.2 | -1.3 | -2.2 | -0.1 | -0.4 | -2.6 | -1.3 | -0.5 | senescence-associated protein-related; similar to senescence-associated protein-related [Arabidopsis thaliana] (TAIR:AT1G22160.1); similar to Os06g0223700 [Oryza sativa (japonica cultivar-group)] (GB:NP\_001057184.1); similar to unknown protein [Oryza sativa (japonica cultivar-group)] (GB:BAD25910.1); similar to Os08g0407600 [Oryza sativa (japonica cultivar-group)] (GB:NP\_001061776.1); contains InterPro domain Protein of unknown function DUF581; (InterPro:IPR007650) |
| 443 | 256168\_at | AT1G51805 |  |  | -1 | 0.2 | 0.5 | 0.1 | 0 | -2.1 | -0.4 | 0.1 | -0.4 | -1 | -0.1 | -1.5 | -0.9 | 0.1 | -2.7 | -1.8 | -1.9 | -0.9 | -0.7 | 0.2 | -0.2 | -0.2 | -0.4 | -0.8 | 0.2 | -1.2 | 0.1 | 0 | leucine-rich repeat protein kinase, putative; similar to leucine-rich repeat protein kinase, putative [Arabidopsis thaliana] (TAIR:AT3G21340.1); similar to leucine-rich repeat protein kinase, putative [Arabidopsis thaliana] (TAIR:AT1G51850.1); similar to leucine-rich repeat protein kinase, putative [Arabidopsis thaliana] (TAIR:AT1G51820.1); similar to Protein kinase [Medicago truncatula] (GB:ABE90329.1); similar to Protein kinase [Medicago truncatula] (GB:ABE90342.1); similar to leaf senescence-associated receptor-like protein kinase [Phaseolus vulgaris] (GB:AAG00510.1); contains InterPro domain Serine/threonine protein kinase; (InterPro:IPR002290); contains InterPro domain Protein kinase-like; (InterPro:IPR011009); contains InterPro domain Leucine-rich repeat; (InterPro:IPR001611); contains InterPro domain Protein kinase; (InterPro:IPR000719); contains InterPro domain Leucine-rich repeat, plant specific; (InterPro:IPR007090); contains InterPro domain Serine/threonine protein kinase, active site; (InterPro:IPR008271) |
| 442 | 255016\_at | AT4G10120 | ATSPS4F | 2 | -2.2 | -0.1 | 0.3 | 0.6 | -0.6 | -1.8 | -0.5 | 0.1 | 0.2 | -2.6 | -0.4 | -0.9 | 0 | 1.8 | -1.2 | -2.6 | -2.3 | 0.1 | -0.3 | -0.2 | -1 | -0.4 | -0.6 | -2.7 | -0.4 | -2.8 | 0.4 | -0.8 | Encodes a protein with putative sucrose-phosphate synthase activity. |
| 441 | 262473\_at | AT1G50250 | FTSH1 | 12 | -0.8 | -0.3 | 0 | 0.3 | 0 | 0.2 | 0 | -0.2 | -0.3 | -0.2 | 0 | -0.8 | -0.7 | -0.1 | -0.2 | -1.7 | -1.1 | -0.9 | -0.6 | -0.2 | -0.3 | 0.1 | -0.2 | -0.3 | 0 | -0.4 | 0.4 | 0.2 | encodes an FTSH protease that is localized to the chloroplast. Involved in the D1 repair cycle of Photosystem II. FtsH1 and FtsH5 are interchangeable in thylakoid membranes. |
| 440 | 251929\_at | AT3G53920 | SIG3, SIGC | 4 | -0.9 | -0.3 | -0.3 | 0.2 | 0 | 0 | -0.2 | -0.2 | -0.3 | -0.7 | -0.3 | -1.2 | -0.9 | -0.3 | -0.7 | -1.5 | -1.6 | -0.8 | -0.8 | -0.4 | -0.3 | 0 | -0.6 | -0.7 | -0.5 | -2.1 | 0.1 | 0 | Encodes a sigma-like transcription factor, Sigma 3 (SIG3 or SIGC). As a subunit of chloroplast RNA polymerase, SIG3 confers the ability to recognize promoter sequences on the core enzyme. SIG3 transcribes specifically the psbN gene in plastids., Encodes a sigma-like transcription factor, Sigma 3 (SIG3 or SIGC). As a subunit of chloroplast RNA polymerase, SIG3 confers the ability to recognize promoter sequences on the core enzyme. SIG3 transcribes specifically the psbN gene in plastids. |
| 439 | 267152\_at | AT2G31040 |  |  | -0.8 | -0.1 | -0.1 | 0.1 | 0 | 0.1 | 0 | -0.3 | -0.6 | -0.8 | -0.3 | -1.1 | -1.1 | -0.1 | -1.8 | -1.3 | -1.4 | -1.2 | -0.4 | -0.8 | -0.2 | -0.2 | -0.2 | -0.2 | 0 | -1 | 0.1 | -0.1 | ATP synthase protein I -related; similar to hypothetical protein MtrDRAFT\_AC135231g19v1 [Medicago truncatula] (GB:ABE82903.1) |
| 438 | 266925\_at | AT2G45740 | PEX11D | 5 | -0.8 | -0.1 | 0 | 0 | 0 | 0.5 | 0 | -0.1 | -0.4 | -0.3 | -0.1 | -1 | -0.9 | -0.2 | -0.5 | -0.9 | -1.4 | -0.4 | -0.4 | -0.2 | -0.5 | -0.2 | -0.5 | -0.7 | -0.4 | -1.1 | -0.1 | -0.2 | member of the peroxin11 (PEX11) gene family, integral to peroxisome membrane, controls peroxisome proliferation. |
[truncated: 317,478 more chars]
