# Supplementary material for: Transcriptional responses to polycyclic aromatic hydrocarbon-induced stress in Arabidopsis thaliana reveal the involvement of hormone and defense signaling pathways
Source: BMC Plant Biol. 2010 Apr 7;10:59. doi: 10.1186/1471-2229-10-59 (PMC2923533; doi:10.1186/1471-2229-10-59)
Supplement: Additional file 7 — Microarray quality control analysis. This file contains a quality control analysis of the raw microarray data used in this study. The analysis was produced using the Bioconductor package arrayQualityMetrics. Jun04 no phe.cel Jun04 phe.cel represent the untreated control and phenanthrene-treated samples, respectively, of the first replicate experiment. From the second replicate experiment, Aug04_no_phe_A.cel and Aug04_no_phe_C.cel represent the control, and Aug04_phe_B.cel represents the treated sample. [file 1471-2229-10-59-S7.PDF]

ab quality metrics report

Summary

Array #	Array Name	MA-plot	Spatial distribution	Boxplots/Density plots	Heatmap	RLE	NUS
1	Aug04_no_phe_A.cel						
2	Aug04_no_phe_C.cel						
3	Aug04_phe_B.cel						*
4	Jun04_no_phe.cel						*
5	Jun04_phe.cel		*				

*array identified as having a potential problem or as being an outlier.

Index

- **Individual array quality**
  - MAplot
  - Spatial distribution of features intensites
  - Row-Column effect
- **Homogeneity between arrays**
  - Boxplots
  - Density plots
- **Between array comparison**
- **Variance mean dependency**
- **Affymetrix specific plots**
  - RNA degradation plot
  - RLE
  - NUSE
  - Affymetrix QC stats
  - PM MM plot

Section 1: Individual array quality

MA plots

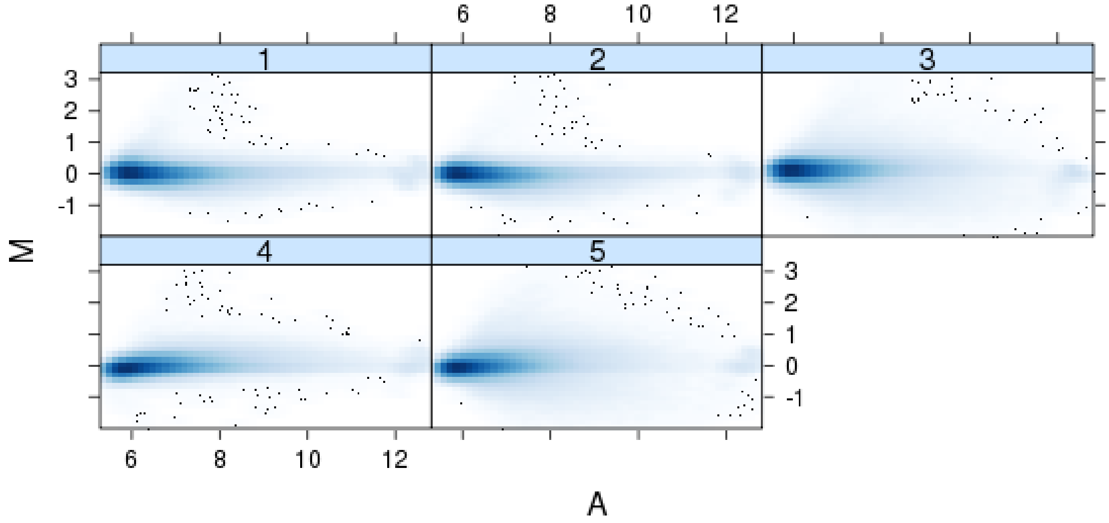

Figure 1

Figure 1 represents MA plot for each array. M and A are defined as :
$M = \log_2(I_1) - \log_2(I_2)$
$A = 1/2\ (\log_2(I_1)+\log_2(I_2))$
where I_1 is the intensity of the array studied and I_2 is the intensity of a "pseudo"-array, which have the median values of all the arrays. Typically, we expect the mass of the distribution in an MA plot to be concentrated along the M = 0 axis, and there should be no trend in the mean of M as a function of A. Note that a bigger width of the plot of the M-distribution at the lower end of the A scale does not necessarily imply that the variance of the M-distribution is larger at the lower end of the A scale: the visual impression might simply be caused by the fact that there is more data at the lower end of the A scale. To visualize whether there is a trend in the variance of M as a function of A, consider plotting M versus rank(A).

Spatial distribution of features intensities

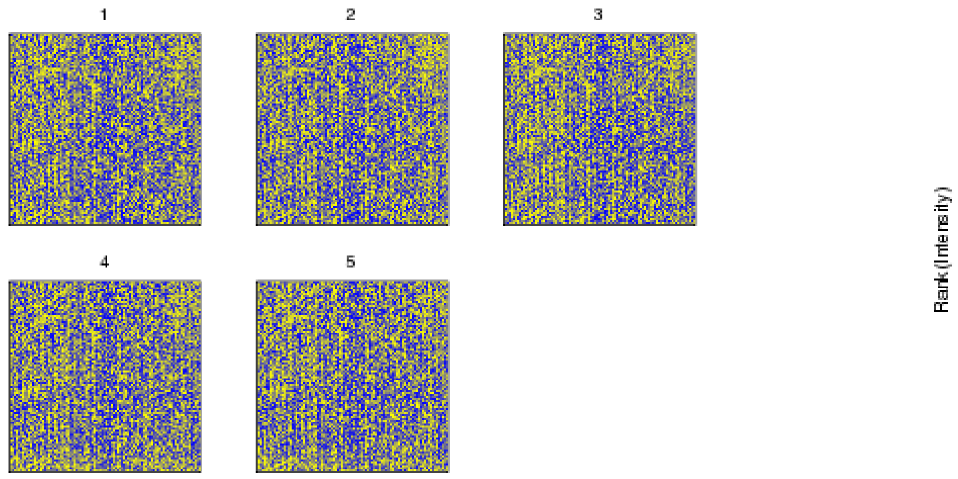

Figure 2

Figure 2: False color representations of the arrays' spatial distributions of feature intensities. The color scale is shown in the panel on the right, and it is proportional to the ranks. These plots may help in identifying patterns that may be caused, for example, spatial gradients in the hybridization chamber, air bubbles, spotting or plating problems.

Row-Column effect

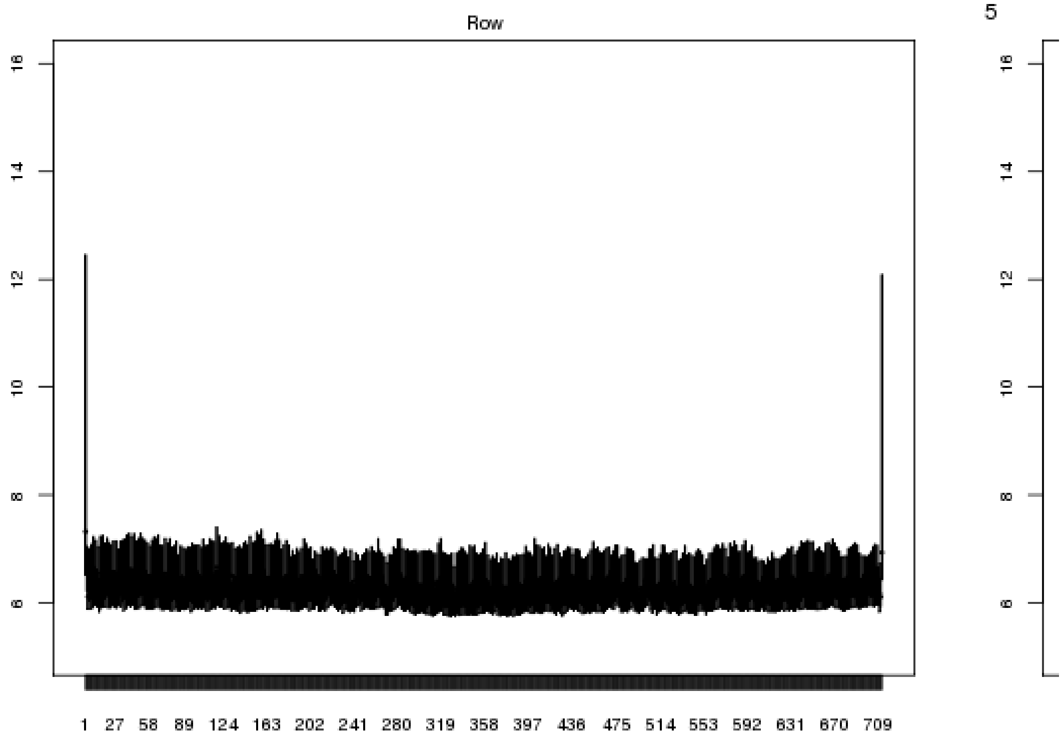

Figure 3

Figure 3 shows the boxplots of the $\log_2$ intensities grouped by row (left panel) and column (right panel) of the array. If there is no spatial effect, the boxes should be homogeneous in wide and y position.

Section 2: Homogeneity between arrays

Boxplots

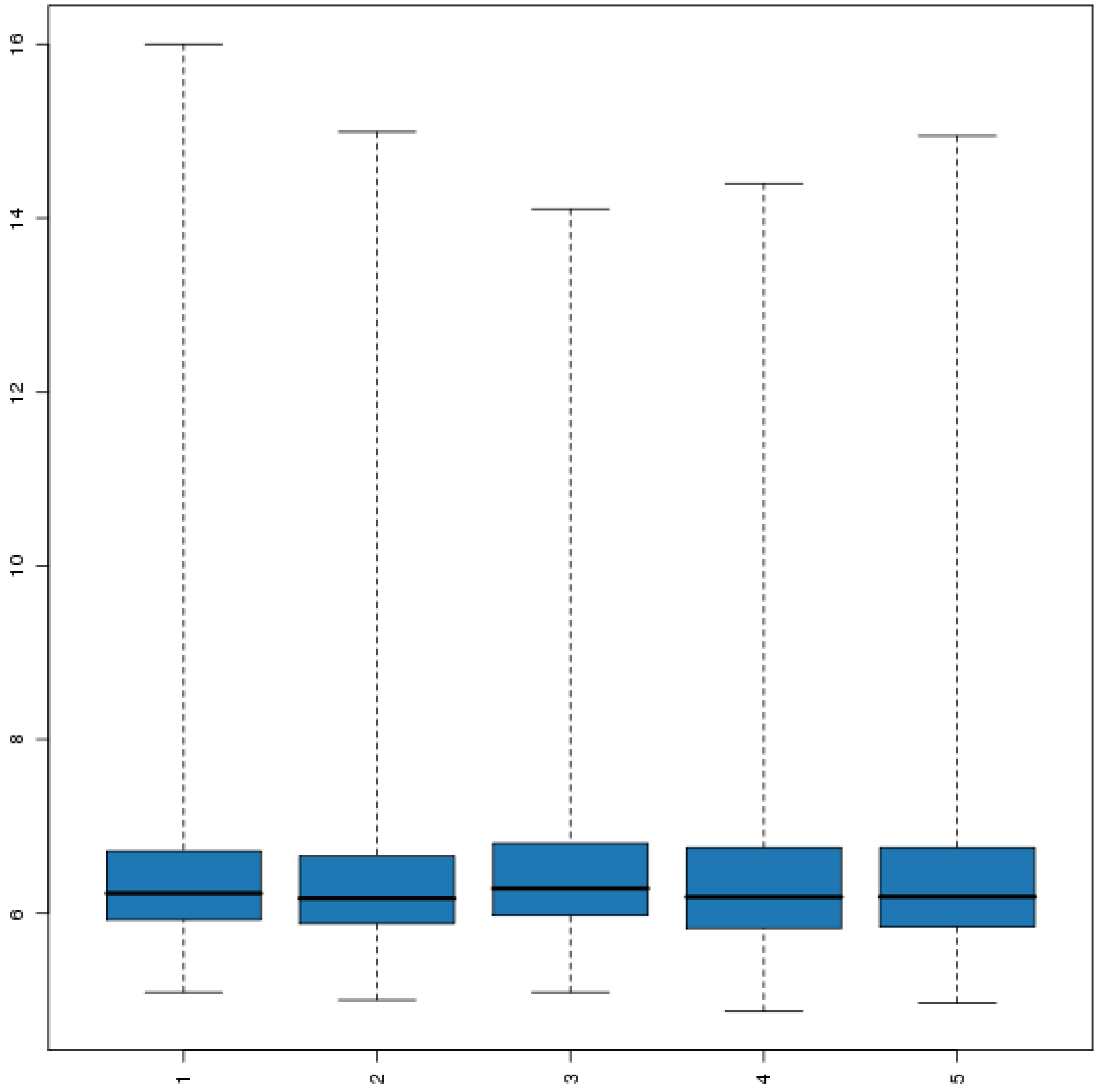

Figure 4

Figure 4 presents boxplots of the $\log_2$(Intensities). Each box corresponds to one array. If the arrays are homogeneous, the boxes should have similar wides and y position.

Density plots

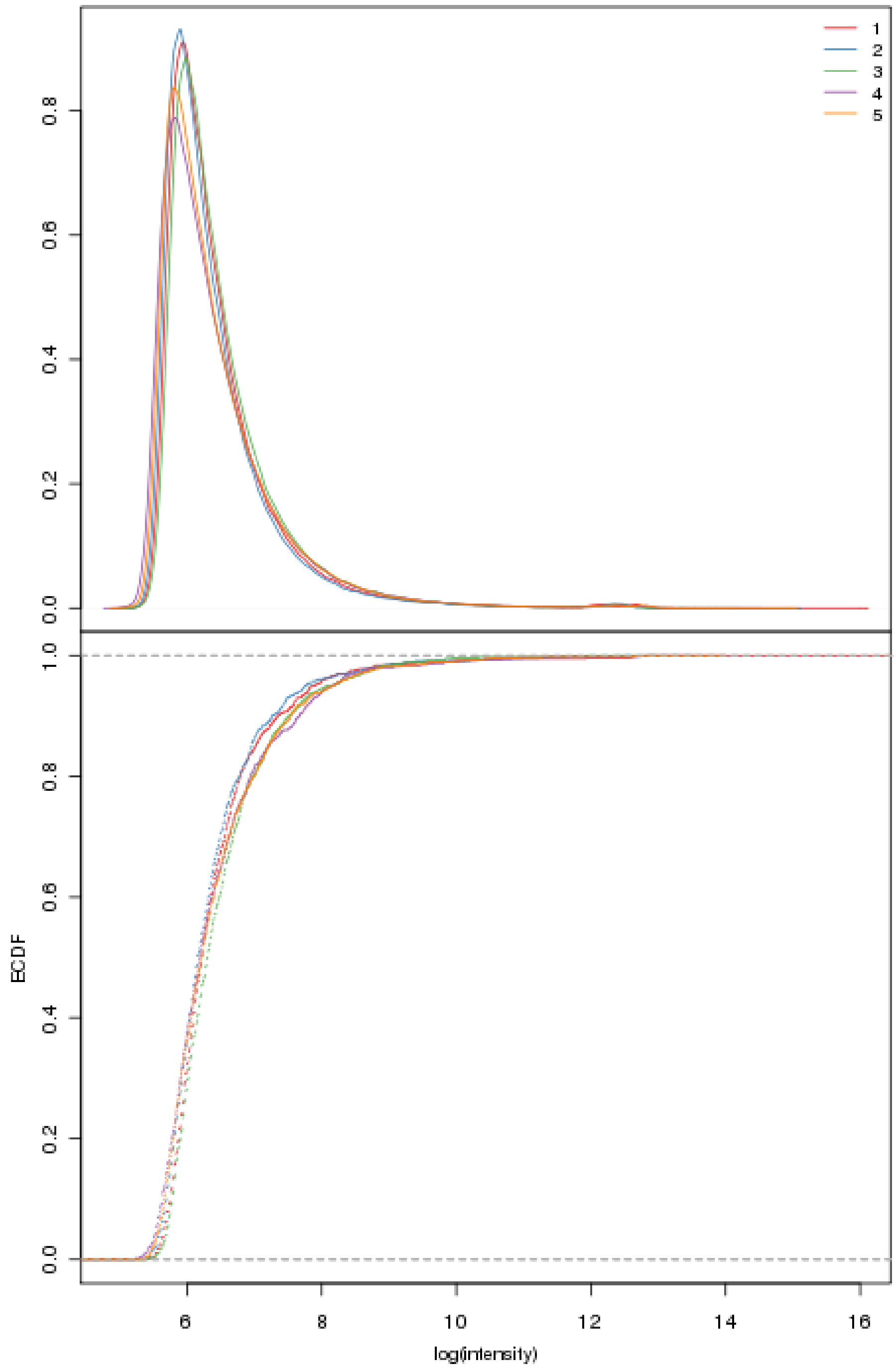

Figure 5

Figure 5 shows density estimates (histograms) of the data. Arrays whose distributions are very different from the others should be considered for possible problems.

Section 3: Between array comparison

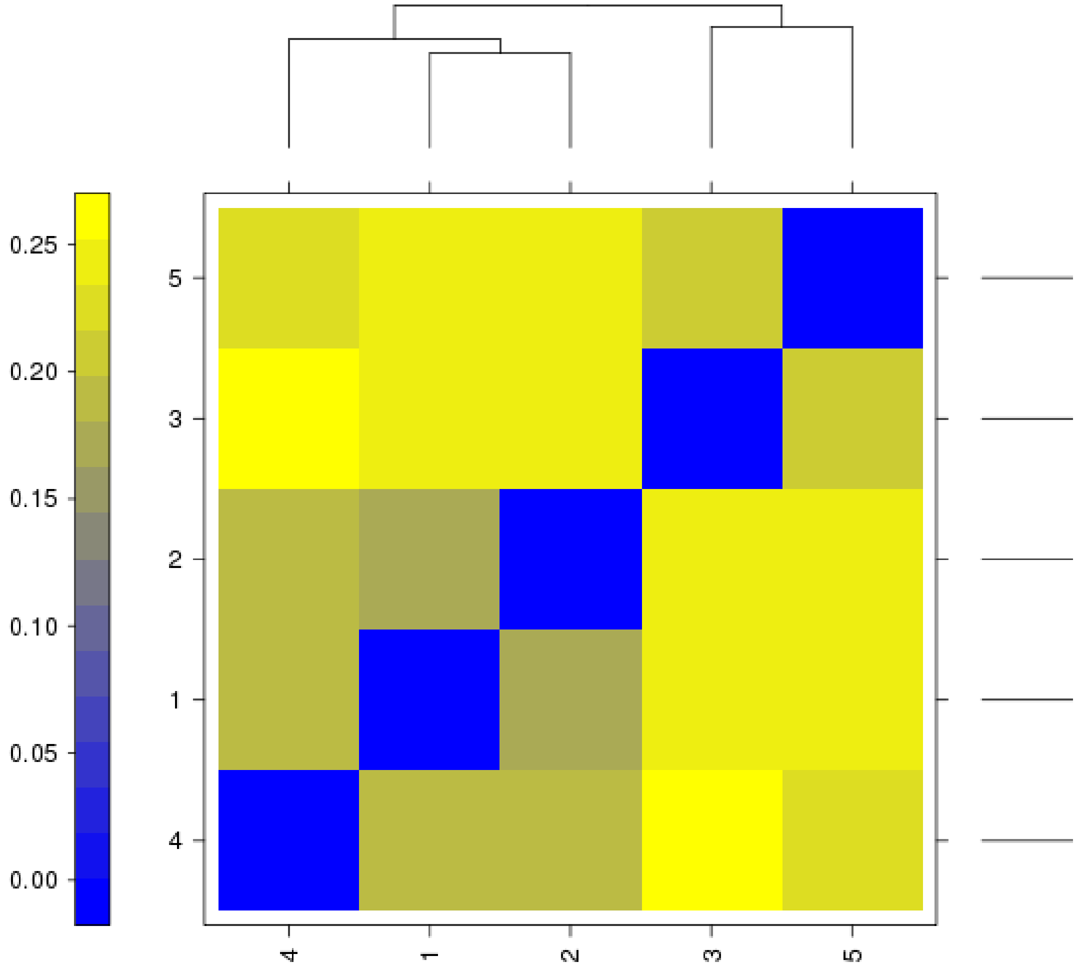

Heatmap representation of the distance between arrays

Figure 6

Figure 6 shows a false color heatmap of between arrays distances, computed as the median absolute difference of the M-value for each pair of arrays.

$$d_{xy} = median|M_{xi}-M_{yi}|$$

Here, M_{xi} is the M-value of the i-th probe on the x-th array, without preprocessing.
This plot can serve to detect outlier arrays.
Consider the following decomposition of M_{xi}: $M_{xi} = z_i + \beta_{xi} + \varepsilon_{xi}$, where z_i is the probe effect for probe i (the same across all arrays), ε_{xi} are i.i.d. random variables with mean zero and β_{xi} is such that for any array x, the majority of values β_{xi} are negligibly small (i. e. close to zero). β_{xi} represents differential expression effects. In this model, all values d_{xy} are (in expectation) the same, namely 2 times the standard deviation of ε_{xi} . Arrays whose distance matrix entries are way different give cause for suspicion. The dendrogram on this plot also can serve to check if, without any probe filtering, the arrays cluster accordingly to a biological meaning.

Section 4: Variance mean dependency

Standard deviation versus rank of the mean

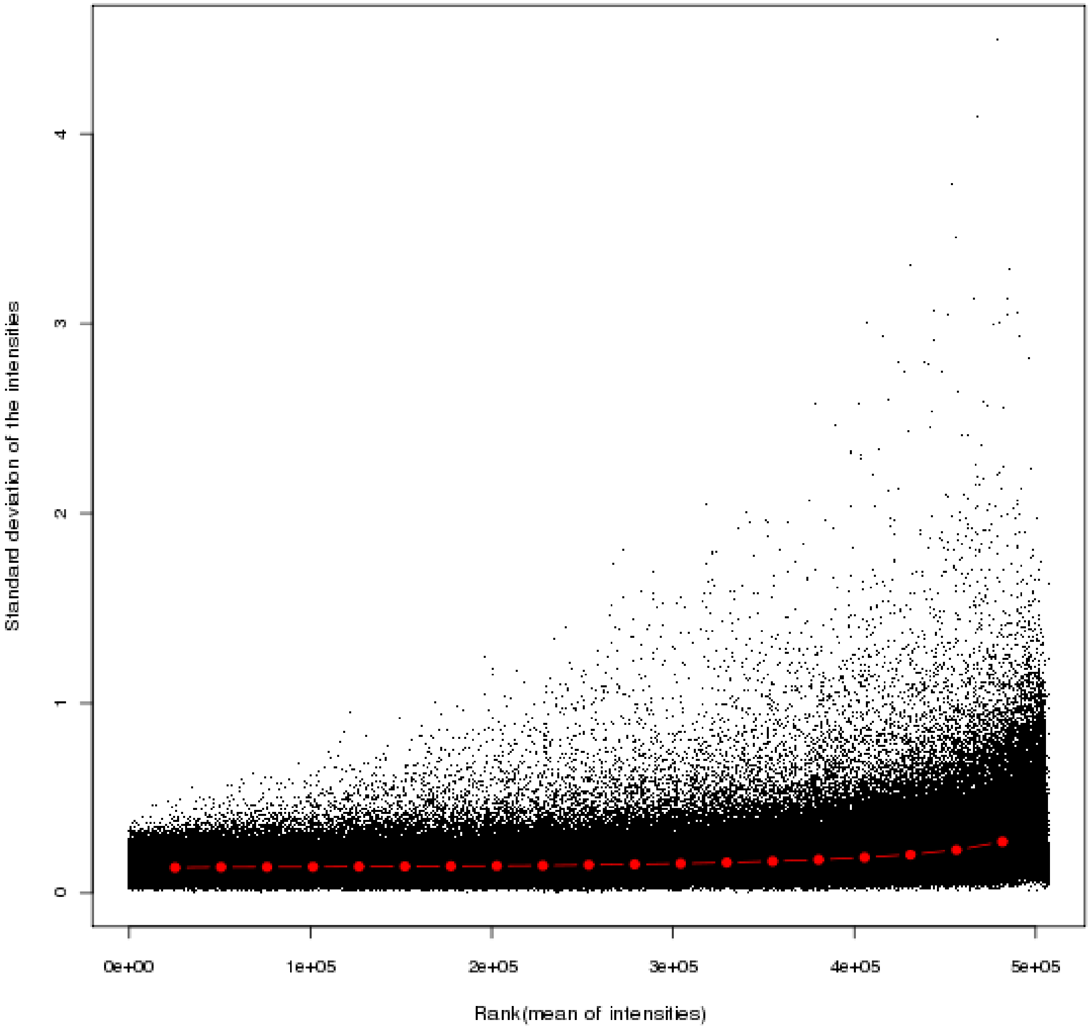

Figure 7

For each feature, the plot on **Figure 7** shows the empirical standard deviation of the intensities of all the arrays on the *y*-axis versus the rank of the mean of intensities of the arrays on the *x*-axis. The red dots, connected by lines, show the running median of the standard deviation. After vsn normalization, this should be approximately horizontal, that is, show no substantial trend.

Section 5: Affymetrix specific plots

RNA degradation plot

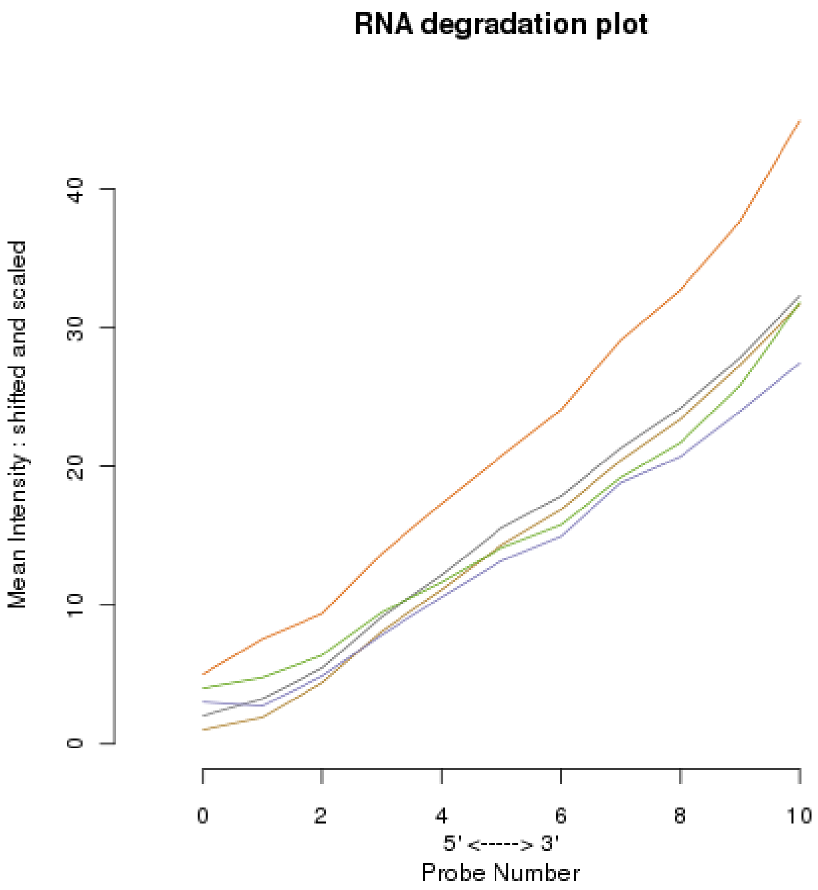

Figure 8

RLE plot

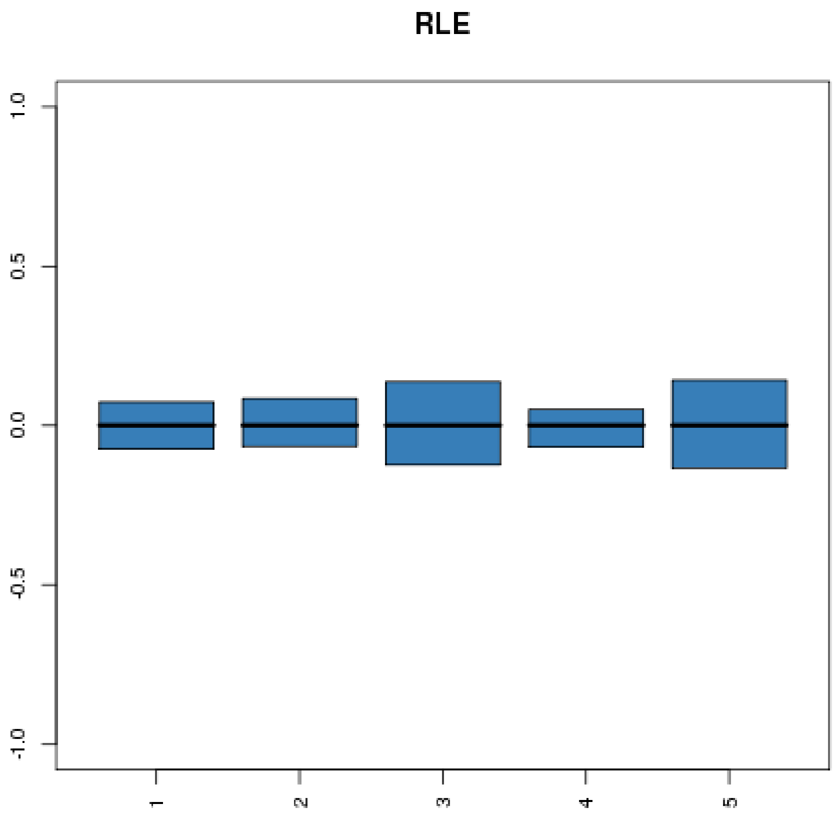

Figure 9

NUSE plot

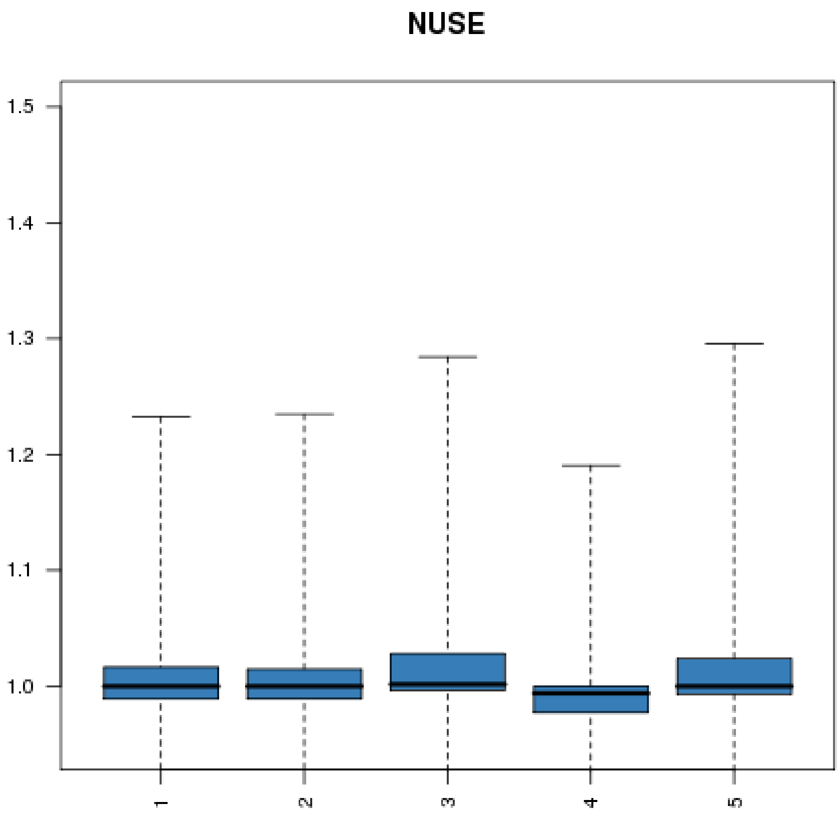

Figure 10

Diagnostic plot recommended by Affymetrix

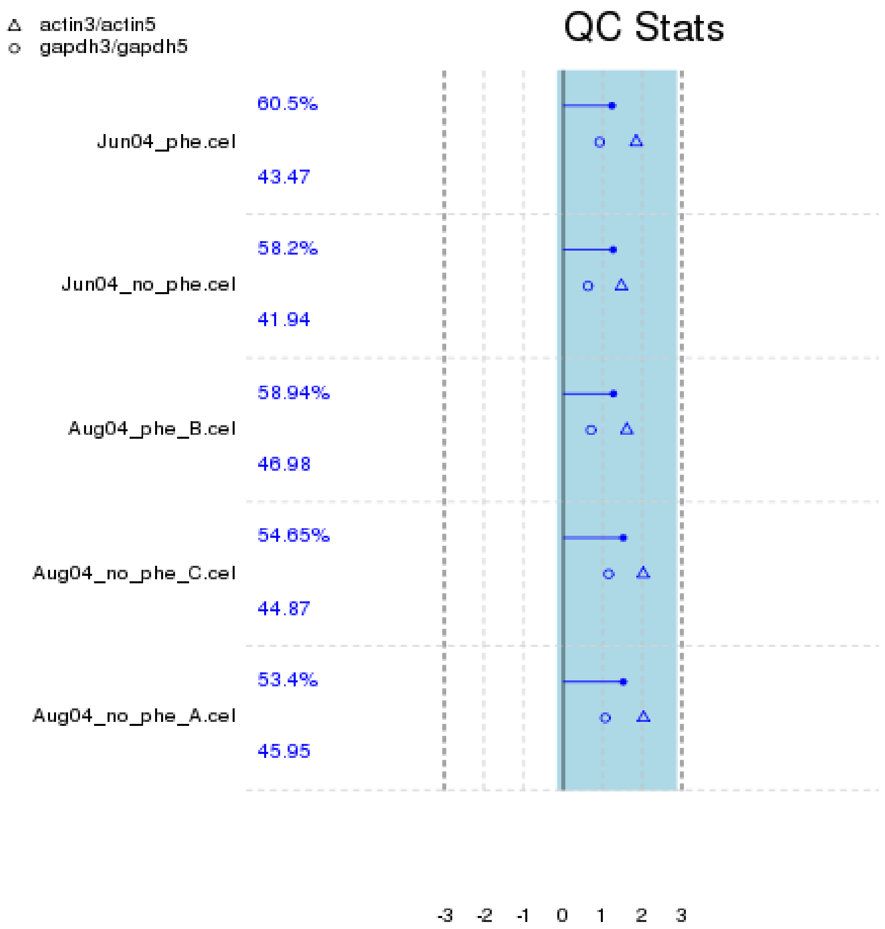

Figure 11

In this section we present diagnostic plots based on tools provided in the affyPLM package. In **Figure 8** a RNA digestion plot is computed on normalized data (so that standard deviation is equal to 1). In this plot each array is represented by a single line. It is important to identify any array(s) that has a slope which is very different from the others. The indication is that the RNA used for that array has potentially been handled quite differently from the other arrays. **Figure 9** is a Relative Log Expression (RLE) plot and an array that has problems will either have larger spread, or will not be centered at M = 0, or both. **Figure 10** is a Normalized Unscaled Standard Error (NUSE) plot. Low quality arrays are those that are substantially elevated or more spread out, relative to the other arrays. NUSE values are not comparable across data sets. Both RLE and NUSE are performed on preprocessed data (background correction and quantile normalization). **Figure 11** represents the diagnostic plot recommended by Affymetrix. It is fully describe in the simpleaffy.pdf vignette of the package simpleaffy. Any metrics that is shown in red is out of the manufacturer's specific boundaries and suggests a potential problem, any metrics shown in blue is fine.

Perfect matchs and mismatchs

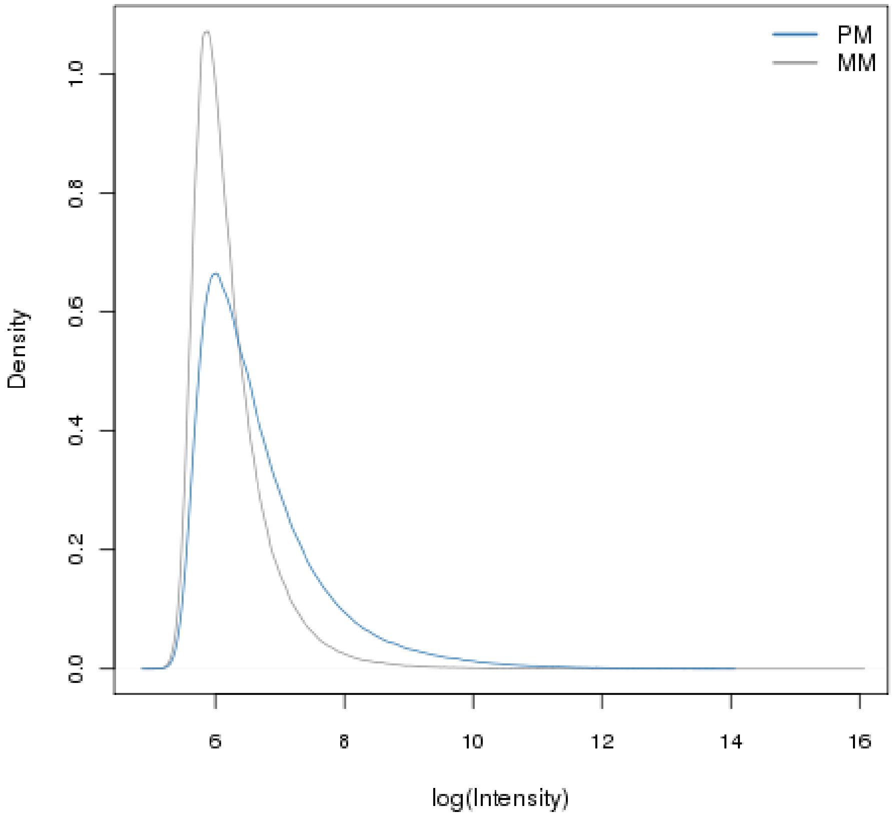

Figure 12

Figure 12 shows the density distributions of the $\log_2$ intensities grouped by the matching of the probes. Blue, density estimate of intensities of perfect match probes (PM) and gray the mismatch probes (MM). We expect that, MM probes having poorer hybridization than PM probes, the PM curve should be shifted to the right of the MM curve.

This report has been created with arrayQualityMetrics 1.6.1 under R version 2.7.1 (2008-06-23)
