# Supplementary material for: Transcriptional responses to polycyclic aromatic hydrocarbon-induced stress in Arabidopsis thaliana reveal the involvement of hormone and defense signaling pathways
Source: BMC Plant Biol. 2010 Apr 7;10:59. doi: 10.1186/1471-2229-10-59 (PMC2923533; doi:10.1186/1471-2229-10-59)
Supplement: Additional file 8 — Microarray volcano plot. The volcano plot represents the dataset from the five microarray chips after gcRMA normalization and linear model processing by the Bioconductor limma package. [file 1471-2229-10-59-S8.PDF]

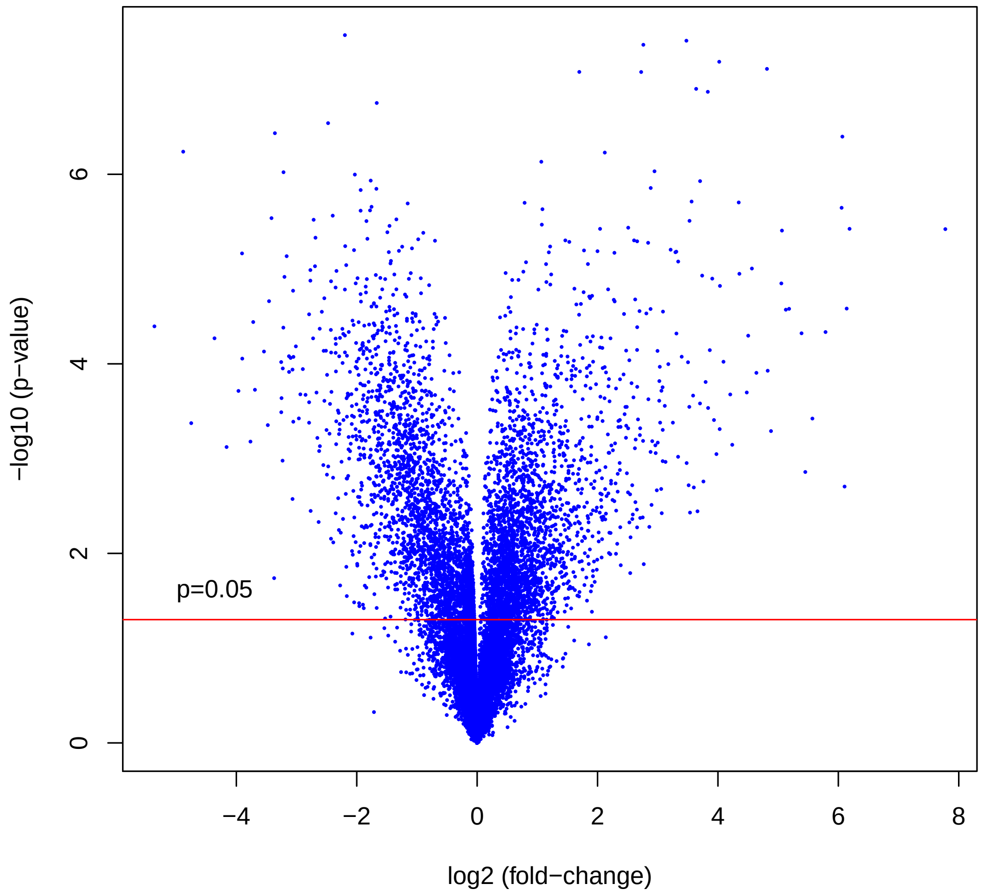

The volcano plot represents the full set of microarray data. The fold-change and *p*-values were generated by the Limma linear model.

The large number of statistically-significant probes (points above the red line) supports that the biological effect of phenanthrene was substantial. In addition, the existence of many statistically-significant probes with fold-change near zero indicates low variance across the replicates.
