# Supplementary material for: Transcriptional responses to polycyclic aromatic hydrocarbon-induced stress in Arabidopsis thaliana reveal the involvement of hormone and defense signaling pathways
Source: BMC Plant Biol. 2010 Apr 7;10:59. doi: 10.1186/1471-2229-10-59 (PMC2923533; doi:10.1186/1471-2229-10-59)
Supplement: Additional file 9 — Minimum information about a microarray experiment (MIAME) checklist. The minimum information about a microarray experiment (MIAME) data is supplied in Additional File 9. [file 1471-2229-10-59-S9.RTF]

MIAME Checklist
Experiment Design:

	Authors: 
	
David Weisman, Adán Colón-Carmona:
	Department of Biology
University of Massachusetts Boston
100 Morrissey Blvd.
Boston, MA 02125
USA

	Merianne Alkio:
	Institute of Biological Production Systems
Fruit Science Section
Leibniz University Hannover
Herrenhäuser Str. 2, D-30419 Hannover
Germany

	 Type of experiment: 
 Measurement of Arabidopsis thaliana response to phenanthrene. 

	Experimental factors: 
Phenanthrene treatment 

	The number of hybridizations performed in the experiment:
5

	Hybridization design: 
Two independent biological replicate experiments were performed, with an additional technical replicate microarray measurement of Pool 3. 

Chip ID	Experimental Condition	Biological/RNA sample ID	
rep_1_no_phe_A	control	Pool 1	
rep_1_phe_B	phenanthrene treatment 	Pool 2	
rep_2_no_phe_A	control	Pool 3	
rep_2_no_phe_B	control	Pool 3	
rep_2_phe_C 	phenanthrene treatment	Pool 4	


	Quality control steps taken: 
Prior to hybridization, RNA quantity and quality were measured using an Agilent Technologies 2100 Bioanalyzer.  

Standard Affymetrix spike-in hybridization  controls were added.

	URLs and database accession numbers: 
We are in the process of submitting our data to NCBI/GEO.  Accession numbers will be provided prior to publication.


Samples used, extract preparation and labeling: 

	The origin of the biological sample:
Seeds of the Arabidopsis ecotype Colombia (WT) were obtained from Arabidopsis Research Centre.

	Manipulation of biological samples and protocols used:
Seeds were surface-sterilized, stratified, and placed in Petri dishes containing half-strength Murashige and Skoog medium, supplemented with sucrose and 0 (control) or 0.25 mM of phenanthrene.  Plants were grown at 23±1 °C under long-day conditions (16/8 h photoperiod at approximately 130 µmol photons m-2 s-1) for 21 d.   At least 20 whole plants were pooled and stored at -80 °C. 

	Protocol for preparing the hybridization extract: 
500mg tissue was removed from each pool and total RNA was isolated using TRIzol (Molecular Research Center) per the manufacturer's instructions. Resulting samples were treated with DNase I (Invitrogen) and purified with RNeasy Mini Cleanup (Qiagen) per the manufacturers' instructions.   cDNA was synthesized per the Affymetrix GeneChip Expression Analysis Technical Manual, and labeling was performed with the Enzo Bioarray HighYield RNA Transcript Labeling Kit.

Hybridization procedures and parameters:  
	Standard Affymetrix hybridization protocols were performed per the Affymetrix GeneChip Expression Analysis Technical Manual.
	 Washing and staining were performed on an Affymetrix Fluidics Station Model 450 using the EukGE-ENZO_18uM_450 protocol.

Measurement data and specifications: 
	Hybridized chips were read on an Affymetrix Scanner Type M10.

Array Design:
	Affymetrix ATH1-121501.

Post-processing:
	The five .CEL files were normalized as a batch by the Bioconductor just.gcrma algorithm using default parameters, resulting in log2-transformed expression values.
